# Synthesis of mono- and di-(*C*-glycopyranosyl-isoxazol(in)yl) derivatives on aromatic scaffolds and their evaluation as anti-adherence agents of *Candida albicans*

**DOI:** 10.3389/fchem.2026.1794047

**Published:** 2026-04-09

**Authors:** Tímea Kaszás, Bence Szakács, Tekla Blága, Kyle Doherty, Rachel Keenan-Dillon, Shauna Reynolds, Kevin Kavanagh, Trinidad Velasco-Torrijos, László Somsák, Marietta Tóth

**Affiliations:** 1 Department of Organic Chemistry, University of Debrecen, Debrecen, Hungary; 2 Doctoral School of Chemistry, University of Debrecen, Debrecen, Hungary; 3 Department of Chemistry, Maynooth University, Maynooth, Ireland; 4 Department of Biology, Maynooth University, Maynooth, Ireland

**Keywords:** 1,2,3-triazole, 1,3-dipolar cycloaddition, anti-adhesive glycoconjugates, *Candida albicans*, glycomimetics, isoxazole, isoxazoline, nitrile oxide

## Abstract

The yeast *Candida albicans* is one of the most prevalent fungal pathogens. It induces superficial and systemic infections in immunocompromised patients. The high pathogenicity of *Candida* species may be related to their adherence to the host tissues. Cell surface glycans are important receptors for *C. albicans* and warrant the development of anti-adherence ligands that can mimic them and disrupt *C. albicans*–epithelial cell interactions. A divalent galactoside glycomimetic containing 1,2,3-triazole units was found to be one of the most potent inhibitors of *C. albicans* adhesion to exfoliated buccal epithelial cells. Based on this lead molecule, herein we report on the synthesis and evaluation of a new series of mono- and divalent glycosides, featuring isophthalamide and benzene scaffolds with *C*-glycosyl isoxazoline and isoxazole, as well as *N*-glycosyl 1,2,3-triazole units. The new compounds were obtained by 1,3-dipolar cycloaddition reactions between the above scaffolds functionalized with one or two alkenyl or alkynyl moieties and glycosyl nitrile oxides and glycosyl azides, respectively. The tested deprotected compounds were non-toxic to the *C. albicans* cells and exhibited significant inhibition of *C. albicans* adhesion, showing good and moderate inhibition in exclusion and competitive assays, respectively. This study demonstrated the utility of isoxazole/isoxazoline heterocycles to replace 1,2,3-triazoles in aromatic-core glycoconjugates to furnish anti-adhesion molecules equipotent with the lead and also showed that the simple benzene core can serve well in potential anti-adhesives against *C. albicans*.

## 1 Introduction

There are approximately 200 known species in the *Candida* genus, but only a few of them are opportunistic human pathogens (Fróis-Martins et al., 2024; Macias-Paz et al., 2023; Parambath et al., 2024; Sahoo et al., 2025; Schille et al., 2025; Shrivastava and Whiteway, 2025). The most common of these is *Candida albicans*, a dimorphic yeast that inhabits the gastrointestinal and genitourinary tracts (Martin et al., 2021a; Pfaller et al., 2006). It is a normal component of the human microbiome and is mainly found in mucous membranes (Arendorf and Walker, 1980; Martin et al., 2021a; Pfaller et al., 2006; Spampinato and Leonardi, 2013). *C. albicans* becomes pathogenic in immunocompromised patients under various conditions, such as superficial and systemic infections, including those caused by catheters that are caused by catheters (Martin et al., 2021a).

The yeast cells have adhesins that allow them to adhere to host cells. *Candida* species are highly pathogenic due to their ability to adhere to and colonize the host organism (Albrecht et al., 2006; Chaffin, 2008; Martin et al., 2021a). Thus, an important research direction aims to block the pathogen’s adhesion to the host cell. This involves disrupting *C*. *albicans* adhesion using antibodies, sugar-containing biomolecules, and small-molecule inhibitors (Martin et al., 2021a). Previous studies have reported that some *C. albicans* adhesins recognize and bind to a wide range of cell surface glycans (Brassart et al., 1991; Collins-Lech et al., 1984; Critchley and Douglas, 1987; Everest-Dass et al., 2012; Johansson et al., 2000; Sandin et al., 1982). These investigations found that mono- and disaccharides (e.g., α-D-methylmannoside (Sandin et al., 1982), L-fucose (Critchley and Douglas, 1987), *N*-acetyl-D-glucosamine (Collins-Lech et al., 1984), and the Fucα(1–2)Galβ moiety (Brassart et al., 1991)) and oligosaccharides can influence the adhesion process of the yeast to human buccal epithelial cells (BECs) (Martin et al., 2021a). Glycosphingolipids are also involved in the adhesion processes and can act as receptors for these fungi. *C*. *albicans* binds specifically to lactosylceramide (for example, Galβ(1–4)Glcβ(1–1)Cer), and the terminal galactosyl group is necessary for binding (Jimenez-Lucho et al., 1990; Martin et al., 2021a).

**SCHEME 1:**
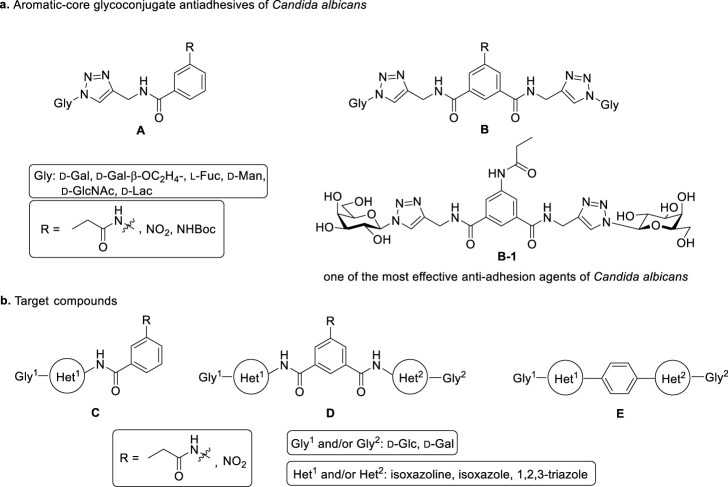
**(a)** Aromatic-core glycoconjugate antiadhesives of *Candida albicans* (Doherty et al., 2025; Martin et al., 2018; Martin et al., 2021a) and **(b)** Target compounds of this study.

Recently, several aromatic-core glycoconjugates were synthesized using copper-catalyzed azide–alkyne cycloaddition (CuAAC) reactions (Scheme 1a). The monovalent derivatives **A** did not show any significant activity, while the bivalent derivatives **B** were more promising, and among them, bis(*N*-galactopyranosyl-triazolyl) derivative **B-1** was found to be one of the most effective anti-adhesive agents of *Candida albicans* (Doherty et al., 2025; Martin et al., 2018; Martin et al., 2021a). Compound **B-1** inhibited adhesion by up to 80% when fungi were pretreated with glycoconjugates. Furthermore, this compound displaced 56% of *Candida albicans* adhering to BECs at a concentration as low as 0.1 mg/ml (Martin et al., 2018; Martin et al., 2021a). Based on these results, divalent galactopyranosyl derivatives featuring aromatic (benzene, squaramide) and bicyclic aliphatic (norbornene) scaffolds (Martin et al., 2020; Martin et al., 2021a), and tetra-, hexa-, and hexadecavalent displays of compound **B-1**, built on regioselectively addressable functionalized templates (RAFT) cyclopeptide- and polylysine-based scaffolds (Martin et al., 2021b), and glycoconjugates from azido-triethylene glycol divalent galactoside (Martin et al., 2021c), were synthesized and tested for their anti-adhesive effect on yeast. However, these compounds did not give better results than **B-1**.

Based on these preliminaries, we aimed to develop new potential anti-adhesives by replacing the triazole units in compounds **A** and **B** with isoxazoline or isoxazole rings (compounds **C** and **D**). Furthermore, compounds **E**, exhibiting benzene instead of the benzamide and isophthalamide type aromatic cores, were also envisaged to check the effect of an extreme simplification (Scheme 1b).

## 2 Materials and methods

### 2.1 Syntheses

#### 2.1.1 General methods

Optical rotations were determined with a Jasco P-2000 (Easton, MD, United States of America) polarimeter at room temperature. NMR spectra were recorded with or Bruker AM Avance I 400 MHz (400/100 MHz for ^1^H/^13^C), or Bruker AM Avance II 500 MHz (500/125 MHz for ^1^H/^13^C), a Bruker Ascend 500 MHz (500/125 MHz for ^1^H/^13^C), or a Bruker Avance Neo 700 MHz (700/175 MHz for ^1^H/^13^C) spectrometer. Chemical shifts are referenced to TMS as the internal reference (^1^H) or to the residual solvent signals (^1^H and ^13^C). The assignments of the ^1^H and ^13^C NMR signals of compounds **3**, **5**, **6**, **7**, **9**, **11**, **12**, **14**, **15**, **16**, **17**, **18**, and **19** were performed by their COSY (**3a**,**b**, **5b**,**c**, **6b**, **7a**,**b**, **9a**,**b**, **11**, **12b**, **14a**‒**d**, **15a**,**b**, **16b**,**c**, **17a**, **18b**, and **19a**,**b**,**d**), HSQC (**3a**,**b**, **5b**,**c**, **6b**, **7a**,**b**, **9a**,**b**, **11**, **12b**, **14a**‒**d**, **15a**,**b**, **16b**,**c**, **17a**, **18b**, and **19a**,**b**,**d**), and HMBC (**3a**,**b**, **5b**,**c**, **6b**, **7a**,**b**, **9a**,**b**, **11**, **12b**, **14a**‒**d**, **15a**,**b**, **16b**,**c**, **17a**, **18b**, and **19a**,**b**,**d**) spectra. Mass spectra were recorded with maXis II UHR ESI-QTOF MS (Bruker Daltonics, Bremen, Germany) instruments in positive ion mode with the electrospray ionization technique, Thermo LTQ XL (Thermo Electron Corp., San Jose, CA, United States) mass spectrometers operated in a full scan positive and negative ion ESI mode, or Agilent-LC 1200 Series coupled to a 6,210 Agilent Time-Of-Flight (TOF) in positive ion mode with electrospray ionization technique. TLC was performed on a DCAlurolle Kieselgel 60 F254 (Merck). Infrared spectra were obtained as a film on NaCl plates, as KBr disks, or via ATR as a solid on a zinc selenide crystal in the region 4,000–400 cm^−1^ on a Perkin Elmer Spectrum 100 FT-IR spectrophotometer. TLC plates were visualized under UV light, and by gentle heating (generally, no spray reagent was used, but if more intense charring was necessary, the plate was sprayed with the following solution: dry EtOH (95 mL), cc. H_2_SO_4_ (5 mL), and anisaldehyde (1 mL)). For column chromatography, Kieselgel 60 (Merck, particle size 0.063–0.200 mm) was applied.

The Supplementary Material contains the synthesis and characterization of the compounds.

### 2.2 Biological evaluation

#### 2.2.1 Fungal strain

*Candida albicans* (MEN, serotype B, clinical isolate) was maintained on Sabouraud dextrose agar. Cultures were grown to the stationary phase (1–2 × 10^8^/mL) overnight in yeast extract peptone dextrose (YEPD) broth (1% (w/v) yeast extract, 2% (w/v) bacteriological peptone, 2% (w/v) glucose) at 30 °C and 200 rpm. Stationary phase yeast cells were harvested, washed with PBS, and resuspended at a density of 1 × 10^8^/mL in PBS.

#### 2.2.2 Buccal epithelial cells

Buccal epithelial cells (BECs) were harvested from healthy volunteers by gently scraping the inside of the cheek with a sterile tongue depressor. Cells were washed in PBS and resuspended at a density of 5 × 10^5^/mL.

#### 2.2.3 Adherence assays

Yeast cells were mixed with BECs in a ratio of 50:1 in a final volume of 2 mL and incubated at 30 °C and 200 rpm for 90 min. The BEC/yeast cell mixture was harvested by passing through a polycarbonate membrane containing 30 µm pores, which trapped the BECs but allowed unattached yeast cells to pass through. This was washed × 2 with 10 mL PBS, and cells remaining on the membrane were collected and placed on glass slides that were left to air dry overnight. The cells were heat fixed and stained using 0.5% (w/v) crystal violet, rinsed using cold water to remove any surplus stain, and left to air dry for 30 min. The number of *C. albicans* cells adhering to a sample of 200 BECs per treatment was assessed microscopically. In the *exclusion assay*, the yeast cells were incubated for 90 min in the presence of each compound at the given concentration. After this time, the cells were harvested and washed twice with PBS, then resuspended in 1 mL PBS and mixed with BECs (as described). In the *competition assay*, format yeast cells, BECs, and compound (at the given concentration) were co-incubated for 90 min prior to harvesting. In the *displacement assay*, adherence was allowed to occur by mixing the yeast cells and BECs together. BECs and adherent yeast cells were harvested and re-incubated with the compound (at the given concentration) for a further 90 min, after which time the level of adherence was measured.

All experiments were performed on three independent occasions. In each assay, the number of yeast cells adhering to 200 randomly chosen BECs was determined. Results are mean ± SEM.

All glycoconjugates were dissolved in water at a given concentration (10 mg/mL), and dilutions from these stock solutions were performed as required.

#### 2.2.4 Growth inhibition assays

A 100-µL aliquot of media was added to every well in a 96-well plate with an 8-channel multi-pipette. Lanes 1 and 12 were sterile negative controls. A 100-μL aliquot of the compound at the desired concentration was added to each well in lane 3, and serial dilution of the compounds across wells 3–11 was achieved by removal of 100 µL from lane 3 and addition to lane 4 was conducted. Dilution was continued in this way until lane 11, when 100 µL from lane 11 was put to waste. *C. albicans* cells were cultured as per Section 3.2, and a sample was removed from the cell broth culture and added to 50 mLs of fresh autoclaved YEPD media. A multi-channel pipette was used to add 100 µL of cells (in media) to every well in lanes 2 to 11. The 96-well plates were then incubated overnight at 35.7 °C, and cell growth was determined using an optical density reader set at 600 nm.

## 3 Results and discussion

### 3.1 Syntheses

**SCHEME 2:**
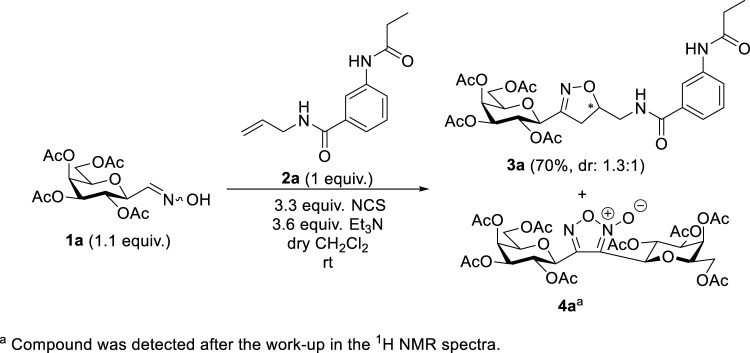
1,3-Dipolar cycloaddition reaction of *O*-peracetylated *C*-(β-D-galactopyranosyl)formaldoxime **1a** with *N*-prop-2-en-1-yl-3-propionamidobenzamide (**2a**)

3-(Galactopyranosyl)isoxazolin-5-yl benzamide **3a** was synthesized by the adaptation of a previously elaborated method (Kaszás et al., 2025), based on the 1,3-dipolar cycloaddition of *C*-galactopyranosyl nitrile oxide, generated *in situ* from *O*-peracetylated *C*-(β-D-galactopyranosyl)formaldoxime **1a** (Tóth and Somsák, 2003) (Scheme 2). Accordingly, 1 equiv. of *N*-prop-2-en-1-yl-3-propionamidobenzamide (**2a**), 1.1-fold excess of oxime **1a**, and 3.3-fold excess of *N*-chlorosuccinimide (NCS) were stirred in dry dichloromethane for 30 min at room temperature. Then, a solution of dry triethylamine (Et_3_N, 3.6 equiv.) in dry dichloromethane was added dropwise over 16 h using a syringe pump to get **3a** in the form of an inseparable mixture of diastereomers in good yield (70%). After the work-up, digalactopyranosylfuroxan **4a** (Baker et al., 2002), formed by the dimerization of nitrile oxides, was also detected in the ^1^H NMR spectra.

The same methodology was used for the formation of the divalent isoxazolines **5b** and **5c** (Table 1). First, 5-nitroisophthalamide **2b** was reacted with 2.2 equiv. of oxime **1a** in the presence of 6.6 equiv. of *N*-chlorosuccinimide and 7.26 equiv. of triethylamine to give the divalent isoxazoline **5b** in moderate yield in the form of an inseparable mixture of three diastereomers beside the monovalent derivative **3b** (entry 1). Increasing the excess of oxime **1a** to 5 equiv. significantly increased the yield of the divalent product **5b**, but the formation of **3b** could not be avoided (entry 2). A further increase in the amount of oxime **1a** did not change the outcome of the reaction significantly (entry 3). The cycloaddition was extended to 5-(propionamido)isophthalamide **2c** (entry 4). The divalent isoxazoline **5c** was formed with excellent yield as an inseparable mixture of two diastereomers. Digalactopyranosylfuroxan **4a** was isolated from all reaction mixtures.

**TABLE 1:** 1,3-Dipolar cycloaddition reactions of *O*-peracetylated *C*-(β-D-galactopyranosyl)formaldoxime 1a with 5-nitro-*N* ^*1*^,*N* ^*3*^-di(prop-2-en-1-yl)isophthalamide (2b), and *N* ^1^,*N* ^*3*^-di(prop-2-en-1-yl)-5-(propionamido)isophthalamide (2c).

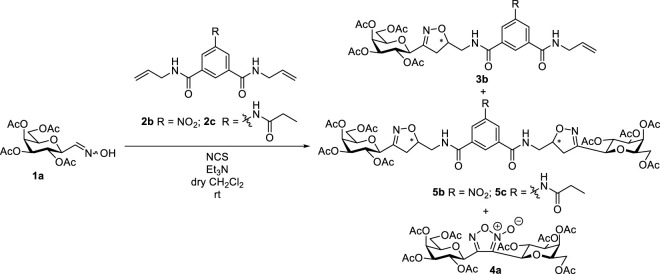									
Entry	Reaction conditions				Isolated yields (%)				
	Isophthalamide	Oxime 1a (Tóth and Somsák, 2003) (equiv.)	NCS (equiv.)	Et_3_N (equiv.)	Monovalent isoxazolines	dr 3	Divalent isoxazolines	dr 5	4a
1	**2b**	2.2	6.6	7.26	**3b** 22	1.3:1	**5b** 42	1.4:1.4:1	+^a^
2	**2b**	5	15	16.5	**3b** 22	1.3:1	**5b** 68	3.3:2.3:1	+^a^
3	**2b**	10	30	33	**3b** 14	1.3:1	**5b** 68	1:1:1	+^a^
4	**2c**	5	15	16.5	Not formed	-	**5c** 99	1.1:1	+^a^

^a^ The remaining oxime completely gave **4a** furoxan.

**SCHEME 3:**
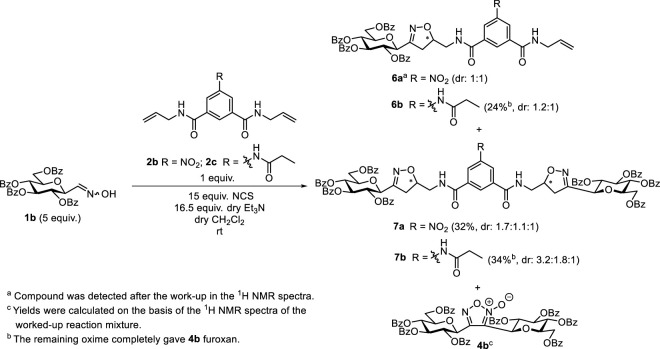
1,3-Dipolar cycloaddition reactions of *O*-perbenzoylated *C*-(β-D-glucopyranosyl)formaldoxime **1b** with *N* ^*1*^,*N* ^*3*^-di(prop-2-en-1-yl)isophthalamide **2b** and **2c**.

To form the glucopyranosyl-isoxazoline derivatives **7,** the transformation was extended to *O*-perbenzoylated *C*-(β-D-glucopyranosyl)formaldoxime **1b** (Tóth and Somsák, 2003) (Scheme 3). Cycloaddition with 5-nitroisophthalamide **2b** resulted in a complex reaction mixture from which the divalent isoxazoline **7a** was isolated in moderate yield as a mixture of two diastereomers. The monovalent product **6a** was detected in the ^1^H NMR spectra after the work-up. Better, but only moderate yields were achieved with 5-(propionamido)isophthalamide **2c** to give **6b** and **7b** as mixtures of two and three diastereomers, respectively. Glucopyranosylfuroxan **4b** (Kaszás et al., 2025) was also formed in the reactions.

To obtain isoxazole derivatives, the 1,3-dipolar cycloaddition reactions were extended to dipropargyl isophthalamides **8a** (Doherty et al., 2025) and **8b** (Martin et al., 2018) (Table 2). The application of 2.2 equiv. of oxime **1a** resulted in monovalent isoxazoles **9a** and **9b** only in low yields (entries 1 and 3). Increasing the amount of oxime **1a** to 5 equivalents increased the yield slightly in the case of the 5-nitroisophthalamide **8a** (entry 2), while a significant increase in yield was achieved with the 5-propionamidoisophthalamide **8b** (entry 4). However, the desired divalent compounds were not formed; these were even not detectable in the ^1^H NMR spectra after the work-up. The significantly lower yield obtained with the nitro derivative **8a** compared to 5-propionamidoisophthalamide **8b** correlates with previous results (see Table 1, entries 2 and 4 or Scheme 3). Our attempts to form the desired bisisoxazolyl derivatives by reacting **9a** or **9b** with oxime **1a** were also unsuccessful.

**TABLE 2:** 1,3-Dipolar cycloaddition reactions of *O*-peracetylated *C*-(β-D-galactopyranosyl)formaldoxime (1a) with *N* ^1^,*N* ^*3*^-di(prop-2-yn-1-yl)isophthalamides 8a and 8b

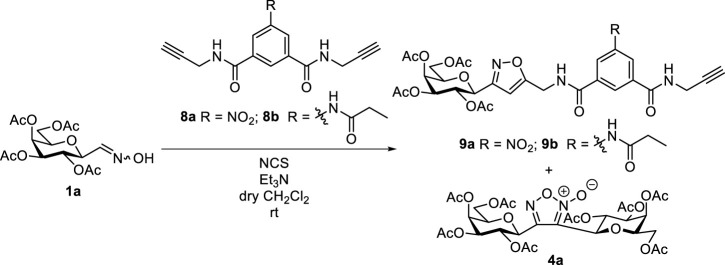						
Entry	Reaction conditions				Isolated yields (%)	
	Isophthalamide	Oxime 1a (Tóth and Somsák, 2003) (equiv.)	NCS (equiv.)	Et_3_N (equiv.)	9	4a
1	**8a** (Doherty et al., 2025)	2.2	6.6	7.26	**9a** 15	+^a^
2	**8a** (Doherty et al., 2025)	5	15	16.5	**9a** 24	+^a^
3	**8b** (Martin et al., 2018)	2.2	6.6	7.26	**9b** 19	+^a^
4	**8b** (Martin et al., 2018)	5	15	16.5	**9b** 60	+^a^

^a^ The remaining oxime completely gave **4a** furoxan.

**SCHEME 4:**
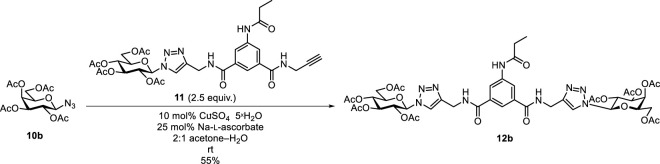
1,3-Dipolar cycloaddition reactions of 2,3,4,6-tetra-*O*-acetyl-β-D-glucopyranosyl azide (**10a**) with isophthalamide **11**.

After these failures, the preparation of a mixed diglycosyl isoxazolyl-triazolyl derivative was also attempted. However, CuAAC of 2,3,4,6-tetra-*O*-acetyl-β-D-glucopyranosyl azide **10a** (Bertho, 1930) with **9a** or **9b** in the presence of copper(II) sulfate/Na-ʟ-ascorbate system (Carvalho et al., 2010; Martin et al., 2018) or bis-triphenylphosphane-copper(I)-butyrate (Bokor et al., 2012) to form isoxazolyl-triazolyl bivalent glycoconjugates also failed. The lack of reactivity of the second ethynyl group after the formation of a glycosylisoxazole unit from the first one was previously observed in 1,3-dipolar cycloadditions of 1,4-diethynylbenzene with glycopyranosyl nitrile oxides, which only resulted in the monoglycosylisoxazolyl derivatives (Kaszás et al., 2025). In order to get the desired mixed diglycosyl isoxazolyl-triazolyl derivative, the reversed order of the heterocyclizations, i. e. formation of the triazole first and subsequent formation of the isoxazole, was also envisaged. To this end, monovalent triazole derivative **11** was prepared by the CuAAC of isophthalamide **8b** with glucopyranosyl azide **10a** (Table 3). A reaction of 1.25-fold excess of azide **10a** in the presence of 10 mol% CuSO_4_ • 5×H_2_O and 25 mol% Na-ʟ-ascorbate in DMF at reflux temperature failed (entry 1). Changing the solvent to 2:1 acetone–H_2_O and using 1 equiv. of azide **10a** resulted in the formation of **11** and the divalent **12a** (Martin, 2019) in low yields (entry 2). Under the same conditions but with 1.25 equiv. of azide **10a**, the reaction gave **11** and **12a** with higher yields in a 1:1.1 ratio (entry 3). Compound **11** was reacted with oxime **1a**, but only furoxan **4a** was formed. Compound **11** did not undergo any transformation; it was recovered in unchanged form from the reaction mixture. Subsequently, bistriazolyl **12b**, analogous to compound **B-1** but containing two different monosaccharide units (*gluco*-*galacto*) was synthesized in the reaction of **11** with glucopyranosyl azide **10a** in moderate yield (55%) (Scheme 4).

**TABLE 3:** Copper-catalyzed azide–alkyne cycloadditions of 2,3,4,6-tetra-*O*-acetyl-β-D-glucopyranosyl azide (**10a**) with *N* ^1^,*N* ^3^-di(prop-2-yn-1-yl)-5-propionamidoisophthalamide **8b**.

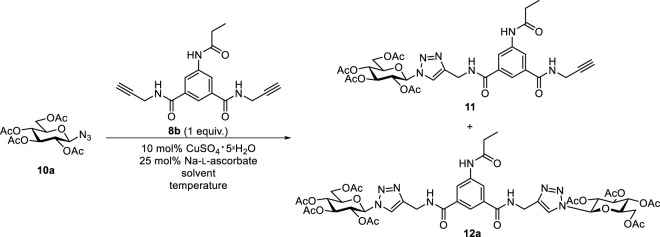					
Entry	Reaction conditions			Isolated yields (%)	
	Azide 10a (Bertho, 1930)	Solvent	T	11	12a (Martin, 2019)
1	1.25	DMF	reflux	-^a^	-^b^
2	1	2:1 acetone–H_2_O	rt	19	6
3	1.25	2:1 acetone–H_2_O	rt	30	33

^a^ Compound **11** was not detected after the work-up.

^b^ Compound **12a** was not detected after the work-up.

Next, benzene was used as a very simple scaffold to display carbohydrates in a multivalent fashion. The diglycosyl isoxazolyl-triazolyl benzenes **14a‒d** were obtained *via* CuAAC of glycopyranosyl azides **10a** and **10b** (Bertho and Maier, 1932) and glycopyranosyl isoxazolyl ethynylbenzenes **13a** (Kaszás et al., 2025) and **13b** (Kaszás et al., 2025) (Table 4). The latter were previously synthesized with cycloadditions of 1,4-diethynylbenzene and oximes **1a** or **1b** (Kaszás et al., 2025). A reaction of azide **10a** with isoxazole **13a** in the presence of copper(I) iodide (5 mol%) in dry dichloromethane at 35 °C failed (entry 1). On the other hand, the use of CuSO_4_ • 5×H_2_O/Na-L-ascorbate catalyst system in DMF at 100 °C (Carvalho et al., 2010) gave the corresponding divalent compounds **14a‒d** in moderate to good yield (entries 2–5).

**TABLE 4:** Copper-catalyzed azide–alkyne cycloaddition reactions of *O*-peracetylated β-D-glycopyranosyl azides **10a** and **10b** with 5-(4-ethynylphenyl)isoxazoles **13a** and **13b**.

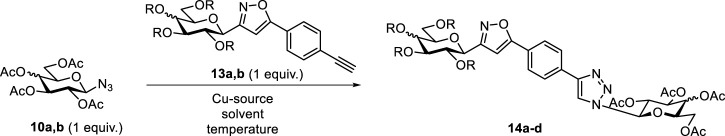										
Entry	Starting compounds					Reaction conditions				Isolated yields (%)
	10	Sugar configuration 10	13 (Kaszás et al., 2025)	Sugar configuration 13	R	Cu-source (mol%)	Na-L-ascorbate (mol%)	Solvent	T (°C)	
1	**a**(Bertho, 1930)	Glc	**a**	Gal	Ac	CuI (5)	-	dry CH_2_Cl_2_	35	**14a** ^a^
2	**a**(Bertho, 1930)	Glc	**a**	Gal	Ac	CuSO_4_ • 5×H_2_O (10)	20	DMF	100	**14a** 87
3	**b**(Bertho and Maier, 1932)	Gal	**a**	Gal	Ac	CuSO_4_ • 5×H_2_O (10)	20	DMF	100	**14b** 39
4	**a**(Bertho, 1930)	Glc	**b**	Glc	Bz	CuSO_4_ • 5×H_2_O (14)	40	DMF	100	**14c** 85
5	**b**(Bertho and Maier, 1932)	Gal	**b**	Glc	Bz	CuSO_4_ • 5×H_2_O (62)	114	DMF	100	**14d** 43

^a^ Compound **14a** was not detected after the work-up.

Finally, the protecting groups of glycopyranosyl-isoxazoline **3a**,**b**, **5b**,**c**, -isoxazole **9a**, **14a**,**b**,**d**, and -triazole **12b** derivatives were removed by the Zemplén method (Zemplén and Pacsu, 1929) using a catalytic amount of NaOMe in dry MeOH at room temperature (Table 5). The isoxazoline **15a**,**b**, **16b**,**c**, -isoxazole **17a**, **19a**,**b**,**d,** and -triazole **18a** derivatives were isolated in moderate to excellent yields.

**TABLE 5:** Deacylation of glycopyranosyl-isoxazoline **3a**,**b**, **5b**,**c**, -isoxazole **9a**, **14a**,**b**,**d**, and -triazole **12b** derivatives.

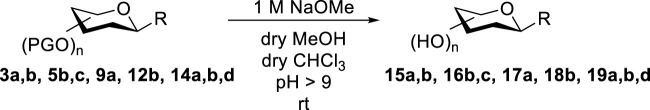				
Entry	Starting compound	Product	dr	Isolated yields (%)
1	**3a**	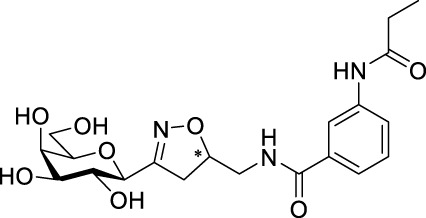 **15a**	1.3:1	81
2	**3b**	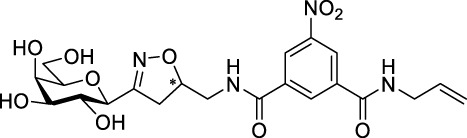 **15b**	1.3:1	51
3	**5b**	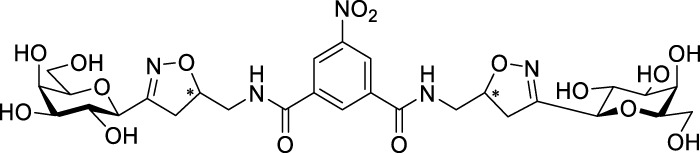 **16b**	2:1:1	60
4	**5c**	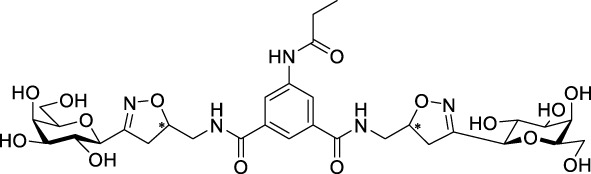 **16c**	1.3:1	93
5	**9a**	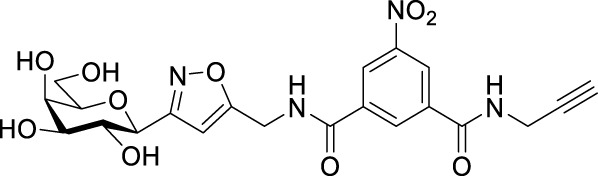 **17a**	—	51
6	**12b**	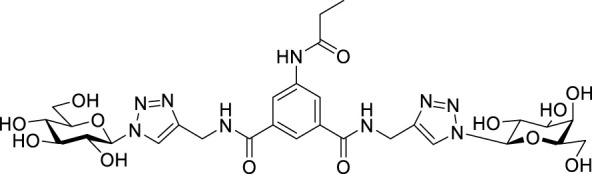 **18b**	—	74
7	**14a**	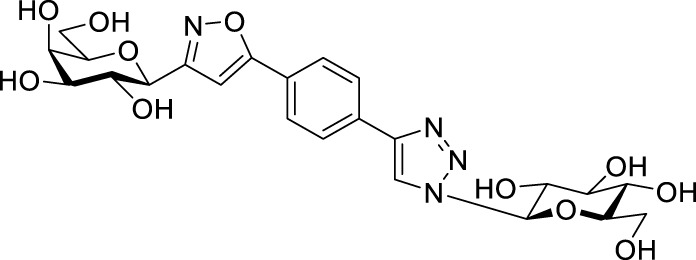 **19a**	—	58
8	**14b**	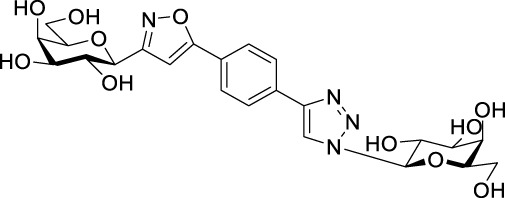 **19b**	—	34
9	**14d**	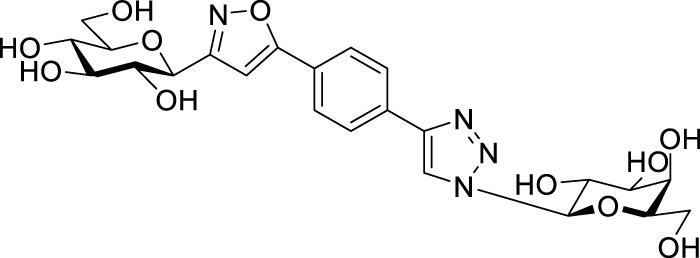 **19d**	—	24

### 3.2 Structure elucidation

The 3-(2′,3′,4′,6′-tetra-*O*-acetyl-β-D-glycopyranosyl)isoxazolines **3**, **5**, **6**, **7**, -isoxazoles **9**, 1-(2′,3′,4′,6′-tetra-*O*-acetyl-β-D-glycopyranosyl)triazole derivatives **11**, **12**, and 3-(β-D-glycopyranosyl)-5-(4-(1-(β-D-glycopyranosyl)-1*H*-1,2,3-triazol-4-yl))phenylisoxazoles **14**, and all their deacetylated derivatives **15**, **16**, **17**, **18**, and **19** were identified by their NMR and high-resolution mass spectroscopy (HRMS) spectral data. The sugar parts of the molecules gave resonances and coupling constants as expected, and these are listed among the compound characterization data in the Supplementary Material.

In the ^1^H NMR spectra of isoxazolines **3**, **5**, **6**, **7**, the H-5 proton of the isoxazoline ring appears at 4.92–4.77 ppm, while the methylene protons H-4_a_ and H-4_b_ resonate at 3.41–3.17 ppm and 3.13–2.89 ppm, respectively, with 17.0–17.5 Hz geminal coupling constants. In the ^13^C NMR spectra, carbons C-3, C-4, and C-5 of the isoxazoline ring appear at 157.0–155.9 ppm, 36.0–35.3 ppm, and 80.0–79.3 ppm, respectively. In the monovalent isoxazoline derivatives **3b** and **6b**, the vinyl CH and CH_2_ proton resonances are observed at 5.94–5.78 ppm and 4.18–3.95 ppm, respectively, with coupling constants of 17.2 Hz and 10.3 Hz between the CH and CH_2_ vinyl protons. The corresponding carbons appear at 133.5 ppm (CH-vinyl) and 43.1–43.0 ppm (CH_2_-vinyl) in the ^13^C NMR spectra. The monovalent isoxazoles **9a** and **9b** were identified by the H-4 proton signals at 6.45 ppm and 6.41 ppm in the ^1^H NMR spectra, and by the resonances of carbon atoms C-3 (168.6 ppm and 169.5 ppm), C-4 (161.4 ppm and 161.2 ppm), and C-5 (101.6 ppm and 101.2 ppm) in the ^13^C NMR spectra. The presence of the unreacted triple bond in **9a** and **9b** is indicated by the terminal acetylene H signals at 2.33 ppm and 2.29 ppm, which give cross peaks with the acetylene CH carbons at 72.8 ppm and 72.2 ppm in the HSQC spectra, respectively. In the ^1^H NMR spectra of 1,2,3-triazoles **11** and **12b**, the triazole H-5 protons are found at 7.91 ppm and 8.02–7.95 ppm and give cross peaks with the C-5 carbons at 121.3 ppm and 121.4 ppm in the HSQC spectra, respectively. The triazole C-4 carbons resonate at 145.5 ppm for the monovalent **11** and at 145.8 ppm and 145.6 ppm for the divalent **12b** compound. These chemical shifts correlate well with those found in the reported divalent triazole **12a** (Martin, 2019) (8.01 ppm (H-5), 146.0 ppm (C-4), and 121.4 ppm (C-5)). In the ^1^H NMR spectra of 3-(β-D-glycopyranosyl)-5-(4-(1-(β-D-glycopyranosyl)-1*H*-1,2,3-triazol-4-yl))phenylisoxazoles **14**, the triazole H-5″ proton appears between 8.14 ppm and 8.06 ppm, while the izoxazole H-4 proton is between 6.76 ppm and 6.72 ppm, to give cross peaks with C-5” (118.6–118.4 ppm) in the HSQC spectra. The quaternary triazole C-4″ carbon appears in the range of 147.7–147.6 ppm, while the isoxazole C-3, C-4, and C-5 carbons are between 161.6 ppm and 161.3 ppm, 98.2–98.1 ppm, and 170.2–170.1 ppm, respectively. Carbons C-4 give cross peaks with the isoxazole H-4 protons in the HSQC spectra. The spectra of deacetylated products **15**, **17**, **18**, and **19** were very similar to those listed above with respect to the characteristic proton and carbon resonances for the heterocycles.

### 3.3 Biological evaluation of the inhibition of adhesion of *Candida albicans*

The initial adherence assay was conducted by pre-treating the *C. albicans* yeast cells with each compound and, following the incubation period, introducing treated yeast to exfoliated BECs (exclusion assay). The percentage increase/decrease in adherence of yeast cells to BECs compared to the control (untreated yeast cells) is shown in Table 6. All the compounds showed moderate to high inhibition of the adherence of *C. albicans*. Compound **15a** (the only monovalent compound evaluated in this series) showed activity with 60% inhibition, which is much better than that of the known compound **A-1** (entries 1 and 2). Its divalent counterpart **16c** showed an inhibition of 77.6%, comparable to that of the nitro derivative **16b** (76.1%) and the *para*-disubstituted benzene analogs *galacto*-*gluco* **19a** (74.7%) (entries 4, 5, and 7). These values are practically the same as those of **B-1** (entry 3).

**TABLE 6:** Reduction in adherence of *C. albicans* cells to BECs following treatment with isoxazoline **15a**, **16b**,**c**, triazole **18b**, isoxazole **20a**,**b**,**d**, and furoxan **20** derivatives (% reduction estimated according to exclusion assays and competition assays, at varying concentrations of AGCs. SE in all cases was less than 10% of mean change in adherence). N.d. non-determined.

Entry	​	Compound	% Adherence reduction (exclusion assay)	% Adherence reduction (competition assay)
			0.1 mg/mL	0.05 mg/mL
1	A-1 (Martin et al., 2018)	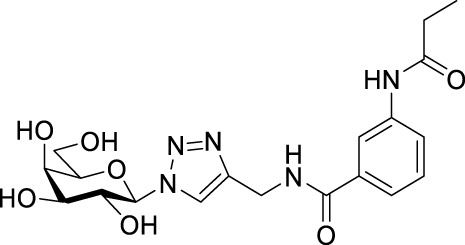	14.5	N.d
2	**15a**	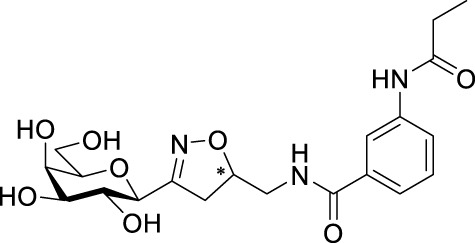	60.9 ± 0.77	48.3 ± 2.50
3	B-1 (Martin et al., 2018)	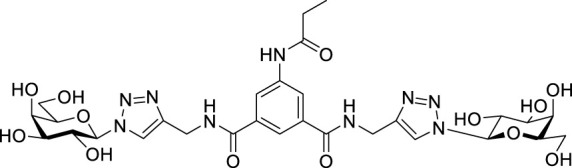	80	65.7^a^
4	**16b**	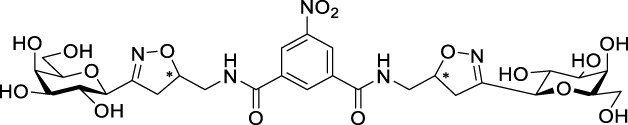	76.1 ± 0.24	64.6 ± 1.40
5	**16c**	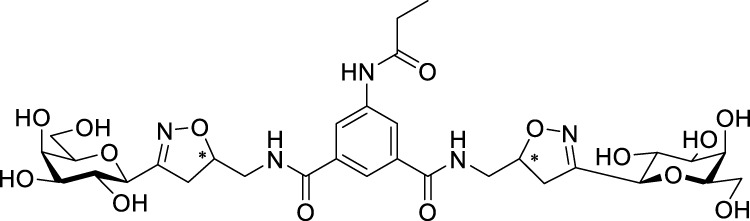	77.6 ± 0.90	50 ± 1.90
6	**18b**	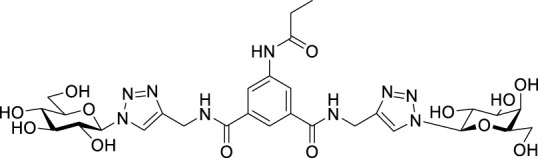	61.6 ± 0.13	N.d
7	**19a**	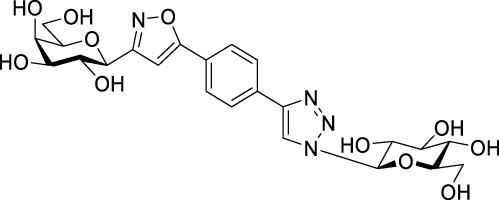	74.7 ± 0.68	41.08 ± 2.00
8	**19b**	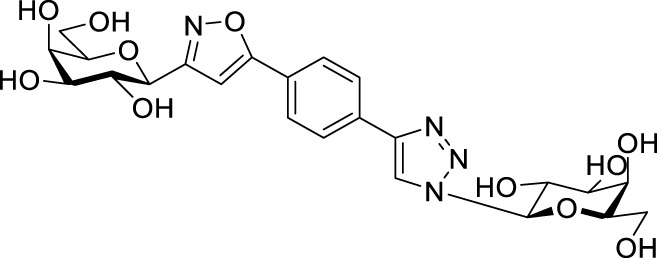	18.4 ± 0.26	N.d
9	**19d**	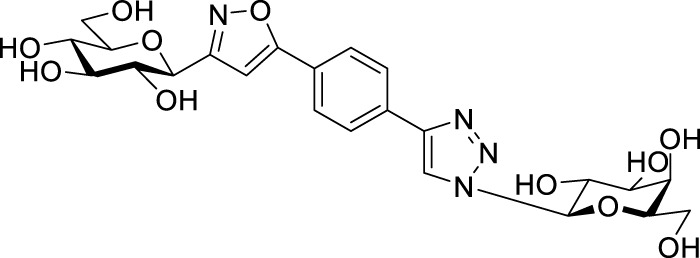	15.8 ± 0.32	N.d
10	20 (Kaszás et al., 2025)	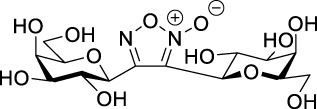	72.9 ± 0.54	64.8 ± 2.30

^a^ Concentration: 0.1 mg/mL.

**FIGURE 1:**
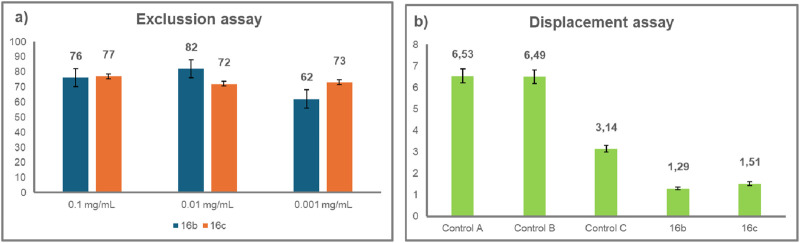
**(a)** Exclusion assay showing the percentage decrease in adhesion of *C. albicans* to BECs induced by glycoconjugates **16b** and **16c**; **(b)** Displacement assay showing the average number of yeast cells attached per BEC. Controls A, B, and C in PBS; Control C indicates the average number of yeast cells attached per BEC after the second filtration, indicating physical detachment of the yeast cells. Compounds **16b** and **16c** were tested at 0.1 mg/mL.

Meanwhile, g*alacto*-*galacto* **19b** (18.4%) and *gluco*-*galacto* **19d** (15.8%) *para*-disubstituted benzenes performed much worse in the exclusion assays (entries 8 and 9). The *gluco*-*galacto* divalent triazole **18b** showed an activity with 61.6% inhibition as an anti-adherence agent of *C*. *albicans*, which is slightly lower than that of **B-1** (80%) (entries 6 and 3). These results indicate that inhibition of adhesion is generally maintained with changes in the heterocyclic linker units (isoxazol(in)e instead of triazole) between the sugar moiety and the core aromatic scaffold, in agreement with previous structure-activity relationship studies carried out in divalent triazolyl derivatives (Kessie et al., 2025; Martin et al., 2020). In some cases, the isoxazol(in)e derivatives were slightly better than the triazoles (compare entries 1 and 2 with 7 and 9). Interestingly, compound **20**, which lacks the internal aromatic core, elicited high inhibition of adherence of the yeast to the BECs (72.9%) (entry 10). In the competition assay, compounds **16b** and **20** exhibited equipotent activity to **B-1** (entries 3, 4, and 10). In this assay, the anti-adhesion activity of compounds **15a**, **16c**, and **19a** dropped to a greater extent. The remaining compounds with low activity in the exclusion assay were not evaluated in the competition assay (entries 6, 8, 9). Two of the best performing compounds, **16b** and **16c** (with 64.6% and 50% reduction in adhesion, respectively, entries 4 and 5), maintained high adhesion inhibition activity when tested at 0.01 mg/mL and 0.001 mg/mL in exclusion assays (Figure 1a). Compounds **16b** and **16c** were further evaluated for their ability to reverse yeast adherence to BECs in the displacement assay (Figure 1b), in which each compound was added to a pre-incubated mixture of *C. albicans* and BECs.

Three controls were included: Control A and B evaluated the binding of *C. albicans* to BECs prior to compound exposure, and Control C involved BECs and adherent yeast cells being re-incubated in PBS for 90 min prior to a second filtration step. The results show that both compounds displayed high reduction in adherence (62.5% and 56.1% for **16b** and **16c**, respectively). These findings suggest that replacement of triazolyl linkers in divalent galactosides reported previously (Kessie et al., 2025; Martin et al., 2020) by isoxazolines does not compromise the anti-adhesion activity toward *C. albicans* of this type of glycoconjugate.

**FIGURE 2:**
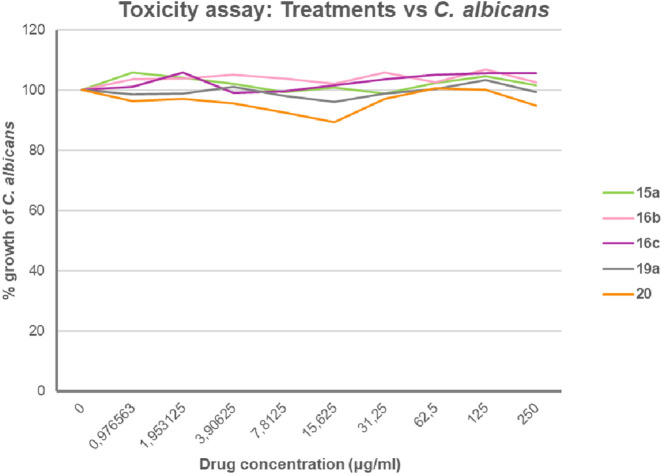
Growth of *C. albicans* under different concentrations of the glycoconjugates (µg/ml). The toxicity assay was performed as described using 96-well plates and incubated for 24 h at 37 °C.

All of the compounds evaluated were found to be water soluble and non-toxic to *C. albicans* growth (the latter was shown with the best performing compounds **15a**, **16b**,**c**, **19a**, and **20** (Kaszás et al., 2025)). This suggests that any observed reduction in adherence is not due to toxic effects (Figure 2).

## 4 Conclusion

Mono- and divalent *O*-peracylated 3-(β-D-glycopyranosyl)isoxazole and -isoxazoline derivatives were synthesized on benzamide and isophthalamide scaffolds by 1,3-dipolar cycloaddition reactions of nitrile oxides, generated *in situ* from anhydro-aldose oximes. Furthermore, *O*-peracylated mixed glycosylisoxazolyl and glycosyltriazolyl derivatives on a benzene scaffold were prepared by nitriloxide–alkyne and azide–alkyne cycloadditions. Removal of the protecting groups gave test compounds, several of which, containing D-galactopyranosyl moieties, showed good to low activity as anti-adherence agents of *C*. *albicans*. The activities of the best compounds were similar to the activity of the lead bis-galactopyranosyltriazolyl isophthalamide derivative **B-1**. These results show that the replacement of the triazole group in the original isophthalamide-derived glycomimetic by isoxazole and isoxazoline moieties does not compromise their anti-adhesion activity. In addition, the activity of a digalactosyl 1-isoxazolyl-4-triazolyl benzene was only slightly worse than that of the lead compound, demonstrating that a very simple aromatic core may be sufficient to get effective anti-adherence agents. This may pave the way toward designing simpler and thereby easier to synthesise anti-adhesives for *C. albicans*. Overall, this study highlights the feasibility of isoxazole and isoxazoline as replacements for triazoles and provides valuable information on the structural requirements for anti-adhesion activity against *C. albicans* in aromatic-core type glycomimetics.

## Data Availability

The original contributions presented in the study are included in the article/Supplementary Material; further inquiries can be directed to the corresponding author.
