# Supplementary material for: Synthesis of mono- and di-(C-glycopyranosyl-isoxazol(in)yl) derivatives on aromatic scaffolds and their evaluation as anti-adherence agents of Candida albicans
Source: Front Chem. 2026 Apr 9;14:1794047. doi: 10.3389/fchem.2026.1794047 (PMC13102757; doi:10.3389/fchem.2026.1794047)
Supplement: Supplementary file 1 [file DataSheet1.pdf]

Synthesis of mono- and di-(*C*-glycopyranosyl-isoxazol(in)yl) derivatives on aromatic scaffolds and their evaluation as anti-adherence agents of *Candida albicans*

Tímea Kaszás[1], Bence Szakács[1,2], Tekla Blága[1], Kyle Doherty[3], Rachel Keenan-Dillon[3], Shauna Reynolds[4], Kevin Kavanagh[4], Trinidad Velasco-Torrijos[3], László Somsák[1] and Marietta Tóth[1,*]

[1]Department of Organic Chemistry, University of Debrecen, Debrecen, Hungary

[2]Doctoral School of Chemistry, University of Debrecen, Debrecen, Hungary

[3]Department of Chemistry, Maynooth University, Maynooth, Ireland

[4]Department of Biology, Maynooth University, Maynooth, Ireland

*** Correspondence:**
Marietta Tóth
toth.marietta@science.unideb.hu

CONTENTS

Synthesis of *N*-prop-2-en-1-yl-3-propionamidobenzamide **2a**	S3–S4
Synthesis of 5-nitro-N^1,N^3-di(prop-2-en-1-yl)isophthalamide **2b**	S4–S5
Synthesis of N^1,N^3-di(prop-2-en-1-yl)-5-(propionamido)isophthalamide **2c**	S5–S6
General procedure I for the synthesis of 3-(2’,3’,4’,6’-tetra-*O*-acyl-β-D-glycopyranosyl)isoxazoline and -isoxazole derivatives **3**, **5**, **6**, **7** and **9**	S6–S7
Characterization of isoxazoline and isoxazole derivatives **3**, **5**, **6**, **7** and **9**	S7–S20
General procedure II for the synthesis of 1-(2’,3’,4’,6’-tetra-*O*-acetyl-β-D-glycopyranosyl)-1,2,3-triazole derivatives **11** and **12**	S21
Characterization of 1,2,3-triazole derivatives **11** and **12**	S21–S24
General procedure III for the synthesis 3-(2’,3’,4’,6’-tetra-*O*-acyl-β-D-glycopyranosyl)-5-(4-(1-(2’’’,3’’’,4’’’,6’’’-tetra-*O*-acetyl-β-D-glycopyranosyl)-1’’*H*-1’’,2’’,3’’-triazol-4-yl))phenylisoxazoles **14**	S25
Characterization of divalent triazole-isoxazole derivatives **14**	S25–S29
General procedure IV for the removal of *O*-acyl protecting groups **15**–**19**	S30
Characterization of (β-D-glycopyranosyl)isoxazoline **15**, **16**, -isoxazole **17**, **19** and -triazole **18** derivatives	S30–S39
Copies of the NMR spectra	S40–S111
Copies of the MS spectra	S112–S114
References	S115

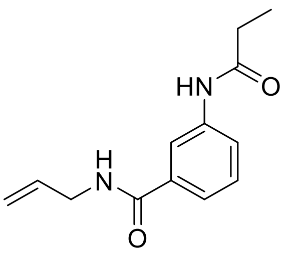

Synthesis of *N*-prop-2-en-1-yl-3-propionamidobenzamide 2a

3-Aminobenzoic acid (2.00 g, 14.58 mmol) was placed under nitrogen atmosphere and dissolved in dry tetrahydrofuran (15 mL). Propionyl chloride (2.5 equiv., 3.19 mL, 3.40 g, 36.71 mmol) was then added very slowly over the course of 30 mins and the reaction mixture was stirred for 10 mins at room temperature. Triethylamine (3 equiv., 6.10 mL, 4.43 g, 43.76 mmol) was then added over the course of 30 mins. A further 10 mL dry tetrahydrofuran was added, and the reaction mixture was stirred overnight at room temperature. The solvent was removed under reduced pressure; an extraction was carried out using ethyl acetate and 1M HCl aqueous solution. The organic phase was dried on anhydrous sodium sulfate and filtered. The solvent was removed under reduced pressure, and the residue was dissolved in hot MeOH. The insoluble material was filtered off and the filtrate was concentrated under reduced pressure. Then the crude product was recrystallised using 1:1 ethyl acetate–diethyl ether to yield the acid which was used without further purification. The acid (0.25 g, 1.29 mmol) and TBTU (1.2 equiv., 0.50 g, 1.55 mmol) were placed under nitrogen atmosphere and dissolved in *N*,*N*-dimethylformamide (5 mL). Triethylamine (1.2 equiv., 0.22 mL, 0.16 g, 1.58 mmol) was added followed by allylamine (1.2 equiv., 0.12 mL, 0.09 g, 1.60 mmol), the reaction mixture was stirred overnight at room temperature. When TLC indicated complete consumption of the acid, the solvent was removed under reduced pressure and an extraction was carried out using ethyl acetate, sat. $NaHCO_3$ and brine. The organic phase was dried on anhydrous sodium sulfate and filtered. The solvent was removed under reduced pressure to yield the pure product **2a** as a white solid (135 mg, 45%). ^{1}H NMR (500 MHz, DMSO-d_6) δ (ppm) 10.00 (1H, s, NH), 8.61

(1H, t, *J* 5.6 Hz, N*H*CH_2CHCH_2), 8.02 (1H, s, Ar), 7.77 (1H, dd, *J* 7.8, 1.6 Hz, Ar), 7.50 (1H, d, *J* 7.8 Hz, Ar), 7.36 (1H, t, *J* 7.9 Hz, Ar), 5.88 (1H, ddt, *J* 17.1, 10.4, 5.2 Hz, $NHCH_2C\mathit{H}CH_2$), 5.16 (1H, dq, *J* 17.2, 1.7 Hz, $NHCH_2CHC\mathit{H_2}$), 5.11–5.01 (1H, m, $NHCH_2CHC\mathit{H_2}$), 3.94–3.83 (2H, m, $NHC\mathit{H_2}CHCH_2$), 2.36–2.27 (2H, m, $COC\mathit{H_2}CH_3$), 1.08 (3H, t, *J* 7.6 Hz, $COCH_2C\mathit{H_3}$). ^{13}C NMR (125 MHz, DMSO-d_6) δ (ppm) 172.6 ($\mathit{C}OCH_2CH_3$), 166.5 ($\mathit{C}ONHCH_2CHCH_2$), 139.8 (Ar), 135.9 ($NHCH_2\mathit{C}HCH_2$), 135.6 (Ar), 129.0 (Ar), 122.0 (Ar), 121.7 (Ar), 118.8 (Ar), 115.5 ($NHCH_2CH\mathit{C}H_2$), 41.9 ($NH\mathit{C}H_2CHCH_2$), 29.9 ($CO\mathit{C}H_2CH_3$), 10.1 ($COCH_2\mathit{C}H_3$). IR (ATR): 3260, 1664, 1634, 1592, 1529, 1476, 1459, 1415, 1432, 1378, 1348, 1307, 1276, 1252, 1208 cm^{-1}. HR-ESI-MS positive mode (*m*/*z*): calcd. for $C_{13}H_{16}N_2O_2$ (232.12) $[M+Na]^+$=255.1109, found $[M+Na]^+$=255.1109.

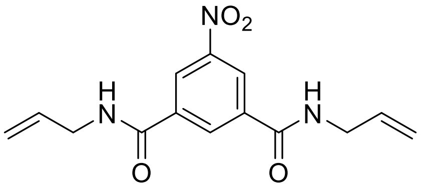

Synthesis of 5-nitro-*N*^{1},*N*^{3}-di(prop-2-en-1-yl)isophthalamide 2b

5-Nitroisopthalic acid (0.50 g, 2.37 mmol) and TBTU (2.2 equiv., 1.67 g, 5.21 mmol) were placed under nitrogen atmosphere and dissolved in *N*,*N*-dimethylformamide (10 mL). Triethylamine (2.2 equiv., 0.72 mL, 0.52 g, 5.21 mmol) was added followed by allylamine (2.2 equiv., 0.39 mL, 0.30 g, 5.21 mmol), the reaction mixture was stirred overnight at room temperature. When TLC indicated complete consumption of the starting compound, the solvent was removed under reduced pressure and and an extraction was carried out using ethyl acetate, sat. $NaHCO_3$ and brine. The organic phase was dried on anhydrous sodium sulfate and filtered. The solvent was removed under reduced pressure to yield the pure product **2b** as a pale yellow solid (415 mg, 61%). ^1H NMR (500 MHz, DMSO-d_6) δ (ppm) 9.16 (2H, t, *J* 5.5 Hz, NH), 8.82 (2H, d, *J* 1.5 Hz, Ar), 8.79 (1H, t, *J* 1.6 Hz, Ar), 5.92 (2H, ddt, *J* 17.1, 10.5, 5.3 Hz,

2×$NHCH_2C\mathit{H}CH_2$), 5.21 (2H, dq, *J* 17.2, 1.7 Hz, $CH_2CHC\mathit{H_2}$), 5.12 (2H, ddd, *J* 10.3, 3.0, 1.4 Hz, $NHCH_2CHC\mathit{H_2}$), 3.98–3.91 (4H, m, 2×$NHC\mathit{H_2}CHCH_2$). ^{13}C NMR (125 MHz, DMSO-d_6) δ (ppm) 163.9 (CO), 148.3 (Ar), 136.5 (Ar), 135.3 ($NHCH_2\mathit{C}HCH_2$), 132.8 (Ar), 124.7 (Ar), 116.1 ($NHCH_2CH\mathit{C}H_2$), 42.3 ($NH\mathit{C}H_2CHCH_2$). IR (ATR): 3346, 3287, 1637, 1582, 1536, 1442, 1420, 1341, 1325, 1276 cm^{-1}. HR-ESI-MS positive mode (*m*/*z*): calcd. for $C_{14}H_{15}N_3O_4$ (289.11) $[M+H]^+$=290.1141, found $[M+H]^+$=290.1137.

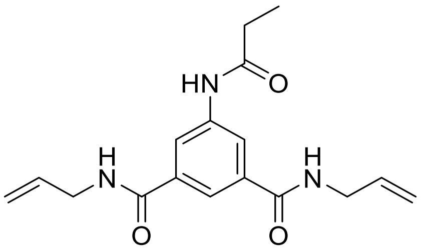

Synthesis of *N*¹,*N*³-di(prop-2-en-1-yl)-5-(propionamido)isophthalamide 2c

5-Aminoisophathalic acid (5.00 g, 27.6 mmol) was dissolved in dry tetrahydrofuran (60 mL) under nitrogen atmosphere and propionyl chloride (1.1. equiv., 2.70 mL, 2.87 g, 30.4 mmol) was added dropwise. The mixture was allowed to stir for 5 min and triethylamine (1.3 equiv., 5.00 mL, 3.63 g, 35.8 mmol) was added slowly. The reaction was left to stir for 22 h at room temperature. The solvent was removed under reduced pressure, and the residue was dissolved in hot MeOH. The insoluble material was filtered off and the filtrate was concentrated under reduced pressure to give crude diacid which was used without further purification (5.03 g, 77%). The diacid (0.50 g, 2.11 mmol) and TBTU (2.2 equiv., 1.49 g, 4.64 mmol) were placed under nitrogen atmosphere and dissolved in *N*,*N*-dimethylformamide (10 mL). Triethylamine (2.2 equiv., 0.65 mL, 0.47 g, 4.64 mmol) was added and the reaction mixture was stirred for 15 mins. Allylamine (2.2 equiv., 0.35 mL, 0.27 g, 4.64 mmol) was added and the reaction mixture was stirred overnight at room temperature. When TLC indicated complete consumption of the acid, the solvent was removed under reduced pressure and an extraction was carried out using

ethyl acetate, sat. $NaHCO_3$ and brine. The organic phase was dried on anhydrous sodium sulfate, filtered and the solvent was removed under reduced pressure. The crude product was triturated in ethyl acetate by dissolving in a minimum amount of ethyl acetate and adding in diethyl ether to encourage precipitation, this yielded the product **2c** as a pale yellow solid (400 mg, 60%). ^{1}H NMR (500 MHz, DMSO-d_6) δ (ppm) 10.14 (1H, s, NH), 8.66 (2H, t, *J* 5.7 Hz, NH), 8.18 (2H, d, *J* 1.4 Hz, Ar), 7.94 (1H, t, *J* 1.4 Hz, Ar), 5.90 (2H, ddt, *J* 17.1, 10.4, 5.3 Hz, 2×$CH_2C\mathit{H}CH_2$), 5.24–5.14 (2H, m, $CH_2CHC\mathit{H_2}$), 5.11 (2H, ddd, *J* 10.3, 4.4, 1.6 Hz, $CH_2CHC\mathit{H_2}$), 3.93–3.85 (4H, m, 2×$C\mathit{H_2}CHCH_2$), 2.39–2.26 (2H, m, $COC\mathit{H_2}CH_3$), 1.09 (3H, t, *J* 7.6 Hz, $COCH_2C\mathit{H_3}$). ^{13}C NMR (125 MHz, DMSO-d_6) δ (ppm) 172.7 ($\mathit{C}OCH_2CH_3$), 166.3 (CO), 139.9 (Ar), 135.8 (Ar), 135.7 ($CH_2\mathit{C}HCH_2$), 121.1 (Ar), 120.5 (Ar), 115.7 ($CH_2CH\mathit{C}H_2$), 42.0 (CH_2), 29.9 ($\mathit{C}H_2CH_3$), 10.0 ($CH_2\mathit{C}H_3$). IR (ATR): 1636, 1605, 1567, 1530, 1444, 1417, 1331, 1310, 1267, 1208 cm^{-1}. HR-ESI-MS positive mode (*m*/*z*): calcd. for $C_{17}H_{21}N_3O_3$ (315.16) $[M+H]^+$=316.1661, found $[M+H]^+$=316.1657.

General procedure I for the synthesis of 3-(2’,3’,4’,6’-tetra-*O*-acyl-β-D-glycopyranosyl)isoxazoline and -isoxazole derivatives 3, 5, 6, 7 and 9

A *C*-(2,3,4,6-tetra-*O*-acyl-β-D-glycopyranosyl)formaldehyde oxime (**1a** and **1b**,[1] 1.1 or 5 mmol (indicated for the particular compounds)), *N*-chlorosuccinimide (3.3 or 15 mmol) and dipolarophile (alkene or alkyne (1 mmol)) were added to dry dichloromethane (19 or 85 mL). The suspension was stirred for 30 minutes at room temperature, and then a solution of triethylamine (3.6 or 16.5 mmol) in dichloromethane (43 or 200 mL) was added dropwise with a syringe pump in 16 hours. When TLC (2:1 EtOAc–hexane or 2:1 EtOAc–heptane for **1a**, 1:1 EtOAc–hexane for **1b**) indicated complete consumption of the starting compound (~16 h), the solvent was removed under reduced pressure, and the residue was purified by silica gel column

chromatography with eluents indicated for the particular compounds to give 3-(2',3',4',6'-tetra-*O*-acyl-β-D-glycopyranosyl)isoxazoline **3**, **5**, **6**, **7** and -isoxazole **9** derivatives.

Characterization of isoxazoline and isoxazole derivatives 3, 5, 6, 7 and 9

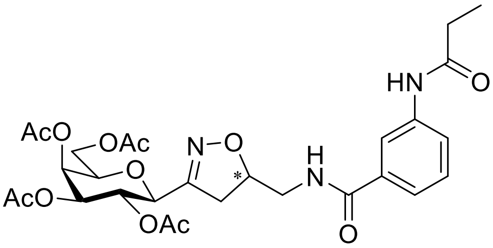

***N*-((3-(2',3',4',6'-Tetra-*O*-acetyl-β-D-galactopyranosyl)isoxazolin-5-yl)methyl)-3-(propionamido)benzamides (3a)**

Prepared from oxime **1a** (1.1 equiv., 0.05 g, 0.13 mmol) and *N*-(prop-2-en-1-yl)-3-(propionamido)benzamide **2a** (0.03 g, 0.12 mmol) according to the General procedure I. Purified by column chromatography (2:1 EtOAc–hexane) to yield 51 mg (70%) of **3a** (diastereomeric ratio: 1.3:1) as a white amorphous product. R_f: 0.18 (2:1 EtOAc–hexane). **3a-I** ^{1}H NMR (400 MHz, $CDCl_3$) δ (ppm) 7.99–7.74 (3H, m, N*H*COCH_2CH_3, Ar), 7.54 (1H, d, *J* 7.7 Hz, Ar), 7.42–7.31 (1H, m, Ar), 6.87 (1H, pseudo t, $J_{NH,CHa}$ 5.8, $J_{NH,CHb}$ 5.9 Hz, CH_2N*H*CO), 5.47 (1H, dd, $J_{4',5'}$ 0.7 Hz, H-4'), 5.18 (1H, pseudo t, $J_{2',3'}$ 10.1 Hz, H-2'), 5.14 (1H, dd, $J_{3',4'}$ 3.1 Hz, H-3'), 4.93–4.79 (1H, m, H-5), 4.33 (1H, d, $J_{1',2'}$ 9.2 Hz, H-1'), 4.15–4.04 (2H, m, H-6_a', H-6_b'), 4.03–3.87 (1H, m, H-5'), 3.79–3.52 (2H, m, C*H*$_2$NHCO), 3.18 (1H, dd, $J_{4a,4b}$ 17.5, $J_{4a,5}$ 11.0, Hz, H-4_a), 2.99 (1H, dd, $J_{4b,5}$ 8.4 Hz, H-4_b), 2.41 (2H, q, *J* 7.6 Hz, NHCOC*H*$_2$CH_3), 2.14, 2.05, 2.00 (12H, 4s, 4×CH_3), 1.24 (3H, t, *J* 7.5 Hz, NHCOCH_2C*H*$_3$). ^{13}C NMR (100 MHz, $CDCl_3$) δ (ppm) 172.6 (NH*C*OCH_2CH_3), 170.6, 170.3, 170.1 (4×CO), 167.7 (CH_2NH*C*O), 156.5 (C-3), 138.8–114.4 (Ar), 79.5 (C-5), 74.6 (C-5'), 74.3 (C-1'), 71.2 (C-3'), 67.4 (C-4'), 66.5 (C-2'), 61.7 (C-6'), 42.6 (*C*H_2NHCO), 36.0 (C-4), 30.7 (NHCO*C*H_2CH_3), 20.8, 20.7 (4×CH_3), 9.7 (NHCOCH_2*C*H_3). **3a-II** ^{1}H NMR (400 MHz, $CDCl_3$) δ (ppm) 7.99–7.74 (3H, m,

N*H*COCH$_2$CH$_3$, Ar), 7.45 (1H, d, *J* 7.8 Hz, Ar), 7.42–7.31 (1H, m, Ar), 6.77 (1H, pseudo t, $J_{NH,CHa}$ 5.9, $J_{NH,CHb}$ 6.2 Hz, CH$_2$N*H*CO), 5.45 (1H, dd, $J_{4',5'}$ 0.7 Hz, H-4'), 5.19 (1H, pseudo t, $J_{2',3'}$ 10.1 Hz, H-2'), 5.12 (1H, dd, $J_{3',4'}$ 3.3 Hz, H-3'), 4.93–4.79 (1H, m, H-5), 4.33 (1H, d, $J_{1',2'}$ 9.2 Hz, H-1'), 4.03–3.87 (3H, m, H-5, H-6$_a$', H-6$_b$'), 3.79–3.52 (2H, m, C*H*$_2$NHCO), 3.27 (1H, dd, $J_{4a,4b}$ 17.4, $J_{4a,5}$ 10.7 Hz, H-4$_a$), 2.92 (1H, dd, $J_{4b,5}$ 7.5 Hz, H-4$_b$), 2.41 (2H, q, *J* 7.6 Hz, NHCOC*H*$_2$CH$_3$), 2.17, 2.02, 2.00 (12H, 4s, 4×CH$_3$), 1.24 (3H, t, *J* 7.5 Hz, NHCOCH$_2$C*H*$_3$). ^{13}C NMR (100 MHz, CDCl$_3$) δ (ppm) 172.7 (NH*C*OCH$_2$CH$_3$), 170.7, 170.3, 170.1 (4×CO), 167.9 (CH$_2$NH*C*O), 156.3 (C-3), 138.8–114.4 (Ar), 79.9 (C-5), 74.3 (C-1'), 74.2 (C-5'), 71.2 (C-3'), 67.3 (C-4'), 66.3 (C-2'), 61.2 (C-6'), 42.9 (*C*H$_2$NHCO), 35.8 (C-4), 30.7 (NHCO*C*H$_2$CH$_3$), 20.8, 20.7 (4×CH$_3$), 9.7 (NHCOCH$_2$*C*H$_3$). HR-ESI-MS positive mode (*m*/*z*): calcd. for $C_{28}H_{35}N_3O_{12}$ (605.22) [M+H]$^+$=606.2294, found: [M+H]$^+$=606.2294

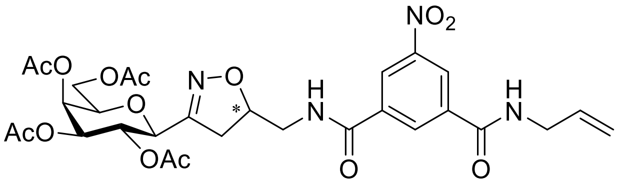

N^1-((3-(2',3',4',6'-Tetra-*O*-acetyl-β-D-galactopyranosyl)isoxazolin-5-yl)methyl)-N^3-(prop-2-en-1-yl)-5-nitroisophthalamides (3b)

Prepared from oxime **1a** (5 equiv., 0.10 g, 0.27 mmol) and 5-nitro-N^1,N^3-di(prop-2-en-1-yl)isophthalamide **2b** (0.02 g, 0.05 mmol) according to the General procedure I. Purified by column chromatography (from 1:1 to 4:1 EtOAc–heptane) to yield 8 mg (22%) of **3b** (diastereomeric ratio: 1.3:1) as a yellow amorphous product. R$_f$: 0.50 (2:1 EtOAc–hexane). **3b-I** ^{1}H NMR (500 MHz, CDCl$_3$) δ (ppm) 8.90 (1H, t, *J* 1.5 Hz, Ar), 8.81 (1H, t, *J* 1.6 Hz, Ar), 8.66 (1H, t *J* 1.4 Hz, Ar), 7.10 (1H, pseudo t, $J_{CHa,NH}$ 5.6, $J_{CHb,NH}$ 5.3 Hz, CH$_2$N*H*CO), 6.73 (1H, pseudo t, $J_{NH,CHc}$ 5.8, $J_{NH,CHd}$ 5.4 Hz, CON*H*CH$_2$CHCH$_2$), 5.94 (1H, dddd, $J_{CH,CHa}$ 17.2, $J_{CH,CHb}$ 10.3, $J_{CHc,CH}$ 6.9, $J_{CHd,CH}$ 4.8 Hz, CONHCH$_2$C*H*CH$_2$), 5.48 (1H, dd, $J_{3',4'}$ 3.0, $J_{4',5'}$ 0.7 Hz, H-

4'), 5.34–5.26 (1H, m, CONHC*H*$_{2c}$CHCH$_2$), 5.26–5.21 (1H, m, CONHC*H*$_{2d}$CHCH$_2$), 5.21–5.11 (1H, m, H-3'), 5.05 (1H, pseudo t, $J_{2',3'}$ 10.2 Hz, H-2'), 4.94–4.81 (1H, m, H-5), 4.32 (1H, d, $J_{1',2'}$ 9.7 Hz, H-1'), 4.18–4.04 (4H, m, H-6$_a$', H-6$_b$', CONHCH$_2$CHC*H*$_2$), 4.00 (1H, ddd, $J_{4',5'}$ 0.5, $J_{5',6a'}$ 6.6, $J_{5',6b'}$ 6.1 Hz, H-5'), 3.96–3.64 (2H, m, C*H*$_2$NHCO), 3.19 (1H, dd, $J_{4a,4b}$ 17.4, $J_{4a,5}$ 10.8 Hz, H-4$_a$), 3.05 (1H, dd, $J_{4b,5}$ 9.9 Hz, H-4$_b$), 2.15, 2.06, 2.02, 2.00 (12H, 4s, 4×CH$_3$). ^{13}C NMR (90 MHz, CDCl$_3$) δ (ppm) 171.5, 170.6, 170.2, 170.0 (4×CO), 165.1 (CH$_2$NH*C*O), 164.3 (*C*ONHCH$_2$CHCH$_2$), 157.0 (C-3), 148.7–124.7 (Ar), 133.5 (CONHCH$_2$*C*HCH$_2$), 117.6 (CONH*C*H$_2$CHCH$_2$), 79.3 (C-5), 74.7 (C-5'), 74.5 (C-1'), 71.0 (C-3'), 67.4 (C-4'), 66.7 (C-2'), 61.7 (C-6'), 43.0 (CONHCH$_2$CH*C*H$_2$), 42.1 (*C*H$_2$NHCO), 35.4 (C-4), 21.0, 20.8, 20.7 (4×CH$_3$).

3b-II ^{1}H NMR (500 MHz, CDCl$_3$) δ (ppm) 8.76 (1H, t, *J* 1.5 Hz, Ar), 8.75 (1H, t, *J* 1.5 Hz, Ar), 8.58 (1H, t, *J* 1.5 Hz, Ar), 7.04 (1H, pseudo t, $J_{CHa,NH}$ 5.9, $J_{CHb,NH}$ 5.9 Hz, CH$_2$N*H*CO), 6.79 (1H, pseudo t, $J_{NH,CHc}$ 5.6, $J_{NH,CHd}$ 5.6 Hz, CON*H*CH$_2$CHCH$_2$), 5.94 (1H, dddd, $J_{CH,CHa}$ 17.2, $J_{CH,CHb}$ 10.3, $J_{CHc,CH}$ 6.9, $J_{CHd,CH}$ 4.8 Hz, CONHCH$_2$C*H*CH$_2$), 5.46 (1H, dd, $J_{3',4'}$ 2.9, $J_{4',5'}$ 0.7 Hz, H-4'), 5.34–5.26 (1H, m, CONHC*H*$_{2c}$CHCH$_2$), 5.26–5.21 (1H, m, CONHC*H*$_{2d}$CHCH$_2$), 5.21–5.11 (2H, m, H-2', H-3'), 4.94–4.81 (1H, m, H-5), 4.36 (1H, d, $J_{1',2'}$ 9.3 Hz, H-1'), 4.18–4.04 (2H, m, NHCOCH$_2$CHC*H*$_2$), 3.96–3.64 (5H, m, H-5', H-6$_a$', H-6$_b$', C*H*$_2$NHCO), 3.31 (1H, dd, $J_{4a,4b}$ 17.3, $J_{4a,5}$ 10.6 Hz, H-4$_a$), 2.90 (1H, dd, $J_{4b,5}$ 7.2 Hz, H-4$_b$), 2.16, 2.05, 2.02, 1.99 (12H, 4s, 4×CH$_3$). ^{13}C NMR (90 MHz, CDCl$_3$) δ (ppm) 170.4, 170.2, 170.1, 170.0 (4×CO), 165.0 (CH$_2$NH*C*O), 164.4 (*C*ONHCH$_2$CHCH$_2$), 156.7 (C-3), 148.7–124.7 (Ar), 133.5 (CONHCH$_2$*C*HCH$_2$), 117.7 (CONH*C*H$_2$CHCH$_2$), 79.6 (C-5), 74.5 (C-5'), 74.2 (C-1'), 71.2 (C-3'), 67.3 (C-4'), 66.3 (C-2'), 61.2 (C-6'), 43.4 (*C*H$_2$NHCO), 43.0 (CONHCH$_2$CH*C*H$_2$), 35.9 (C-4), 20.8, 20.7 (4×CH$_3$). HR-ESI-MS positive mode (*m*/*z*): calc. for $C_{29}H_{34}N_4O_{14}$ (662.21) $[M+Na]^+$=685.1964, found: $[M+Na]^+$=685.1964.

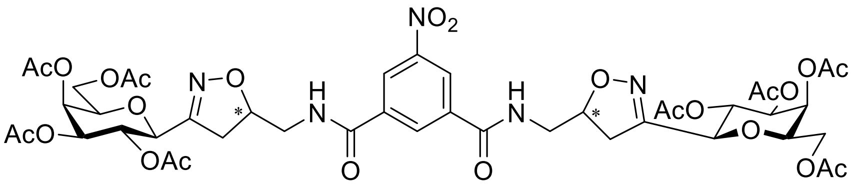

***N*^{1},*N*^{3}-Di-((3-(2’,3’,4’,6’-tetra-*O*-acetyl-β-D-galactopyranosyl)isoxazolin-5-yl)methyl)-5-nitroisophthalamides (5b)**

Prepared in the previous reaction mixture, from oxime **1a** (5 equiv., 0.10 g, 0.27 mmol) and 5-nitro-*N*^{1},*N*^{3}-di(prop-2-en-1-yl)isophthalamide **2b** (0.02 g, 0.05 mmol) according to the General procedure I. Purified by column chromatography (from 1:1 to 4:1 EtOAc–hexane) to yield 38 mg (68%) of **5b** (diastereomeric ratio: 3.3:2.3:1) as a yellow amorphous product. R_f: 0.29 (4:1 EtOAc–hexane). **5b-I** ^{1}H NMR (500 MHz, $CDCl_3$) δ (ppm) 9.03–8.39 (3H, m, Ar), 7.18–6.83 (2H, m, 2×NH), 5.48 (2H, dd, $J_{3',4'}$ 3.2, $J_{4',5'}$ 0.7 Hz, 2×H-4’), 5.23–5.12 (2H, m, 2×H-3’), 5.06 (2H, pseudo t, $J_{2',3'}$ 10.2 Hz, 2×H-2’), 4.96–4.81 (2H, m, 2×H-5), 4.32 (2H, d, $J_{1',2'}$ 9.6 Hz, 2×H-1’), 4.19–3.54 (10H, m, 2×H-5’, 2×H-6$_a$’, 2×H-6$_b$’, 2×CH_2), 3.20 (2H, dd, $J_{4a,4b}$ 17.5, $J_{4a,5}$ 11.0 Hz, 2×H-4$_a$), 3.04 (2H, dd, $J_{4b,5}$ 9.5 Hz, 2×H-4$_b$), 2.15, 2.06, 2.00 (24H, 8s, 8×CH_3). ^{13}C NMR (90 MHz, $CDCl_3$) δ (ppm) 170.5, 170.2, 170.0 (8×CO), 165.1 (2×*C*ONHCH_2), 156.7 (2×C-3), 148.6–124.9 (Ar), 79.4 (2×C-5), 74.6 (2×C-5’), 74.4 (2×C-1’), 71.0 (2×C-3’), 67.4 (2×C-4’), 66.6 (2×C-2’), 61.7 (2×C-6’), 42.4 (2×*C*H_2NHCO), 35.6 (2×C-4), 20.8, 20.7, 20.6 (8×CH_3). **5b-II** ^{1}H NMR (500 MHz, $CDCl_3$) δ (ppm) 9.03–8.39 (3H, m, Ar), 7.18–6.83 (2H, m, 2×NH), 5.46 (2H, dd, $J_{3',4'}$ 2.9, $J_{4',5'}$ 0.7 Hz, 2×H-4’), 5.23–5.12 (4H, m, 2×H-2’, 2×H-3’), 4.96–4.81 (2H, m, 2×H-5), 4.37 (2H, d, $J_{1',2'}$ 9.4 Hz, 2×H-1’), 4.19–3.54 (10H, m, 2×H-5’, 2×H-6$_a$’, 2×H-6$_b$’, 2×CH_2), 3.32 (2H, dd, $J_{4a,4b}$ 17.4, $J_{4a,5}$ 10.4 Hz, 2×H-4$_a$), 2.89 (2H, dd, $J_{4b,5}$ 7.6 Hz, 2×H-4$_b$), 2.17, 2.04, 2.03 (24H, 8s, 8×CH_3). ^{13}C NMR (90 MHz, $CDCl_3$) δ (ppm) 171.1, 170.3, 170.0 (8×CO), 165.1 (2×*C*ONHCH_2), 156.7 (2×C-3), 148.6–124.9 (Ar), 79.6 (2×C-5), 74.5 (2×C-5’), 74.1 (2×C-1’), 71.1 (2×C-3’), 67.3 (2×C-4’), 66.3 (2×C-2’), 61.2 (2×C-6’), 43.4 (2×*C*H_2NHCO), 36.0 (2×C-4), 20.8, 20.7, 20.6 (8×CH_3). **5b-III** ^{1}H NMR (500 MHz, $CDCl_3$) δ

(ppm) 9.03–8.39 (3H, m, Ar), 7.18–6.83 (2H, m, 2×NH), 5.50–5.42 (2H, m, 2×H-4'), 5.23–5.12 (4H, m, 2×H-2', 2×H-3'), 4.96–4.81 (2H, m, 2×H-5), 4.39 (2H, d, $J_{1',2'}$ 10.1 Hz, 2×H-1'), 4.19–3.54 (10H, m, 2×H-5', 2×H-6$_a$', 2×H-6$_b$', 2×CH_2), 3.20 (2H, dd, $J_{4a,5}$ 11.0 Hz, 2×H-4$_a$), 3.04 (2H, dd, $J_{4a,4b}$ 17.3, $J_{4b,5}$ 9.4 Hz, 2×H-4$_b$), 2.03, 2.00 (24H, 8s, 8×CH_3). ^{13}C NMR (90 MHz, $CDCl_3$) δ (ppm) 170.5, 170.3, 170.1 (8×CO), 165.2 (2×*C*ONHCH$_2$), 156.8 (2×C-3), 148.6–124.9 (Ar), 79.4 (2×C-5), 74.4 (2×C-5'), 74.1 (2×C-1'), 71.4 (2×C-3'), 67.4 (2×C-4'), 66.3 (2×C-2'), 61.7 (2×C-6'), 42.4 (2×*C*H$_2$NHCO), 35.6 (2×C-4), 20.8, 20.7, 20.6 (8×CH_3). HR-ESI-MS positive mode (*m*/*z*): calc. for $C_{44}H_{53}N_5O_{24}$ (1035.31) $[M+Na]^+$=1058.2973, found: $[M+Na]^+$=1058.2972.

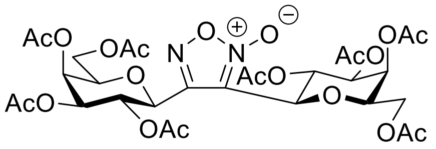

3,4-Di(2,3,4,6-tetra-*O*-acetyl-β-D-galactopyranosyl)-1,2,5-oxadiazole-2-oxide (4a)

Prepared in the previous reaction mixture, from oxime **1a** (5 equiv., 0.10 g, 0.27 mmol) and 5-nitro-N^1,N^3-di(prop-2-en-1-yl)isophthalamide **2b** (0.02 g, 0.05 mmol) according to the General procedure I. Purified by column chromatography (from 1:1 to 4:1 EtOAc–hexane) to yield 83 mg (82%, The yield was calculated form the amount of oxime **1a**.) of **4a** as a yellow amorphous product. R_f: 0.47 (2:1 EtOAc–hexane). ^{1}H NMR (500 MHz, $CDCl_3$) δ (ppm) 5.65 (1H, pseudo t, $J_{2',3'}$ 10.0 Hz, H-2'), 5.58 (1H, pseudo t, $J_{2'',3''}$ 10.0 Hz, H-2''), 5.55 (1H, dd, $J_{4',5'}$ 0.7 Hz, H-4'), 5.51 (1H, dd, $J_{4'',5''}$ 0.7 Hz, H-4''), 5.19 (1H, dd, $J_{3',4'}$ 3.3 Hz, H-3'), 5.17 (1H, dd, $J_{3'',4''}$ 3.3 Hz, H-3''), 4.84 (1H, d, $J_{1',2'}$ 10.1 Hz, H-1'), 4.82 (1H, d, $J_{1'',2''}$ 10.1 Hz, H-1''), 4.31–4.01 (6H, m, H-5', H-5'', H-6$_a$', H-6$_a$'', H-6$_b$', H-6$_b$''), 2.24, 2.06, 2.05, 2.02, 2.00, 1.99 (24H, 8s, 8×CH_3). ^{13}C NMR (125 MHz, $CDCl_3$) δ (ppm) 170.4, 170.2, 170.1, 170.0, 169.5, 169.2 (8×CO), 153.3 (C-4), 111.7 (C-3), 75.4 (C-5''), 75.2 (C-5'), 72.9 (C-1''), 71.7 (C-3', C-3''), 70.8 (C-1'), 67.3

(C-4’, C-4”), 66.9 (C-2”), 65.9 (C-2’), 61.4 (C-6’, C-6”), 20.7, 20.6, 20.5 (4×CH_3). NMR spectra are identical with those reported.[2]

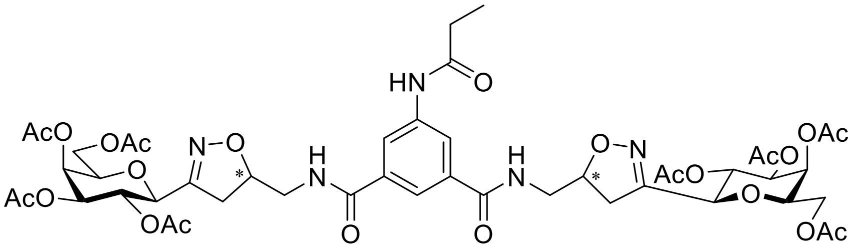

***N*^1^,*N*^3^-Di-((3-(2’,3’,4’,6’-tetra-*O*-acetyl-β-D-galactopyranosyl)isoxazolin-5-yl)methyl)-5-(propionamido)isophthalamides (5c)**

Prepared from oxime **1a** (5 equiv., 0.50 g, 1.33 mmol) and N^1,N^3-di(prop-2-en-1-yl)-5-(propionamido)isophthalamide **2c** (0.08 g, 0.27 mmol) according to the General procedure I. Purified by column chromatography (from 2:1 to 6:1 EtOAc–hexane) to yield 280 mg (99%) of **5c** (diastereomeric ratio: 1.1:1) as a yellow amorphous product. R_f: 0.39 (5:1 EtOAc–toluene). **5c-I** ^{1}H NMR (400 MHz, $CDCl_3$) δ (ppm) 8.29–7.78 (4H, m, Ar, N*H*$COCH_2CH_3$), 7.13–6.92 (2H, m, 2×NH), 5.47 (2H, dd, $J_{3',4'}$ 2.7, $J_{4',5'}$ 1.0 Hz, 2×H-4’), 5.23–5.11 (4H, m, 2×H-2’, 2×H-3’) 4.96–4.79 (2H, m, 2×H-5), 4.42–4.30 (2H, m, 2×H-1’), 4.17–4.05 (4H, m, 2×H-6$_a$’, 2×H-6$_b$’), 4.04–3.86 (2H, m, 2×H-5’), 3.81–3.50 (4H, m, 2×CH_2), 3.20 (2H, dd, $J_{4a,4b}$ 17.5, $J_{4a,5}$ 11.0 Hz, 2×H-4$_a$), 2.98 (2H, dd, $J_{4b,5}$ 8.5 Hz, 2×H-4$_b$), 2.44 (2H, q, *J* 7.5 Hz, $NHCOCH_2CH_3$ with *H*$_2$), 2.17, 2.06, 2.02, 2.00 (24H, 8s, 8×CH_3), 1.24 (3H, t, *J* 7.4 Hz, $NHCOCH_2CH_3$). ^{13}C NMR (100 MHz, $CDCl_3$) δ (ppm) 173.0 (NH*C*OCH_2CH_3), 170.8, 170.6, 170.3, 170.1 (8×CO), 166.9 (2×*C*ONHCH_2), 156.2, 156.1 (2×C-3), 139.3–121.0 (Ar), 79.4 (2×C-5), 74.7 (2×C-5’), 74.2 (2×C-1’), 71.2 (2×C-3’), 67.4, 67.3 (2×C-4’), 66.5 (2×C-2’), 61.7 (2×C-6’), 42.9, 42.8 (2×*C*H_2NHCO), 36.0, 35.9 (2×C-4), 30.6 (NHCO*C*H_2CH_3), 20.8, 20.7 (8×CH_3), 9.6 (NHCOCH_2*C*H_3). **5c-II** ^{1}H NMR (400 MHz, $CDCl_3$) δ (ppm) 8.29–7.78 (4H, m, Ar, N*H*COCH_2CH_3), 7.13–6.92 (2H, m, 2×NH), 5.46 (2H, dd, $J_{3',4'}$ 2.1, $J_{4',5'}$ 1.0 Hz, 2×H-4’),

5.23–5.11 (4H, m, 2×H-2’, 2×H-3’) 4.96–4.79 (2H, m, 2×H-5), 4.42–4.30 (2H, m, 2×H-1’), 4.04–3.86 (6H, m, 2×H-5’, 2×H-6_a’, 2×H-6_b’), 3.81–3.50 (4H, m, 2×CH_2), 3.28 (2H, dd, $J_{4a,4b}$ 17.4, $J_{4a,5}$ 10.9 Hz, 2×H-4_a), 2.91 (2H, dd, $J_{4b,5}$ 7.4 Hz, 2×H-4_b), 2.44 (2H, q, *J* 7.5 Hz, NHCOCH_2CH$_3$), 2.18, 2.16, 2.01, 2.00 (24H, 8s, 8×CH_3), 1.24 (3H, t, *J* 7.4 Hz, NHCOCH$_2$CH_3). ^{13}C NMR (100 MHz, $CDCl_3$) δ (ppm) 173.0 (NH*C*OCH$_2$CH$_3$), 170.7, 170.4, 170.3, 170.1 (8×CO), 167.2 (2×*C*ONHCH$_2$), 156.5 (2×C-3), 139.3–121.0 (Ar), 79.7 (2×C-5), 74.4 (2×C-5’), 74.3 (2×C-1’), 71.2 (2×C-3’), 67.4 (2×C-4’), 66.3 (2×C-2’), 61.3, 61.2 (2×C-6’), 43.2 (2×*C*H$_2$NHCO), 36.0 (2×C-4), 30.6 (NHCO*C*H$_2$CH$_3$), 20.8, 20.7 (8×CH_3), 9.6 (NHCOCH$_2$*C*H$_3$). HR-ESI-MS positive mode (*m*/*z*): calc. for $C_{47}H_{59}N_5O_{23}$ (1061.36) $[M+H]^+$=1062.3647, found: $[M+H]^+$=1062.3657.

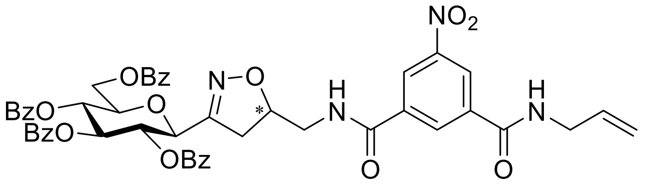

***N*^{1}-((3-(2’,3’,4’,6’-Tetra-*O*-benzoyl-β-D-glucopyranosyl)isoxazolin-5-yl)methyl)-*N*^{3}-(prop-2-en-1-yl)-5-nitroisophthalamides (6a)**

Prepared from oxime **1b** (5 equiv., 0.10 g, 0.16 mmol) and 5-nitro-*N*^{1},*N*^{3}-di(prop-2-en-1-yl)isophthalamide **2b** (0.01 g, 0.03 mmol) according to the General procedure I. Purified by column chromatography (from 1:3 to 1:1 EtOAc–hexane) to detect **6a** (diastereomeric ratio: 1:1) as a white amorphous product. R_f: 0.18 (1:1 EtOAc–hexane). HR-ESI-MS positive mode (*m*/*z*): calc. for $C_{49}H_{42}N_4O_{14}$ (910.27) $[M+Na]^+$=933.2590, found: $[M+Na]^+$=933.2585.

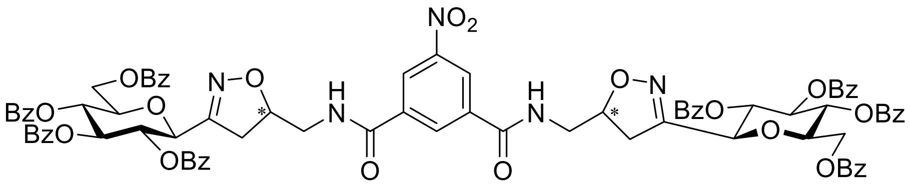

***N*[1],*N*[3]-Di-((3-(2',3',4',6'-tetra-*O*-benzoyl-β-D-glucopyranosyl)isoxazolin-5-yl)methyl)-5-nitroisophthalamides (7a)**

Prepared from the previous reaction mixture, from oxime **1b** (5 equiv., 0.10 g, 0.16 mmol) and 5-nitro-N^1,N^3-di(prop-2-en-1-yl)isophthalamide **2b** (0.01 g, 0.03 mmol) according to the General procedure I. Purified by column chromatography (from 1:3 to 1:1 EtOAc–hexane) to yield 15 mg (32%) of **7a** (diastereomeric ratio: 1.7:1.1:1) as a white amorphous product. R_f: 0.18 (1:1 EtOAc–hexane). **7a-I** ^{1}H NMR (500 MHz, $CDCl_3$) δ (ppm) 8.89–7.04 (45H, m, Ar, 2×NH), 6.05–5.96 (2H, m, 2×H-3'), 5.76–5.66 (2H, m, 2×H-4'), 5.53–5.43 (2H, m, 2×H-2'), 4.94–4.79 (2H, m, 2×H-5), 4.75 (2H, d, $J_{1',2'}$ 9.8 Hz, 2×H-1'), 4.67–4.59 (2H, m, 2×H-6$_a$'), 4.44 (2H, dd, $J_{5',6b'}$ 2.7, $J_{6a',6b'}$ 12.4 Hz, 2×H-6$_b$'), 4.24–4.15 (2H, m, 2×H-5'), 3.83–3.49 (4H, m, 2×CH_2), 3.26 (2H, dd, $J_{4a,4b}$ 17.2, $J_{4a,5}$ 10.7 Hz, 2×H-4$_a$), 3.13 (2H, dd, $J_{4b,5}$ 7.6 Hz, 2×H-4$_b$). ^{13}C NMR (125 MHz, $CDCl_3$) δ (ppm) 166.3, 165.8, 165.3, 165.0 (8×CO, 2×*C*ONHCH$_2$), 156.2 (2×C-3), 148.7–125.1 (Ar), 79.7 (2×C-5), 76.6 (2×C-5'), 74.1 (2×C-1'), 73.5 (2×C-3'), 70.2 (2×C-2'), 69.2 (2×C-4'), 63.0 (2×C-6'), 43.0 (2×*C*H$_2$NHCO), 35.8 (2×C-4). **7a-II** ^{1}H NMR (500 MHz, $CDCl_3$) δ (ppm) 8.89–7.04 (45H, m, Ar, 2×NH), 6.05–5.96 (2H, m, 2×H-3'), 5.76–5.66 (2H, m, 2×H-4'), 5.53–5.43 (2H, m, 2×H-2'), 4.94–4.79 (2H, m, 2×H-5), 4.69 (2H, d, $J_{1',2'}$ 9.1 Hz, 2×H-1'), 4.56 (2H, dd, $J_{5',6a'}$ 2.0, $J_{6a',6b'}$ 12.5 Hz, 2×H-6$_a$'), 4.30 (2H, dd, $J_{5',6b'}$ 4.0 Hz, 2×H-6$_b$'), 4.24–4.15 (2H, m, 2×H-5'), 3.83–3.49 (4H, m, 2×CH_2), 3.39 (2H, dd, $J_{4a,4b}$ 17.0, $J_{4a,5}$ 10.7 Hz, 2×H-4$_a$), 2.89 (2H, dd, $J_{4b,5}$ 7.2 Hz, 2×H-4$_b$). ^{13}C NMR (125 MHz, $CDCl_3$) δ (ppm) 166.3, 165.8, 165.3, 165.0 (8×CO, 2×*C*ONHCH$_2$), 156.4 (2×C-3), 148.7–125.1 (Ar), 79.6 (2×C-5), 76.4 (2×C-5'), 74.4 (2×C-1'), 73.4 (2×C-3'), 70.2 (2×C-2'), 69.2 (2×C-4'), 62.7 (2×C-6'), 43.3 (2×*C*H$_2$NHCO), 35.8 (2×C-4). **7a-III** ^{1}H NMR (500 MHz, $CDCl_3$) δ (ppm) 8.89–7.04

(45H, m, Ar, 2×NH), 6.05–5.96 (2H, m, 2×H-3'), 5.76–5.66 (2H, m, 2×H-4'), 5.53–5.43 (2H, m, 2×H-2'), 4.94–4.79 (2H, m, 2×H-5), 4.71 (2H, d, $J_{1',2'}$ 9.1 Hz, 2×H-1'), 4.79–4.67 (2H, m, 2×H-6_a'), 4.24 (2H, dd, $J_{5',6b'}$ 2.3, $J_{6a',6b'}$ 12.4 Hz, 2×H-6_b'), 4.24–4.15 (2H, m, 2×H-5'), 3.83–3.49 (4H, m, 2×CH_2NHCO), 3.34 (2H, dd, $J_{4a,4b}$ 17.4, $J_{4a,5}$ 10.7 Hz, 2×H-4_a), 2.87 (2H, dd, $J_{4b,5}$ 7.9 Hz, 2×H-4_b). ^{13}C NMR (125 MHz, $CDCl_3$) δ (ppm) 166.3, 165.8, 165.3, 165.0 (8×CO, 2×*C*ONHCH$_2$), 156.6 (2×C-3), 148.7–125.1 (Ar), 79.6 (2×C-5), 76.3 (2×C-5'), 74.0 (2×C-1'), 73.4 (2×C-3'), 70.0 (2×C-2'), 69.1 (2×C-4'), 62.2 (2×C-6'), 42.5 (2×*C*H$_2$NHCO), 35.3 (2×C-4). HR-ESI-MS positive mode (*m*/*z*): calc. for $C_{84}H_{69}N_5O_{24}$ (1531.43) $[M+Na]^{2+}$=788.7058, found: $[M+Na]^{2+}$=788.7046.

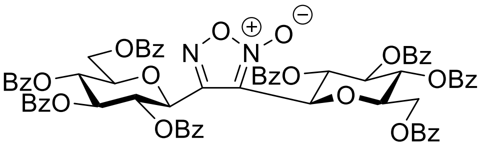

3,4-Di(2,3,4,6-tetra-*O*-benzoyl-β-D-glucopyranosyl)-1,2,5-oxadiazole-2-oxide (4b)

Prepared from the previous reaction mixture, from oxime **1b** (5 equiv., 0.10 g, 0.16 mmol) and 5-nitro-N^1,N^3-di(prop-2-en-1-yl)isophthalamide **2b** (0.01 g, 0.03 mmol) according to the General procedure I. Purified by column chromatography (from 1:3 to 1:1 EtOAc–hexane) to yield 47 mg (47%, The yield was calculated from the amount of oxime **1a**.) of **4b** as a white amorphous product. R_f: 0.29 (1:2 EtOAc–hexane); $[\alpha]_D$ -18 (*c* 0.14, CH_2Cl_2). ^{1}H NMR (500 MHz, $CDCl_3$) δ (ppm) 8.15–7.89 (8H, m, Ar), 7.87–7.70 (8H, m, Ar), 7.59–7.09 (24H, m, Ar), 6.18–6.09 (3H, m, H-2', H-3', H-3''), 6.07 (1H, pseudo t, $J_{2'',3''}$ 9.5 Hz, H-2''), 5.79 (1H, pseudo t, $J_{3',4'}$ 9.7, $J_{4',5'}$ 10.2 Hz, H-4'), 5.77 (1H, pseudo t, $J_{3'',4''}$ 9.7, $J_{4'',5''}$ 9.9 Hz, H-4''), 5.26 (1H, d, $J_{1',2'}$ 10.1 Hz, H-1'), 5.17 (1H, d, $J_{1'',2''}$ 9.4 Hz, H-1''), 4.87–4.81 (2H, m, H-6_a', H-6_a''), 4.81 (1H, dd, H-6_b'), 4.75 (1H, dd, $J_{6a'',6b''}$ 12.8 Hz, H-6_b''), 4.47 (1H, ddd, $J_{5',6a'}$ 2.8, $J_{5',6b'}$ 6.9 Hz, H-5'), 4.43 (1H, ddd, $J_{5'',6a''}$ 2.7, $J_{5'',6b''}$ 7.4 Hz, H-5''). ^{13}C NMR (125 MHz, $CDCl_3$) δ (ppm) 166.2, 165.9, 165.8, 165.4, 165.0, 164.9 (8×CO), 153.7 (C-4), 134.0–128.1 (Ar), 112.9 (C-3),

77.6 (C-5’, C-5”), 74.4 (C-1”), 73.7 (C-3’), 73.5 (C-3”), 72.0 (C-1’), 71.2 (C-2’), 71.0 (C-2”), 70.0 (C-4’), 69.9 (C-4”), 63.8 (C-6’, C-6”). NMR spectra are identical with those reported.[3]

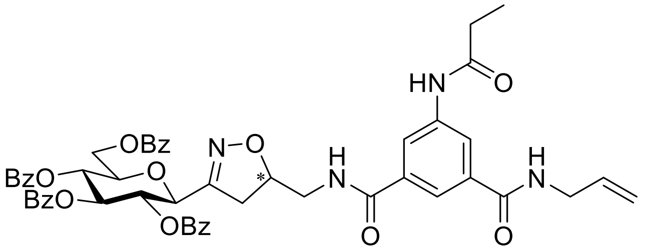

***N*[1]-((3-(2’,3’,4’,6’-Tetra-*O*-benzoyl-β-D-glucopyranosyl)isoxazolin-5-yl)methyl)-*N*[3]-(prop-2-en-1-yl)- 5-(propionamido)isophthalamides (6b)**

Prepared from oxime **1b** (5 equiv., 0.10 g, 0.16 mmol) and N^1,N^3-di(prop-2-en-1-yl)-5-(propionamido)isophthalamide **2c** (0.01 g, 0.03 mmol) according to the General procedure I. Purified by column chromatography (from 1:3 to 2:1 EtOAc–hexane) to yield 7 mg (24%) of **6b** (diastereomeric ratio: 1.2:1) as a white amorphous product. R_f: 0.50 (3:1 EtOAc–hexane). **6b-I** ^{1}H NMR (700 MHz, $CDCl_3$) δ (ppm) 8.33–7.22 (23H, m, Ar), 7.00–6.53 (3H, m, 3×NH), 6.05–5.95 (1H, m, H-3’), 5.93–5.84 (1H, m, $CONHCH_2C\textit{H}CH_2$), 5.71–5.66 (1H, m, H-4’), 5.54–5.45 (1H, m, H-2’), 5.28–5.05 (2H, m, $CONHC\textit{H}_2CHCH_2$), 4.92–4.77 (1H, m, H-5), 4.76–4.67 (1H, m, H-1’), 4.66–4.59 (1H, m, H-6_a’), 4.47–4.41 (1H, m, H-6_b’), 4.23–4.18 (1H, m, H-5’), 4.08–4.02 (2H, m, $CONHCH_2CHC\textit{H}_2$), 3.77–3.45 (2H, m, $C\textit{H}_2NHCO$), 3.25–3.17 (1H, m, H-4_a), 3.13–3.08 (1H, m, H-4_b), 2.34 (2H, q, *J* 7.7 Hz, $NHCOC\textit{H}_2CH_3$), 1.17 (3H, t, *J* 7.5 Hz, $NHCOCH_2C\textit{H}_3$). ^{13}C NMR (175 MHz, $CDCl_3$) δ (ppm) 172.7 ($NH\textit{C}OCH_2CH_3$), 167.8–164.8 (4×CO, $CH_2NH\textit{C}O$, $\textit{C}ONHCH_2CHCH_2$), 156.2 (C-3), 140.7–120.8 (Ar), 133.5 ($CONHCH_2\textit{C}HCH_2$), 117.1 ($CONH\textit{C}H_2CHCH_2$), 79.6 (C-5), 76.6 (C-5’), 74.5 (C-1’), 73.6 (C-3’), 70.2 (C-2’), 69.2 (C-4’), 63.1 (C-6’), 43.1 ($CONHCH_2CH\textit{C}H_2$), 42.8 ($\textit{C}H_2NHCO$), 35.9 (C-4), 30.7 ($NHCO\textit{C}H_2CH_3$), 9.6 ($NHCOCH_2\textit{C}H_3$). **6b-II** ^{1}H NMR (700 MHz, $CDCl_3$) δ (ppm) 8.33–7.22 (23H, m, Ar), 7.00–6.53 (3H, m, 3×NH), 6.05–5.95 (1H, m, H-3’), 5.83–5.78 (1H,

m, CONHCH$_2$C*H*CH$_2$), 5.76 (1H, pseudo t, $J_{3',4'}$ 9.7, $J_{4',5'}$ 9.5 Hz, H-4'), 5.75 (1H, pseudo t, $J_{1',2'}$ 9.7, $J_{2',3'}$ 9.7 Hz, H-2'), 5.28–5.05 (2H, m, CONHC*H$_2$*CHCH$_2$), 4.92–4.77 (1H, m, H-5), 4.76–4.67 (1H, m, H-1'), 4.55–4.49 (1H, m, H-6$_a$'), 4.30–4.24 (1H, m, H-6$_b$'), 4.17–4.11 (1H, m, H-5'), 4.00–3.95 (2H, m, CONHCH$_2$CHC*H$_2$*), 3.77–3.45 (2H, m, C*H$_2$*NHCO), 3.41–3.30 (1H, m, H-4$_a$), 3.00–2.91 (1H, m, H-4$_b$), 2.41 (2H, dq, *J* 3.2, 7.6 Hz, NHCOC*H$_2$*CH$_3$), 1.22 (3H, t, *J* 7.5 Hz, NHCOCH$_2$C*H$_3$*). ^{13}C NMR (175 MHz, CDCl$_3$) δ (ppm) 172.7 (NH*C*OCH$_2$CH$_3$), 167.8–164.8 (4×CO, CH$_2$NH*C*O, *C*ONHCH$_2$CHCH$_2$), 156.1 (C-3), 140.7–120.8 (Ar), 133.5 (CONHCH$_2$*C*HCH$_2$), 117.2 (CONH*C*H$_2$CHCH$_2$), 79.9 (C-5), 76.2 (C-5'), 74.2 (C-1'), 73.6 (C-3'), 70.2 (C-2'), 69.3 (C-4'), 62.8 (C-6'), 43.1 (CONHCH$_2$CH*C*H$_2$), 42.9 (*C*H$_2$NHCO), 35.5 (C-4), 30.6 (NHCO*C*H$_2$CH$_3$), 9.6 (NHCOCH$_2$*C*H$_3$). HR-ESI-MS positive mode (*m*/*z*): calc. for $C_{52}H_{48}N_4O_{13}$ (936.32) $[M+H]^+$=937.3291, found: $[M+H]^+$=937.3294.

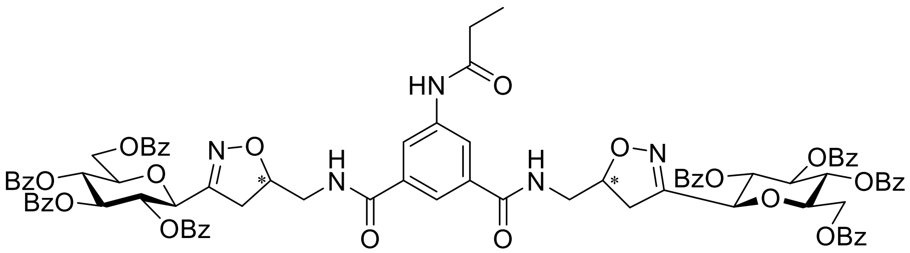

***N*^{1},*N*^{3}-Di-((3-(2',3',4',6'-tetra-*O*-benzoyl-β-D-glucopyranosyl)isoxazolin-5-yl)methyl)-5-(propionamido)isophthalamides (7b)**

Prepared from the previous reaction mixture, from oxime **1b** (5 equiv., 0.10 g, 0.16 mmol) and *N*^{1},*N*^{3}-di(prop-2-en-1-yl)-5-(propionamido)isophthalamide **2c** (0.01 g, 0.03 mmol) according to the General procedure I. Purified by column chromatography (from 1:3 to 2:1 EtOAc–hexane) to yield 17 mg (34%) of **7b** (diastereomeric ratio: 3.2:1.8:1) as a white amorphous product. R$_f$: 0.43 (3:1 EtOAc–hexane). **7b-I** ^{1}H NMR (700 MHz, CDCl$_3$) δ (ppm) 8.33–7.22 (43H, m, Ar), 7.00–6.53 (3H, m, 3×NH), 6.00 (2H, pseudo t, $J_{3',4'}$ 9.6 Hz, 2×H-3'), 5.69 (2H, pseudo t, $J_{4',5'}$ 9.9 Hz, 2×H-4'), 5.50 (2H, pseudo t, $J_{2',3'}$ 9.8 Hz, 2×H-2'), 4.92–4.77 (2H, m, 2×H-5), 4.71

(2H, d, $J_{1',2'}$ 9.9 Hz, 2×H-1'), 4.63 (2H, dd, $J_{5',6a'}$ 2.7, $J_{6a',6b'}$ 12.3 Hz, 2×H-6$_a$'), 4.44 (2H, dd, $J_{5',6b'}$ 5.0 Hz, 2×H-6$_b$'), 4.23–4.18 (2H, m, 2×H-5'), 3.77–3.45 (4H, m, 2×CH_2), 3.22 (2H, dd, $J_{4a,4b}$ 17.2, $J_{4a,5}$ 9.8 Hz, 2×H-4$_a$), 3.12 (2H, dd, $J_{4b,5}$ 7.8 Hz, 2×H-4$_b$), 2.34 (2H, q, *J* 7.7 Hz, NHCOCH_2CH$_3$), 1.17 (3H, t, *J* 7.5 Hz, NHCOCH$_2$CH_3). ^{13}C NMR (175 MHz, $CDCl_3$) δ (ppm) 172.7 (NH*C*OCH$_2$CH$_3$), 167.8–164.8 (8×CO, 2×*C*ONHCH$_2$), 155.9 (2×C-3), 140.7–120.8 (Ar), 79.6 (2×C-5), 76.6 (2×C-5'), 74.5 (2×C-1'), 73.6 (2×C-3'), 69.9 (2×C-2'), 69.3 (2×C-4'), 63.1 (2×C-6'), 42.8 (2×*C*H$_2$NHCO), 35.9 (2×C-4), 30.7 (NHCO*C*H$_2$CH$_3$), 9.6 (NHCOCH$_2$*C*H$_3$). **7b-II** ^{1}H NMR (700 MHz, $CDCl_3$) δ (ppm) 8.33–7.22 (43H, m, Ar), 7.00–6.53 (3H, m, 3×NH), 6.05–5.95 (2H, m, 2×H-3'), 5.76 (2H, pseudo t, $J_{3',4'}$ 9.7, $J_{4',5'}$ 9.5 Hz, 2×H-4'), 5.75 (1H, pseudo t, $J_{1',2'}$ 9.7, $J_{2',3'}$ 9.7 Hz, 2×H-2'), 4.92–4.77 (2H, m, 2×H-5), 4.76–4.67 (2H, m, 2×H-1'), 4.52 (2H, dd, $J_{5',6a'}$ 2.8, $J_{6a',6b'}$ 12.2 Hz, 2×H-6$_a$'), 4.24 (2H, dd, $J_{5',6b'}$ 4.0 Hz, 2×H-6$_b$'), 4.17–4.11 (2H, m, 2×H-5'), 3.77–3.45 (4H, m, 2×CH_2), 3.41–3.30 (2H, m, 2×H-4$_a$), 3.00–2.91 (2H, m, 2×H-4$_b$), 2.41 (2H, dq, *J* 3.2, 7.6 Hz, NHCOCH_2CH$_3$), 1.22 (3H, t, *J* 7.5 Hz, NHCOCH$_2$CH_3). ^{13}C NMR (175 MHz, $CDCl_3$) δ (ppm) 172.7 (NH*C*OCH$_2$CH$_3$), 167.8–164.8 (8×CO, 2×*C*ONHCH$_2$), 156.2 (2×C-3), 140.7–120.8 (Ar), 79.8 (2×C-5), 76.2 (2×C-5'), 74.2 (2×C-1'), 73.7, 73.6 (2×C-3'), 70.2 (2×C-2'), 69.2 (2×C-4'), 62.8 (2×C-6'), 42.9 (2×*C*H$_2$NHCO), 35.5 (2×C-4), 30.7 (NHCO*C*H$_2$CH$_3$), 9.6 (NHCOCH$_2$*C*H$_3$). **7b-III** ^{1}H NMR (700 MHz, $CDCl_3$) δ (ppm) 8.33–7.22 (43H, m, Ar), 7.00–6.53 (3H, m, 3×NH), 6.05–5.95 (2H, m, 2×H-3'), 5.69 (2H, pseudo t, $J_{3',4'}$ 9.7, $J_{4',5'}$ 9.7 Hz, 2×H-4'), 5.54–5.45 (2H, m, 2×H-2'), 4.92–4.77 (2H, m, 2×H-5), 4.76–4.67 (2H, m, 2×H-1'), 4.64 (2H, dd, $J_{5',6a'}$ 2.9 Hz, 2×H-6$_a$'), 4.30–4.24 (2H, m, 2×H-6$_b$'), 4.17–4.11 (2H, m, 2×H-5'), 3.77–3.45 (4H, m, 2×CH_2), 3.41–3.30 (2H, m, 2×H-4$_a$), 3.00–2.91 (2H, m, 2×H-4$_b$), 2.34 (2H, q, *J* 8.0 Hz, NHCOCH_2CH$_3$), 1.17 (3H, t, *J* 7.5 Hz, NHCOCH$_2$CH_3). ^{13}C NMR (175 MHz, $CDCl_3$) δ (ppm) 172.7 (NH*C*OCH$_2$CH$_3$), 167.8–164.8 (8×CO, 2×*C*ONHCH$_2$), 156.1 (2×C-3), 140.7–120.8 (Ar), 80.0 (2×C-5), 76.2 (2×C-5'), 74.5 (2×C-1'), 73.7 (2×C-3'), 70.0 (2×C-2'), 69.2 (2×C-4'), 62.7 (2×C-6'), 43.1

($2\times CH_2$NHCO), 35.4 (2×C-4), 30.6 (NHCOCH_2CH_3), 9.7 (NHCOC$H_2$$CH_3$). HR-ESI-MS positive mode (*m/z*): calc. for $C_{87}H_{75}N_5O_{23}$ (1557.49) $[M+H]^+$=1558.4926, found: $[M+H]^+$=1558.4935.

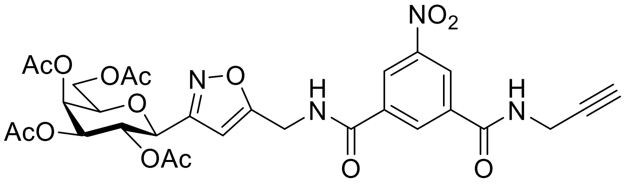

***N*[1]-((3-(2',3',4',6'-Tetra-*O*-acetyl-β-D-galactopyranosyl)isoxazol-5-yl)methyl)-*N*[3]-(prop-2-yn-1-yl)-5-nitroisophthalamide (9a)**

Prepared from oxime **1a** (5 equiv., 0.10 g, 0.27 mmol) and 5-nitro-N^1,N^3-di(prop-2-yn-1-yl)isophthalamide **8a**[4] (0.02 g, 0.05 mmol) according to the General procedure I. Purified by column chromatography (from 2:1 to 1:0 EtOAc–hexane) to yield 13 mg (24%) of **9a** as a yellow amorphous product. R_f: 0.18 (2:1 EtOAc–hexane); $[\alpha]_D$ -8 (*c* 0.21, CH_2Cl_2). ^1H NMR (400 MHz, $CDCl_3$) δ (ppm) 8.82 (1H, t, *J* 1.7 Hz, Ar), 8.81 (1H, t, *J* 1.8 Hz, Ar), 8.62 (1H, t, *J* 1.6 Hz, Ar), 7.36 (1H, pseudo t, $J_{CHa,NH}$ 5.4, $J_{CHb,NH}$ 5.8 Hz, CH_2N*H*CO), 6.98 (1H, pseudo t, $J_{NH,CHa}$ 4.6, $J_{NH,CHb}$ 5.2 Hz, CON*H*CH_2CCH), 6.45 (1H, s, H-4), 5.53 (1H, dd, $J_{4',5'}$ 1.0 Hz, H-4'), 5.34 (1H, pseudo t, $J_{2',3'}$ 10.2 Hz, H-2'), 5.18 (1H, dd, $J_{3',4'}$ 3.4 Hz, H-3'), 4.88–4.72 (2H, m, CH_2NHCO), 4.62 (1H, d, $J_{1',2'}$ 9.8 Hz, H-1'), 4.33–4.24 (2H, m, CONHCH_2CCH), 4.19–4.03 (3H, m, H-5', H-6_a', H-6_b'), 2.33 (1H, pseudo t, $J_{CHa,CH}$ 2.5, $J_{CHb,CH}$ 2.6 Hz, CONHCH_2CC*H*), 2.19, 2.04, 2.00, 1.97 (12H, 4s, $4\times CH_3$). ^{13}C NMR (90 MHz, $CDCl_3$) δ (ppm) 170.7, 170.3, 170.2, 170.0 (4×CO), 168.6 (C-5), 164.4 (CH_2NH*C*O), 163.9 (*C*ONHCH_2CCH), 161.4 (C-3), 149.3–125.0 (Ar), 101.6 (C-4), 78.7 (CONHCH_2*C*CH), 75.1 (C-5'), 73.3 (C-1'), 72.8 (CONHCH_2C*C*H), 71.7 (C-3'), 69.7 (C-2'), 69.6 (C-4'), 61.8 (C-6'), 36.1 (*C*H_2NHCO), 30.3 (CONH*C*H_2CCH), 20.9, 20.8, 20.7 ($4\times CH_3$). HR-ESI-MS positive mode (*m/z*): calc. for $C_{29}H_{30}N_4O_{14}$ (658.18) $[M+Na]^+$=681.1651, found: $[M+Na]^+$=681.1652.

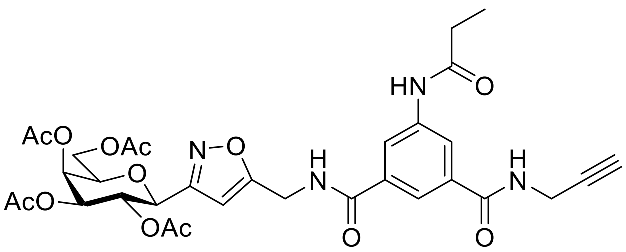

***N*[1]-((3-(2’,3’,4’,6’-Tetra-*O*-acetyl-β-D-galactopyranosyl)isoxazol-5-yl)methyl)-*N*[3]-(prop-2-yn-1-yl)-5-(propionamido)isophthalamide (9b)**

Prepared from oxime **1a** (5 equiv., 0.10 g, 0.27 mmol) and N^1,N^3-di(prop-2-yn-1-yl)-5-(propionamido)isophthalamide **8b**[5] (0.02 g, 0.05 mmol) according to the General procedure I. Purified by column chromatography (from 2:1 to 4:1 EtOAc–hexane) to yield 22 mg (60%) of **9b** as a yellow amorphous product. R_f: 0.21 (2:1 EtOAc–hexane); $[\alpha]_D$ -1 (*c* 0.21, CH_2Cl_2). ^{1}H NMR (400 MHz, $CDCl_3$) δ (ppm) 8.29 (1H, bs, N*H*$COCH_2CH_3$), 8.20 (1H, s, Ar), 8.14 (1H, s, Ar), 7.94 (1H, s, Ar), 7.61 (1H, pseudo t, $J_{CHa,NH}$ 5.2, $J_{CHb,NH}$ 5.4 Hz, CH_2N*H*CO), 7.11 (1H, pseudo t, $J_{NH,CHa}$ 4.8, $J_{NH,CHb}$ 5.0 Hz, CON*H*CH_2CCH), 6.41 (1H, s, H-4), 5.51 (1H, dd, $J_{4',5'}$ 0.6 Hz, H-4’), 5.35 (1H, pseudo t, $J_{2',3'}$ 10.1 Hz, H-2’), 5.17 (1H, dd, $J_{3',4'}$ 3.3 Hz, H-3’), 4.83–4.73 (2H, m, C*H*$_2$NHCO), 4.61 (1H, d, $J_{1',2'}$ 9.8 Hz, H-1’), 4.31–4.17 (2H, m, CONHC*H*$_2$CCH), 4.16–4.03 (3H, m, H-5’, H-6_a’, H-6_b’), 2.44 (2H, q, *J* 7.5 Hz, NHCOC*H*$_2$$CH_3$), 2.29 (1H, pseudo t, $J_{CHa,CH}$ 2.1, $J_{CHb,CH}$ 2.1 Hz, CONHCH_2CC*H*), 2.18, 2.03, 1.99, 1.95 (12H, 4s, 4×CH_3), 1.23 (3H, t, *J* 7.6 Hz, NHCOCH_2C*H*$_3$). ^{13}C NMR (100 MHz, $CDCl_3$) δ (ppm) 173.3 (NH*C*OCH_2CH_3), 170.7, 170.3, 170.2, 170.0 (4×CO), 169.5 (C-5), 166.7 (CH_2NH*C*O), 166.2 (*C*ONHCH_2CCH), 161.2 (C-3), 139.4–121.0 (Ar), 101.2 (C-4), 79.3 (CONHCH_2*C*CH), 75.0 (C-5’), 73.3 (C-1’), 72.2 (CONHCH_2C*C*H), 71.7 (C-3’), 67.9 (C-2’), 67.6 (C-4’), 61.7 (C-6’), 36.0 (*C*H_2NHCO), 30.7 (NHCO*C*H_2CH_3), 30.0 (CONH*C*H_2CCH), 20.8, 20.7 (4×CH_3), 9.6 (NHCOCH_2*C*H_3). HR-ESI-MS positive mode (*m*/*z*): calc. for $C_{32}H_{36}N_4O_{13}$ (684.22) $[M+H]^+$=685.2352, found: $[M+H]^+$=685.2354.

General procedure II for the synthesis of 1-(2',3',4',6'-tetra-*O*-acetyl-β-D-glycopyranosyl)-1,2,3-triazole derivatives 11 and 12

2,3,4,6-Tetra-*O*-acetyl-β-D-glycopyranosyl azide (**10a**[6] and **10b**[7] 1.25 or 2.5 mmol (indicated for the particular compounds)) and N^1,N^3-di(prop-2-yn-1-yl)-5-(propionamido)isophthalamide (**8b**,[5] 1 mmol) or N^1-((1-(2',3',4',6'-tetra-*O*-acetyl-β-D-glucopyranosyl)-1*H*-1,2,3-triazol-4-yl)methyl)-N^3-(prop-2-yn-1-yl)-5-(propionamido)isophthalamide (**11**, 1 mmol) were dissolved in 2:1 acetone–H_2O (10 mL and 5 mL) then copper sulfate pentahydrate ($CuSO_4 \bullet 5{\times}H_2O$, 10 mol%) and sodium-L-ascorbate (Na-L-ascorbate, 25 mol%) were added to the solution. The suspension was stirred at room temperature. When TLC (10:1 EtOAc–hexane for) indicated complete consumption of the starting compound (~1 day). The reaction mixture was diluted with water (500 mL) and washed with ethyl acetate (3 × 200 mL) and then dried on anhydrous magnesium sulfate. The solvent was removed under reduced pressure, and the residue was purified by silica gel column chromatography with eluents indicated for the particular compounds to give 1-(2',3',4',6'-tetra-*O*-acetyl-β-D-glycopyranosyl)-1,2,3-triazole derivatives **11** and **12**.

Characterization of 1,2,3-triazole derivatives 11 and 12

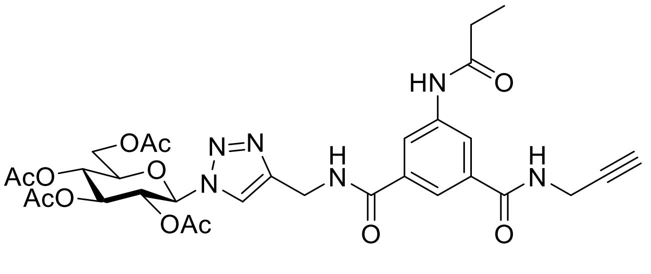

***N*^{1}-((1-(2',3',4',6'-Tetra-*O*-acetyl-β-D-glucopyranosyl)-1*H*-1,2,3-triazol-4-yl)methyl)-*N*^{3}-(prop-2-yn-1-yl)-5-(propionamido)isophthalamide (11)**

Prepared from 2,3,4,6-tetra-*O*-acetyl-β-D-glucopyranosyl azide **10a** (1.25 equiv., 0.04 g, 0.12 mmol) and N^1,N^3-di(prop-2-yn-1-yl)-5-(propionamido)isophthalamide **8b** (0.03 g, 0.10 mmol)

according to the General procedure II. Purified by column chromatography (from 10:1 to 60:1 EtOAc–hexane) to yield 20 mg (30%) of **11** as a pale yellow amorphous product. R_f: 0.16 (10:1 EtOAc–hexane); $[\alpha]_D$ -18 (*c* 0.24, CH_2Cl_2). ^{1}H NMR (700 MHz, $CDCl_3$) δ (ppm) 8.13 (1H, s, Ar), 8.06 (1H, s, Ar), 8.01 (1H, bs, N*H*COCH$_2$CH$_3$), 7.91 (1H, s, H-5), 7.86 (1H, s, Ar), 7.37 (1H, bs, CH$_2$N*H*CO), 6.98 (1H, bs, CON*H*CH$_2$CCH), 5.88 (1H, d, $J_{1',2'}$ 9.3 Hz, H-1'), 5.49 (1H, pseudo t, $J_{2',3'}$ 9.3 Hz, H-2'), 5.42 (1H, pseudo t, $J_{3',4'}$ 9.4 Hz, H-3'), 5.27 (1H, pseudo t, $J_{4',5'}$ 9.9 Hz, H-4'), 4.77–4.63 (2H, m, C*H$_2$*NHCO), 4.29 (1H, dd, $J_{6a',6b'}$ 12.6 Hz, H-6$_a$'), 4.26–4.20 (2H, m, CONHC*H$_2$*CCH), 4.17 (1H, dd, H-6$_b$'), 4.03 (1H, ddd, $J_{5',6b'}$ 1.8, $J_{5',6a'}$ 5.0 Hz, H-5'), 2.44 (2H, q, *J* 7.6 Hz, NHCOC*H$_2$*CH$_3$), 2.28 (1H, pseudo t, $J_{CHa,CH}$ 2.5, $J_{CHb,CH}$ 2.5 Hz, CONHCH$_2$CC*H*), 2.07, 2.06, 2.02, 1.86 (12H, 4s, 4×CH$_3$), 1.24 (3H, t, *J* 7.6 Hz, NHCOCH$_2$C*H$_3$*). ^{13}C NMR (175 MHz, $CDCl_3$) δ (ppm) 173.1 (NH*C*OCH$_2$CH$_3$), 170.8, 170.1, 169.5, 169.4 (4×CO), 166.6 (CH$_2$NH*C*O), 166.2 (*C*ONHCH$_2$CCH), 145.5 (C-4), 139.7–120.7 (Ar), 121.3 (C-5), 85.9 (C-1'), 79.5 (CONHCH$_2$*C*CH), 75.3 (C-5'), 72.8 (C-3'), 72.1 (CONHCH$_2$C*C*H), 70.6 (C-2'), 67.8 (C-4'), 61.7 (C-6'), 35.7 (*C*H$_2$NHCO), 30.7 (NHCO*C*H$_2$CH$_3$), 29.9 (CONH*C*H$_2$CCH), 20.9, 20.7, 20.4 (4×CH$_3$), 9.6 (NHCOCH$_2$*C*H$_3$). HR-ESI-MS positive mode (*m*/*z*): calc. for $C_{31}H_{36}N_6O_{12}$ (684.24) $[M+H]^+$=685.2464, found: $[M+H]^+$=685.2467.

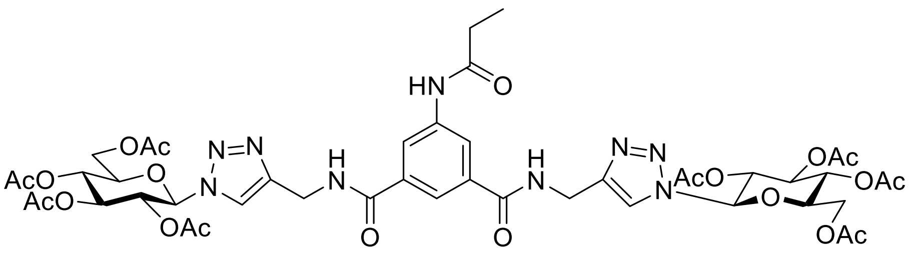

***N*^{1},*N*^{3}-Di-((1-(2',3',4',6'-tetra-*O*-acetyl-β-D-glucopyranosyl)-1*H*-1,2,3-triazol-4-yl)methyl)-5-(propionamido)isophthalamide (12a)**

Prepared from the previous reaction mixture, from 2,3,4,6-tetra-*O*-acetyl-β-D-glucopyranosyl azide **10a** (1.25 equiv., 0.04 g, 0.12 mmol) and N^1,N^3-di(prop-2-yn-1-yl)-5-(propionamido)isophthalamide **8b** (0.03 g, 0.10 mmol) according to the General procedure II. Purified by column chromatography (from 10:1 to 60:1 EtOAc–hexane) to yield 34 mg (33%) of **12a** as a pale yellow amorphous product. R_f: 0.05 (60:1 EtOAc–hexane); $[\alpha]_D$ -20 (*c* 0.12, CH_2Cl_2). ^{1}H NMR (700 MHz, $CDCl_3$) δ (ppm) 8.20 (1H, bs, N*H*$COCH_2CH_3$), 8.01 (2H, s, 2×H-5), 7.88 (2H, bs, Ar), 7.77–7.60 (3H, m, Ar, 2×NH), 5.92 (2H, d, $J_{1',2'}$ 9.3 Hz, 2×H-1'), 5.60 (2H, pseudo t, $J_{2',3'}$ 10.0 Hz, 2×H-2'), 5.43 (2H, pseudo t, $J_{3',4'}$ 9.6 Hz, 2×H-3'), 5.28 (2H, pseudo t, $J_{4',5'}$ 10.0 Hz, 2×H-4'), 4.79–4.61 (4H, m, 2×CH_2), 4.29 (2H, dd, $J_{6a',6b'}$ 12.4 Hz, 2×H-6_a'), 4.14 (2H, dd, 2×H-6_b'), 4.05 (2H, ddd, $J_{5',6b'}$ 1.5, $J_{5',6a'}$ 4.7 Hz, 2×H-5'), 2.49–2.38 (2H, m, NHCOC*H*$_2CH_3$), 2.07, 2.02, 1.83 (24H, 8s, 8×CH_3), 1.23 (3H, t, *J* 7.5 Hz, $NHCOCH_2C$*H*$_3$). ^{13}C NMR (175 MHz, $CDCl_3$) δ (ppm) 173.2 (NH*C*OCH_2CH_3), 170.7, 170.1, 169.6, 169.5 (8×CO), 166.7 (2×*C*ONHCH_2), 146.0 (2×C-4), 140.0–121.2 (Ar), 121.4 (2×C-5), 85.9 (2×C-1'), 75.2 (2×C-5'), 72.9 (2×C-3'), 70.7 (2×C-2'), 67.9 (2×C-4'), 61.8 (2×C-6'), 35.6 (2×*C*H_2NHCO), 30.7 (NHCO*C*H_2CH_3), 20.8, 20.7, 20.4 (8×CH_3), 9.6 ($NHCOCH_2$*C*H_3). NMR spectra are identical with those reported.[8]

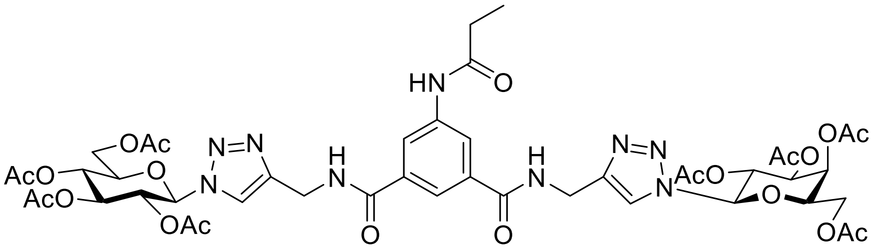

***N*^{1}-((1-(2',3',4',6'-Tetra-*O*-acetyl-β-D-glucopyranosyl)-1*H*-1,2,3-triazol-4-yl)methyl)-*N*^{3}-((1-(2''',3''',4''',6'''-tetra-*O*-acetyl-β-D-galactopyranosyl)-1''*H*-1'',2'',3''-triazol-4-yl)methyl)-5-(propionamido)isophthalamide (12b)**

Prepared from 2,3,4,6-tetra-*O*-acetyl-β-D-galactopyranosyl azide **10b** (2.5 equiv., 0.01 g, 0.03 mmol) and N^1-((1-(2',3',4',6'-tetra-*O*-acetyl-β-D-glucopyranosyl)-1*H*-1,2,3-triazol-4-yl]methy))-N^3-(prop-2-yn-1-yl)-5-(propionamido)isophthalamide **11** (0.01 g, 0.01 mmol) according to the General procedure II. Purified by column chromatography (from 10:1 to 60:1 EtOAc–hexane) to yield 8 mg (55%) of **12b** as a pale yellow amorphous product. R_f: 0.08 (10:1 EtOAc–hexane); $[\alpha]_D$ –20 (*c* 0.12, CH_2Cl_2). ^{1}H NMR (700 MHz, $CDCl_3$) δ (ppm) 8.03 (1H, bs, N*H*$COCH_2CH_3$), 8.02–7.95 (3H, m, Ar, H-5, H-5''), 7.93 (1H, s, Ar), 7.76 (1H, s, Ar), 7.64 (1H, bs, CON*H*CH_2), 7.58 (1H, bs, CON*H*CH_2), 5.90 (1H, d, $J_{1',2'}$ 10.0 Hz, H-1'), 5.88 (1H, d, $J_{1''',2'''}$ 9.8 Hz, H-1'''), 5.59 (1H, pseudo t, $J_{2',3'}$ 9.4 Hz, H-2'), 5.57 (1H, pseudo t, $J_{2''',3'''}$ 9.4 Hz, H-2'''), 5.55 (1H, dd, $J_{4''',5'''}$ 0.8 Hz, H-4'''), 5.43 (1H, pseudo t, $J_{3',4'}$ 9.8 Hz, H-3'), 5.29 (1H, pseudo t, $J_{4',5'}$ 9.5 Hz, H-4'), 5.27 (1H, dd, $J_{3''',4'''}$ 3.1 Hz, H-3'''), 4.76–4.64 (4H, m, 2×CH_2), 4.31–4.24 (2H, m, H-5''', H-6_a'''), 4.19 (1H, dd, $J_{6a',6b'}$ 11.6 Hz, H-6_a'), 4.18–4.13 (2H, m, H-6_b', H-6_b'''), 4.03 (1H, ddd, $J_{5',6b'}$ 1.5, $J_{5',6a'}$ 6.1 Hz, H-5'), 2.48–2.39 (2H, m, $NHCOCH_2CH_3$ with *H*$_2$), 2.22, 2.07, 2.02, 2.01, 1.85, 1.83 (24H, 8s, 8×CH_3), 1.23 (3H, t, *J* 7.5 Hz, $NHCOCH_2C\mathit{H}_3$). ^{13}C NMR (175 MHz, $CDCl_3$) δ (ppm) 173.0 (NH*C*OCH_2CH_3), 170.8, 170.7, 170.5, 170.2, 170.1, 170.0, 169.5, 169.4 (8×CO), 166.7, 166.6 (2×*C*ONHCH_2), 145.8, 145.6 (C-4, C-4''), 139.1–121.2 (Ar), 121.4 (C-5, C-5''), 86.4 (C-1'''), 85.9 (C-1'), 75.3 (C-5'), 74.2 (C-5'''), 72.8 (C-3'), 70.9 (C-3'''), 70.6 (C-2'), 68.2 (C-2'''), 67.9 (C-4'), 67.0 (C-4'''), 61.8 (C-6'), 61.3 (C-6'''), 35.7, 35.6 (2×*C*H_2NHCO), 29.8 (NHCO*C*H_2CH_3), 20.8, 20.7, 20.4 (8×CH_3), 9.6 ($NHCOCH_2$*C*H_3). HR-ESI-MS positive mode (*m*/*z*): calc. for $C_{45}H_{55}N_9O_{21}$ (1057.35) $[M+H]^+$=1058.3585, found: $[M+H]^+$=1058.3580.

General procedure III for the synthesis 3-(2',3',4',6'-tetra-*O*-acyl-β-D-glycopyranosyl)-5-(4-(1-(2''',3''',4''',6'''-tetra-*O*-acetyl-β-D-glycopyranosyl)-1''*H*-1'',2'',3''-triazol-4-yl))phenylisoxazoles 14

2,3,4,6-Tetra-*O*-acetyl-β-D-glycopyranosyl azide (**10a**[6] and **10b**[7] 1 mmol) and 3-(2',3',4',6'-tetra-*O*-acyl-β-D-glycopyranosyl)-5-(4-ethynylphenyl)isoxazole (**13a** and **13b**,[3] 1 mmol) were dissolved in *N*,*N*-dimethylformamide (DMF, 50 mL for **13a**, 172 mL for **13b**), then copper sulfate pentahydrate ($CuSO_4 \cdot 5\times H_2O$, 10–62 mol%, indicated for the particular compounds) and sodium-L-ascorbate (Na-L-ascorbate, 20–226 mol%, indicated for the particular compounds) were added to the solution. The suspension was stirred and heated to 100°C. When TLC (1:2 EtOAc–hexane) indicated complete consumption of the starting compound (~2 days), the mixture was cooled down. The reaction mixture was diluted with water (500 mL) and washed with ethyl acetate (3 × 200 mL) and then dried on anhydrous magnesium sulfate. The solvent was removed under reduced pressure, and the residue was purified by silica gel column chromatography with eluents indicated for the particular compounds to give 3-(2',3',4',6'-tetra-*O*-acyl-β-D-glycopyranosyl)-5-(4-(1-(2''',3''',4''',6'''-tetra-*O*-acetyl-β-D-glycopyranosyl)-1''*H*-1'',2'',3''-triazol-4-yl))phenylisoxazoles **14**.

Characterization of divalent triazole-isoxazole derivatives 14

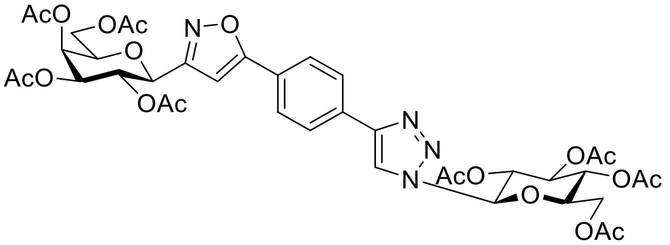

3-(2',3',4',6'-Tetra-*O*-acetyl-β-D-galactopyranosyl)-5-(4-(1-(2''',3''',4''',6'''-tetra-*O*-acetyl-β-D-glucopyranosyl)-1''*H*-1'',2'',3''-triazol-4-yl))phenylisoxazole (14a)

Prepared from 2,3,4,6-tetra-*O*-acetyl-β-D-glucopyranosyl azide **10a** (1 equiv., 0.04 g, 0.10 mmol), 3-(2',3',4',6'-tetra-*O*-acetyl-β-D-galactopyranosyl)-5-(4-ethynylphenyl)isoxazole **13a** (0.05 g, 0.10 mmol), $CuSO_4 \cdot 5 \times H_2O$ (3.0 mg, 10 mol%) and Na-L-ascorbate (5.0 mg, 20 mol%) according to the General procedure III. Purified by column chromatography (1:1 EtOAc–hexane) to yield 56 mg (87%) of **14a** as a yellow amorphous product. R_f: 0.28 (2:1 EtOAc–hexane); $[\alpha]_D$ –69 (*c* 0.19, CH_2Cl_2). ^{1}H NMR (500 MHz, $CDCl_3$) δ (ppm) 8.10 (1H, s, H-5''), 7.95 (2H, d, *J* 7.9 Hz, Ar), 7.86 (2H, d, *J* 8.1 Hz, Ar), 6.72 (1H, s, H-4), 5.96 (1H, d, $J_{1''',2'''}$ 9.2 Hz, H-1'''), 5.56 (1H, dd, $J_{4',5'}$ 0.5 Hz, H-4'), 5.53 (1H, pseudo t, $J_{2''',3'''}$ 9.4 Hz, H-2'''), 5.47 (1H, pseudo t, $J_{2',3'}$ 10.1 Hz, H-2'), 5.47 (1H, pseudo t, $J_{3''',4'''}$ 9.5 Hz, H-3'''), 5.28 (1H, pseudo t, $J_{4''',5'''}$ 9.7 Hz, H-4'''), 5.22 (1H, dd, $J_{3',4'}$ 2.9 Hz, H-3'), 4.70 (1H, d, $J_{1',2'}$ 9.9 Hz, H-1'), 4.35 (1H, dd, $J_{6a''',6b'''}$ 12.5 Hz, H-6_a'''), 4.23–4.10 (3H, m, H-6_a', H-6_b', H-6_b'''), 4.13 (1H, ddd, $J_{5',6a'}$ 6.1, $J_{5',6b'}$ 6.7 Hz, H-5'), 4.06 (1H, ddd, $J_{5''',6a'''}$ 5.0, $J_{5''',6b'''}$ 1.6 Hz, H-5'''), 2.22, 2.10, 2.09, 2.06, 2.05, 2.01, 1.99, 1.91 (24H, 8s, 8×CH_3). ^{13}C NMR (125 MHz, $CDCl_3$) δ (ppm) 170.6, 170.3, 170.2, 170.0, 169.6, 169.5, 169.2 (8×CO), 170.1 (C-5), 161.6 (C-3), 147.6 (C-4''), 132.0–126.3 (Ar), 118.5 (C-5''), 98.2 (C-4), 86.0 (C-1'''), 75.4 (C-5'''), 75.1 (C-5'), 73.4 (C-1'), 72.8 (C-3'''), 71.8 (C-3'), 70.4 (C-2'''), 67.8 (C-4'''), 67.7 (C-2', C-4'), 61.8 (C-6'''), 61.7 (C-6'), 20.8, 20.7, 20.6, 20.3 (8×CH_3). HR-ESI-MS positive mode (*m/z*): calc. for $C_{39}H_{44}N_4O_{19}$ (872.26) $[M+Na]^+$=895.2492, found: $[M+Na]^+$=895.2485.

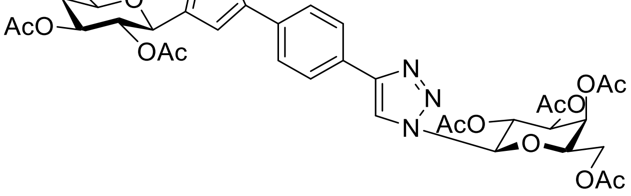

3-(2',3',4',6'-Tetra-*O*-acetyl-β-D-galactopyranosyl)-5-(4-(1-(2''',3''',4''',6'''-tetra-*O*-acetyl-β-D-galactopyranosyl)-1''*H*-1'',2'',3''-triazol-4-yl))phenylisoxazole (14b)

Prepared from 2,3,4,6-tetra-*O*-acetyl-β-D-galactopyranosyl azide **10b** (1 equiv., 0.05 g, 0.13 mmol), 3-(2',3',4',6'-tetra-*O*-acetyl-β-D-galactopyranosyl)-5-(4-ethynylphenyl)isoxazole **13a** (0.06 g, 0.13 mmol), $CuSO_4 \cdot 5\times H_2O$ (3.1 mg, 10 mol%) and Na-L-ascorbate (5.0 mg, 20 mol%) according to the General procedure III. Purified by column chromatography (from 1:1 to 2:1 EtOAc–hexane) to yield 43 mg (39%) of **14b** as a white amorphous product. R_f: 0.35 (2:1 EtOAc–hexane); $[\alpha]_D$ –62 (*c* 0.30, CH_2Cl_2). ^{1}H NMR (700 MHz, $CDCl_3$) δ (ppm) 8.14 (1H, s, H-5''), 7.97 (2H, d, *J* 7.5 Hz, Ar), 7.86 (2H, d, *J* 8.0 Hz, Ar), 6.73 (1H, s, H-4), 5.93 (1H, d, $J_{1''',2'''}$ 9.3 Hz, H-1'''), 5.64 (1H, pseudo t, $J_{2''',3'''}$ 9.7 Hz, H-2'''), 5.59 (1H, dd, $J_{4''',5'''}$ 0.4 Hz, H-4'''), 5.56 (1H, dd, $J_{4',5'}$ 0.4 Hz, H-4'), 5.48 (1H, pseudo t, $J_{2',3'}$ 10.1 Hz, H-2'), 5.30 (1H, dd, $J_{3''',4'''}$ 3.2 Hz, H-3'''), 5.22 (1H, dd, $J_{3',4'}$ 3.0 Hz, H-3'), 4.70 (1H, d, $J_{1',2'}$ 9.9 Hz, H-1'), 4.29 (1H, ddd, $J_{5''',6a'''}$ 5.8, $J_{5''',6b'''}$ 7.0 Hz, H-5'''), 4.23 (1H, dd, $J_{6a''',6b'''}$ 11.7 Hz, H-6_a'''), 4.21–4.16 (3H, m, H-6_a', H-6_b', H-6_b'''), 4.13 (1H, ddd, $J_{5',6a'}$ 6.2, $J_{5',6b'}$ 7.5 Hz, H-5'), 2.26, 2.22, 2.06, 2.03, 2.02, 1.99, 1.93 (24H, 8s, 8×CH_3). ^{13}C NMR (175 MHz, $CDCl_3$) δ (ppm) 170.6, 170.5, 170.3, 170.2, 170.1, 169.9, 169.7, 169.4 (8×CO), 170.1 (C-5), 161.6 (C-3), 147.6 (C-4''), 132.1–126.3 (Ar), 118.6 (C-5''), 98.2 (C-4), 86.5 (C-1'''), 75.1 (C-5'), 74.3 (C-5'''), 73.4 (C-1'), 71.8 (C-3'), 70.9 (C-3'''), 68.0 (C-2'''), 67.7 (C-2', C-4'), 67.0 (C-4'''), 61.8 (C-6'''), 61.3 (C-6'), 20.8, 20.7, 20.6, 20.4 (8×CH_3). HR-ESI-MS positive mode (*m/z*): calc. for $C_{39}H_{44}N_4O_{19}$ (872.26) $[M+H]^+$=873.2673, found: $[M+H]^+$=873.2671.

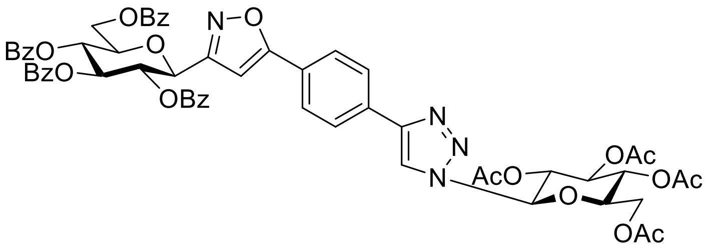

3-(2',3',4',6'-Tetra-*O*-benzoyl-β-D-glucopyranosyl)-5-(4-(1-(2''',3''',4''',6'''-tetra-*O*-acetyl-β-D-glucopyranosyl)-1''*H*-1'',2'',3''-triazol-4-yl))phenylisoxazole (14c)

Prepared from 2,3,4,6-tetra-*O*-acetyl-β-D-glucopyranosyl azide **10a** (1 equiv., 0.01 g, 0.03 mmol), 3-(2',3',4',6'-tetra-*O*-benzoyl-β-D-glucopyranosyl)-5-(4-ethynylphenyl)isoxazole **13b** (0.02 g, 0.03 mmol), $CuSO_4 \cdot 5\times H_2O$ (1.0 mg, 14 mol%) and Na-L-ascorbate (2.3 mg, 40 mol%) according to the General procedure III. Purified by column chromatography (1:2 EtOAc–hexane) to yield 28 mg (85%) of **14c** as a pale yellow amorphous product. R_f: 0.23 (1:1 EtOAc–hexane); $[\alpha]_D$ –30 (*c* 0.22, CH_2Cl_2). ^{1}H NMR (500 MHz, $CDCl_3$) δ (ppm) 8.09–8.02 (2H, m, Ar), 8.06 (1H, s, H-5''), 7.98–7.89 (4H, m, Ar), 7.89–7.78 (6H, m, Ar), 7.59–7.23 (12H, m, Ar), 6.75 (1H, s, H-4), 6.05 (1H, pseudo t, $J_{3',4'}$ 9.6 Hz, H-3'), 5.94 (1H, d, $J_{1''',2'''}$ 9.2 Hz, H-1'''), 5.83 (1H, pseudo t, $J_{4',5'}$ 9.8 Hz, H-4'), 5.82 (1H, pseudo t, $J_{2',3'}$ 9.5 Hz, H-2'), 5.51 (1H, pseudo t, $J_{2''',3'''}$ 9.3 Hz, H-2'''), 5.45 (1H, pseudo t, $J_{3''',4'''}$ 9.5 Hz, H-3'''), 5.27 (1H, pseudo t, $J_{4''',5'''}$ 10.1 Hz, H-4'''), 5.10 (1H, d, $J_{1',2'}$ 10.0 Hz, H-1'), 4.70 (1H, dd, $J_{6a',6b'}$ 12.4 Hz, H-6_a'), 4.53 (1H, dd, H-6_b'), 4.35 (1H, ddd, $J_{5',6a'}$ 2.8, $J_{5',6b'}$ 5.2 Hz, H-5'), 4.35 (1H, dd, $J_{6a''',6b'''}$ 12.7 Hz, H-6_a'''), 4.17 (1H, dd, H-6_b'''), 4.04 (1H, ddd, $J_{5''',6a'''}$ 5.2, $J_{5''',6b'''}$ 1.8 Hz, H-5'''), 2.10, 2.08, 2.05, 1.90 (12H, 4s, $4\times CH_3$). ^{13}C NMR (125 MHz, $CDCl_3$) δ (ppm) 170.5, 170.2, 169.5, 169.2, 166.3, 165.9, 165.4, 165.2 (8×CO), 170.2 (C-5), 161.3 (C-3), 147.7 (C-4''), 134.0–126.1 (Ar), 118.4 (C-5''), 98.1 (C-4), 86.0 (C-1'''), 76.9 (C-5'), 75.4 (C-5'''), 74.3 (C-3'), 73.5 (C-1'), 72.8 (C-3'''), 71.3 (C-2'), 70.4 (C-2'''), 69.6 (C-4'), 67.9 (C-4'''), 63.1 (C-6'), 61.7 (C-6'''), 20.9, 20.8, 20.7 ($4\times CH_3$). HR-ESI-MS positive mode (*m/z*): calc. for $C_{59}H_{52}N_4O_{19}$ (1120.32) $[M+H]^+$=1121.3299, found: $[M+H]^+$=1121.3302.

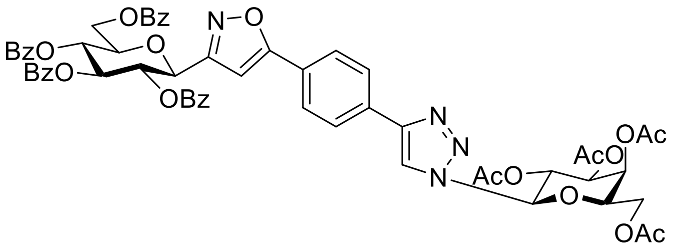

3-(2',3',4',6'-Tetra-*O*-benzoyl-β-D-glucopyranosyl)-5-(4-(1-(2''',3''',4''',6'''-tetra-*O*-acetyl-β-D-galactopyranosyl)-1''*H*-1'',2'',3''-triazol-4-yl))phenylisoxazole (14d)

Prepared from 2,3,4,6-tetra-*O*-acetyl-β-D-galactopyranosyl azide **10b** (1 equiv., 0.04 g, 0.10 mmol), 3-(2',3',4',6'-tetra-*O*-benzoyl-β-D-glucopyranosyl)-5-(4-ethynylphenyl)isoxazole **13b** (0.08 g, 0.10 mmol), $CuSO_4 \cdot 5\times H_2O$ (15.6 mg, 62 mol%) and Na-L-ascorbate (22.6 mg, 114 mol%) according to the General procedure III. Purified by column chromatography (from 1:2 to 2:1 EtOAc–hexane) to yield 45 mg (43%) of **14d** as a pale yellow amorphous product. R_f: 0.30 (1:1 EtOAc–hexane); $[\alpha]_D$ –96 (*c* 0.25, CH_2Cl_2). ^{1}H NMR (700 MHz, $CDCl_3$) δ (ppm) 8.11 (1H, s, H-5''), 8.07–8.03 (2H, m, Ar), 7.96–7.91 (4H, m, Ar), 7.88–7.77 (6H, m, Ar), 7.59–7.27 (12H, m, Ar), 6.76 (1H, s, H-4), 6.05 (1H, pseudo t, $J_{3',4'}$ 9.6 Hz, H-3'), 5.90 (1H, d, $J_{1''',2'''}$ 9.3 Hz, H-1'''), 5.83 (1H, pseudo t, $J_{4',5'}$ 9.8 Hz, H-4'), 5.82 (1H, pseudo t, $J_{2',3'}$ 9.8 Hz, H-2'), 5.63 (1H, pseudo t, $J_{2''',3'''}$ 10.3 Hz, H-2'''), 5.58 (1H, dd, $J_{4''',5'''}$ 0.8 Hz, H-4'''), 5.28 (1H, dd, $J_{3''',4'''}$ 3.3 Hz, H-3'''), 5.10 (1H, d, $J_{1',2'}$ 9.9 Hz, H-1'), 4.70 (1H, dd, $J_{6a',6b'}$ 12.4 Hz, H-6_a'), 4.53 (1H, dd, H-6_b'), 4.35 (1H, ddd, $J_{5',6a'}$ 2.8, $J_{5',6b'}$ 5.2 Hz, H-5'), 4.26 (1H, ddd, $J_{5''',6a'''}$ 5.8, $J_{5''',6b'''}$ 7.0 Hz, H-5'''),4.22 (1H, dd, $J_{6a''',6b'''}$ 11.6 Hz, H-6_a'''), 4.18 (1H, dd, H-6_b'''), 2.25, 2.05, 2.03, 1.91 (12H, 4s, 4×CH_3). ^{13}C NMR (175 MHz, $CDCl_3$) δ (ppm) 170.5, 170.2, 170.0, 169.4, 166.3, 165.9, 165.4, 165.2 (8×CO), 170.1 (C-5), 161.3 (C-3), 147.6 (C-4''), 133.8–126.3 (Ar), 118.6 (C-5''), 98.1 (C-4), 86.6 (C-1'''), 77.0 (C-5'), 74.3 (C-5'''), 74.3 (C-3'), 73.5 (C-1'), 71.3 (C-2'), 70.9 (C-3'''), 69.6 (C-4'), 68.0 (C-2'''), 67.0 (C-4'''), 63.3 (C-6'), 61.4 (C-6'''), 20.8, 20.6, 20.4 (4×CH_3). HR-ESI-MS positive mode (*m/z*): calc. for $C_{59}H_{52}N_4O_{19}$ (1120.32) $[M+H]^+$=1121.3299, found: $[M+H]^+$=1121.33297.

General procedure IV for the removal of *O*-acetyl protecting groups[9] 15–19

A (2’,3’,4’,6’-tetra-*O*-acyl-β-D-glycopyranosyl)isoxazoline **3**, **5**, and -isoxazole **9**, **14** and -triazole **12** derivatives (100 mg) were dissolved in a mixture of dry MeOH (5 mL) and dry chloroform (3 mL) then a solution of NaOMe (1 M in MeOH) was added to the solution in a catalytic amount. The reaction mixture was stirred at room temperature. When the reaction was complete (TLC, 7:3 $CHCl_3$–MeOH) (1–3 h) the solution was neutralized with a cation exchange resin Amberlyst 15 (H^+ form). The resin was filtered off with suction, and the filtrate was evaporated under reduced pressure. The crude product was purified by silica gel column chromatography with eluents indicated for the particular compounds to give (β-D-glycopyranosyl)isoxazoline **15**, **16**, -isoxazole **17**, **19** and -triazole **18** derivatives.

Characterization of (β-D-glycopyranosyl)isoxazoline 15, 16, -isoxazole 17, 19 and -triazole 18 derivatives

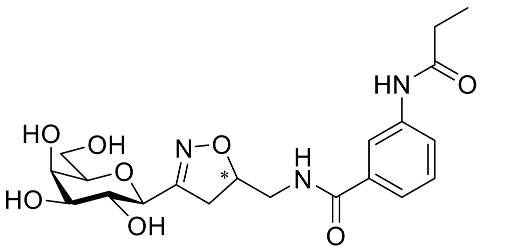

***N*-((3-(β-D-Galactopyranosyl)isoxazolin-5-yl)methyl)-3-(propionamido)benzamides (15a)**

Prepared from isoxazole **3a** (0.05 g, 0.08 mmol) according to the General procedure IV. Purified by column chromatography (7:3 $CHCl_3$–MeOH) to yield 29 mg (81%) of **15a** (diastereomeric ratio: 1.3:1) as a colourless amorphous product. R_f: 0.30 (7:3 $CHCl_3$–MeOH). **15a-I** ^{1}H NMR (700 MHz, CD_3OD) δ (ppm) 8.00–7.95 (1H, m, Ar), 7.73 (1H, t, *J* 8.2 Hz, Ar), 7.52 (1H, t, *J* 7.1 Hz, Ar), 7.40 (1H, t, *J* 7.9 Hz, Ar), 4.89–4.77 (1H, m, H-5), 4.03 (1H, d, $J_{1',2'}$ 9.7 Hz, H-1’), 3.91 (1H, dd, $J_{3',4'}$ 3.1, $J_{4',5'}$ 0.4 Hz, H-4’), 3.73 (1H, pseudo t, $J_{2',3'}$ 10.2 Hz, H-2’), 3.69–3.56 (3H, m, H-5’, H-6$_a$’, H-6$_b$’), 3.55–3.44 (3H, m, CH_2NHCO, H-3’), 3.26 (1H, dd, $J_{4a,4b}$ 17.5, $J_{4a,5}$

10.6 Hz, H-4_a), 2.97 (1H, dd, $J_{4b,5}$ 7.1 Hz, H-4_b), 2.41 (2H, q, *J* 7.6 Hz, NHCOC$H_2$$CH_3$), 1.21 (3H, t, *J* 7.6 Hz, NHCOCH_2CH_3). [13]C NMR (175 MHz, CD_3OD) δ (ppm) 175.6 (NH*C*OCH_2CH_3), 170.6 (CH_2NH*C*O), 159.3 (C-3), 140.3–120.1 (Ar), 80.9 (C-5'), 80.2 (C-5), 77.0 (C-1'), 76.0 (C-3'), 70.7 (C-4'), 69.8 (C-2'), 62.8 (C-6'), 44.0 (*C*H_2NHCO), 37.8 (C-4), 31.0 (NHCO*C*H_2CH_3), 10.2 (NHCOCH_2*C*H_3). **15a-II** [1]H NMR (700 MHz, CD_3OD) δ (ppm) 8.00–7.95 (1H, m, Ar), 7.73 (1H, t, *J* 8.2 Hz, Ar), 7.52 (1H, t, *J* 7.1 Hz, Ar), 7.40 (1H, t, *J* 7.9 Hz, Ar), 4.89–4.77 (1H, m, H-5), 4.03 (1H, d, $J_{1',2'}$ 9.7 Hz, H-1'), 3.91 (1H, dd, $J_{3',4'}$ 3.1, $J_{4',5'}$ 0.4 Hz, H-4'), 3.76 (1H, pseudo t, $J_{2',3'}$ 9.6 Hz, H-2'), 3.69–3.56 (3H, m, H-5', H-6_a', H-6_b'), 3.55–3.44 (3H, m, CH_2NHCO, H-3'), 3.22 (1H, dd, $J_{4a,4b}$ 17.6, $J_{4a,5}$ 10.6 Hz, H-4_a), 3.03 (1H, dd, $J_{4b,5}$ 6.5 Hz, H-4_b), 2.41 (2H, q, *J* 7.6 Hz, NHCOC$H_2$$CH_3$), 1.21 (3H, t, *J* 7.6 Hz, NHCOCH_2CH_3). ^{13}C NMR (175 MHz, CD_3OD) δ (ppm) 175.5 (NH*C*OCH_2CH_3), 170.5 (CH_2NH*C*O), 159.1 (C-3), 140.3–120.1 (Ar), 80.8 (C-5'), 80.1 (C-5), 77.1 (C-1'), 75.8 (C-3'), 70.7 (C-4'), 69.6 (C-2'), 62.7 (C-6'), 44.2 (*C*H_2NHCO), 37.5 (C-4), 31.0 (NHCO*C*H_2CH_3), 10.2 (NHCOCH_2*C*H_3). HR-ESI-MS positive mode (*m*/*z*): calcd. for $C_{20}H_{27}N_3O_8$ (437.18) $[M+Na]^+$=460.1690, found: $[M+Na]^+$=460.1692.

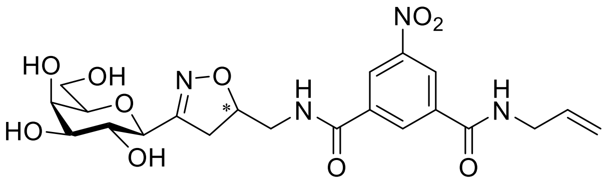

***N*[1]-((3-(β-D-Galactopyranosyl)isoxazolin-5-yl)methyl)-*N*[3](prop-2-en-1-yl)-5-nitroisophthalamides (15b)**

Prepared from isoxazolines **3b** (0.04 g, 0.07 mmol) according to the General procedure IV. Purified by column chromatography (from 10:1 to 3:1 $CHCl_3$–MeOH) to yield 16 mg (51%) of **15b** (diastereomeric ratio: 1.3:1) as a colourless yellow product. R_f: 0.34 (3:1 $CHCl_3$–MeOH). **15b-I** ^{1}H NMR (500 MHz, CD_3OD) δ (ppm) 8.86–8.79 (2H, m, Ar), 8.71–8.65 (1H, m, Ar),

5.97 (1H, dddd, $J_{CH,CHa}$ 17.2, $J_{CH,CHb}$ 10.2, $J_{CHc,CH}$ 5.5, $J_{CHd,CH}$ 5.4 Hz, CONHCH$_2$C*H*CH$_2$), 5.32–5.24 (1H, m, CONHCH_{2c}CHCH$_2$), 5.20–5.15 (1H, m, CONHCH_{2d}CHCH$_2$), 4.94–4.80 (1H, m, H-5), 4.08–4.00 (3H, m, H-1', CONHCH$_2$CHCH_2), 3.90 (1H, dd, $J_{4',5'}$ 0.7 Hz, H-4'), 3.78–3.53 (6H, m, H-2', H-5', H-6$_a$', H-6$_b$', CH_2NHCO), 3.51 (1H, dd, $J_{2',3'}$ 9.4, $J_{3',4'}$ 3.2 Hz, H-3'), 3.28 (1H, dd, $J_{4a,5}$ 7.8, H-4$_a$), 2.99 (1H, dd, $J_{4a,4b}$ 17.6, $J_{4b,5}$ 7.0 Hz, H-4$_b$). ^{13}C NMR (125 MHz, CD$_3$OD) δ (ppm) 167.6 (CH$_2$NH*C*O), 166.8 (*C*ONHCH$_2$CHCH$_2$), 159.2 (C-3), 149.9–125.6 (Ar), 135.1 (CONHCH$_2$*C*HCH$_2$), 116.7 (CONH*C*H$_2$CHCH$_2$), 80.9 (C-5'), 80.1 (C-5), 77.0 (C-1'), 76.0 (C-3'), 70.7 (C-4'), 69.8 (C-2'), 62.8 (C-6'), 44.2 (*C*H$_2$NHCO), 43.6 (CONHCH$_2$CH*C*H$_2$), 37.7 (C-4). **15b-II** ^{1}H NMR (500 MHz, CD$_3$OD) δ (ppm) 8.86–8.79 (2H, m, Ar), 8.71–8.65 (1H, m, Ar), 5.97 (1H, dddd, $J_{CH,CHa}$ 17.2, $J_{CH,CHb}$ 10.2, $J_{CHc,CH}$ 5.5, $J_{CHd,CH}$ 5.4 Hz, CONHCH$_2$C*H*CH$_2$), 5.32–5.24 (1H, m, CONHCH_{2c}CHCH$_2$), 5.20–5.15 (1H, m, CONHCH_{2d}CHCH$_2$), 4.94–4.80 (1H, m, H-5), 4.08–4.00 (3H, m, H-1', CONHCH$_2$CHCH_2), 3.90 (1H, dd, $J_{4',5'}$ 0.7 Hz, H-4'), 3.78–3.53 (6H, m, H-2', H-5', H-6$_a$', H-6$_b$', CH_2NHCO), 3.51 (1H, dd, $J_{2',3'}$ 9.4, $J_{3',4'}$ 3.2 Hz, H-3'), 3.25 (1H, dd, $J_{4a,5}$ 10.6, H-4$_a$), 3.05 (1H, dd, $J_{4a,4b}$ 17.5, $J_{4b,5}$ 6.3 Hz, H-4$_b$). ^{13}C NMR (125 MHz, CD$_3$OD) δ (ppm) 167.5 (CH$_2$NH*C*O), 166.8 (*C*ONHCH$_2$CHCH$_2$), 159.2 (C-3), 149.9–125.6 (Ar), 135.1 (CONHCH$_2$*C*HCH$_2$), 116.7 (CONH*C*H$_2$CHCH$_2$), 80.8 (C-5'), 80.0 (C-5), 77.1 (C-1'), 75.8 (C-3'), 70.6 (C-4'), 69.6 (C-2'), 62.6 (C-6'), 44.5 (*C*H$_2$NHCO), 43.6 (CONHCH$_2$CH*C*H$_2$), 37.5 (C-4). HR-ESI-MS positive mode (*m*/*z*): calcd. for $C_{21}H_{26}N_4O_{10}$ (494.16) [M+H]$^+$=495.1722, found: [M+H]$^+$=495.1723.

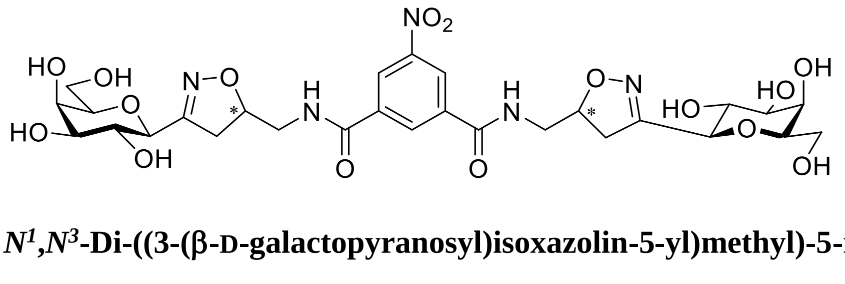

***N*^{1},*N*^{3}-Di-((3-(β-D-galactopyranosyl)isoxazolin-5-yl)methyl)-5-nitroisophthalamides (16b)**

Prepared from isoxazolines **5b** (0.06 g, 0.06 mmol) according to the General procedure IV. Purified by column chromatography (1:1 $CHCl_3$–MeOH) to yield 24 mg (60%) of **16b** (diastereomeric ratio: 2:1:1) as a yellow amorphous product. R_f: 0.56 (1:2 $CHCl_3$–MeOH). **16b-I** ^{1}H NMR (700 MHz, CD_3OD) δ (ppm) 8.83–8.80 (2H, m, Ar), 8.66–8.64 (1H, m, Ar), 4.94–4.82 (2H, m, 2×H-5), 4.05 (1H, d, $J_{1',2'}$ 9.7 Hz, 2×H-1'), 3.91–3.88 (1H, m, 2×H-4'), 3.78–3.54 (12H, m, 2×H-2', 2×H-5', 2×H-6_a', 2×H-6_b', 2×CH_2), 3.54–3.50 (2H, m, 2×H-3'), 3.29–3.21 (1H, m, H-4_a), 3.09–2.96 (1H, m, H-4_b). ^{13}C NMR (125 MHz, CD_3OD) δ (ppm) 167.7 (2×*C*ONHCH_2), 159.2 (2×C-3), 149.8–125.8 (Ar), 80.9 (2×C-5'), 80.1 (2×C-5), 76.9 (2×C-1'), 76.0 (2×C-3'), 70.7 (2×C-4'), 69.9 (2×C-2'), 62.8 (2×C-6'), 44.5 (2×*C*H_2NHCO), 37.6 (2×C-4). **16b-II** ^{1}H NMR (700 MHz, CD_3OD) δ (ppm) 8.83–8.80 (2H, m, Ar), 8.66–8.64 (1H, m, Ar), 4.94–4.82 (2H, m, 2×H-5), 4.04 (1H, d, $J_{1',2'}$ 9.5 Hz, 2×H-1'), 3.91–3.88 (1H, m, 2×H-4'), 3.78–3.54 (12H, m, 2×H-2', 2×H-5', 2×H-6_a', 2×H-6_b', 2×CH_2), 3.54–3.50 (2H, m, 2×H-3'), 3.29–3.21 (1H, m, H-4_a), 3.09–2.96 (1H, m, H-4_b). ^{13}C NMR (125 MHz, CD_3OD) δ (ppm) 167.6 (2×*C*ONHCH_2), 159.2 (2×C-3), 149.8–125.8 (Ar), 80.7 (2×C-5'), 80.1 (2×C-5), 77.0 (2×C-1'), 75.8 (2×C-3'), 70.7 (2×C-4'), 69.6 (2×C-2'), 62.6 (2×C-6'), 44.2 (2×*C*H_2NHCO), 37.6 (2×C-4). **16b-III** ^{1}H NMR (700 MHz, CD_3OD) δ (ppm) 8.83–8.80 (2H, m, Ar), 8.66–8.64 (1H, m, Ar), 4.94–4.82 (2H, m, 2×H-5), 4.04 (1H, d, $J_{1',2'}$ 9.5 Hz, 2×H-1'), 3.91–3.88 (1H, m, 2×H-4'), 3.78–3.54 (12H, m, 2×H-2', 2×H-5', 2×H-6_a', 2×H-6_b', 2×CH_2), 3.54–3.50 (2H, m, 2×H-3'), 3.29–3.21 (1H, m, H-4_a), 3.09–2.96 (1H, m, H-4_b). ^{13}C NMR (125 MHz, CD_3OD) δ (ppm) 167.7 (2×*C*ONHCH_2), 159.2 (2×C-3), 149.8–125.8 (Ar), 80.7 (2×C-5'), 80.0 (2×C-5), 77.0 (2×C-1'), 75.9 (2×C-3'), 70.6 (2×C-4'), 69.6 (2×C-2'), 62.8 (2×C-6'), 44.1 (2×*C*H_2NHCO), 37.6 (2×C-4). HR-ESI-MS positive mode (*m*/*z*): calcd. for $C_{28}H_{37}N_5O_{16}$ (699.22) $[M+H]^+$=700.2308, found: $[M+H]^+$=700.2309.

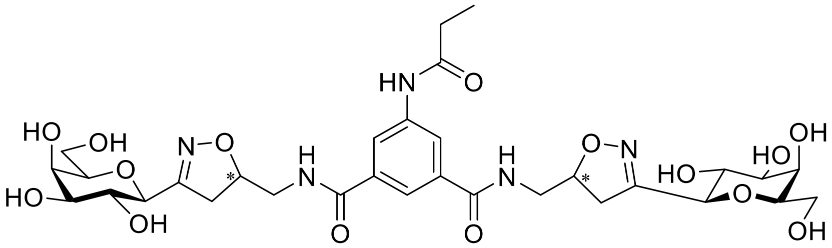

N^1,N^3-Di-((3-(β-D-galactopyranosyl)isoxazolin-5-yl)methyl)-5-(propionamido)isophthalamides (16c)

Prepared from isoxazolines **5c** (0.04 g, 0.03 mmol) according to the General procedure IV. Purified by column chromatography (2:1 $CHCl_3$–MeOH) to yield 24 mg (93%) of **16c** (diastereomeric ratio: 1.3:1) as a yellow amorphous product. R_f: 0.19 (7:3 $CHCl_3$–MeOH). **16c-I** ^{1}H NMR (700 MHz, CD_3OD) δ (ppm) 8.25–8.10 (2H, m, Ar), 7.98–7.91 (1H, m, Ar), 4.93–4.79 (2H, m, 2×H-5), 4.03 (1H, d, $J_{1',2'}$ 9.6 Hz, 2×H-1'), 3.91 (1H, dd, $J_{3',4'}$ 2.6, $J_{4',5'}$ 0.5 Hz, 2×H-4'), 3.78–3.62 (6H, m, 2×H-2', 2×H-6a', 2×H-6b'), 3.62–3.46 (8H, m, 2×H-3', 2×H-5', 2×CH2), 3.27 (1H, dd, $J_{4a,5}$ 10.5 Hz, H-4_a), 2.98 (1H, dd, $J_{4a,4b}$ 17.6, $J_{4b,5}$ 7.1 Hz, H-4_b), 2.44 (2H, q, *J* 7.6 Hz, NHCOC*H*$_2$CH$_3$), 1.22 (3H, t, *J* 7.6 Hz, NHCOCH$_2$C*H*$_3$). ^{13}C NMR (175 MHz, CD_3OD) δ (ppm) 175.7 (NH*C*OCH$_2$CH$_3$), 169.9 (2×*C*ONHCH$_2$), 159.2 (2×C-3), 140.9–116.3 (Ar), 80.8 (2×C-5'), 80.2 (2×C-5), 77.0 (2×C-1'), 76.0 (2×C-3'), 70.8 (2×C-4'), 69.8 (2×C-2'), 62.7 (2×C-6'), 44.0 (2×*C*H$_2$NHCO), 37.6 (2×C-4), 31.0 (NHCO*C*H$_2$CH$_3$), 10.1 (NHCOCH$_2$*C*H$_3$). **16c-II** ^{1}H NMR (700 MHz, CD_3OD) δ (ppm) 8.25–8.10 (2H, m, Ar), 7.98–7.91 (1H, m, Ar), 4.93–4.79 (2H, m, 2×H-5), 4.04 (1H, d, $J_{1',2'}$ 9.6 Hz, 2×H-1'), 3.91 (1H, dd, $J_{3',4'}$ 2.6, $J_{4',5'}$ 0.5 Hz, 2×H-4'), 3.78–3.62 (6H, m, 2×H-2', 2×H-6_a', 2×H-6_b'), 3.62–3.46 (8H, m, 2×H-3', 2×H-5', 2×CH$_2$), 3.23 (1H, dd, $J_{4a,5}$ 10.6 Hz, H-4_a), 3.03 (1H, dd, $J_{4a,4b}$ 17.5, $J_{4b,5}$ 6.5 Hz, H-4_b), 2.44 (2H, q, *J* 7.6 Hz, NHCOC*H*$_2$CH$_3$), 1.22 (3H, t, *J* 7.6 Hz, NHCOCH$_2$C*H*$_3$). ^{13}C NMR (175 MHz, CD_3OD) δ (ppm) 175.7 (NH*C*OCH$_2$CH$_3$), 169.7 (2×*C*ONHCH$_2$), 159.2 (2×C-3), 140.9–116.3 (Ar), 80.9 (2×C-5'), 80.1 (2×C-5), 77.1 (2×C-1'), 75.9 (2×C-3'), 70.7 (2×C-4'), 69.6 (2×C-2'), 62.8 (2×C-6'), 44.3 (2×*C*H$_2$NHCO), 37.6 (2×C-4), 31.0

(NHCOCH_2CH$_3$), 10.1 (NHCOCH$_2$$CH_3$). HR-ESI-MS positive mode (*m*/*z*): calcd. for $C_{31}H_{42}N_5O_3$ (725.28) $[M+H]^+$=726.2828, found: $[M+H]^+$=726.2823.

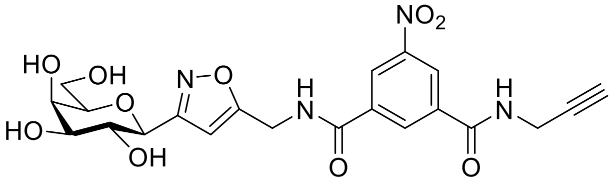

N^1-((3-(β-D-Galactopyranosyl)isoxazol-5-yl)methyl)-N^3-(prop-2-yn-1-yl)-5-nitroisophthalamide (17a)

Prepared from isoxazole **9a** (0.04 g, 0.07 mmol) according to the General procedure IV. Purified by column chromatography (3:1 $CHCl_3$–MeOH) to yield 14 mg (51%) of **17a** as a yellow amorphous product. R_f: 0.32 (3:1 $CHCl_3$–MeOH); $[\alpha]_D$ +0.3 (*c* 0.11, CH_2Cl_2). ^{1}H NMR (500 MHz, CD_3OD) δ (ppm) 8.87 (1H, s, Ar), 8.85 (1H, s, Ar), 8.73 (1H, s, Ar), 6.56 (1H, s, H-4), 4.74 (2H, s, CH_2NHCO), 4.28 (1H, d, $J_{1',2'}$ 9.7 Hz, H-1'), 4.23–4.17 (2H, m, CONHCH_2CCH), 3.94 (1H, dd, $J_{4',5'}$ 0.6 Hz, H-4'), 3.78 (1H, pseudo t, $J_{2',3'}$ 9.5 Hz, H-2'), 3.74 (1H, dd, $J_{5',6a'}$ 6.6, $J_{6a',6b'}$ 11.1 Hz, H-6$_a$'), 3.68 (1H, dd, $J_{5',6b'}$ 4.9 Hz, H-6$_b$'), 3.67–3.62 (1H, m, H-5'), 3.57 (1H, dd, $J_{3',4'}$ 3.3 Hz, H-3'), 2.66 (1H, pseudo t, $J_{CHc,CH}$ 2.1, $J_{CHd,CH}$ 2.3 Hz, CONHCH$_2$CC*H*). ^{13}C NMR (125 MHz, CD_3OD) δ (ppm) 170.8 (C-5), 166.9 (CH$_2$NH*C*O), 166.5 (*C*ONHCH$_2$CCH), 164.5 (C-3), 150.2–125.4 (Ar), 102.2 (C-4), 81.1 (C-5'), 80.3 (CONHCH$_2$*C*CH), 76.4 (C-1'), 76.0 (C-3'), 72.5 (CONHCH$_2$C*C*H), 71.4 (C-2'), 70.8 (C-4'), 62.8 (C-6'), 36.8 (*C*H$_2$NHCO), 30.2 (CONH*C*H$_2$CCH). HR-ESI-MS positive mode (*m*/*z*): calcd. for $C_{21}H_{22}N_4O_{10}$ (490.13) $[M+H]^+$=491.1409, found: $[M+H]^+$=491.1403.

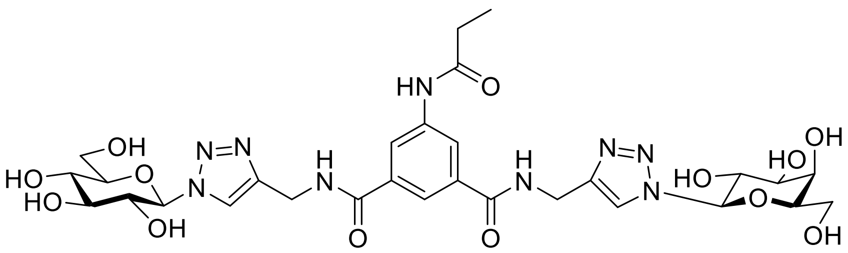

***N*[1]-((1-(β-D-Glucopyranosyl)-1*H*-1,2,3-triazol-4-yl)methyl)-*N*[3]-((1-(β-D-galactopyranosyl)-1''*H*-1'',2'',3''-triazol-4-yl)methyl)-5-(propionamido)isophthalamide (18b)**

Prepared from triazole **12b** (0.01 g, 0.01 mmol) according to the General procedure IV to yield 4 mg (74%) of **18b** as a yellow amorphous product. R_f: 0.09 (1:1 $CHCl_3$–MeOH); $[\alpha]_D$ –13 (*c* 0.01, CH_2Cl_2). ^{1}H NMR (700 MHz, CD_3OD) δ (ppm) 8.23–8.16 (3H, m, Ar, H-5 or H-5''), 8.15 (1H, s, H-5 or H-5''), 8.00 (1H, s, Ar), 5.59 (1H, d, $J_{1',2'}$ 9.2 Hz, H-1'), 5.56 (1H, d, $J_{1''',2'''}$ 9.2 Hz, H-1'''), 4.71–4.63 (4H, m, 2×CH_2), 4.12 (1H, pseudo t, $J_{2''',3'''}$ 9.4 Hz, H-2'''), 3.97 (1H, dd, $J_{4''',5'''}$ 0.5 Hz, H-4'''), 3.88 (1H, pseudo t, $J_{2',3'}$ 9.1 Hz, H-2'), 3.86 (2H, dd, $J_{6a',6b'}$ 12.4 Hz, H-6_a'), 3.83 (1H, ddd, $J_{5''',6a'''}$ 6.8, $J_{5''',6b'''}$ 5.3 Hz, H-5'''), 3.75 (1H, dd, $J_{6a''',6b'''}$ 11.6 Hz, H-6_a'''), 3.72 (1H, dd, H-6_b'''), 3.70 (1H, dd, H-6_b'), 3.69 (1H, dd, $J_{3''',4'''}$ 3.6 Hz, H-3'''), 3.57 (1H, ddd, $J_{5',6a'}$ 2.3, $J_{5',6b'}$ 5.3 Hz, H-5'), 3.55 (1H, pseudo t, $J_{3',4'}$ 9.0 Hz, H-3'), 3.49 (1H, pseudo t, $J_{4',5'}$ 9.5 Hz, H-4'), 2.42 (2H, q, *J* 7.5 Hz, NHCOC*H*$_2$CH$_3$), 1.21 (3H, t, *J* 7.6 Hz, NHCOCH$_2$C*H*$_3$). ^{13}C NMR (175 MHz, CD_3OD) δ (ppm) 175.2 (NH*C*OCH$_2$CH$_3$), 169.0, 168.8 (2×*C*ONHCH$_2$), 146.2, 145.9 (C-4, C-4''), 141.5–121.7 (Ar), 123.0, 122.4 (C-5, C-5''), 90.3 (C-1'''), 89.7 (C-1'), 81.1 (C-5'), 80.0 (C-5'''), 78.5 (C-3'), 75.3 (C-3'''), 74.0 (C-2'), 71.5 (C-2'''), 70.9 (C-4'), 70.4 (C-4'''), 62.4 (C-6', C-6'''), 36.2 (2×*C*H$_2$NHCO), 30.8 (NHCO*C*H$_2$CH$_3$), 10.1 (NHCOCH$_2$*C*H$_3$). HR-ESI-MS negative mode (*m/z*): calcd. for calc. for $C_{29}H_{39}N_9O_{13}$ (721.27) $[M+HCOO]^-$=766.2649, found: $[M+HCOO]^-$=766.2645.

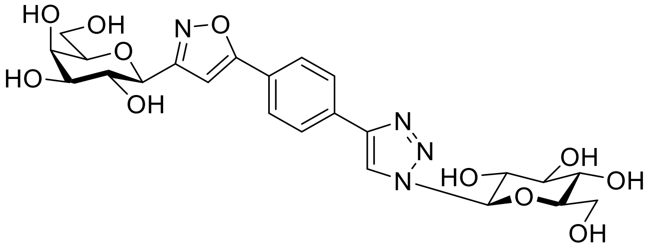

3-(β-D-Galactopyranosyl)-5-(4-(1-(β-D-glucopyranosyl)-1*H*-1,2,3-triazol-4-yl))phenyl-isoxazole (19a)

Prepared from isoxazole **14a** (0.04 g, 0.05 mmol) according to the General procedure IV. Purified by column chromatography (4:1 $CHCl_3$–MeOH) to yield 16 mg (58%) of **19a** as a yellow amorphous product. R_f: 0.41 (4:1 $CHCl_3$–MeOH); $[\alpha]_D$ +2 (*c* 0.08, MeOH). ^{1}H NMR (700 MHz, CD_3OD) δ (ppm) 8.68 (1H, s, H-5’’), 8.01 (2H, d, *J* 8.6 Hz, Ar), 7.93 (2H, d, *J* 8.6 Hz, Ar), 7.01 (1H, s, H-4), 5.68 (1H, d, $J_{1''',2'''}$ 9.2 Hz, H-1’’’), 4.36 (1H, d, $J_{1',2'}$ 9.7 Hz, H-1’), 3.99 (1H, dd, $J_{4',5'}$ 0.7 Hz, H-4’), 3.96 (1H, pseudo t, $J_{2''',3'''}$ 9.2 Hz, H-2’’’), 3.91 (1H, dd, $J_{5''',6a'''}$ 2.1, $J_{6a''',6b'''}$ 12.3 Hz, H-6_a’’’), 3.88 (1H, pseudo t, $J_{2',3'}$ 9.4 Hz, H-2’), 3.87–3.67 (3H, m, H-5’, H-6_b’, H-6_b’’’), 3.80 (1H, dd, $J_{5',6a'}$ 6.9, $J_{6a',6b'}$ 11.4 Hz, H-6_a’), 3.66–3.58 (1H, m, H-5’’’), 3.62 (1H, dd, $J_{3',4'}$ 3.1 Hz, H-3’), 3.60 (1H, pseudo t, $J_{3''',4'''}$ 9.1 Hz, H-3’’’), 3.54 (1H, pseudo t, $J_{4''',5'''}$ 9.5 Hz, H-4’’’). ^{13}C NMR (125 MHz, CD_3OD) δ (ppm) 170.7 (C-5), 165.2 (C-3), 147.9 (C-4’’), 133.6–126.8 (Ar), 122.2 (C-5’’), 100.1 (C-4), 89.8 (C-1’’’), 81.2 (C-5’’’), 81.1 (C-5’), 78.4 (C-3’’’), 76.5 (C-1’), 76.0 (C-3’), 74.1 (C-2’’’), 71.5 (C-2’), 70.9 (C-4’’’), 70.8 (C-4’), 62.9 (C-6’), 62.4 (C-6’’’). HR-ESI-MS positive mode (*m*/*z*): calcd. for $C_{23}H_{28}N_4O_{11}$ (536.18) $[M+Na]^+$=559.1647, found: $[M+Na]^+$=559.1645.

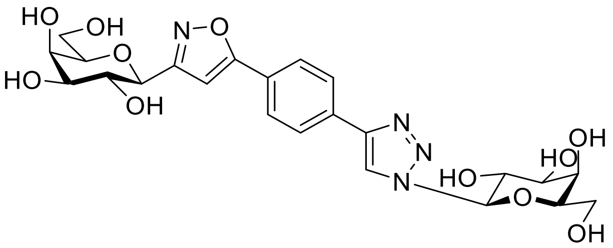

3-(β-D-Galactopyranosyl)-5-(4-(1-(β-D-galactopyranosyl)-1*H*-1,2,3-triazol-4-yl))phenyl-isoxazole (19b)

Prepared from isoxazole **14b** (0.03 g, 0.03 mmol) according to the General procedure IV to yield 6 mg (34%) of **19b** as a brown amorphous product. R_f: 0.18 (1:1 $CHCl_3$–MeOH); $[\alpha]_D$ –9 (*c* 0.03, MeOH). ^{1}H NMR (700 MHz, CD_3OD) δ (ppm) 8.72 (1H, s, H-5’’), 8.01 (2H, d, *J* 8.5 Hz, Ar), 7.94 (2H, d, *J* 8.5 Hz, Ar), 7.02 (1H, s, H-4), 5.65 (1H, d, $J_{1''',2'''}$ 9.2 Hz, H-1’’’), 4.36 (1H, d, $J_{1',2'}$ 9.7 Hz, H-1’), 4.21 (1H, pseudo t, $J_{2''',3'''}$ 9.2 Hz, H-2’’’), 4.02 (1H, dd, $J_{3''',4'''}$ 3.0, $J_{4''',5'''}$ 0.4 Hz, H-4’’’), 3.99 (1H, dd, $J_{4',5'}$ 0.6 Hz, H-4’), 3.91–3.86 (1H, m, H-5’’’), 3.88 (1H, pseudo t, $J_{2',3'}$ 9.4 Hz, H-2’), 3.83–3.68 (4H, m, H-3’’’, H-5’, H-6_a’, H-6_b’), 3.81 (1H, dd, $J_{5''',6a'''}$ 6.5, $J_{6a''',6b'''}$ 11.7 Hz, H-6_a’’’), 3.77 (1H, dd, $J_{5''',6b'''}$ 5.1 Hz, H-6_b’’’), 3.62 (1H, dd, $J_{3',4'}$ 3.4 Hz, H-3’). ^{13}C NMR (175 MHz, CD_3OD) δ (ppm) 170.6 (C-5), 165.2 (C-3), 147.9 (C-4’’), 134.7–127.1 (Ar), 121.8 (C-5’’), 100.1 (C-4), 90.6 (C-1’’’), 81.1 (C-5’), 80.1 (C-5’’’), 76.5 (C-1’), 76.1 (C-3’), 75.3 (C-3’’’), 71.6 (C-2’’’), 71.5 (C-2’), 70.8 (C-4’), 70.4 (C-4’’’), 62.9 (C-6’), 62.5 (C-6’’’). HR-ESI-MS negative mode (*m*/*z*): calcd. for $C_{23}H_{28}N_4O_{11}$ (536.18) $[M+HCOO]^-$=581.1737, found: $[M+HCOO]^-$=581.1735.

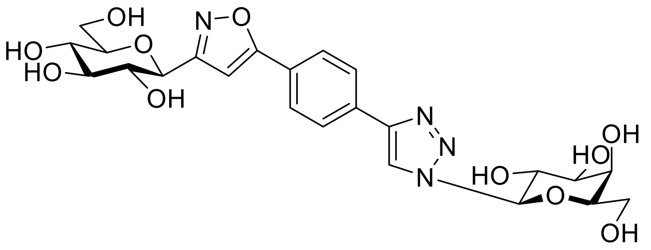

3-(β-D-Glucopyranosyl)-5-(4-(1-(β-D-galactopyranosyl)-1*H*-1,2,3-triazol-4-yl))phenyl-isoxazole (19d)

Prepared from isoxazole **14d** (0.04 g, 0.04 mmol) according to the General procedure IV to yield 5 mg (24%) of **19d** as a pale yellow amorphous product. R_f: 0.13 (1:1 $CHCl_3$–MeOH); $[\alpha]_D$ +27 (*c* 0.05, MeOH). ^{1}H NMR (700 MHz, CD_3OD) δ (ppm) 8.69 (1H, s, H-5''), 8.00 (2H, d, *J* 8.7 Hz, Ar), 7.94 (2H, d, *J* 8.6 Hz, Ar), 6.97 (1H, s, H-4), 5.64 (1H, d, $J_{1''',2'''}$ 9.2 Hz, H-1'''), 4.43 (1H, d, $J_{1',2'}$ 9.3 Hz, H-1'), 4.22 (1H, pseudo t, $J_{2''',3'''}$ 9.4 Hz, H-2'''), 4.02 (1H, dd, $J_{3''',4'''}$ 3.2, $J_{4''',5'''}$ 0.5 Hz, H-4'''), 3.93–3.87 (2H, m, H-5''', H-6_a'), 3.81 (1H, dd, $J_{5''',6a'''}$ 6.9, $J_{6a''',6b'''}$ 11.7 Hz, H-6_a'''), 3.80–3.69 (2H, m, H-3''', H-6_b'), 3.77 (1H, dd, $J_{5''',6b'''}$ 5.1 Hz, H-6_b'''), 3.55 (1H, pseudo t, $J_{2',3'}$ 9.0 Hz, H-2'), 3.51 (1H, pseudo t, $J_{3',4'}$ 8.5 Hz, H-3'), 3.48–3.43 (2H, m, H-4', H-5'). ^{13}C NMR (175 MHz, CD_3OD) δ (ppm) 170.7 (C-5), 165.1 (C-3), 148.0 (C-4''), 134.3–126.9 (Ar), 121.7 (C-5''), 100.1 (C-4), 90.6 (C-1'''), 82.4 (C-5'), 80.1 (C-5'''), 79.5 (C-3'), 76.1 (C-1'), 75.3 (C-3'''), 74.7 (C-2'), 71.6 (C-4'), 71.5 (C-2'''), 70.4 (C-4'''), 62.9 (C-6'), 62.5 (C-6'''). HR-ESI-MS negative mode (*m*/*z*): calcd. for $C_{23}H_{28}N_4O_{11}$ (536.18) $[M+HCOO]^-$=581.1737, found: $[M+HCOO]^-$=581.1734.

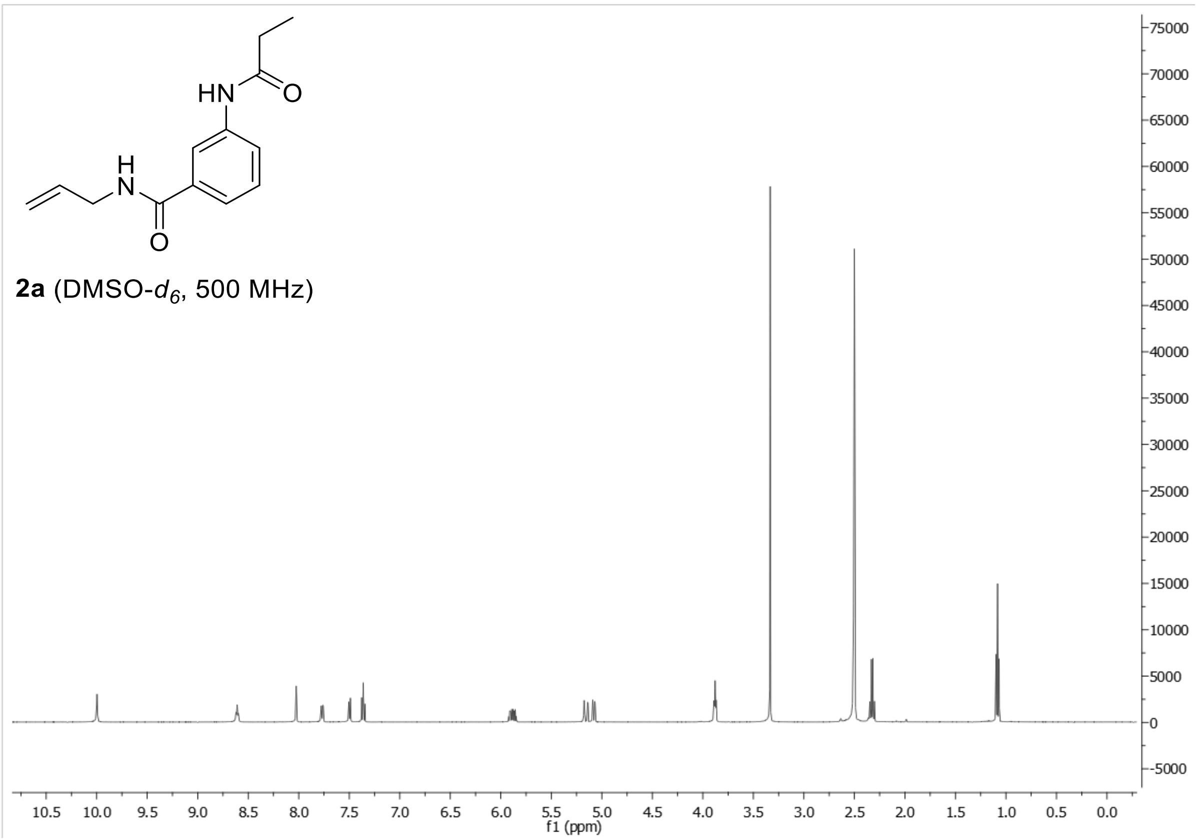

Figure S1. ^{1}H NMR spectrum of **2a**

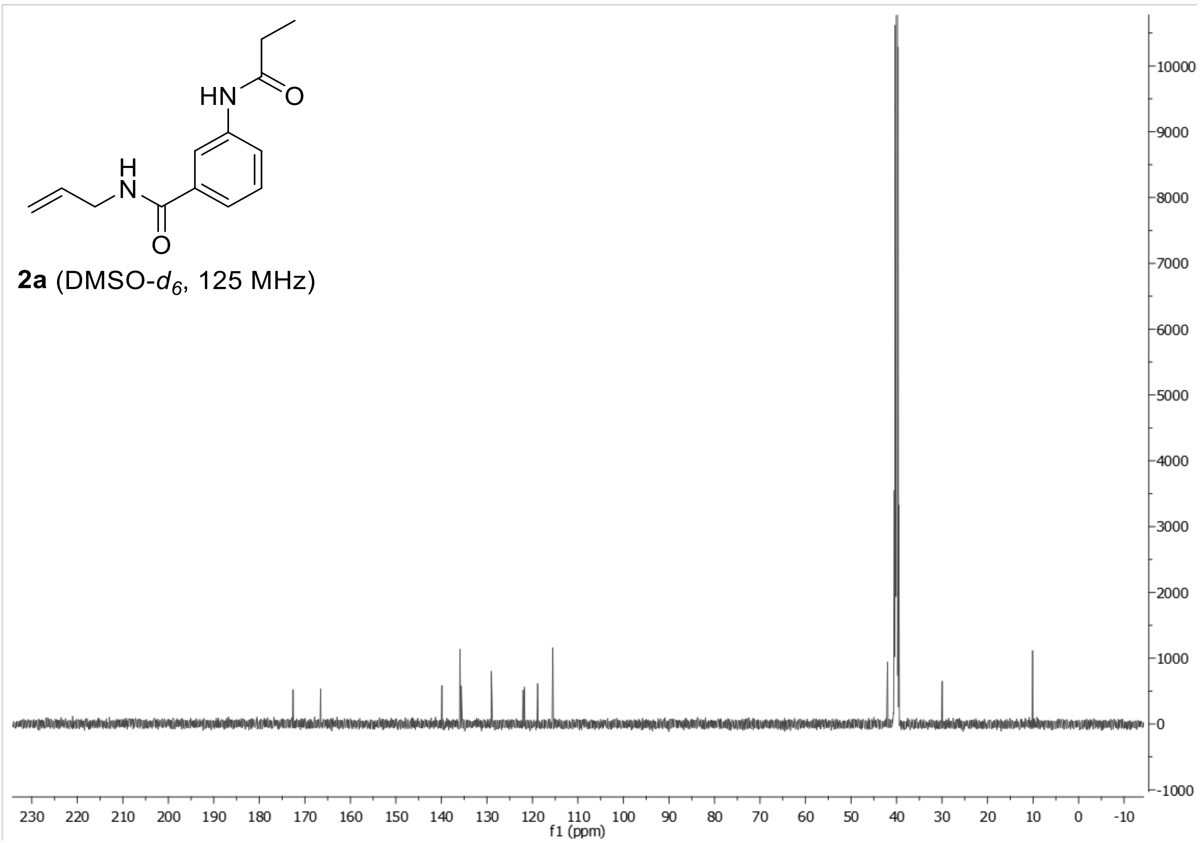

Figure S2. ^{13}C NMR spectrum of **2a**

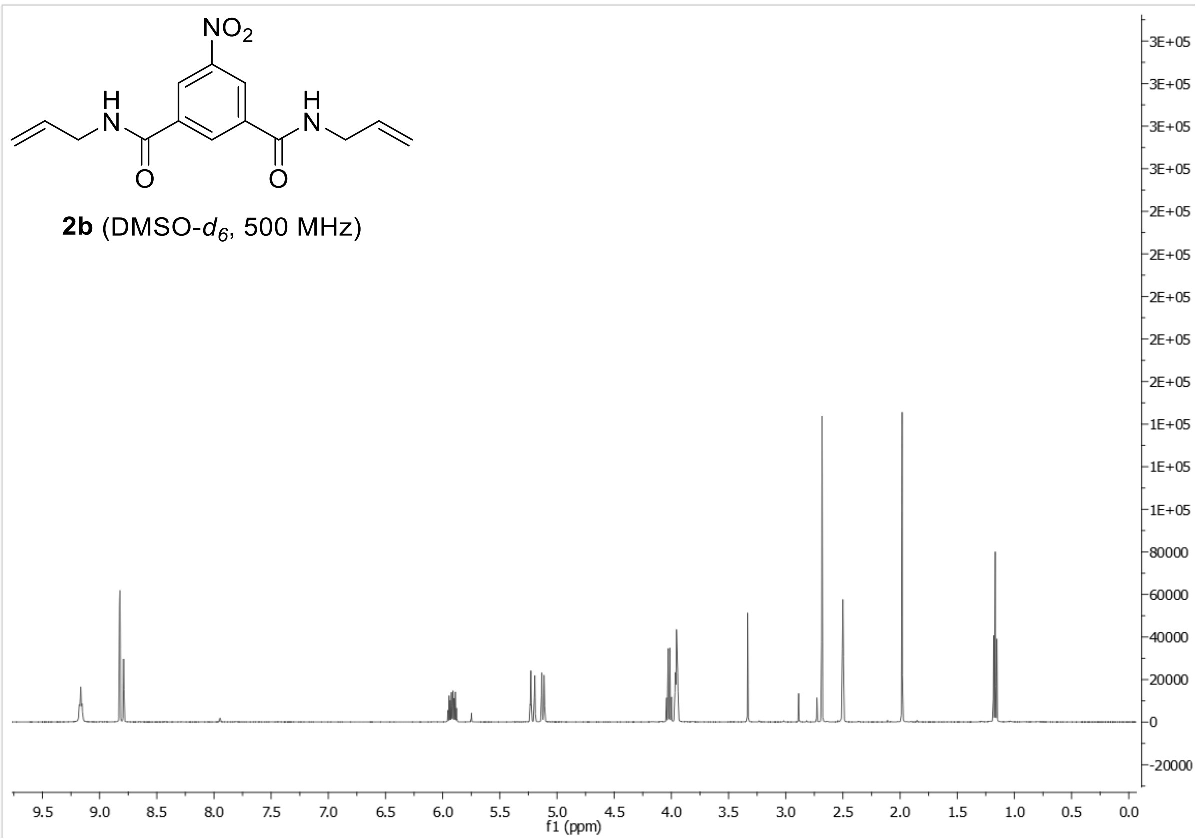

Figure S3. ^{1}H NMR spectrum of **2b**

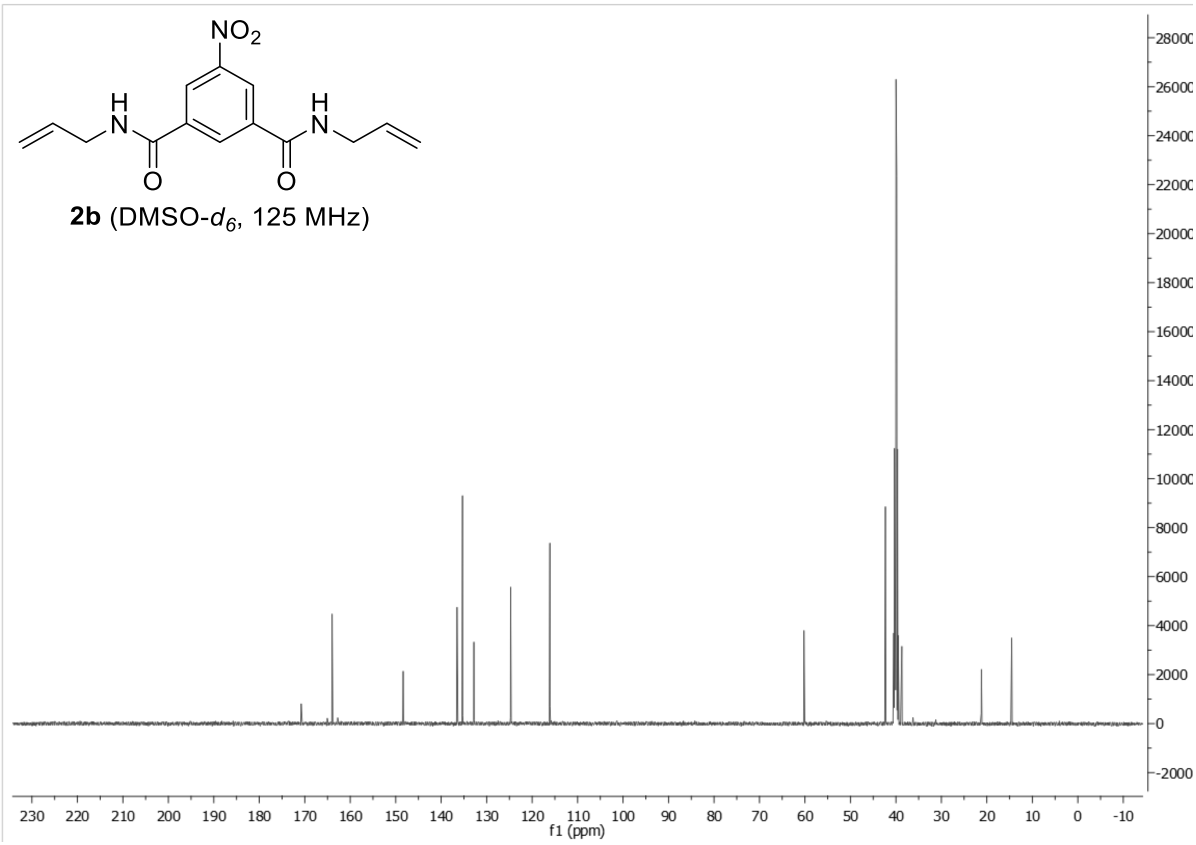

Figure S4. ^{13}C NMR spectrum of **2b**

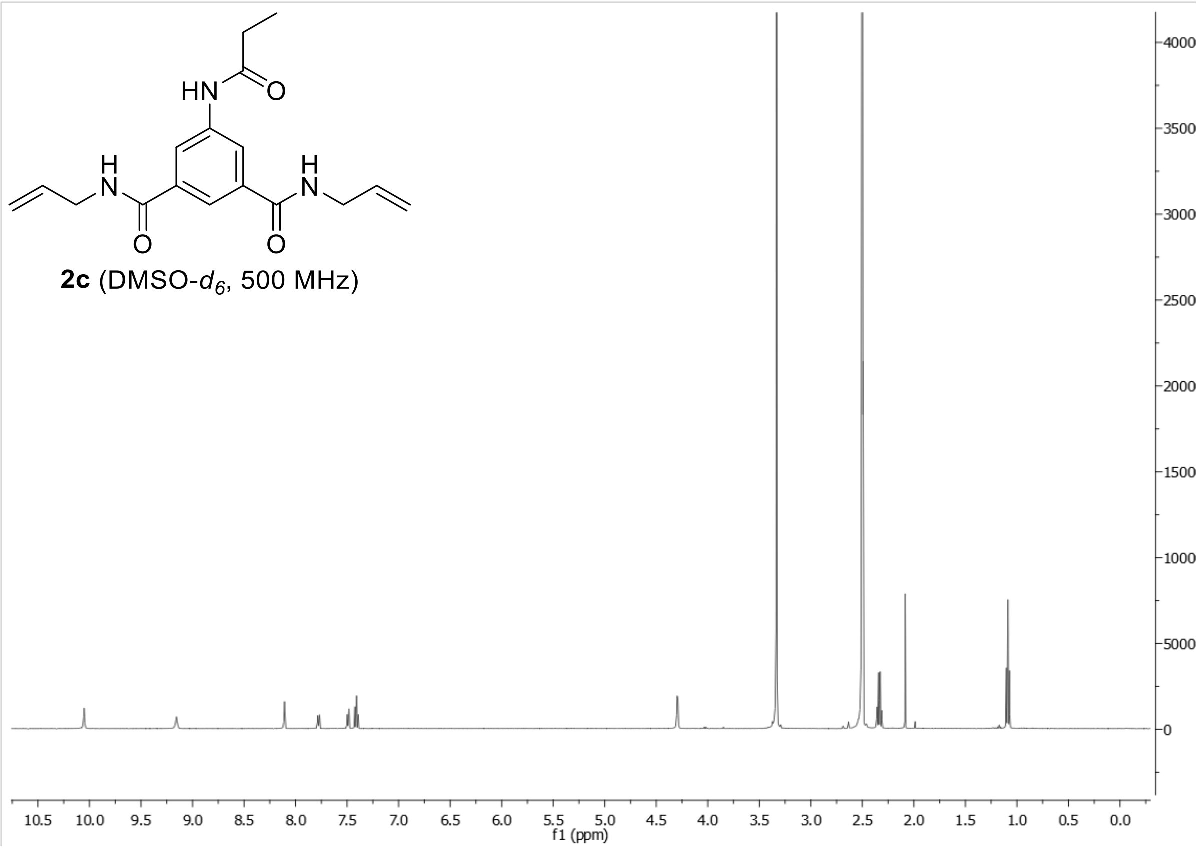

Figure S5. ^{1}H NMR spectrum of **2c**

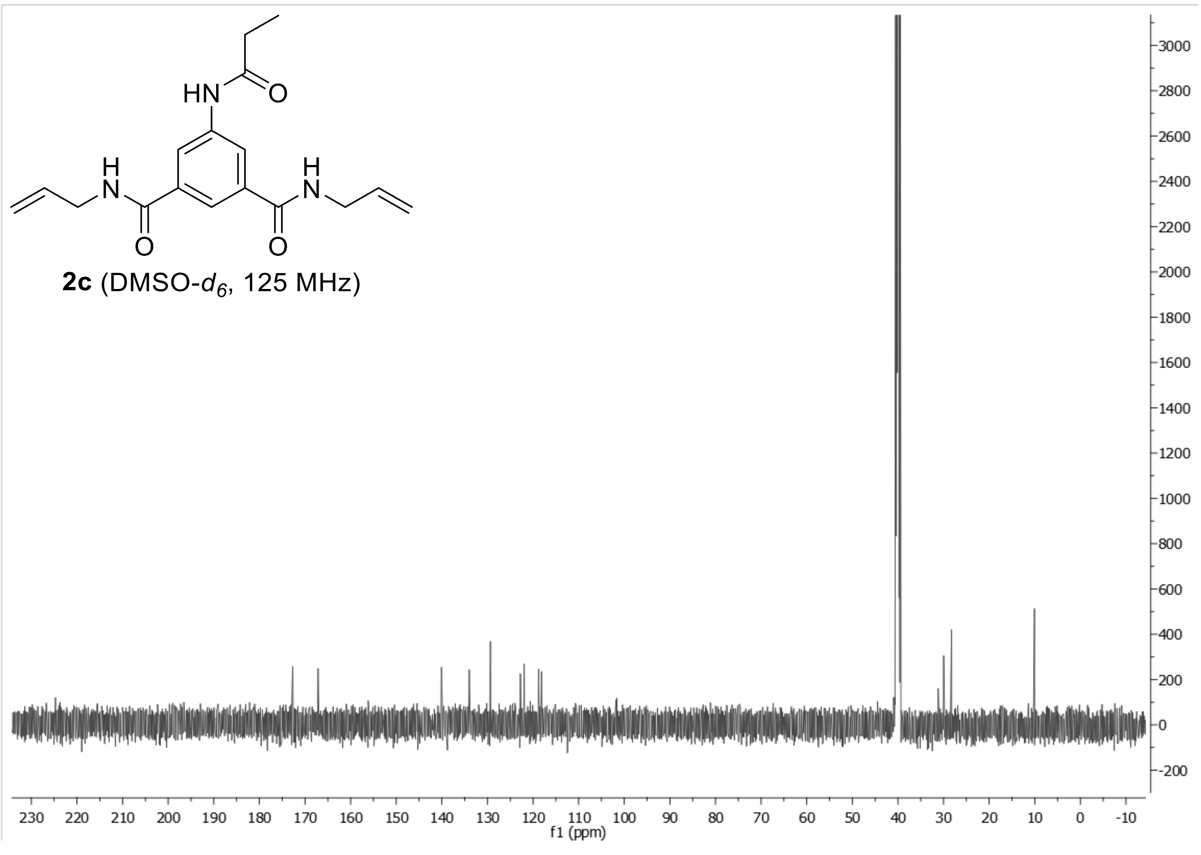

Figure S6. ^{13}C NMR spectrum of **2c**

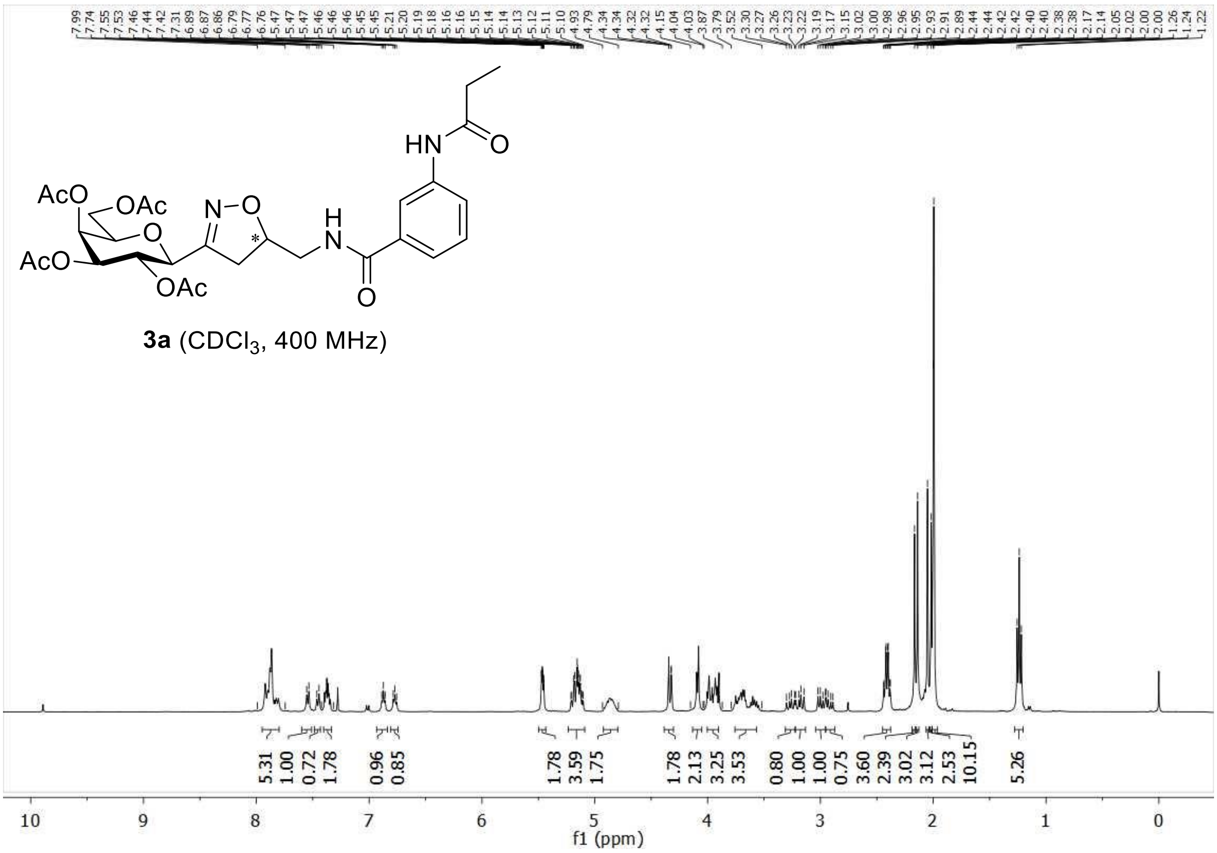

Figure S7. ^{1}H NMR spectrum of **3a**

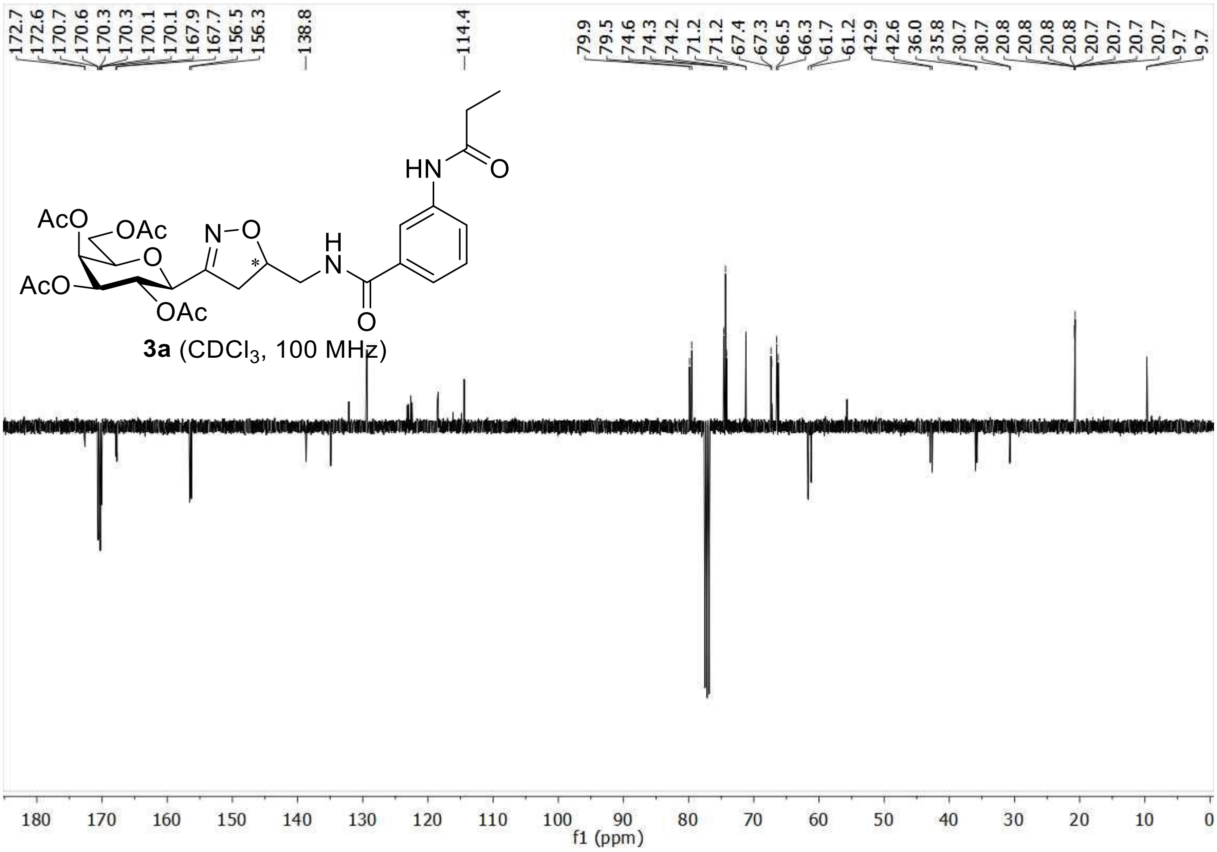

Figure S8. ^{13}C NMR spectrum of **3a**

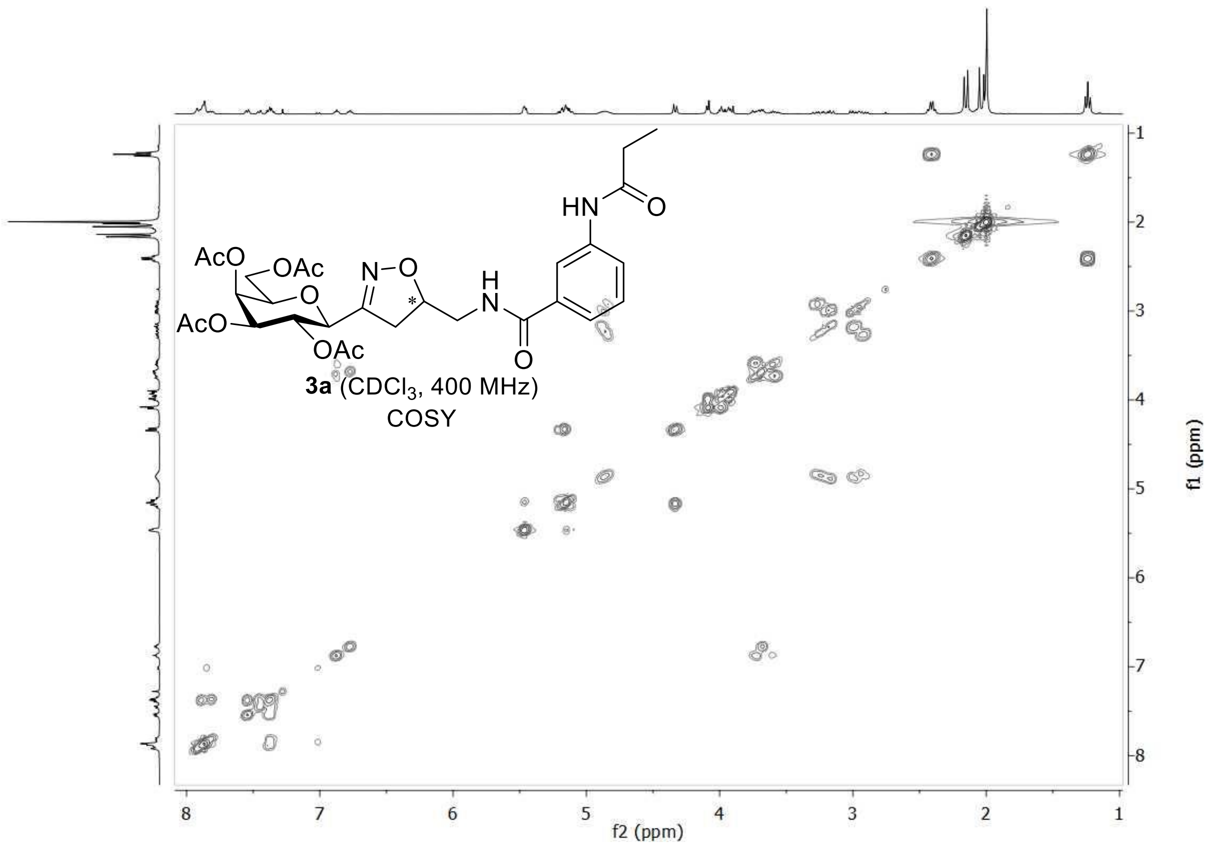

Figure S9. ^{1}H–^{1}H COSY spectrum of **3a**

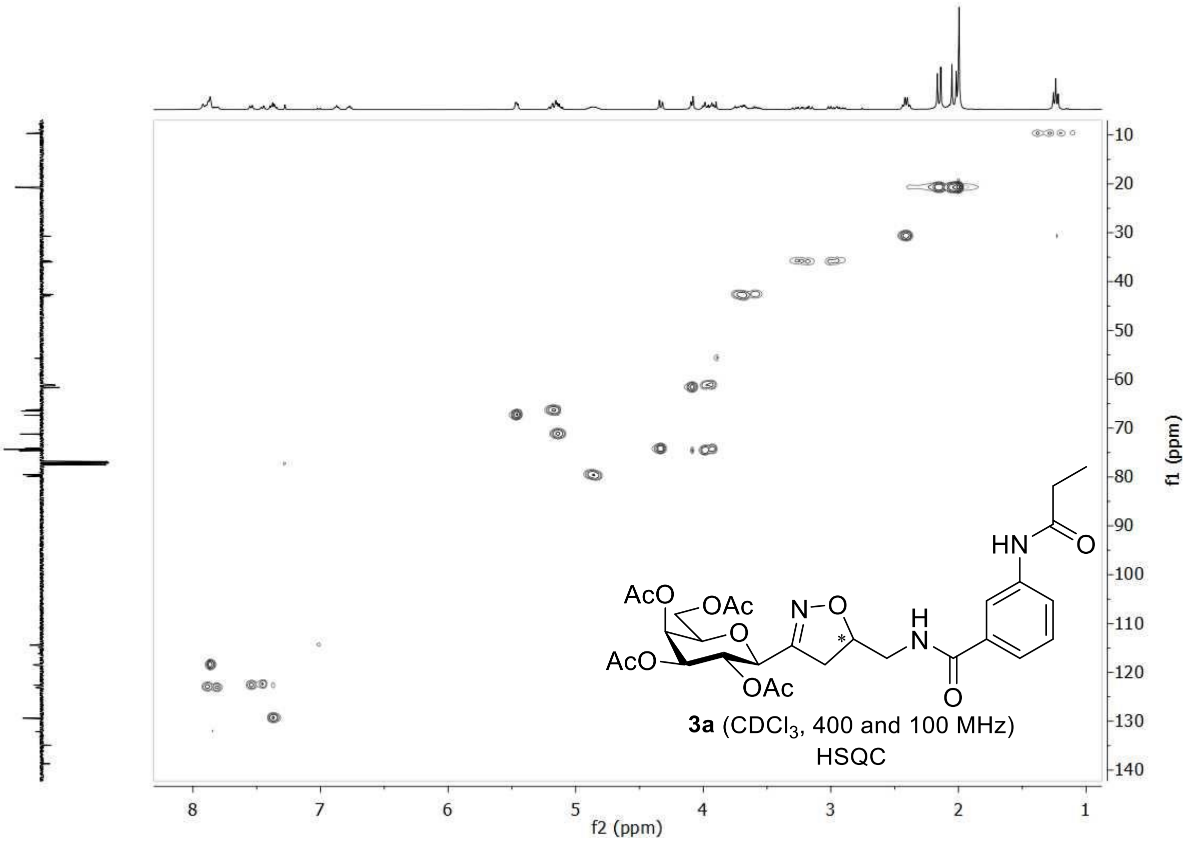

Figure S10. ^{1}H–^{13}C HSQC spectrum of **3a**

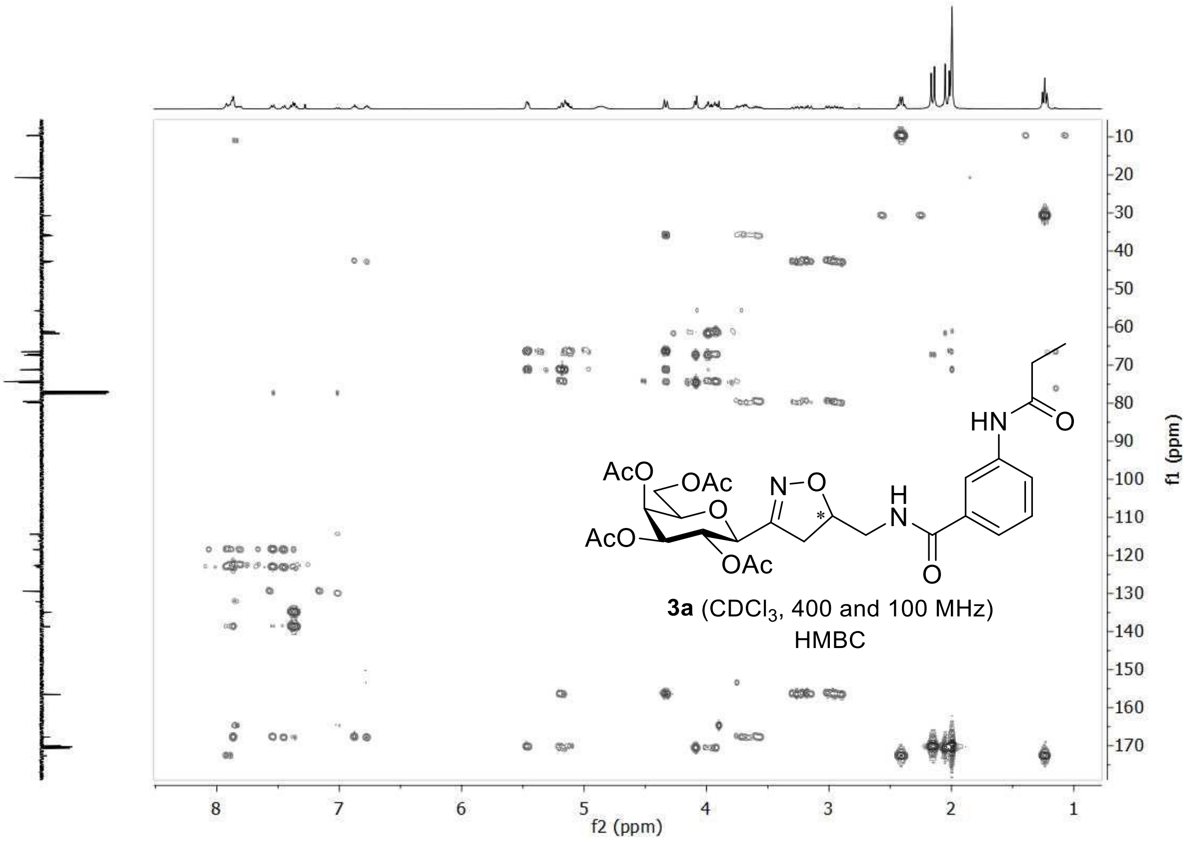

Figure S11. ^{1}H–^{13}C HMBC spectrum of **3a**

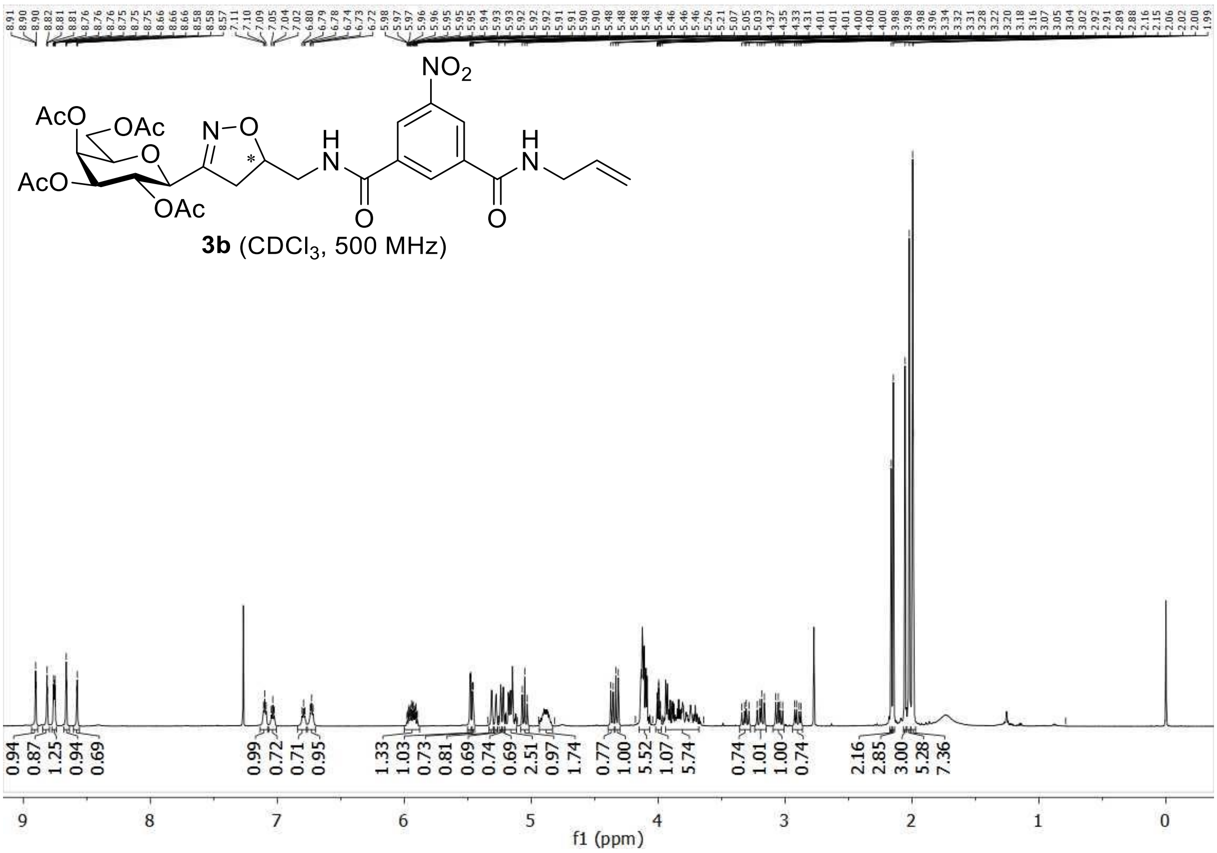

Figure S12. ^{1}H NMR spectrum of **3b**

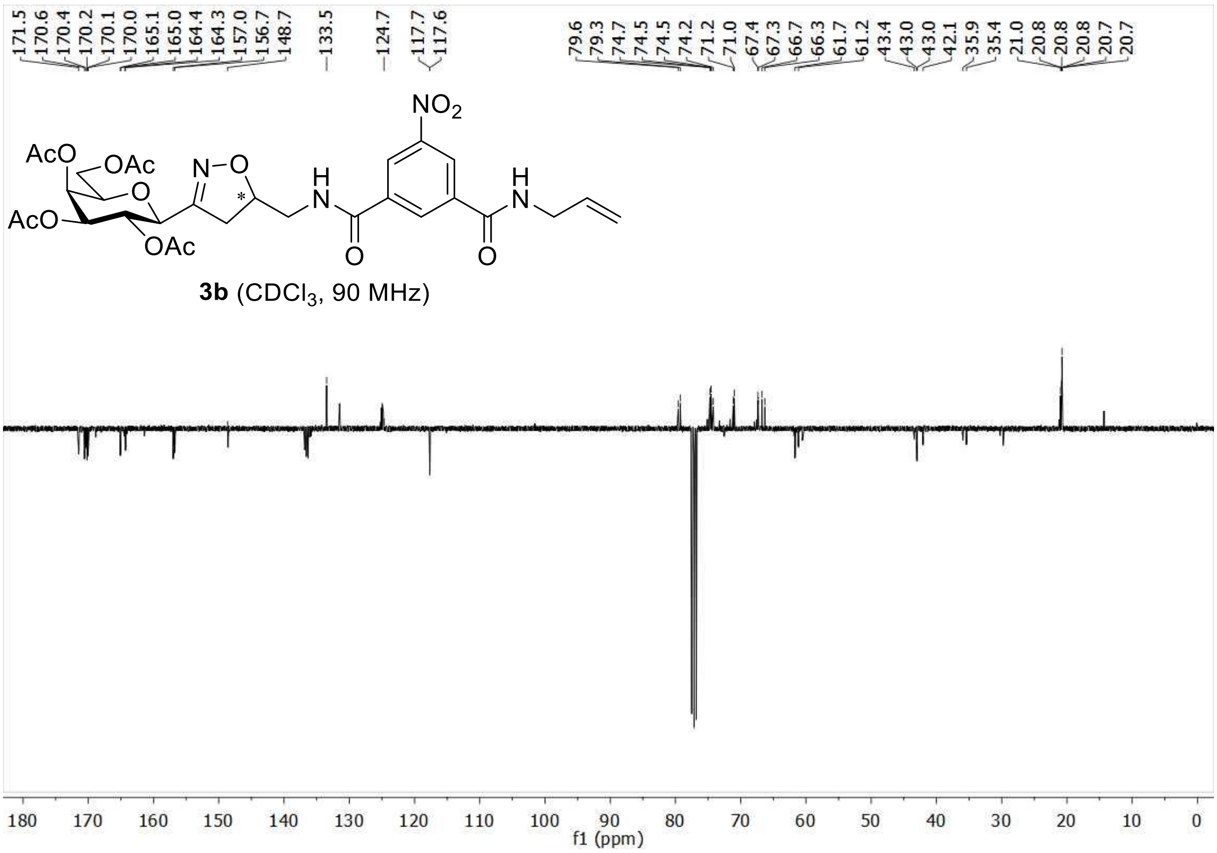

Figure S13. ^{13}C NMR spectrum of **3b**

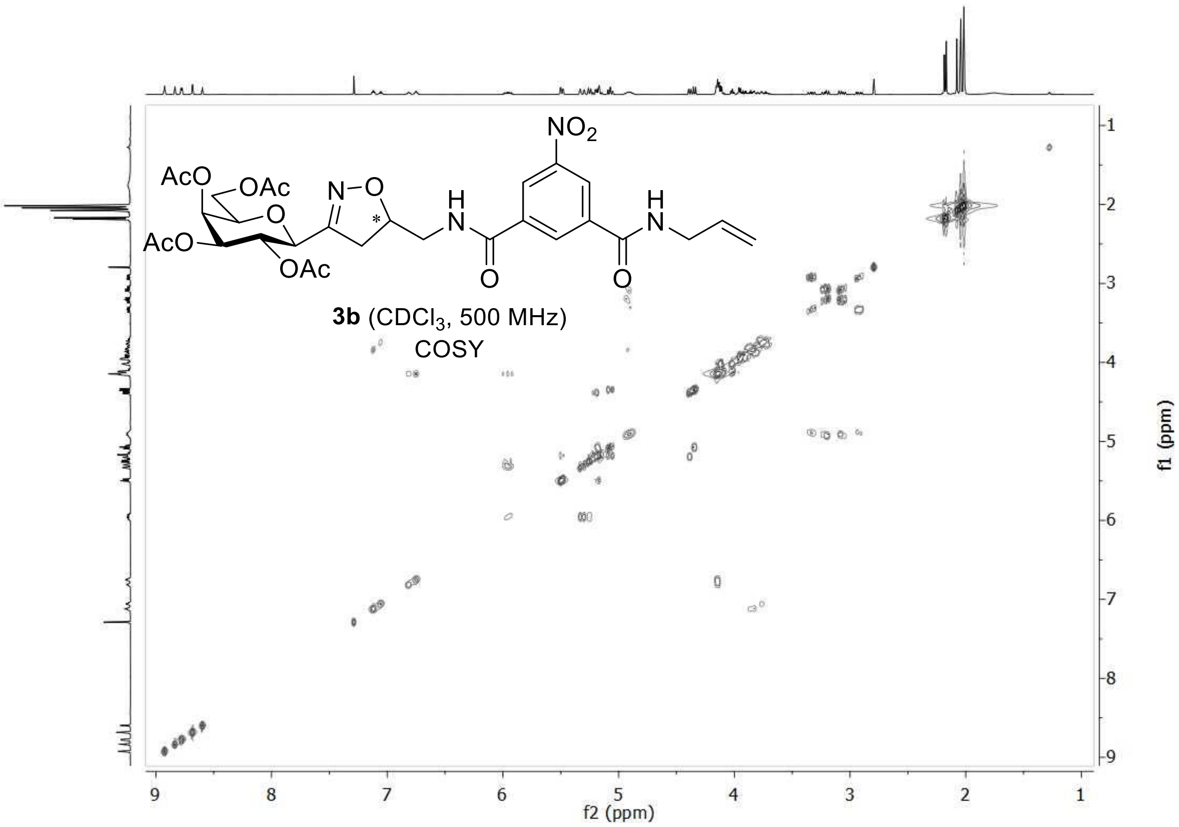

Figure S14. ^{1}H–^{1}H COSY spectrum of **3b**

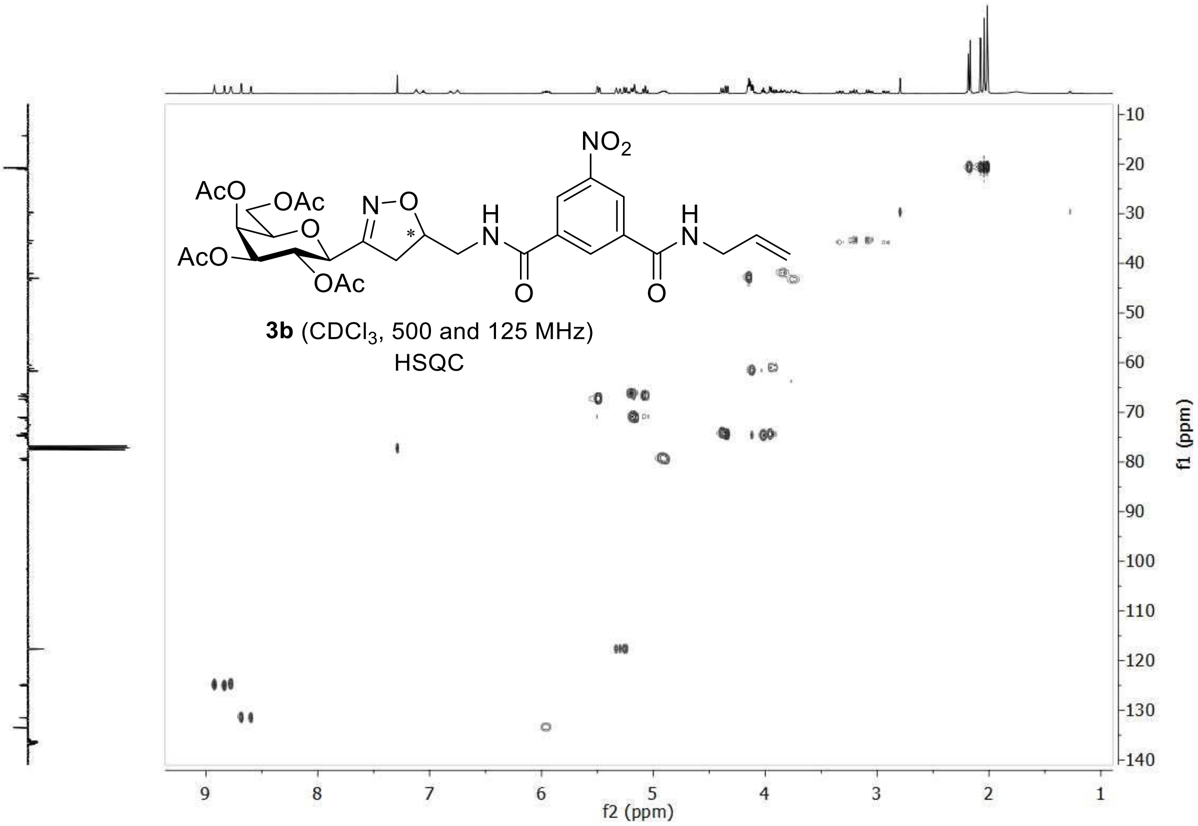

Figure S15. ^{1}H–^{13}C HSQC spectrum of **3b**

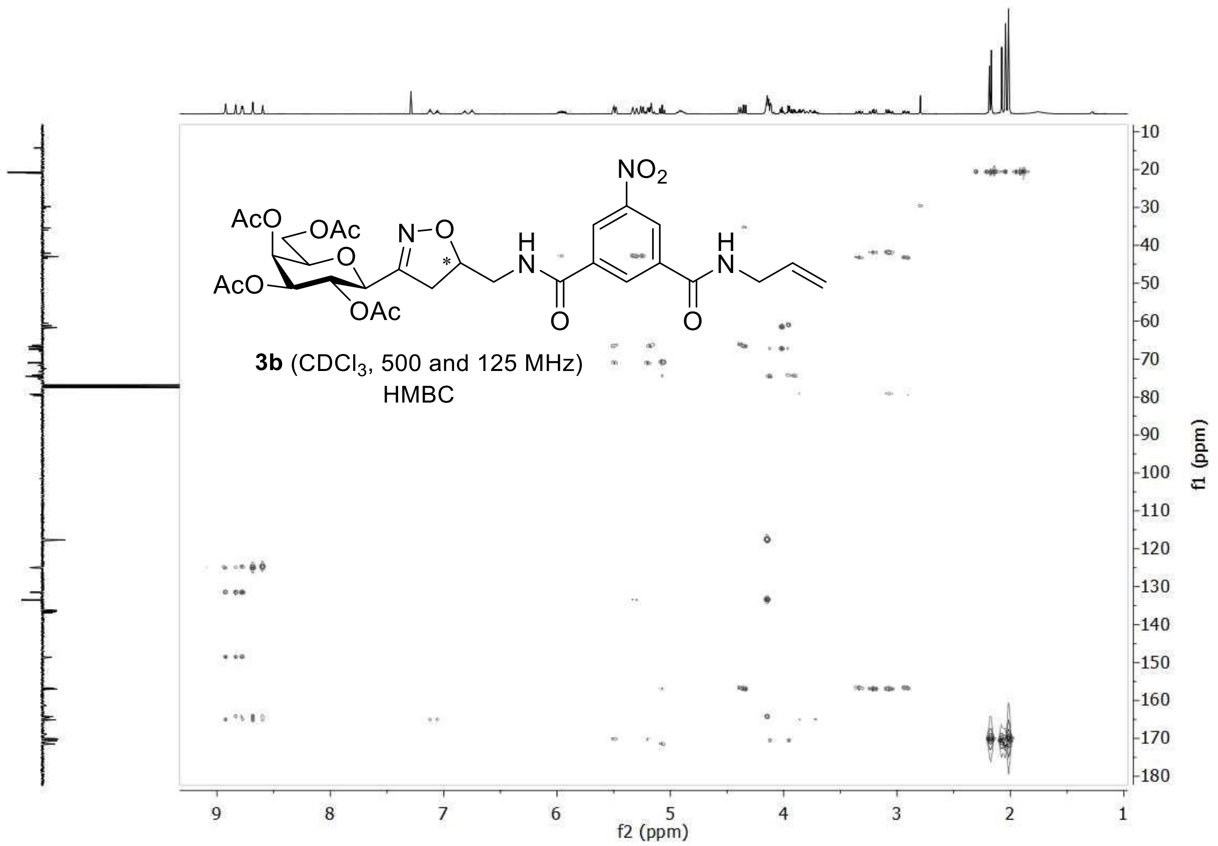

Figure S16. ^{1}H–^{13}C HMBC spectrum of **3b**

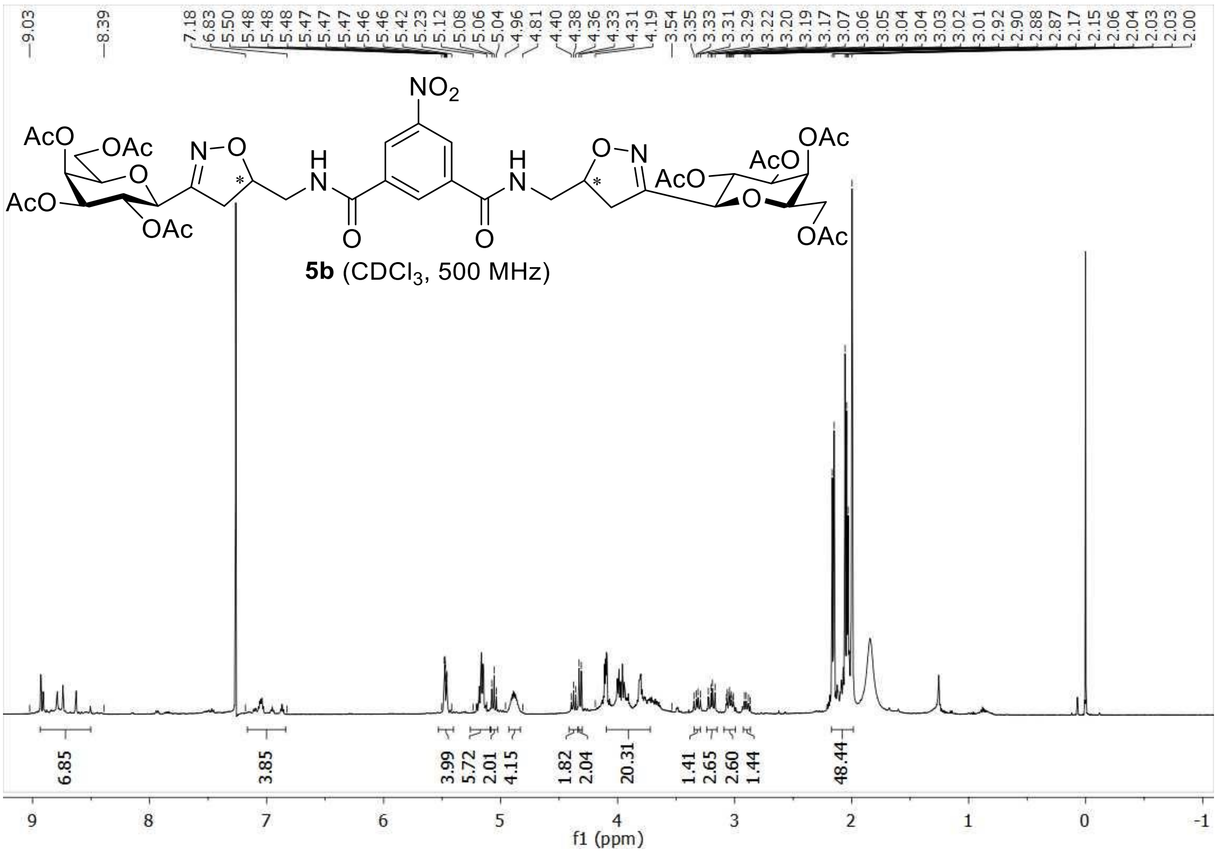

Figure S17. ^{1}H NMR spectrum of **5b**

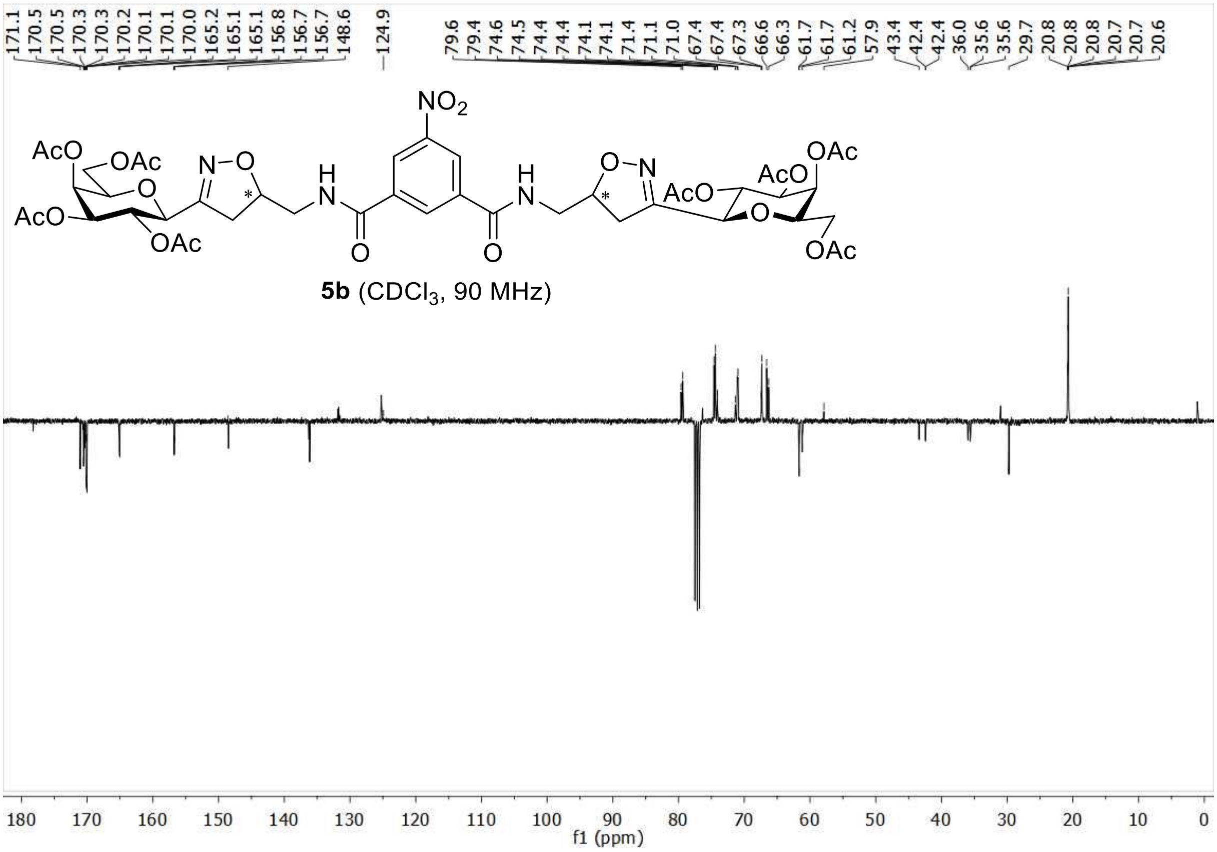

Figure S18. ^{13}C NMR spectrum of **5b**

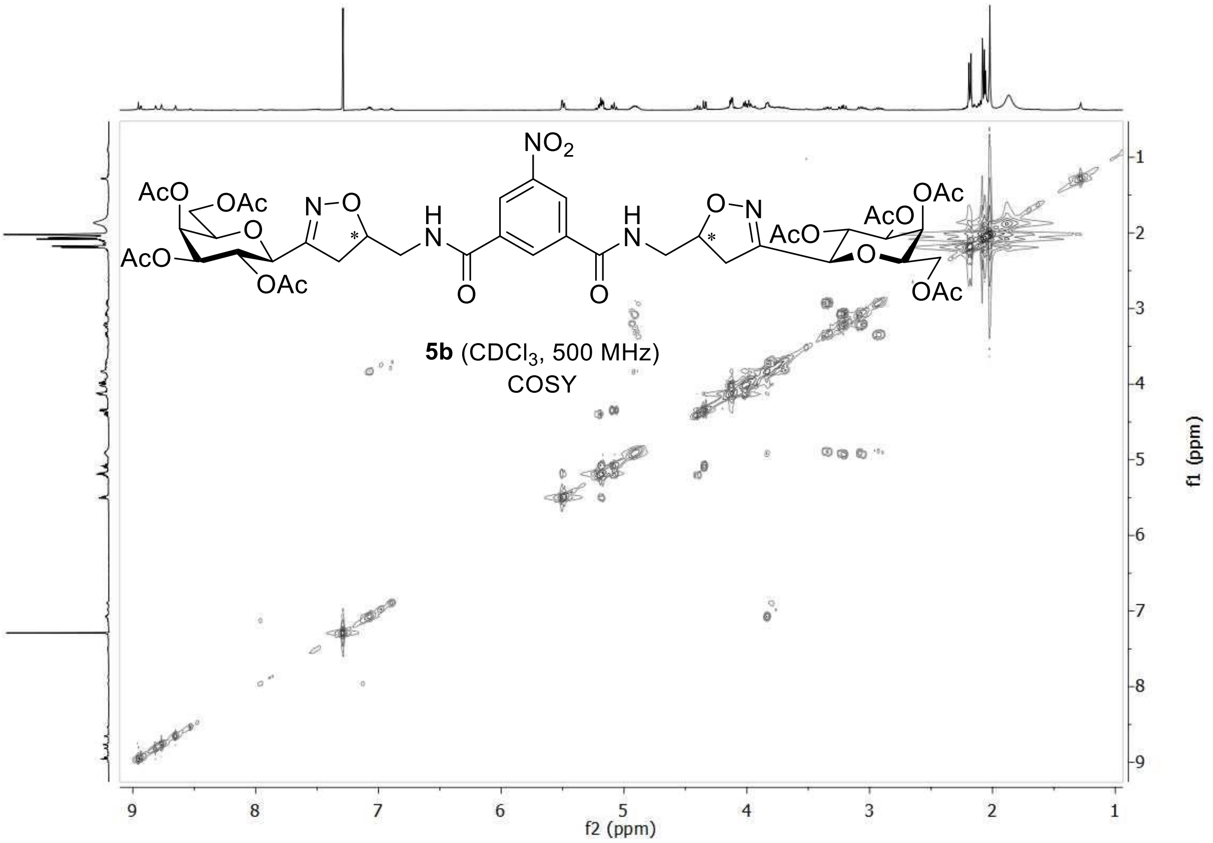

Figure S19. ^{1}H–^{1}H COSY spectrum of **5b**

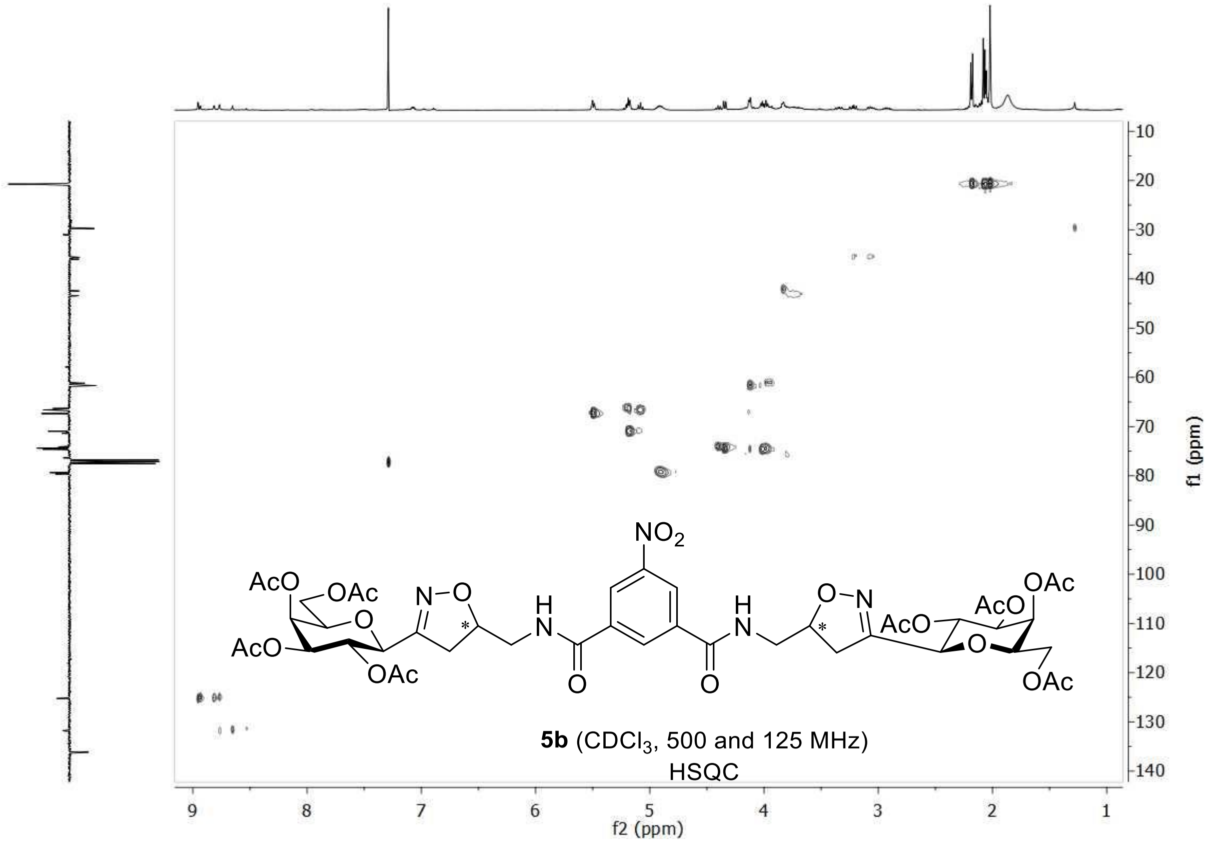

Figure S20. ^{1}H–^{13}C HSQC spectrum of **5b**

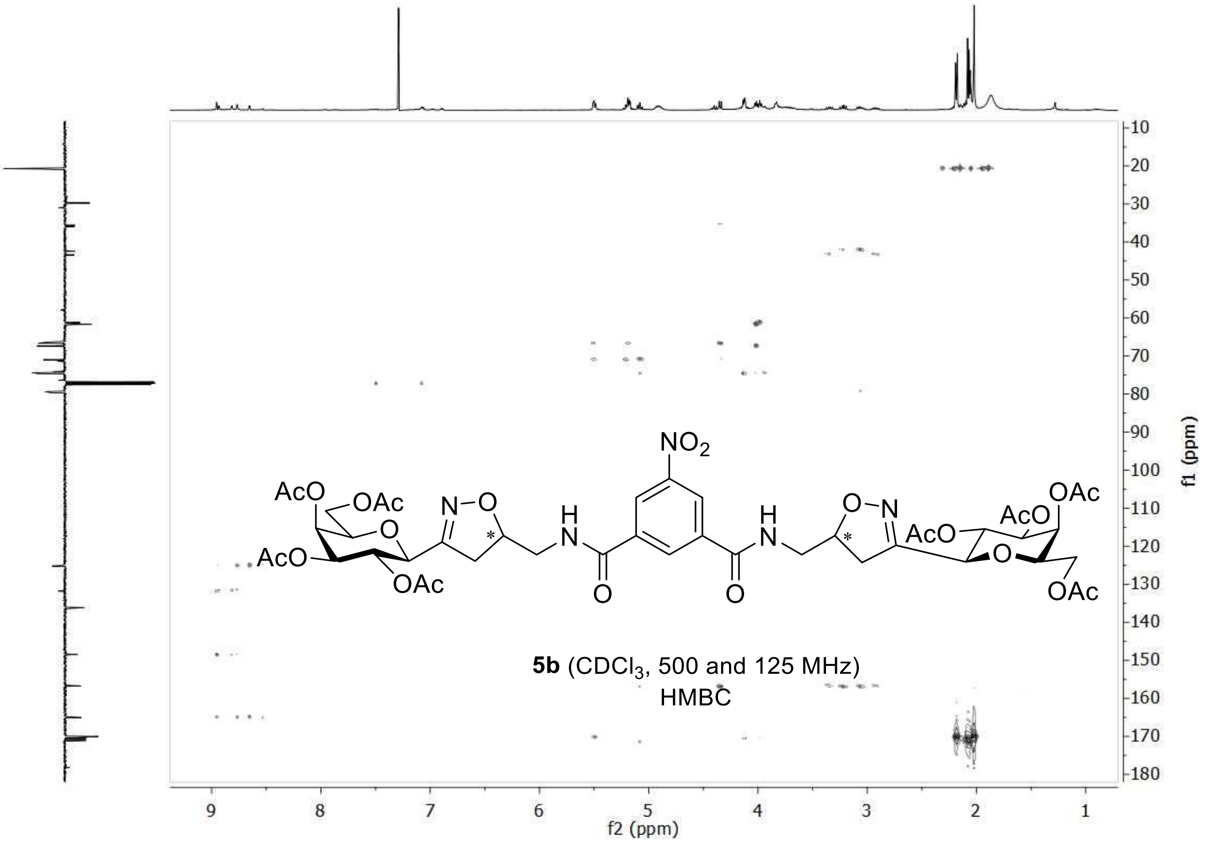

Figure S21. ^{1}H–^{13}C HMBC spectrum of **5b**

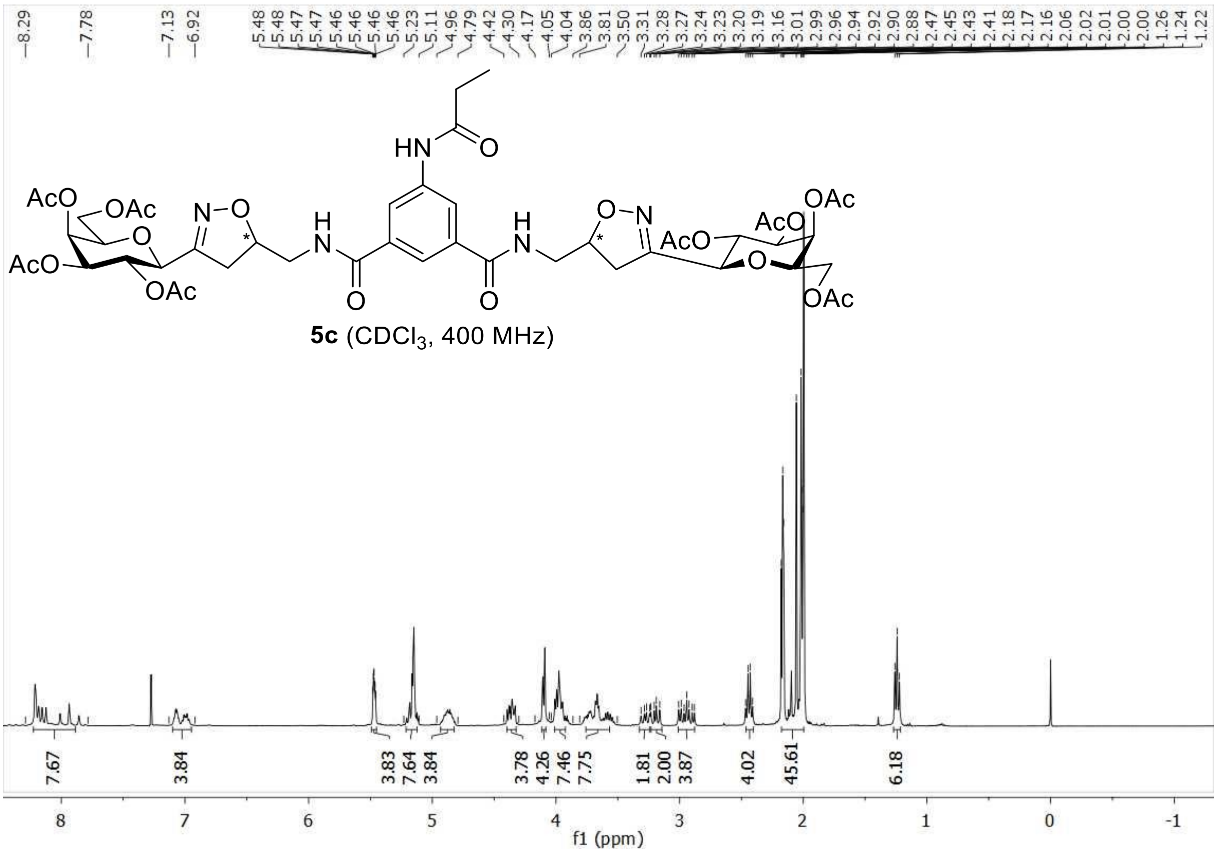

Figure S22. ^{1}H NMR spectrum of **5c**

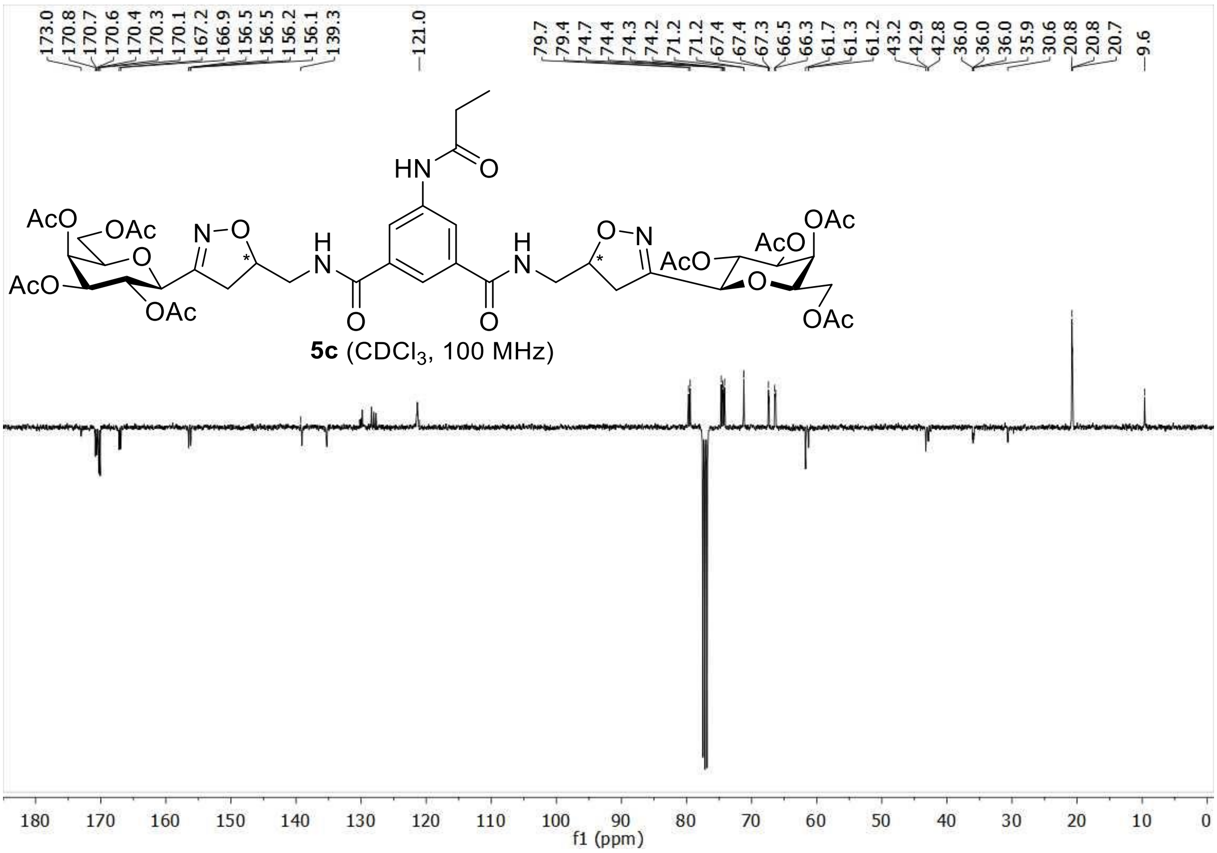

Figure S23. ^{13}C NMR spectrum of **5c**

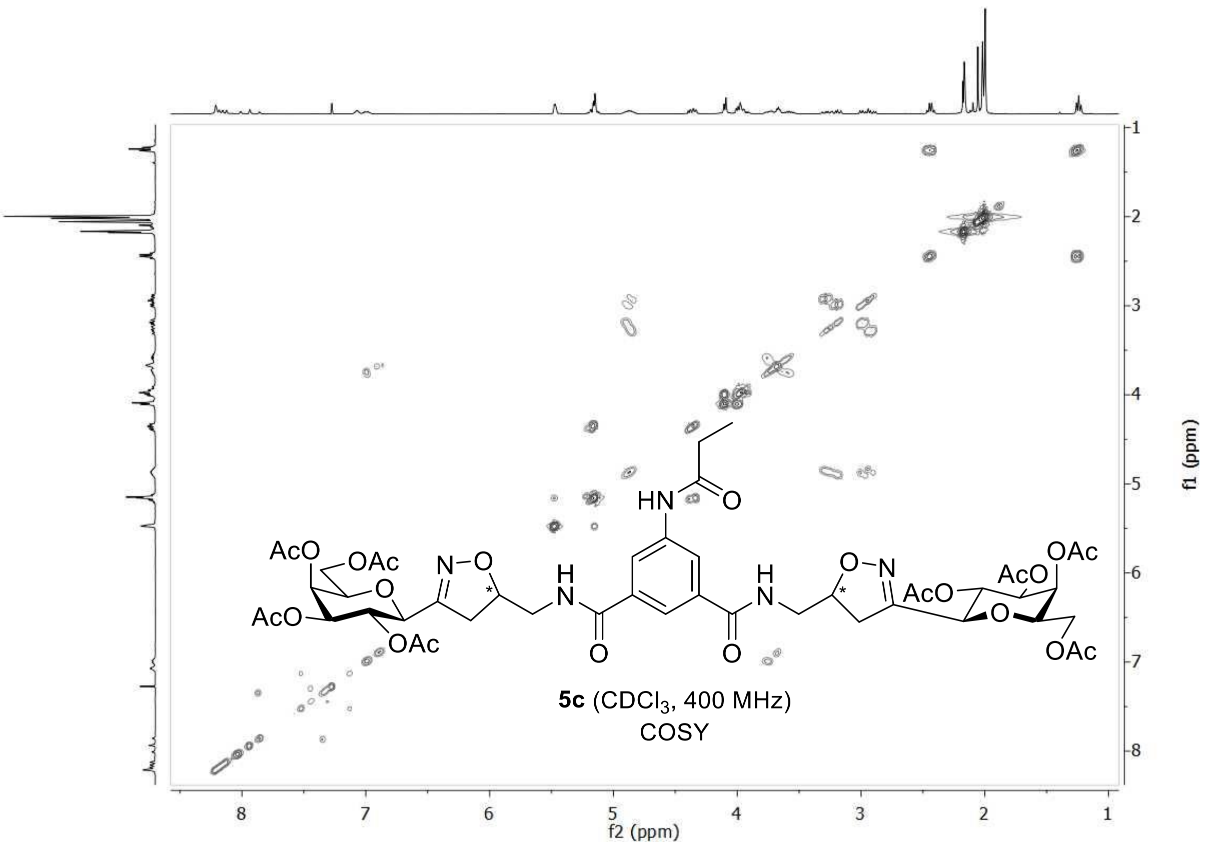

Figure S24. ^{1}H–^{1}H COSY spectrum of **5c**

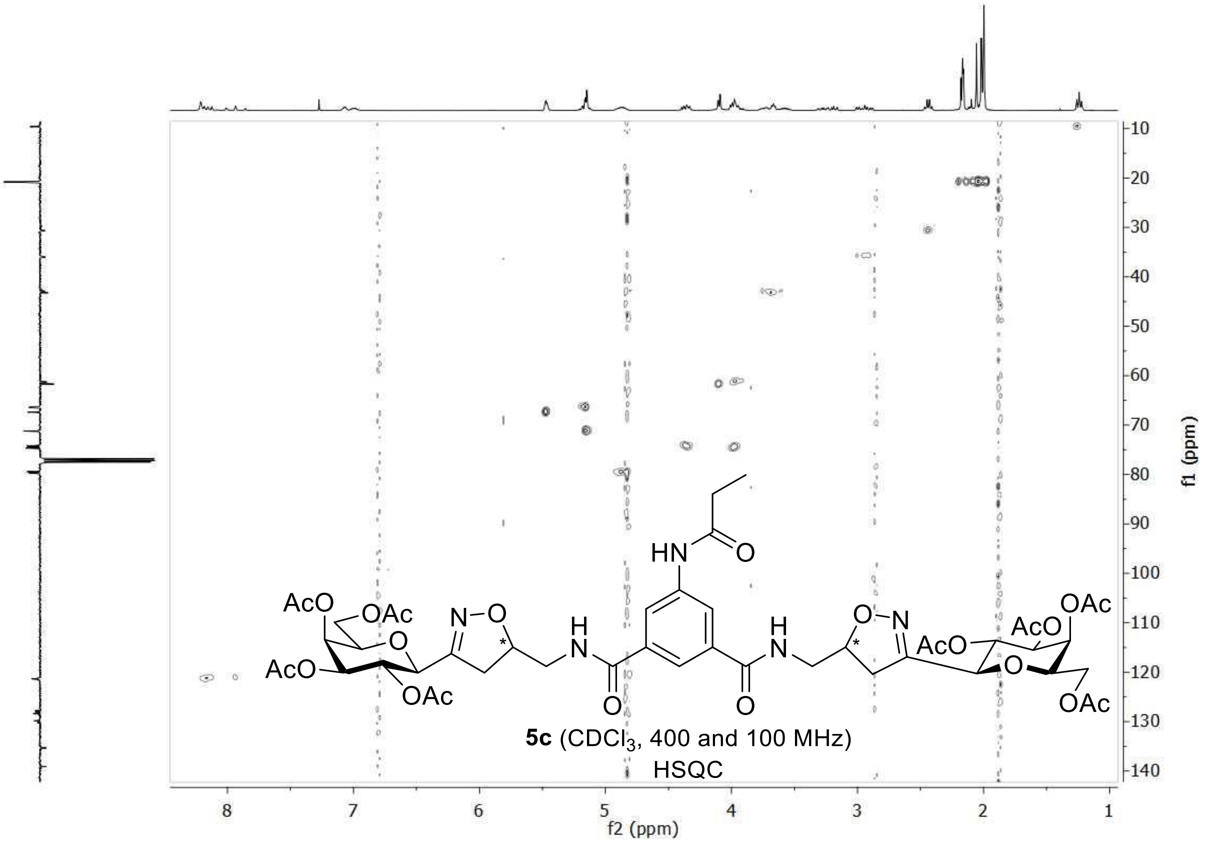

Figure S25. ^{1}H–^{13}C HSQC spectrum of **5c**

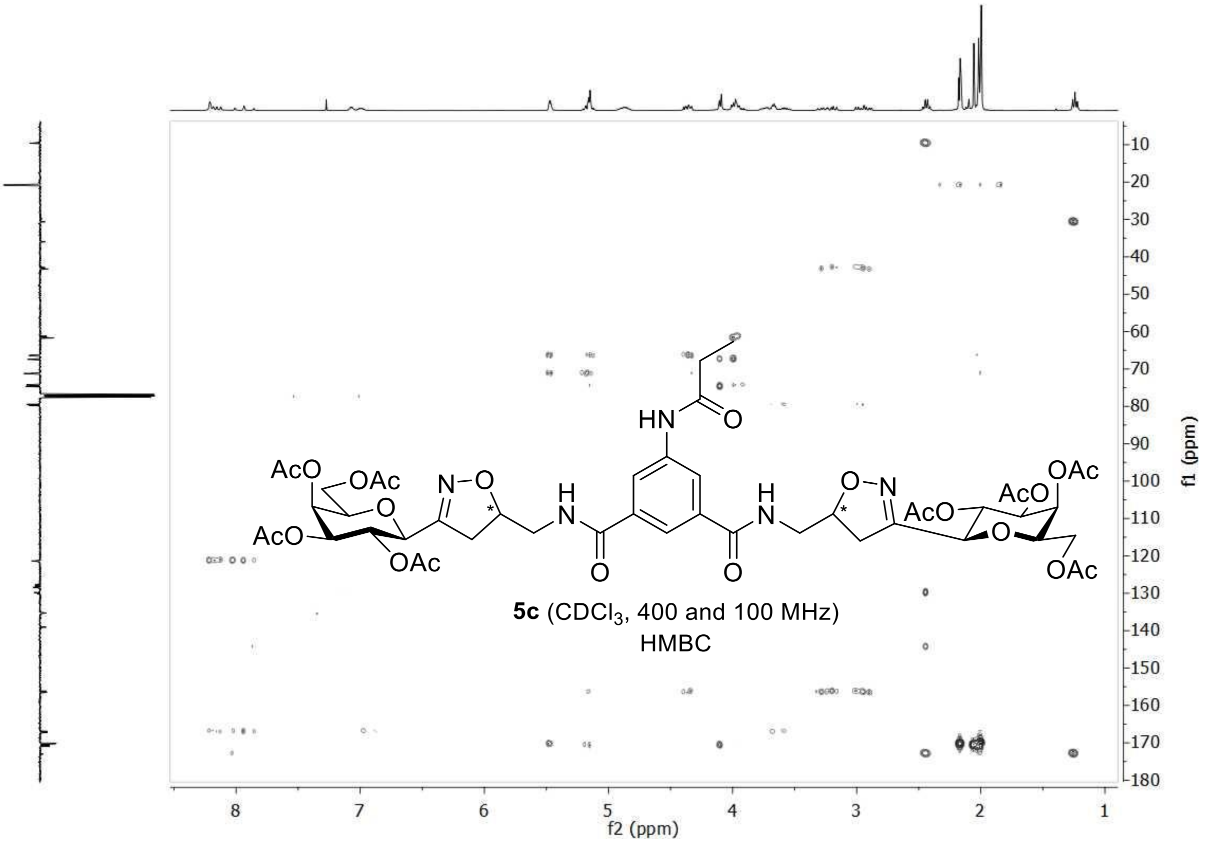

Figure S26. ¹H–¹³C HMBC spectrum of **5c**

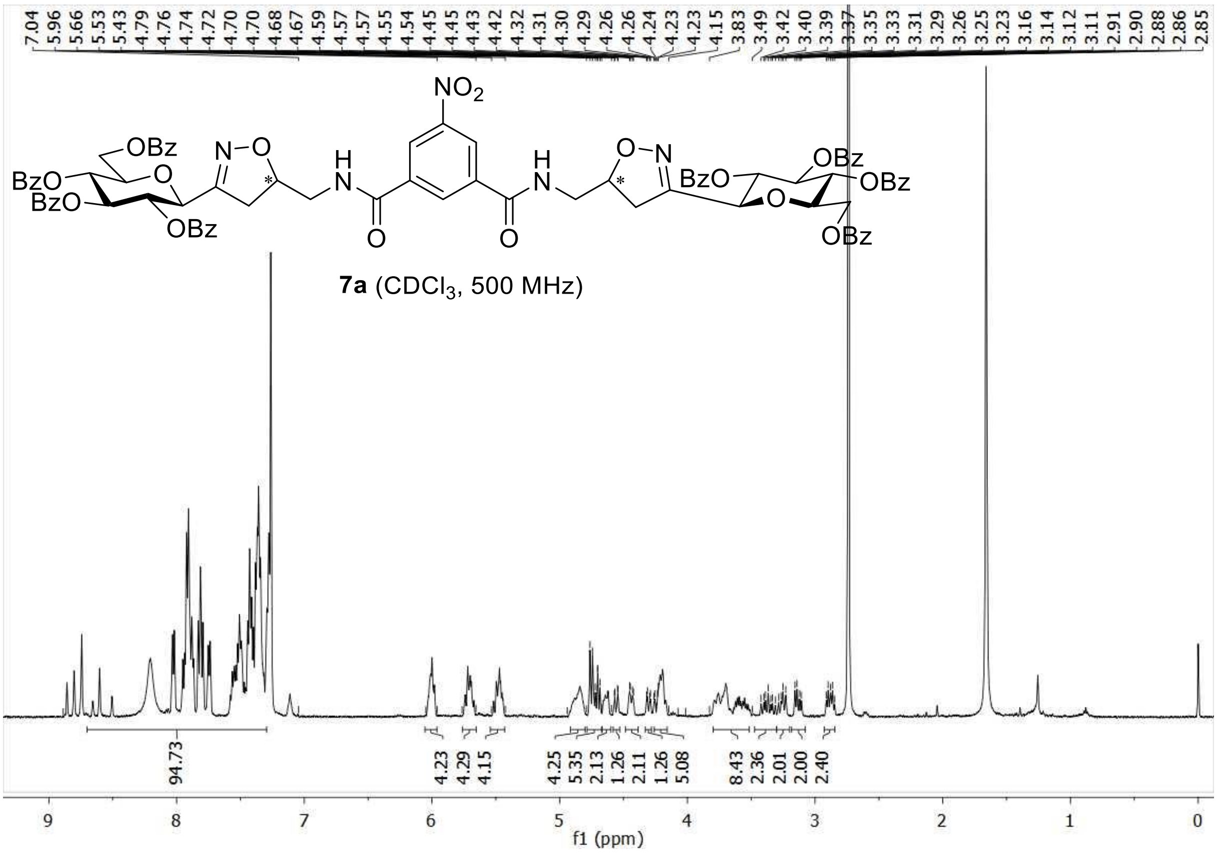

Figure S27. ^{1}H NMR spectrum of **7a**

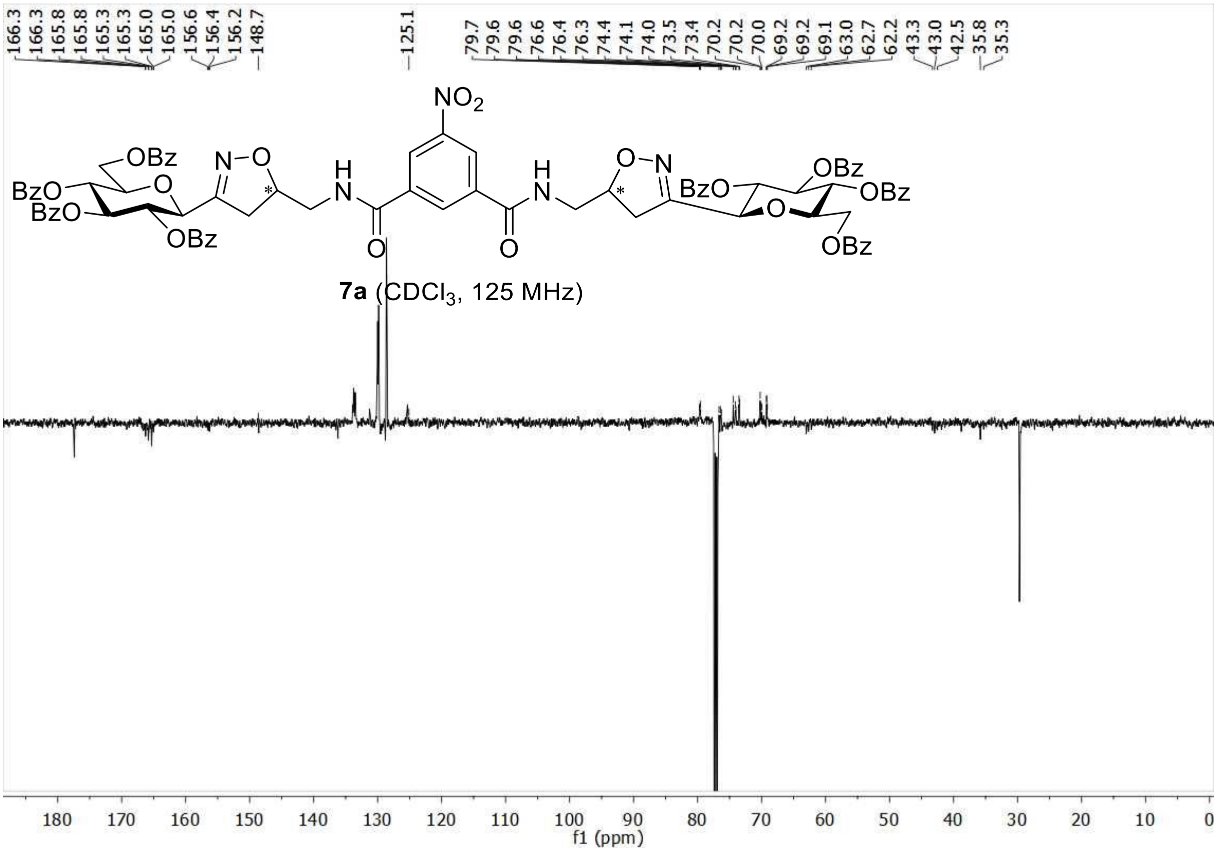

Figure S28. ^{13}C NMR spectrum of **7a**

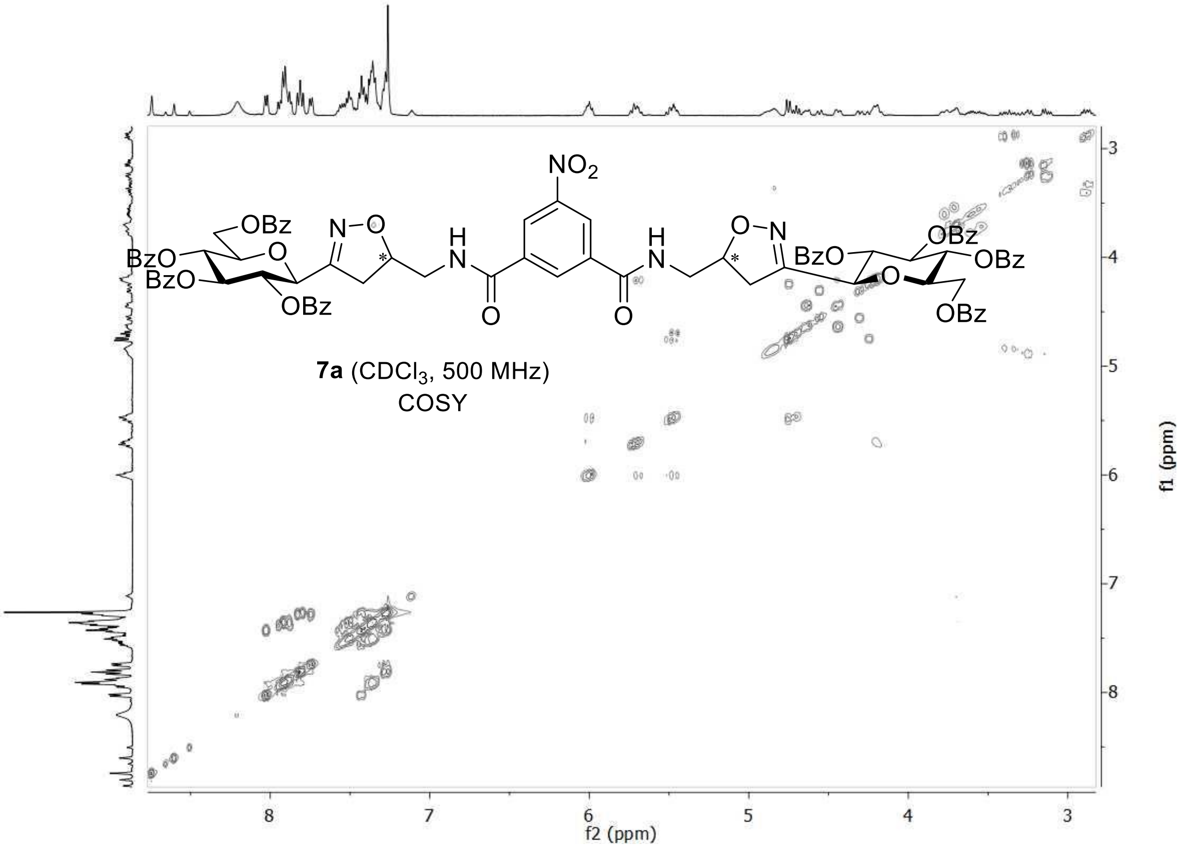

Figure S29. ¹H–¹H COSY spectrum of **7a**

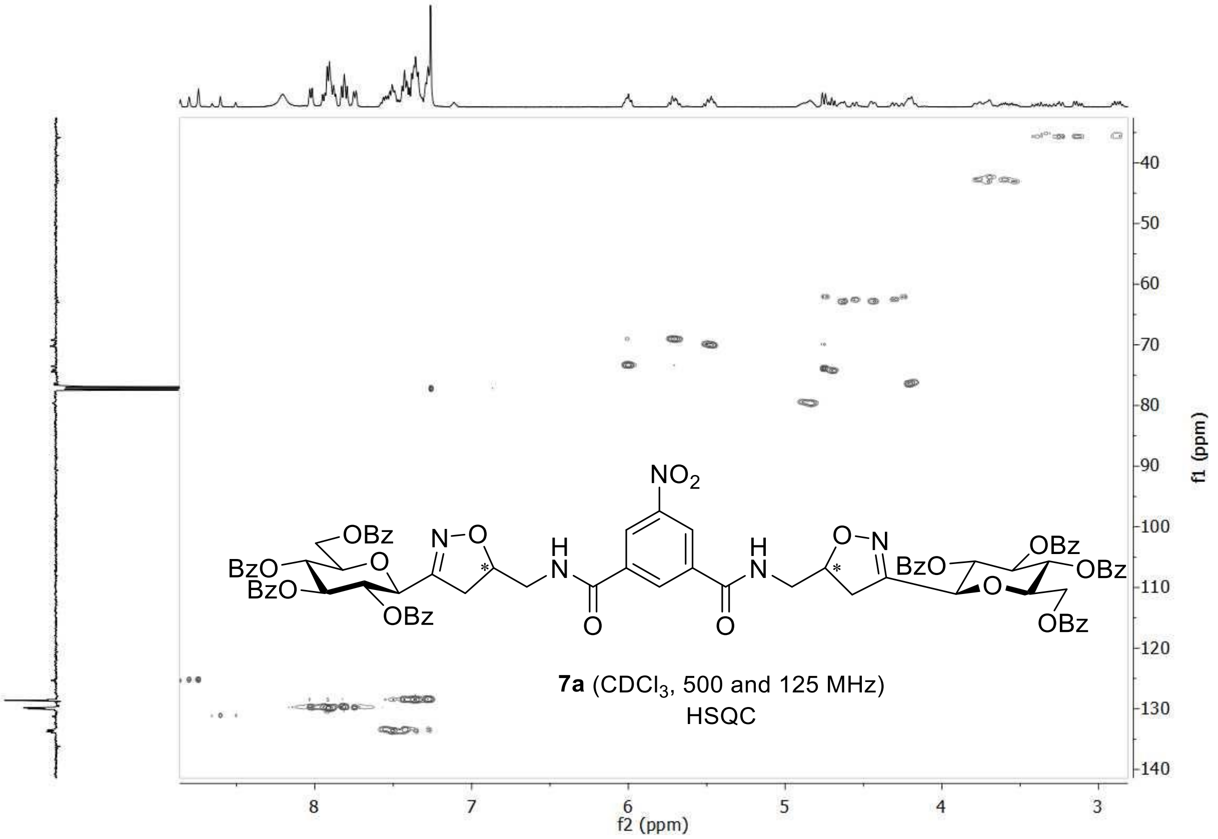

Figure S30. ¹H–¹³C HSQC spectrum of **7a**

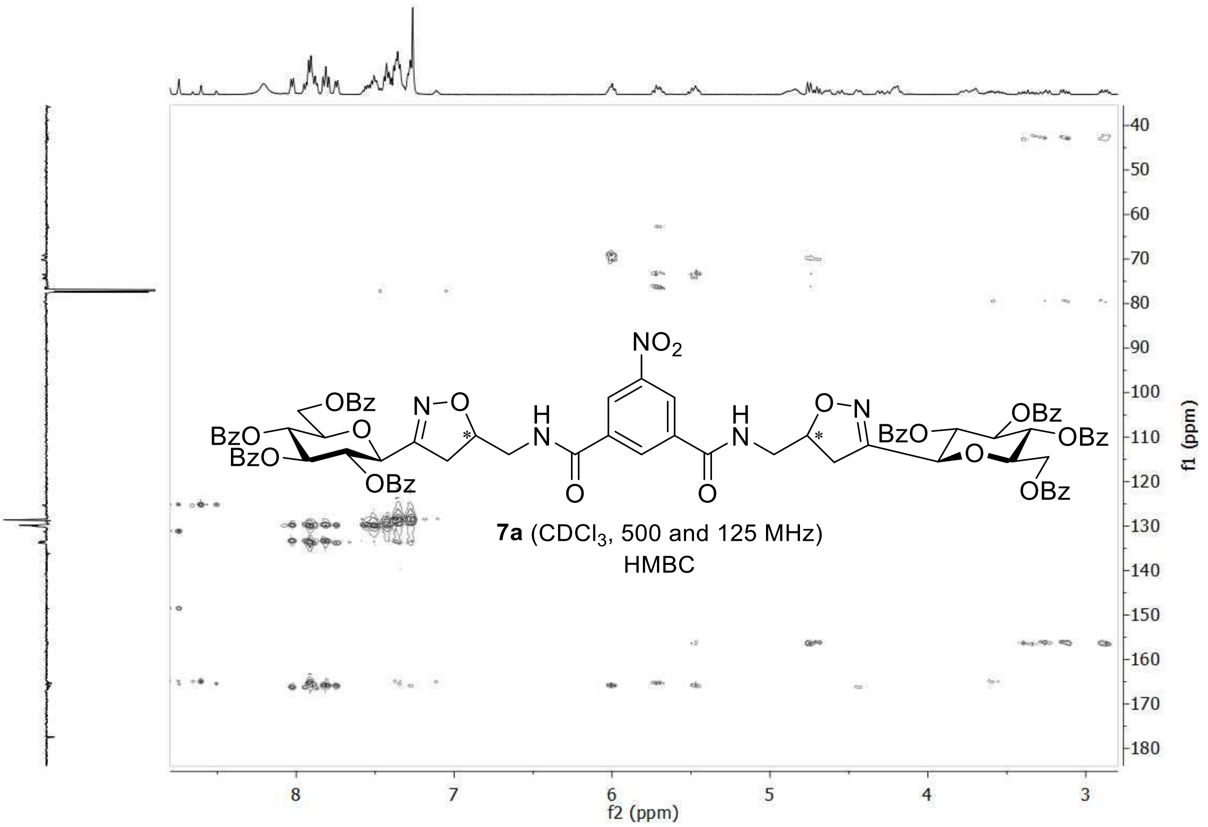

Figure S31. ^{1}H–^{13}C HMBC spectrum of **7a**

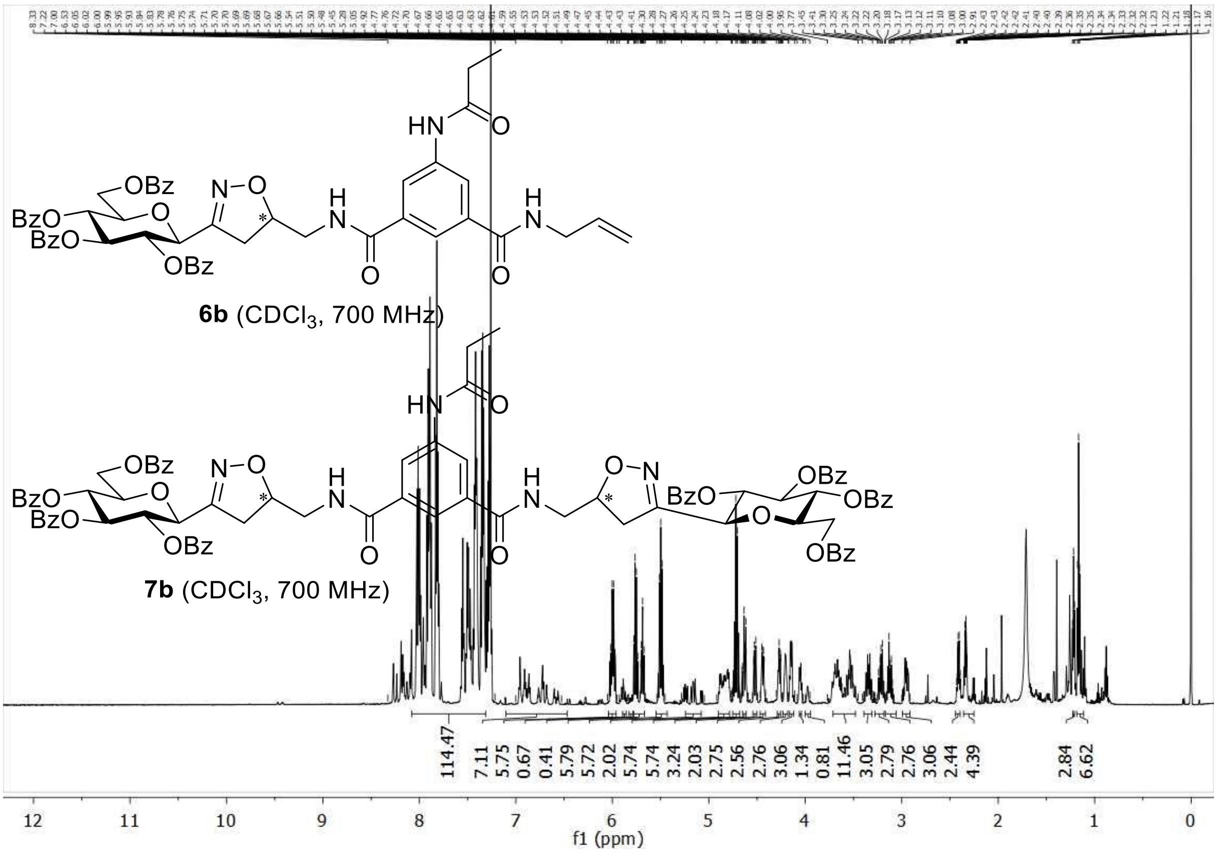

Figure S32. ^{1}H NMR spectrum of **6b** and **7b**

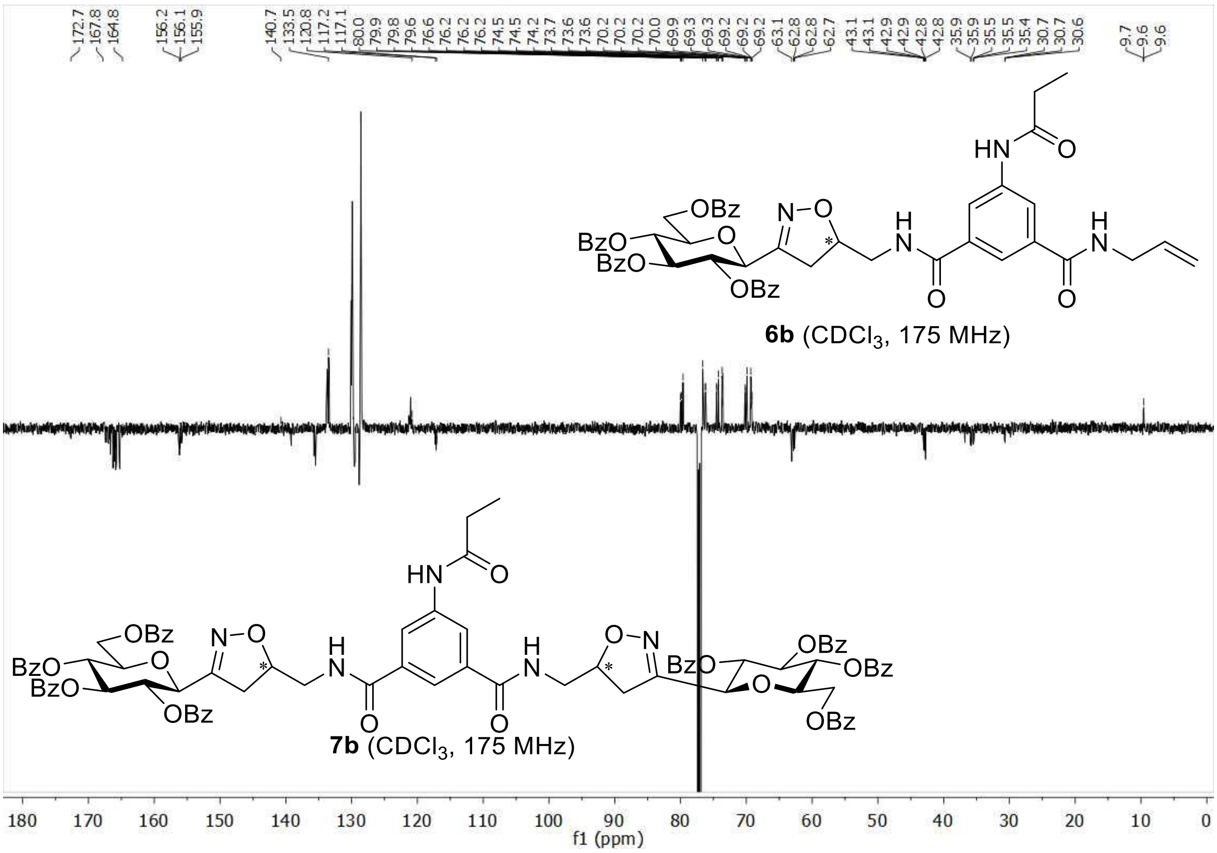

Figure S33. ^{13}C NMR spectrum of **6b** and **7b**

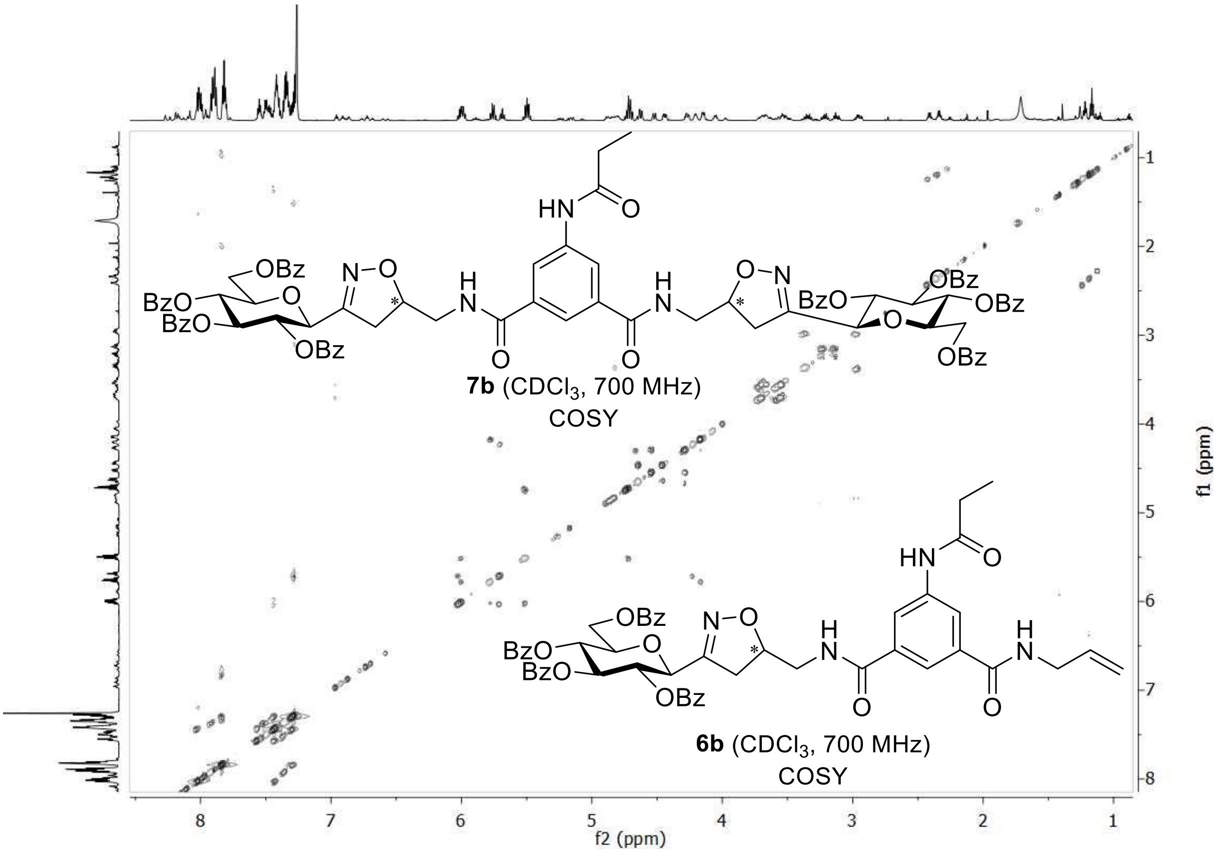

Figure S34. ^{1}H–^{1}H COSY spectrum of **6b** and **7b**

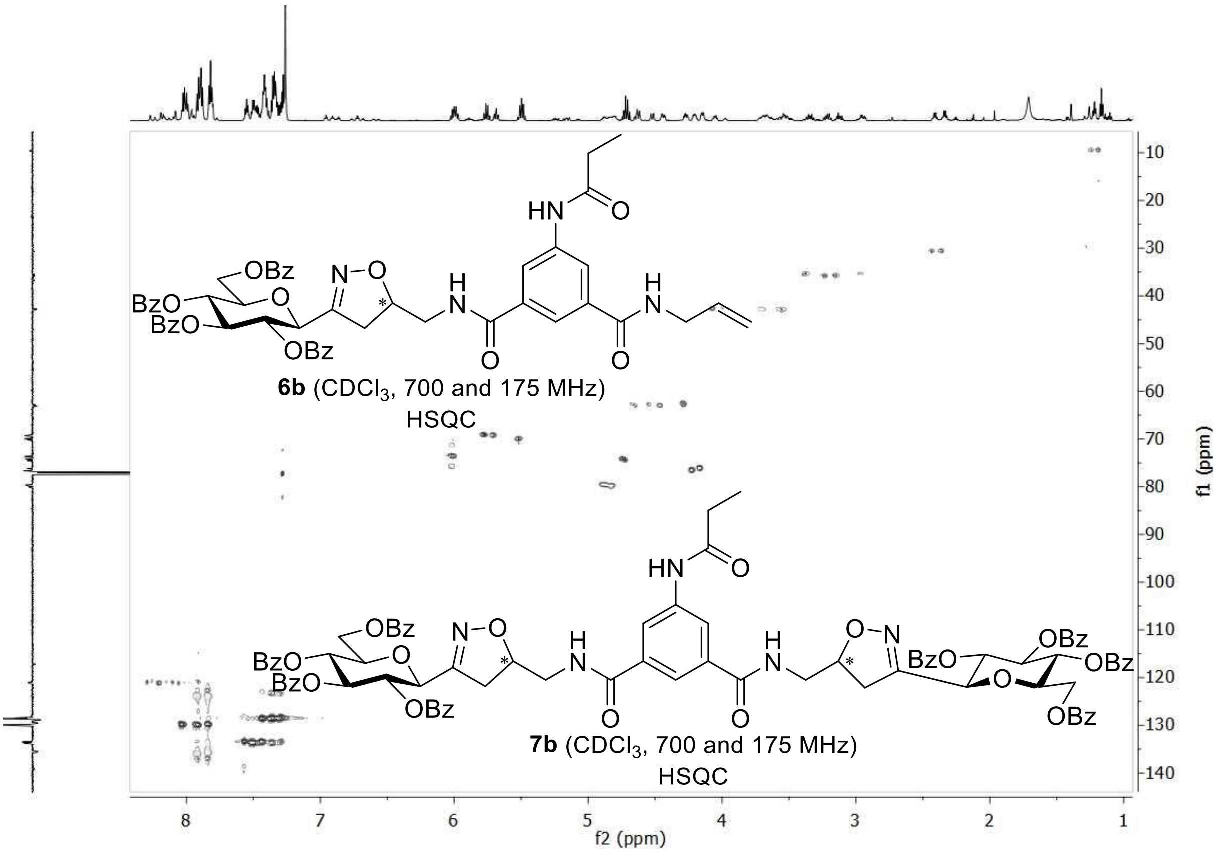

Figure S35. ^{1}H–^{13}C HSQC spectrum of **6b** and **7b**

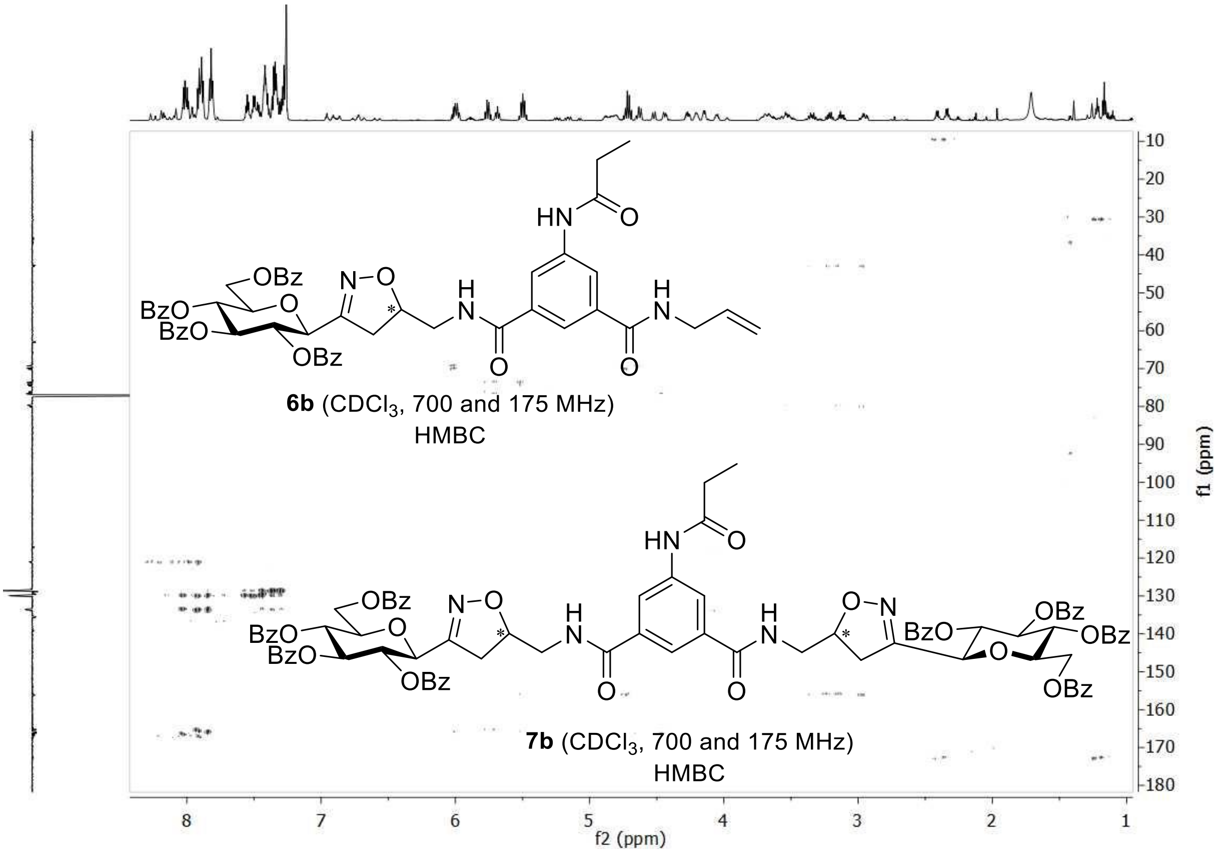

Figure S36. ^{1}H–^{13}C HMBC spectrum of **6b** and **7b**

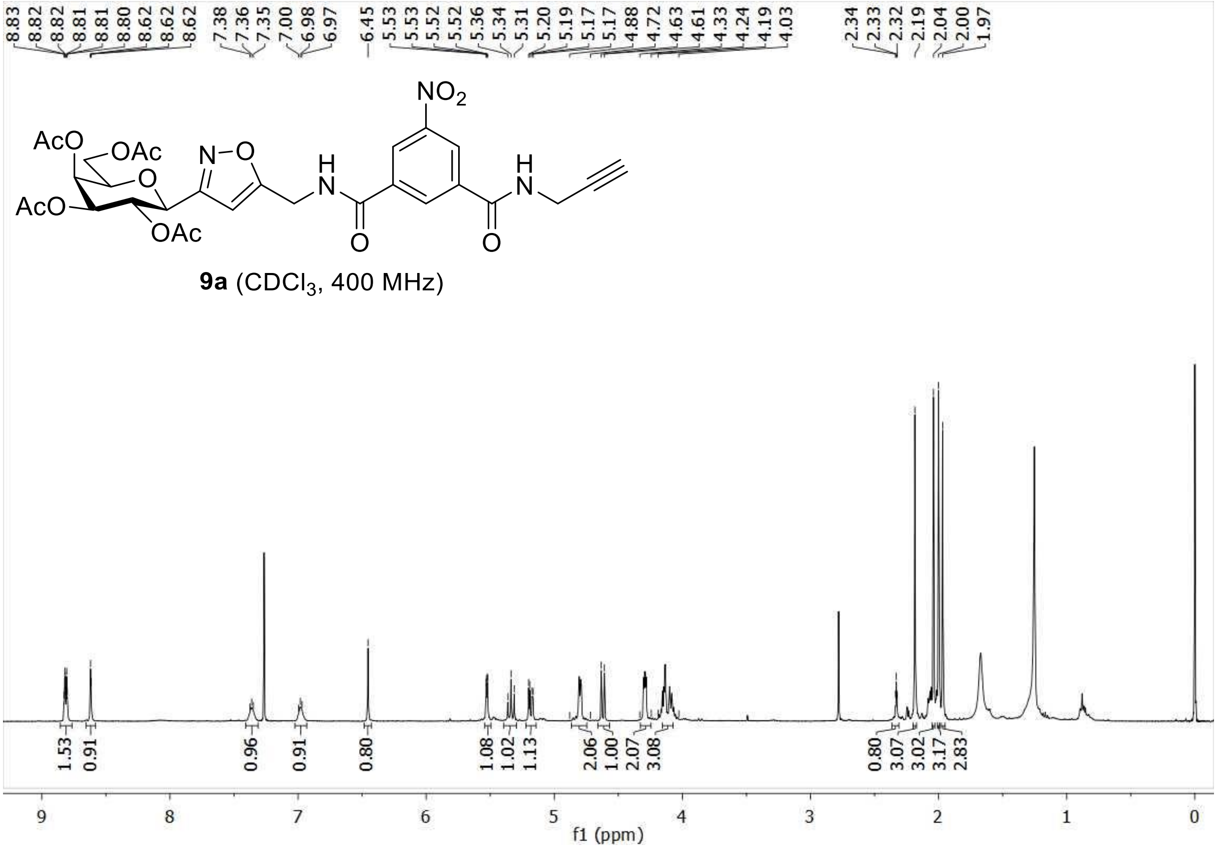

Figure S37. ^{1}H NMR spectrum of **9a**

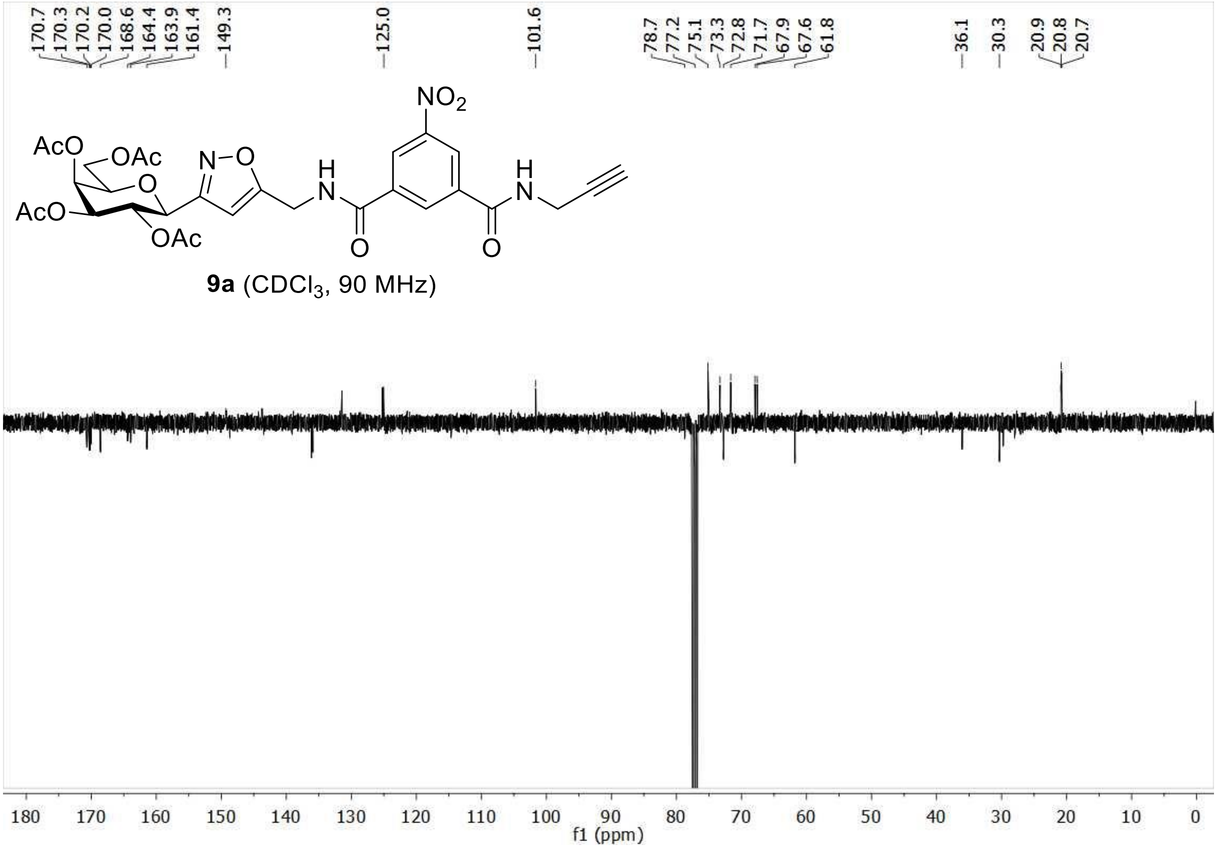

Figure S38. ^{13}C NMR spectrum of **9a**

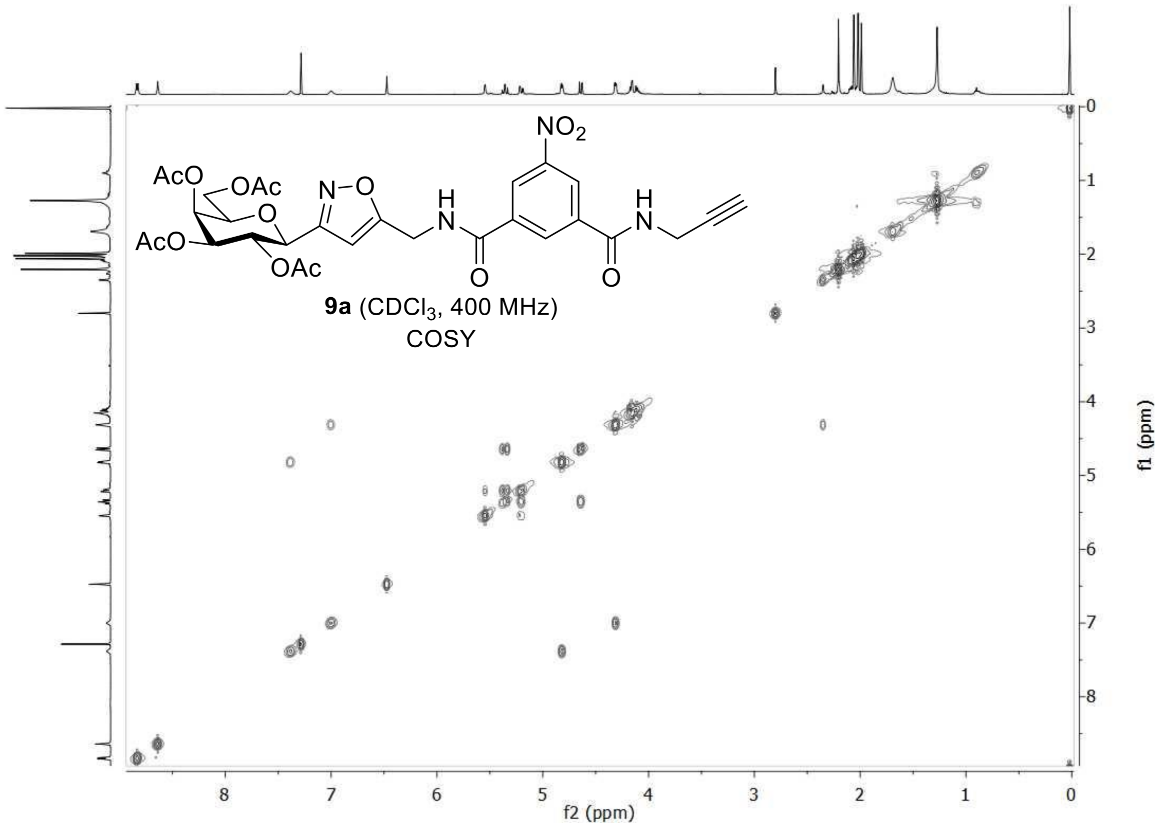

Figure S39. ^{1}H–^{1}H COSY spectrum of **9a**

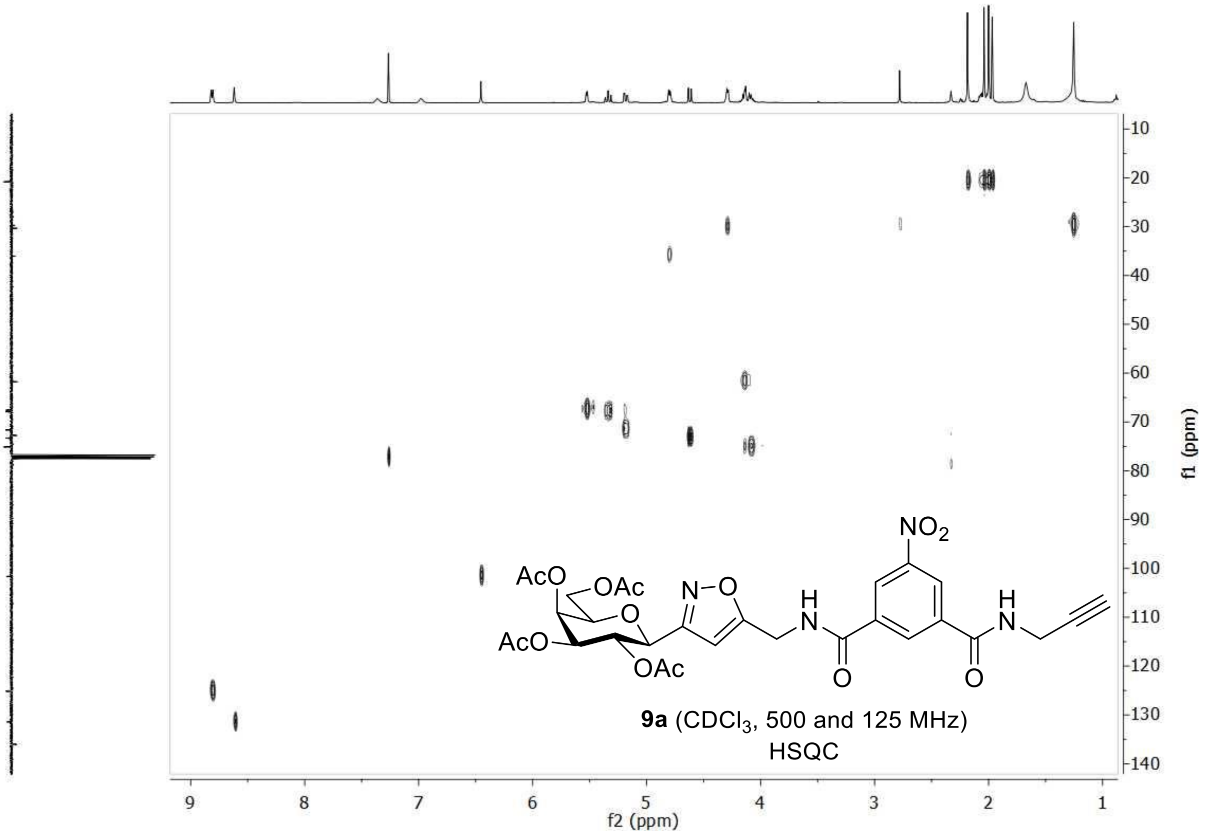

Figure S40. ^{1}H–^{13}C HSQC spectrum of **9a**

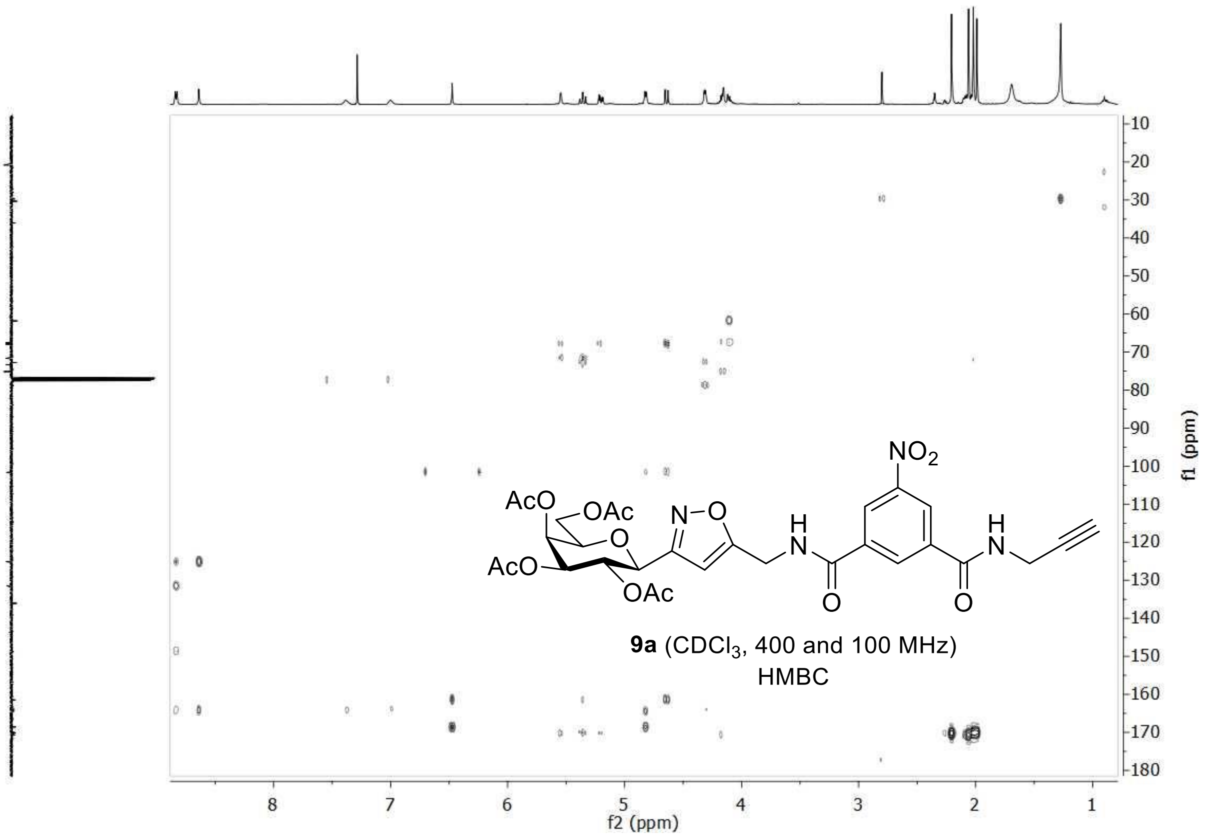

Figure S41. ^{1}H–^{13}C HMBC spectrum of **9a**

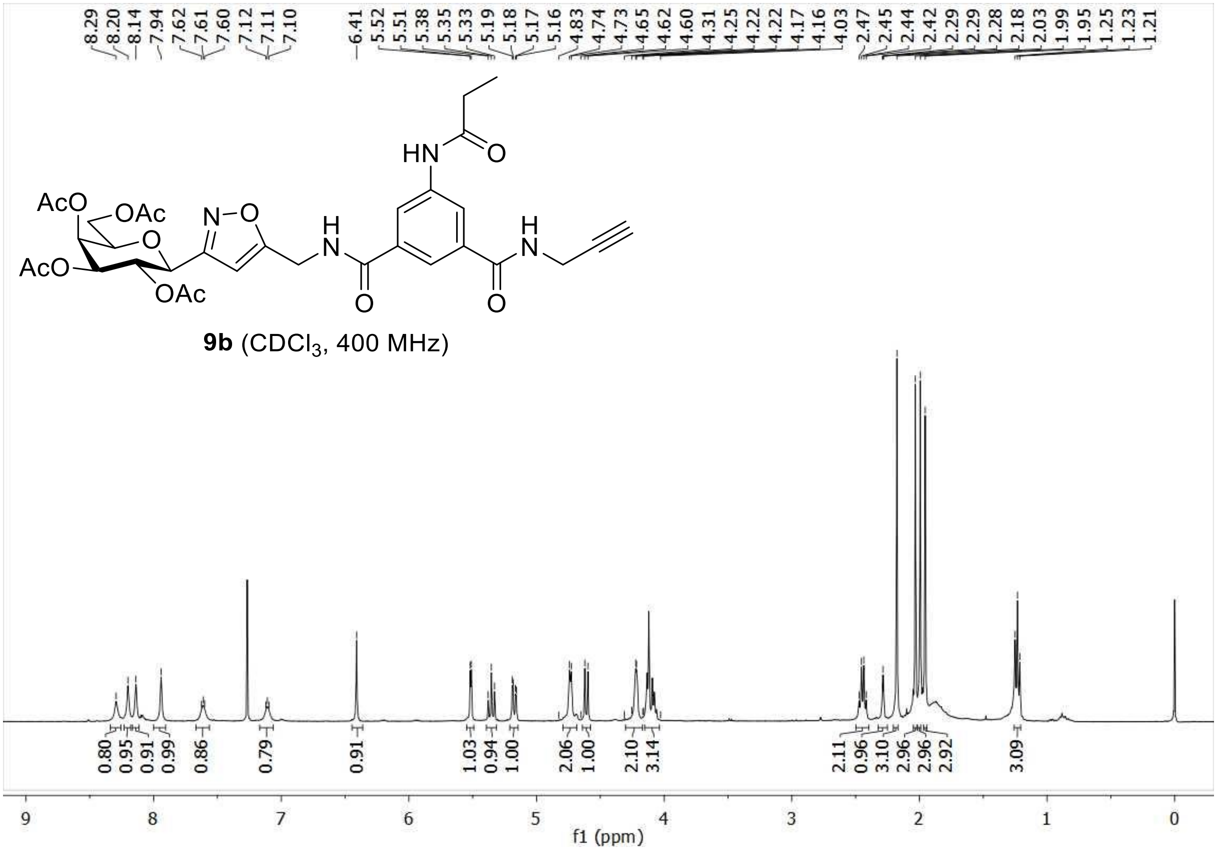

Figure S42. ^{1}H NMR spectrum of **9b**

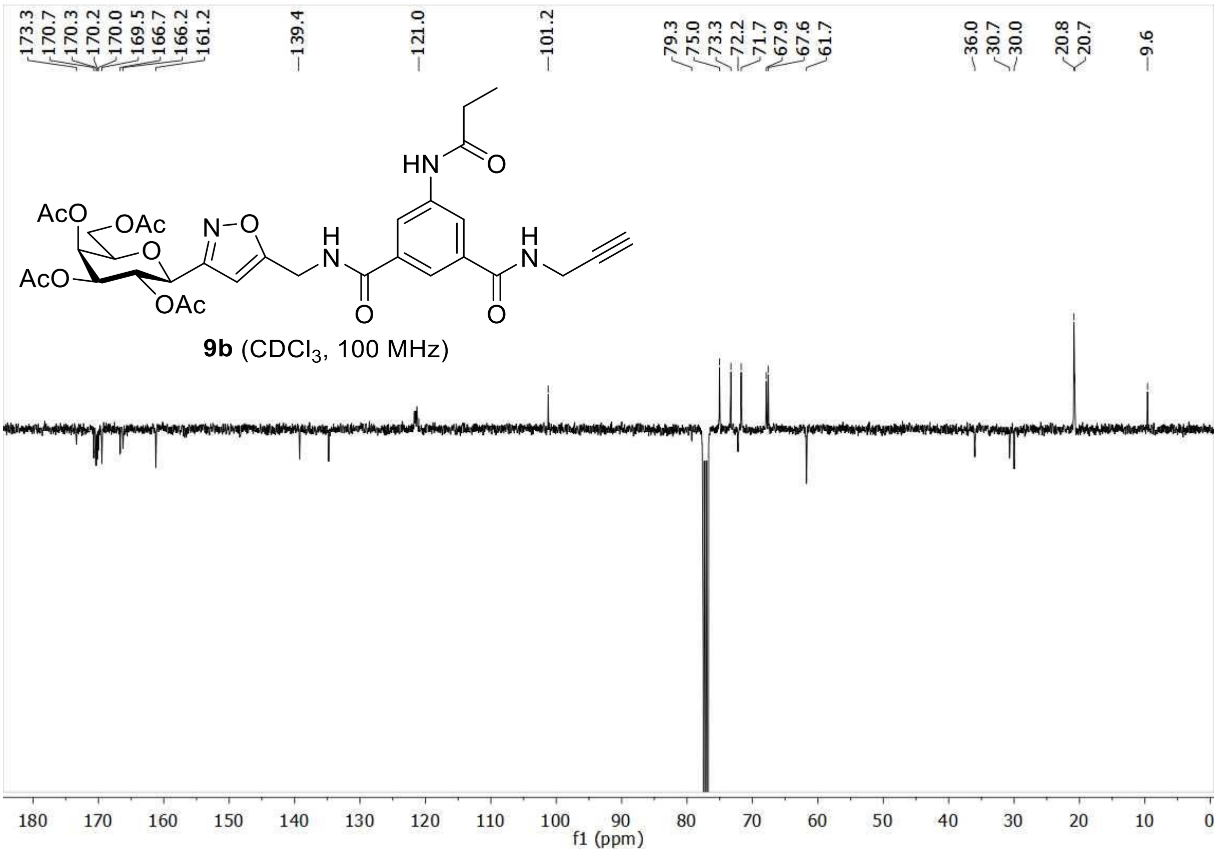

Figure S43. ^{13}C NMR spectrum of **9b**

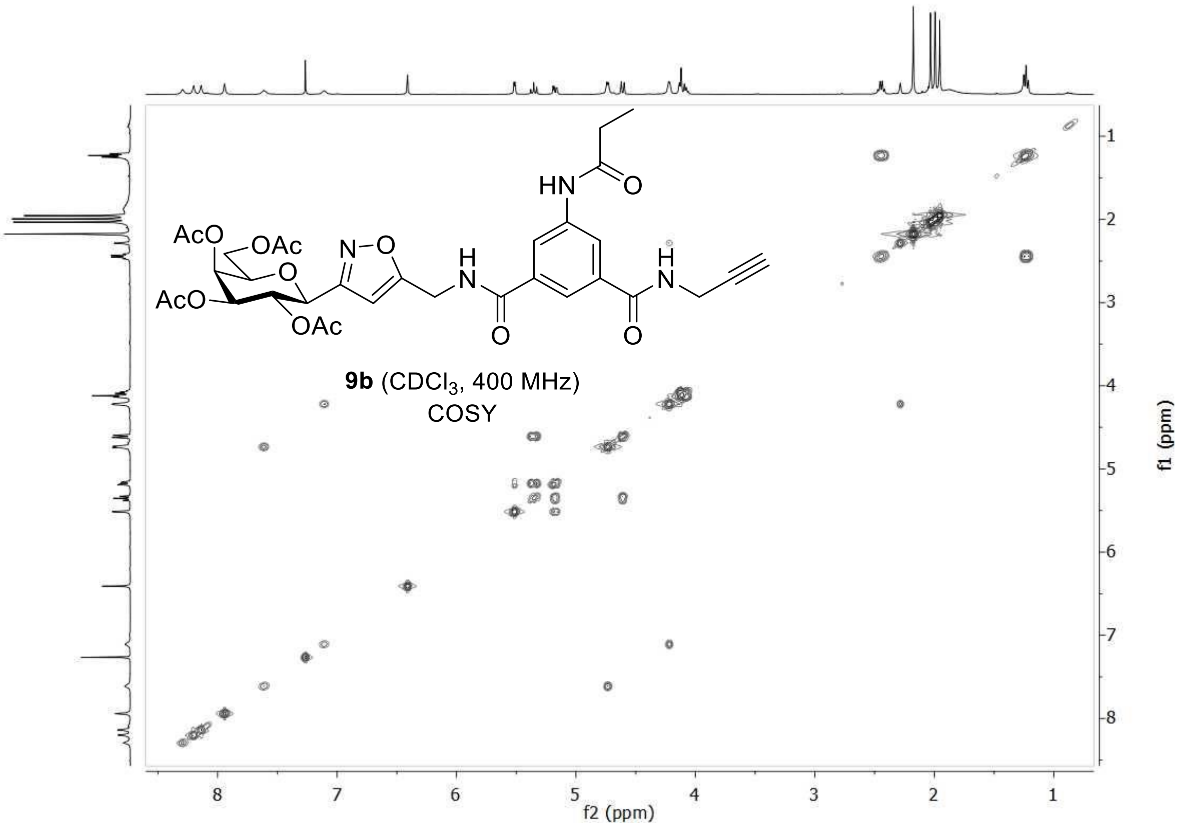

Figure S44. ^{1}H–^{1}H COSY spectrum of **9b**

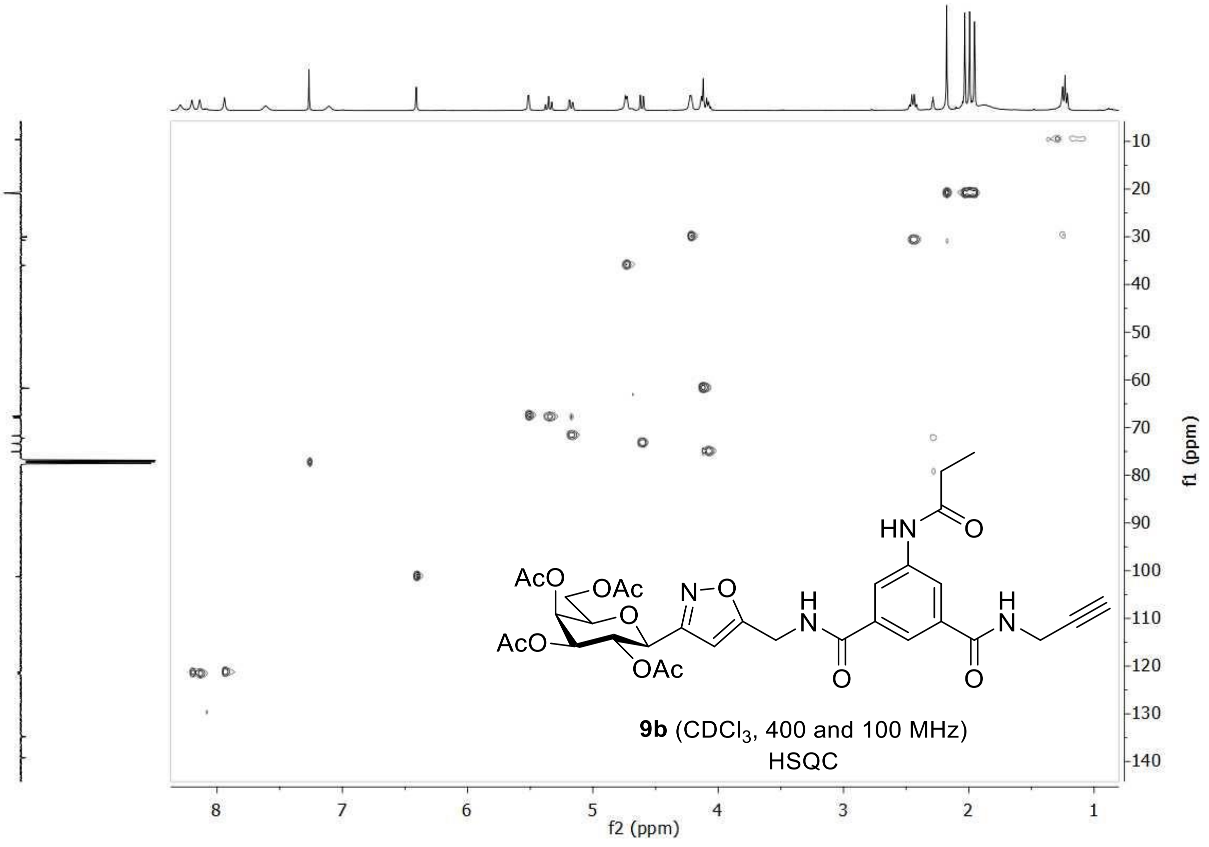

Figure S45. ^{1}H–^{13}C HSQC spectrum of **9b**

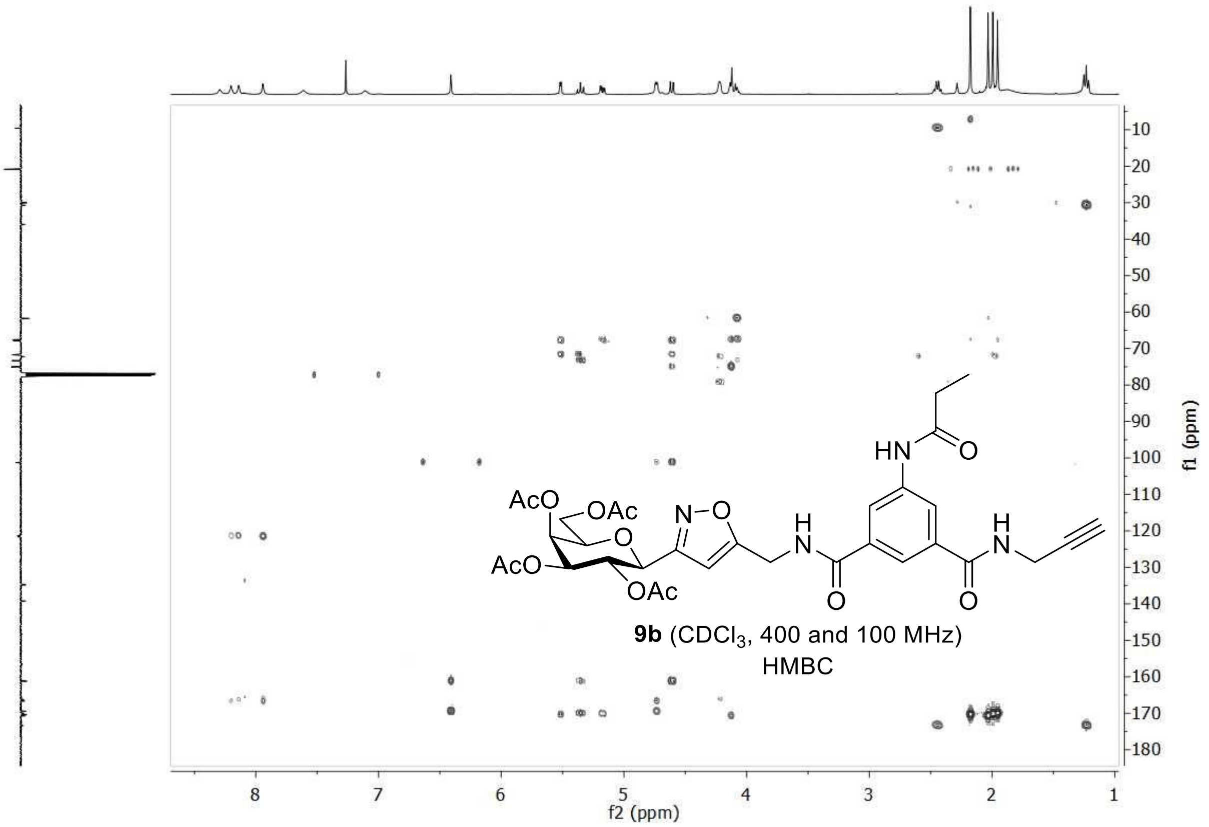

Figure S46. ^{1}H–^{13}C HMBC spectrum of **9b**

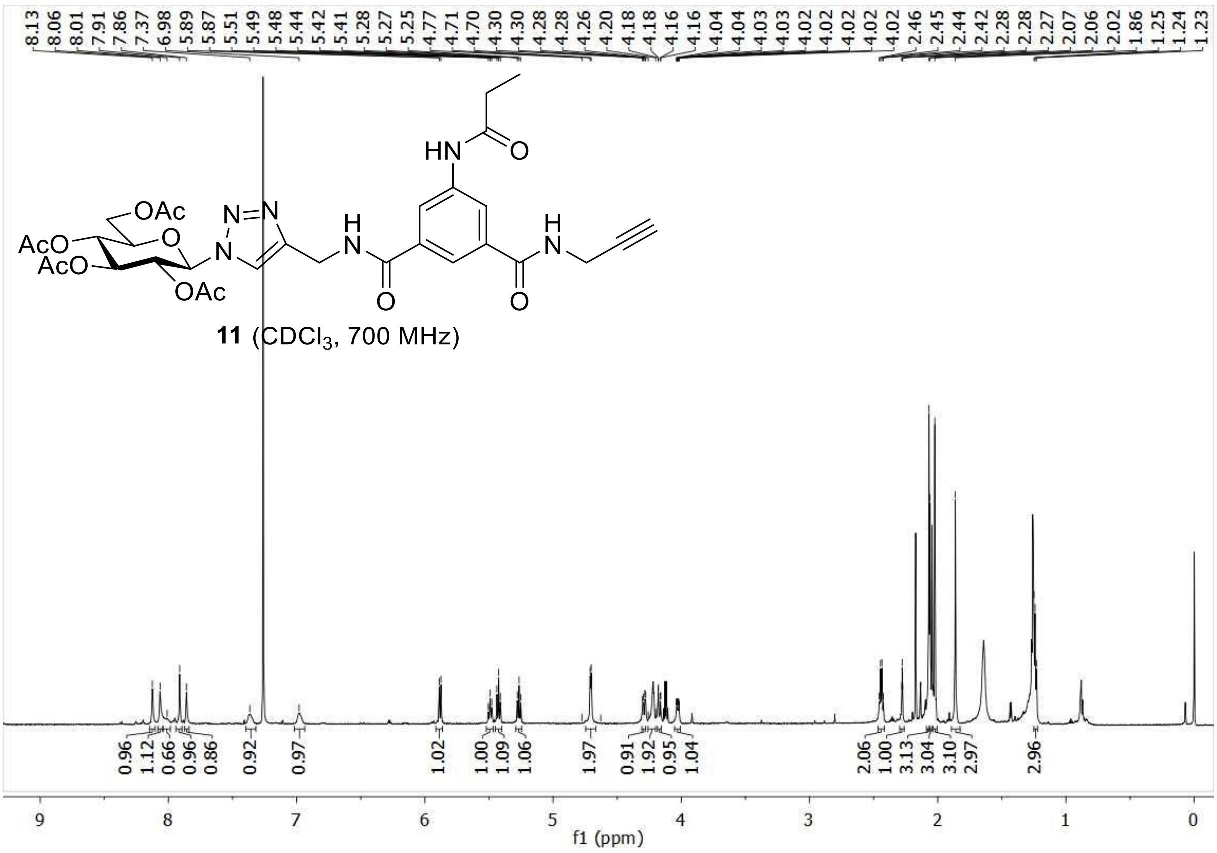

Figure S47. ^{1}H NMR spectrum of **11**

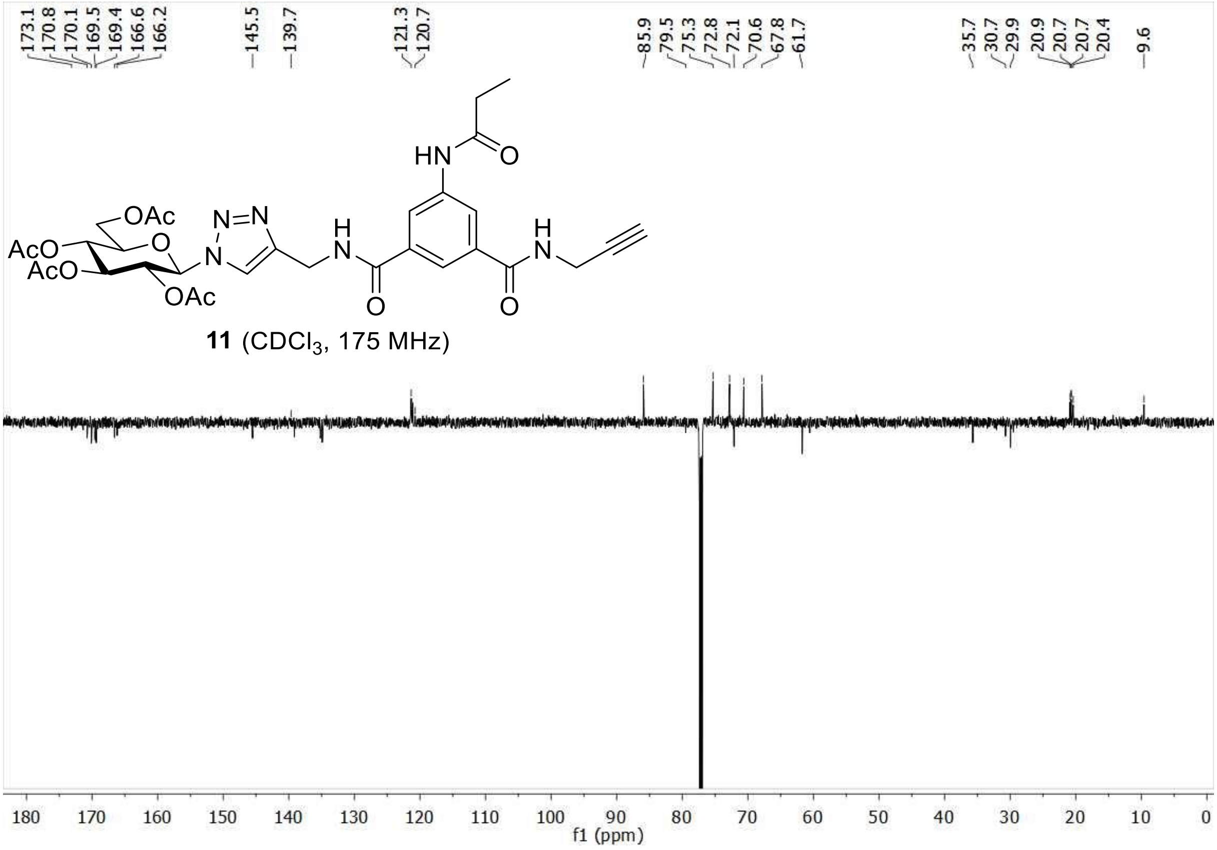

Figure S48. ^{13}C NMR spectrum of **11**

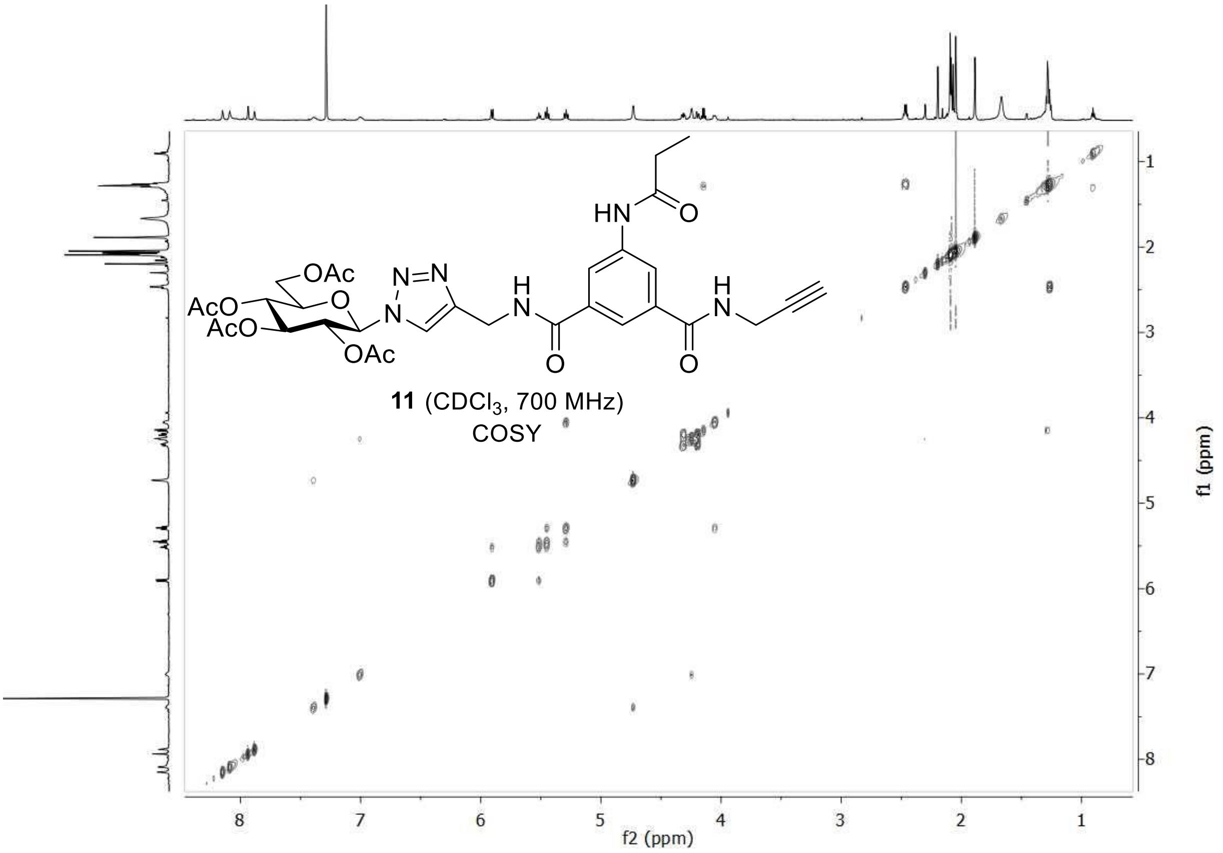

Figure S49. ^{1}H–^{1}H COSY spectrum of **11**

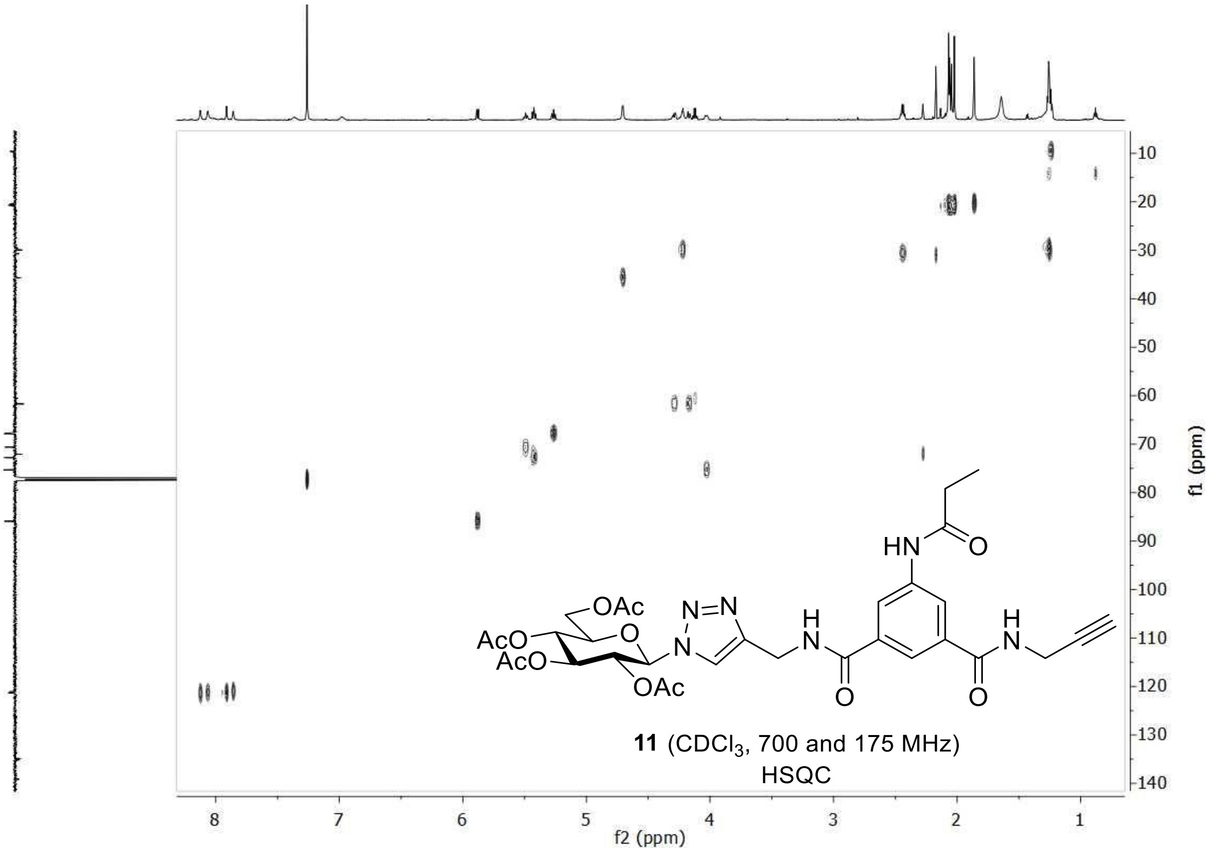

Figure S50. ^{1}H–^{13}C HSQC spectrum of **11**

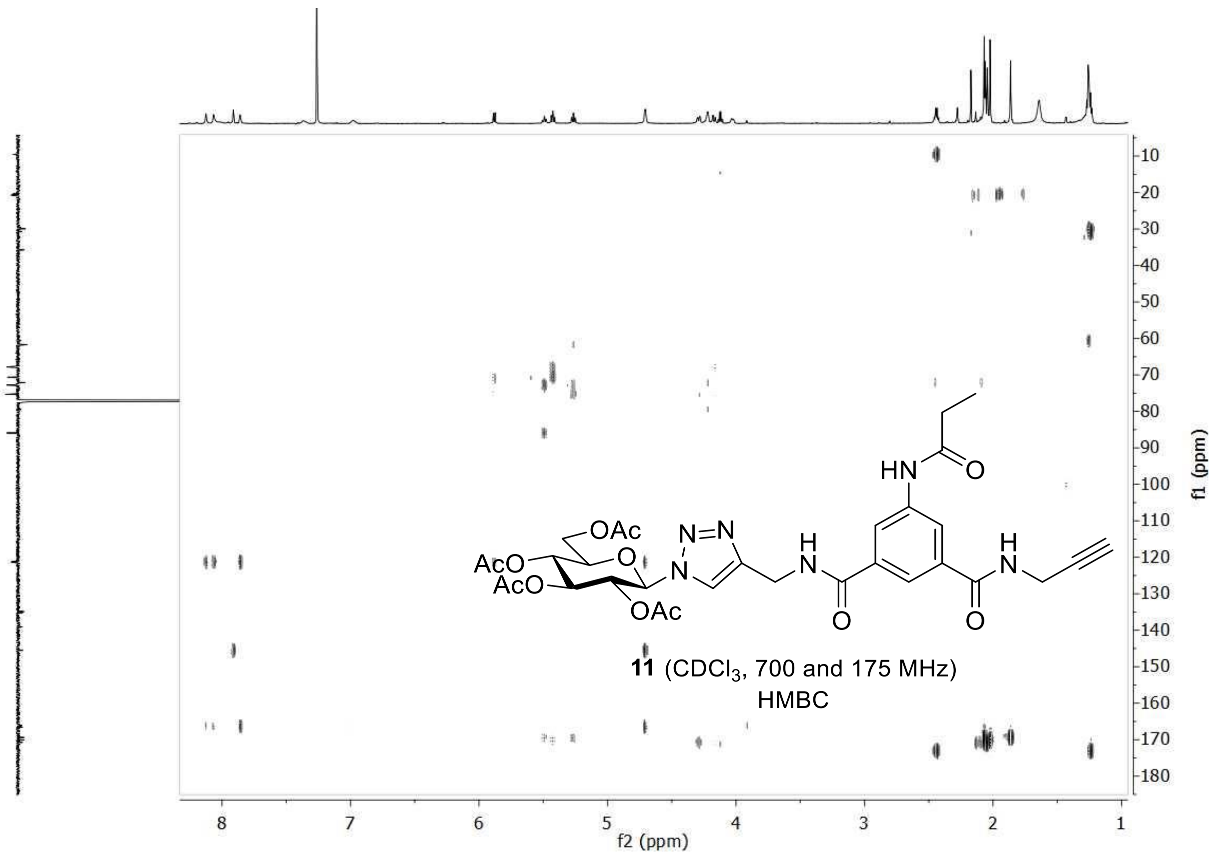

Figure S51. ^{1}H–^{13}C HMBC spectrum of **11**

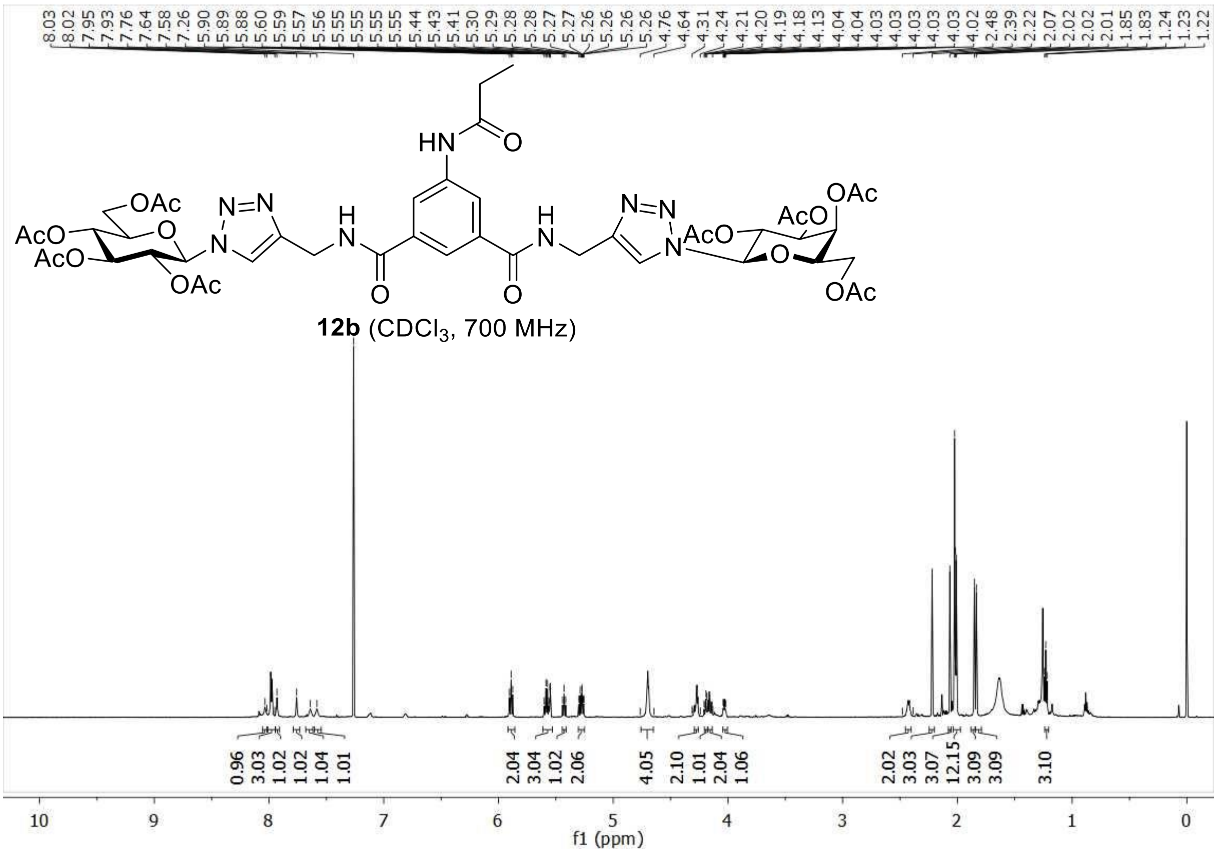

Figure S52. ^{1}H NMR spectrum of **12b**

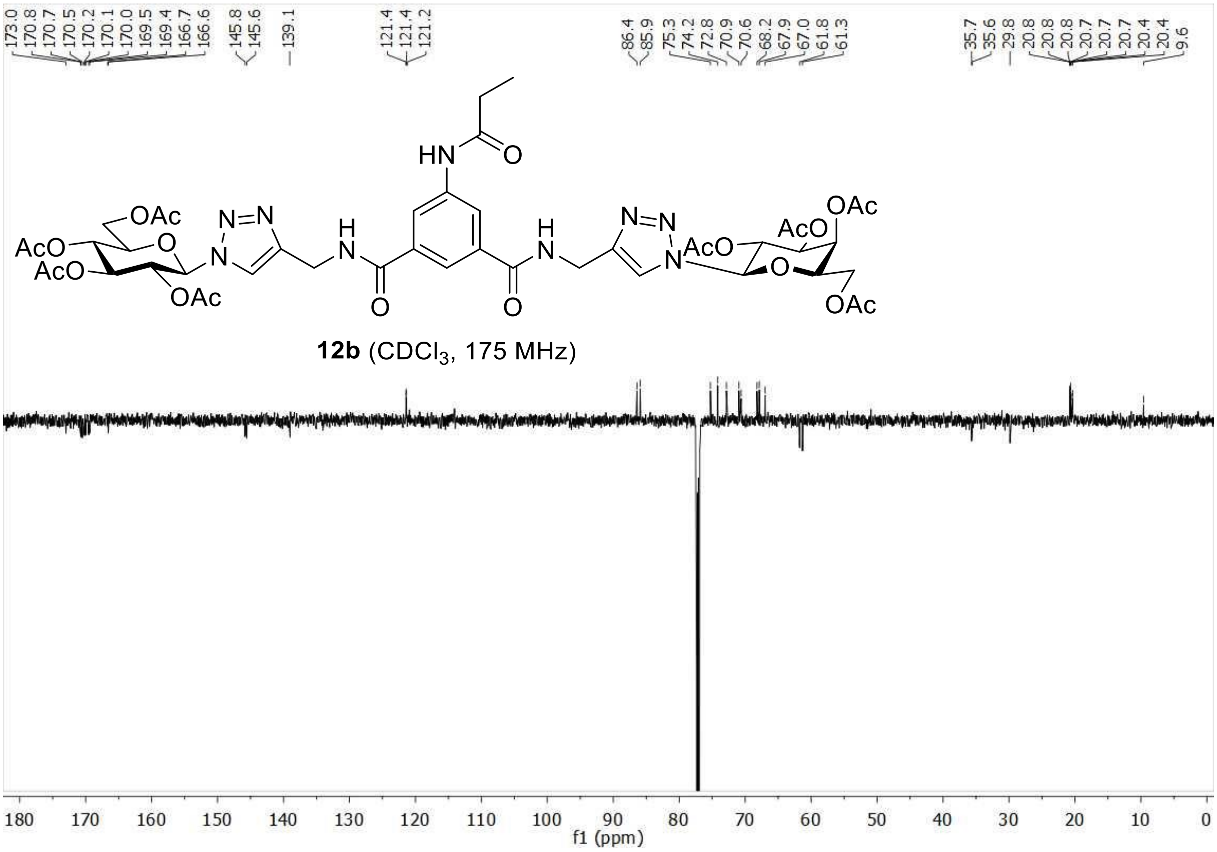

Figure S53. ^{13}C NMR spectrum of **12b**

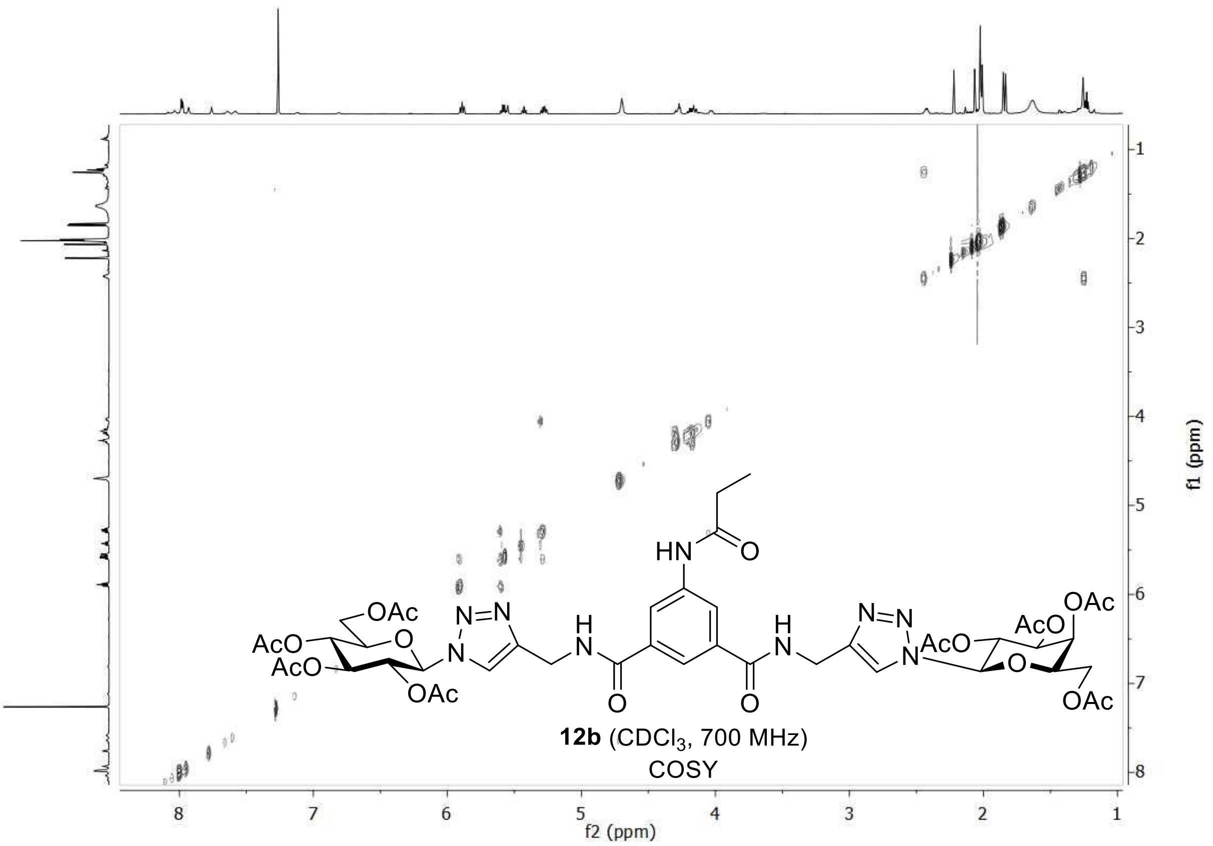

Figure S54. ^{1}H–^{1}H COSY spectrum of **12b**

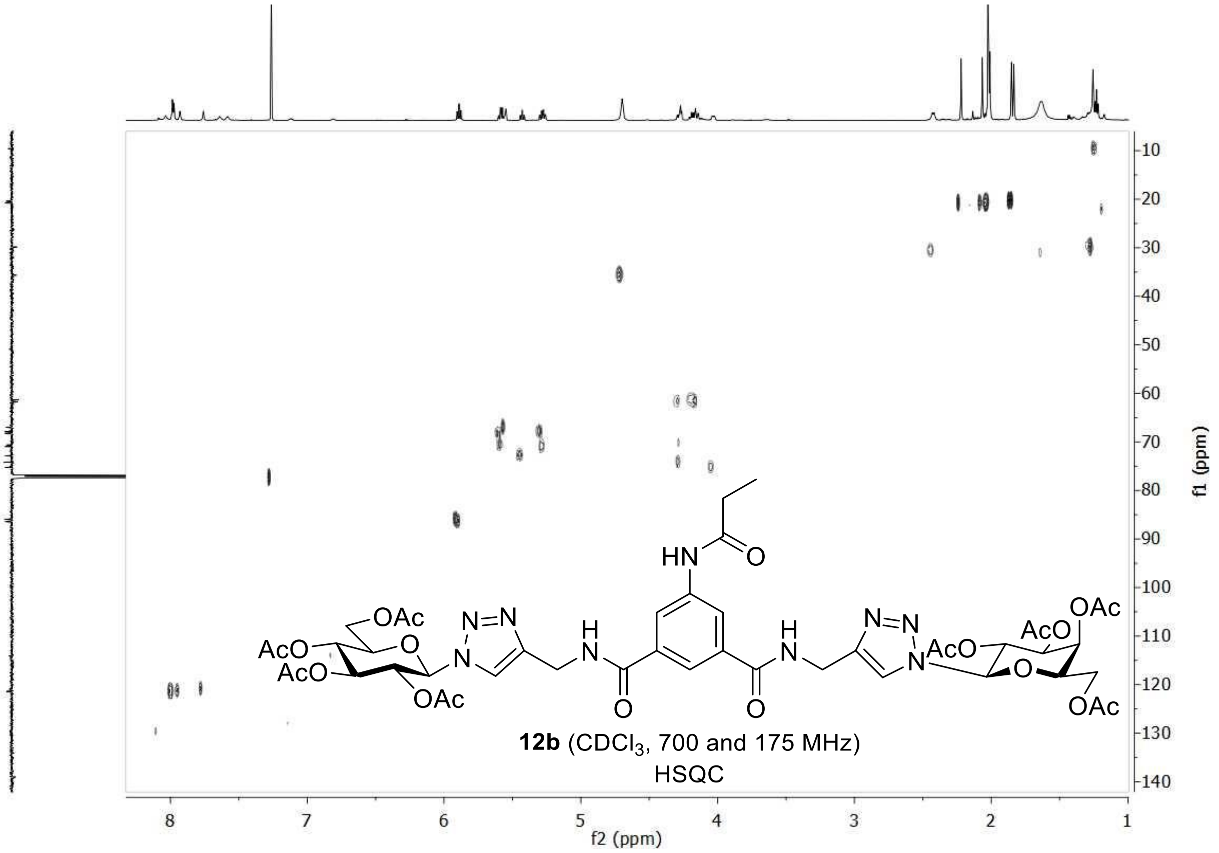

Figure S55. ^{1}H–^{13}C HSQC spectrum of **12b**

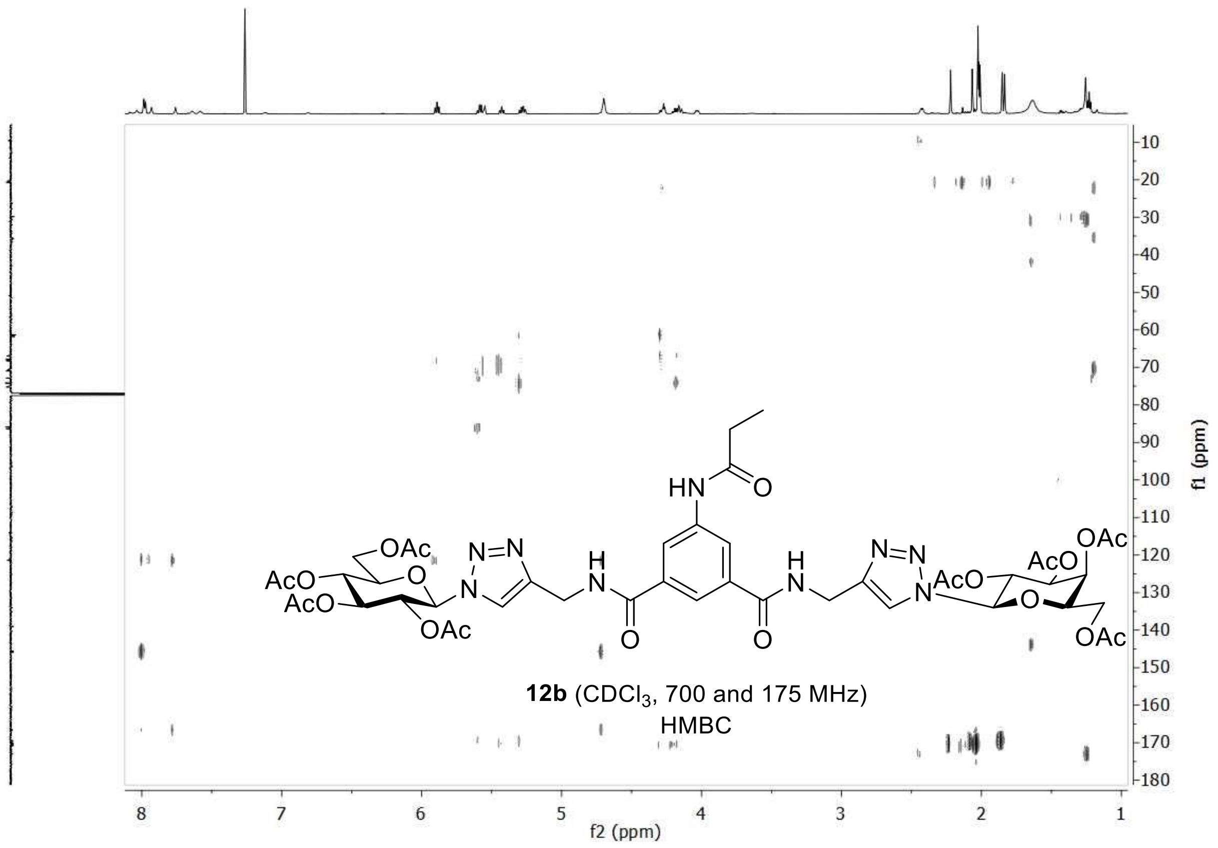

Figure S56. ^{1}H–^{13}C HMBC spectrum of **12b**

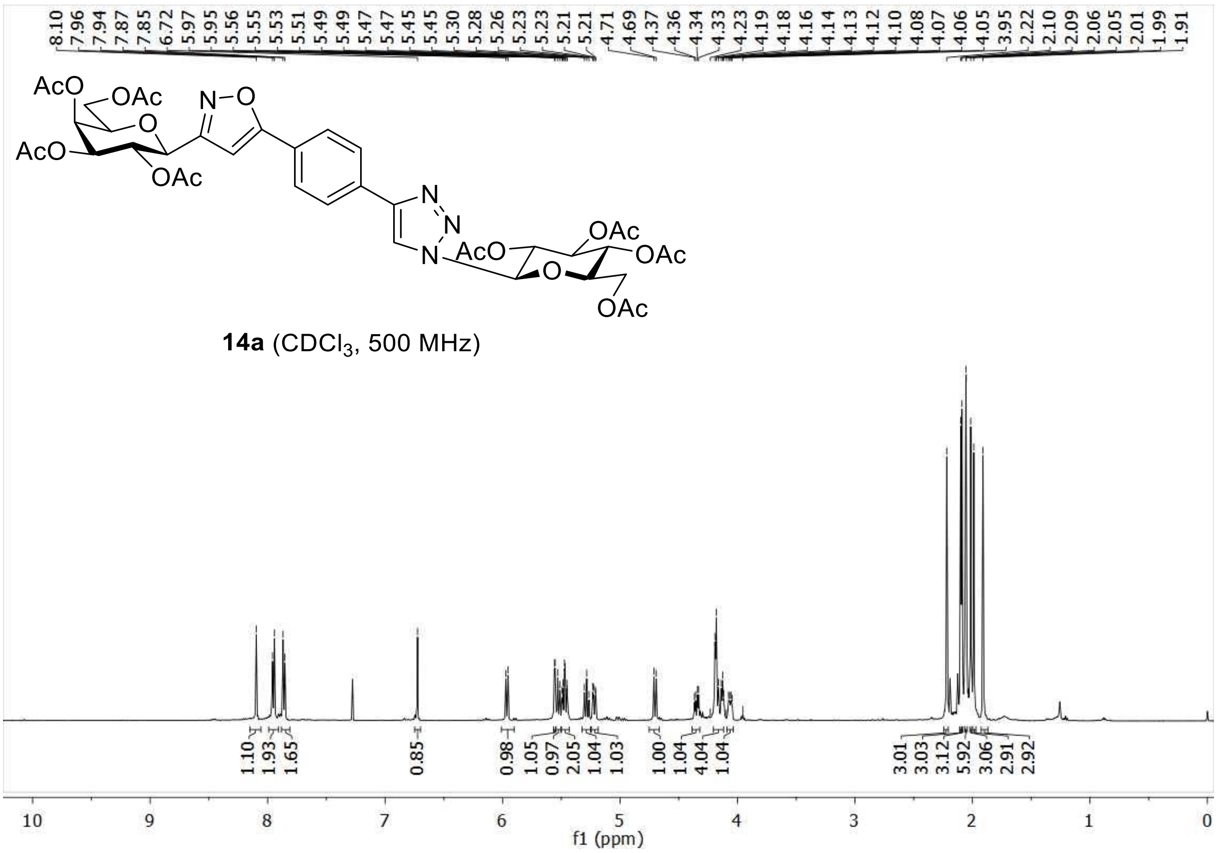

Figure S57. ^{1}H NMR spectrum of **14a**

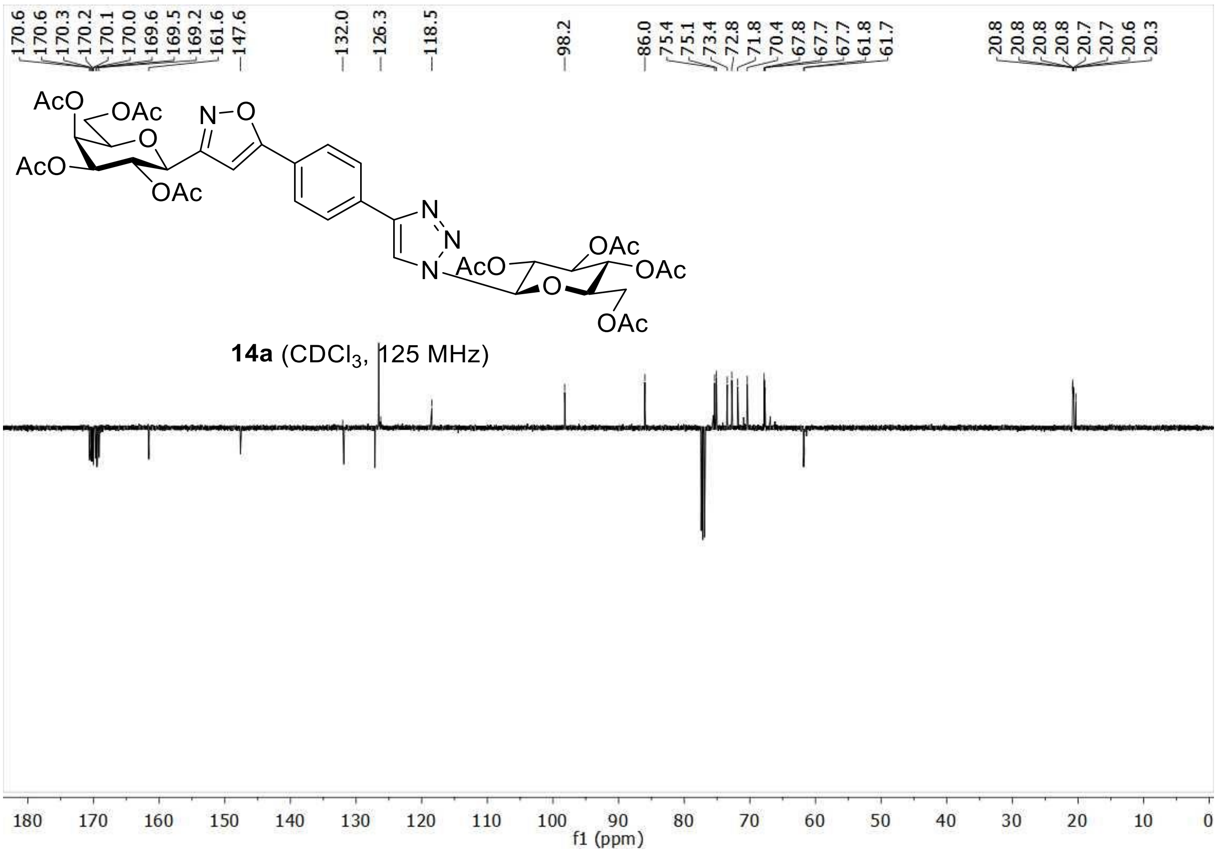

Figure S58. ^{13}C NMR spectrum of **14a**

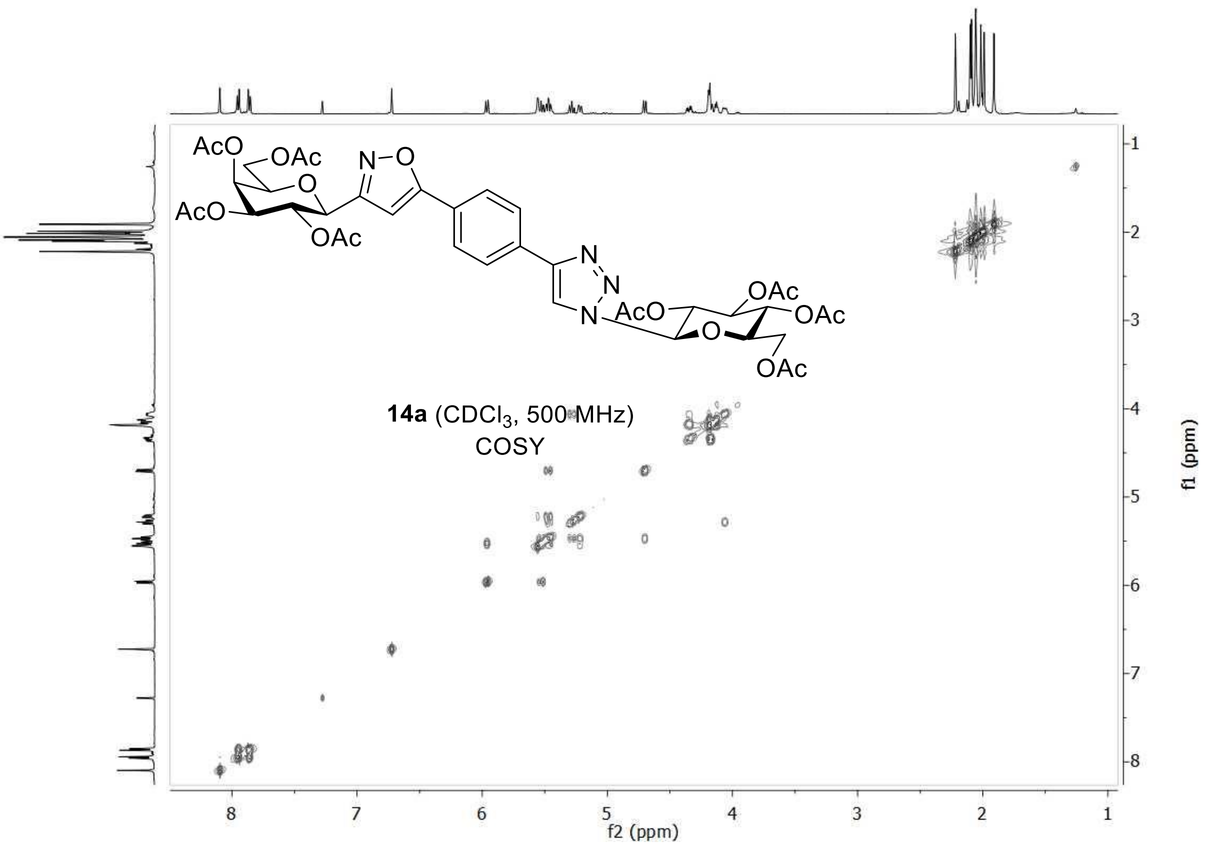

Figure S59. ^{1}H–^{1}H COSY spectrum of **14a**

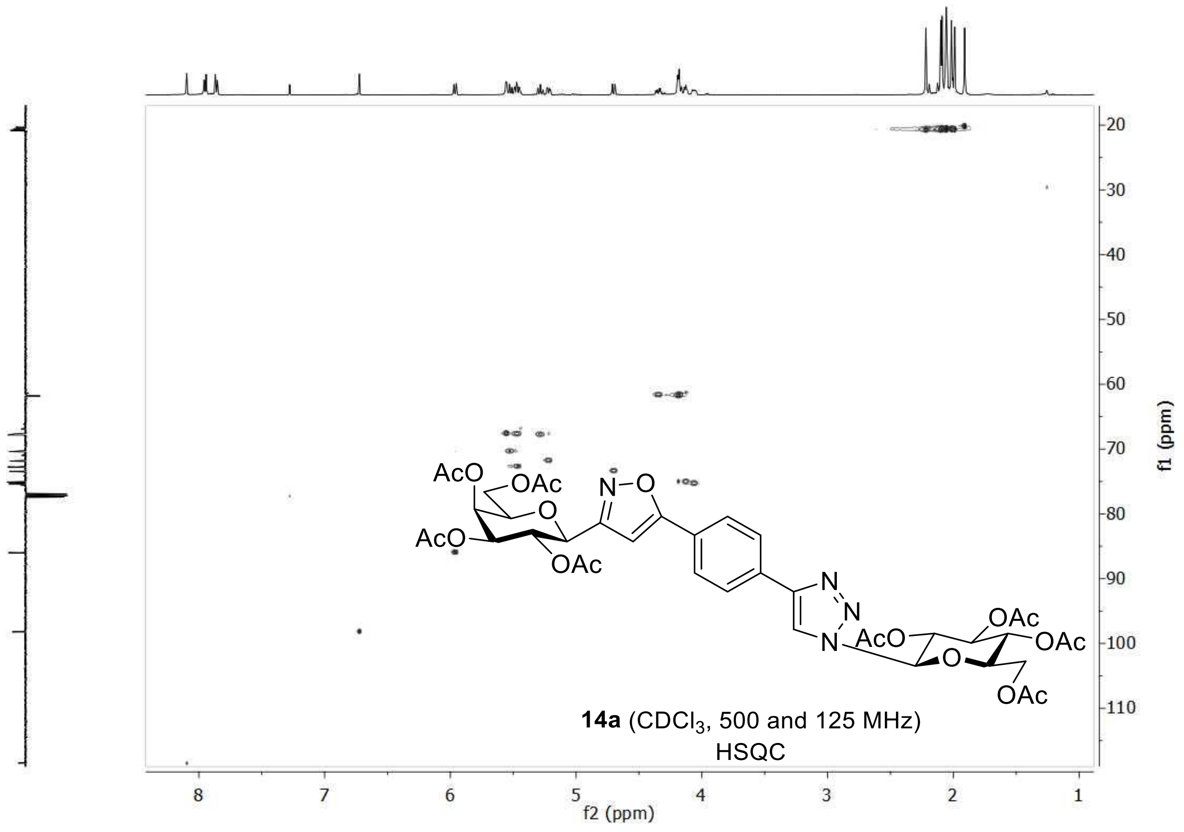

Figure S60. ^{1}H–^{13}C HSQC spectrum of **14a**

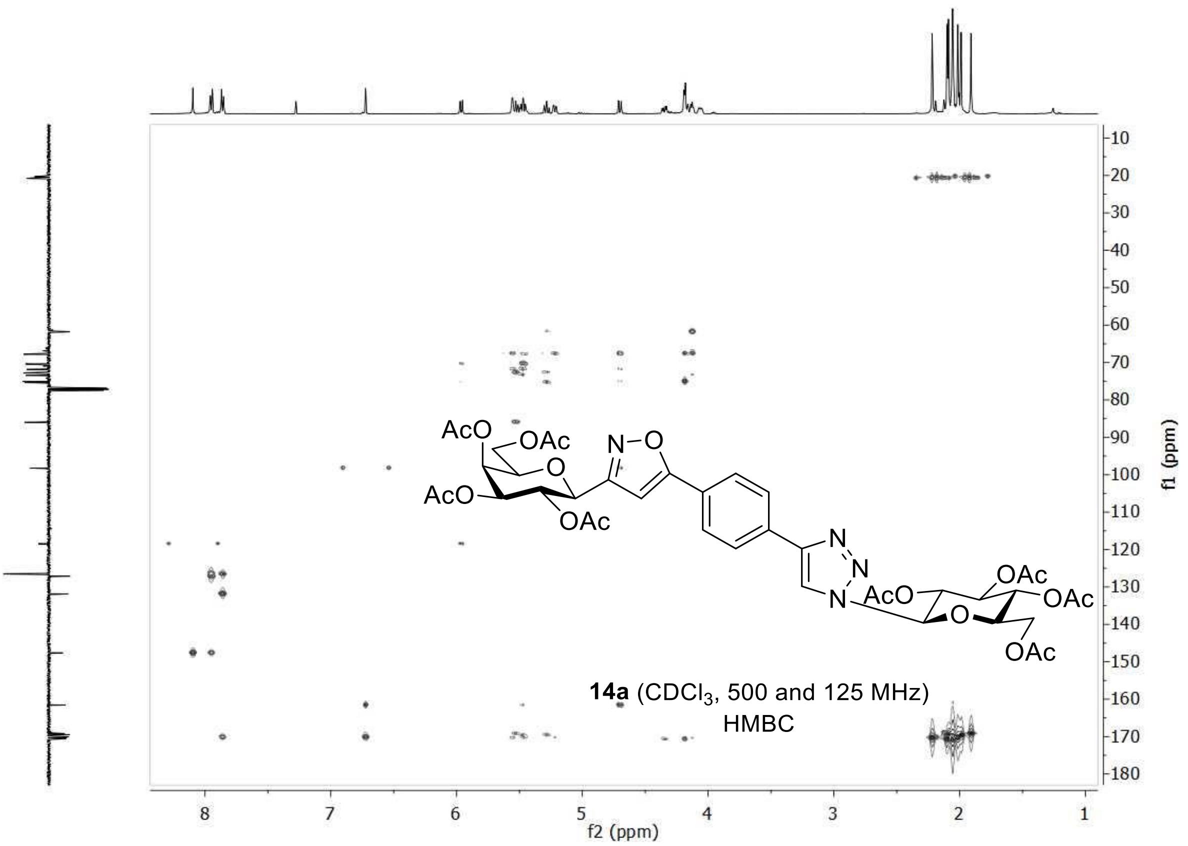

Figure S61. ^{1}H–^{13}C HMBC spectrum of **14a**

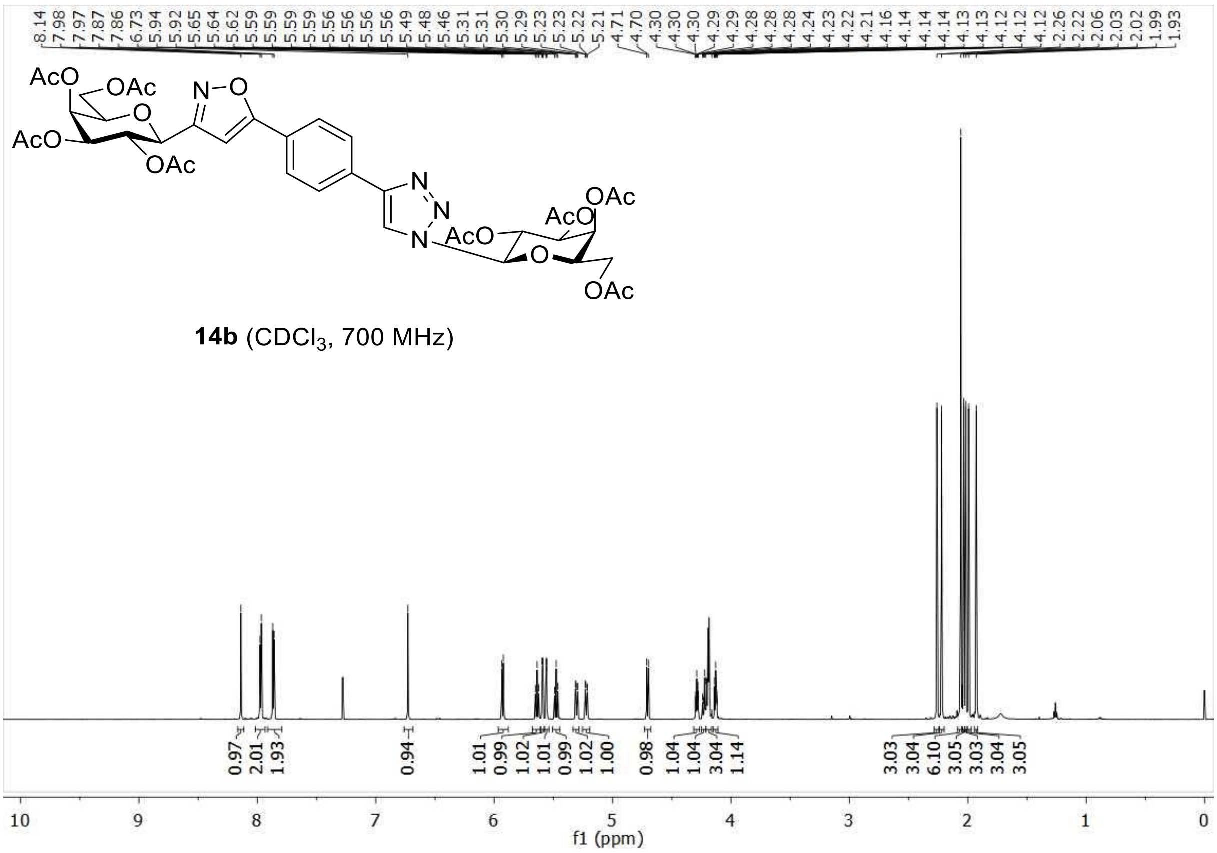

Figure S62. ^{1}H NMR spectrum of **14b**

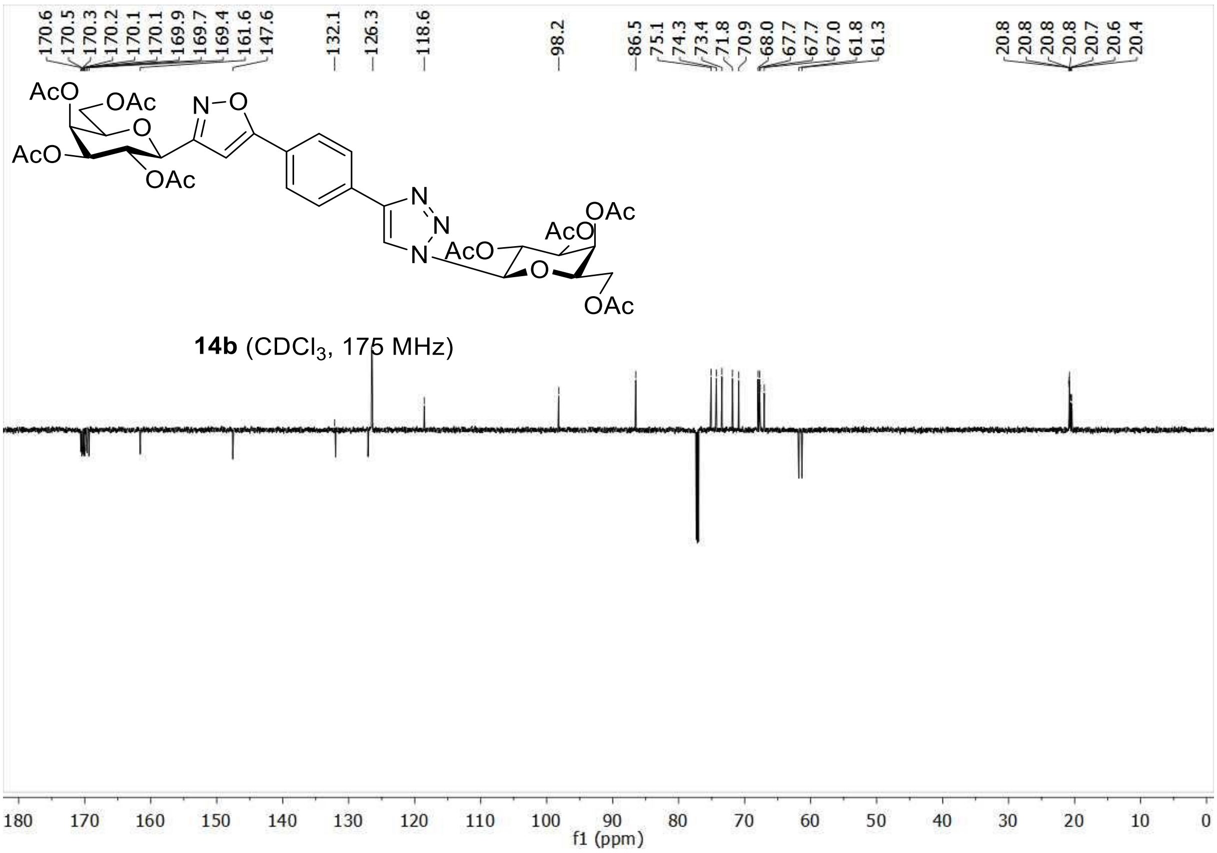

Figure S63. ^{13}C NMR spectrum of **14b**

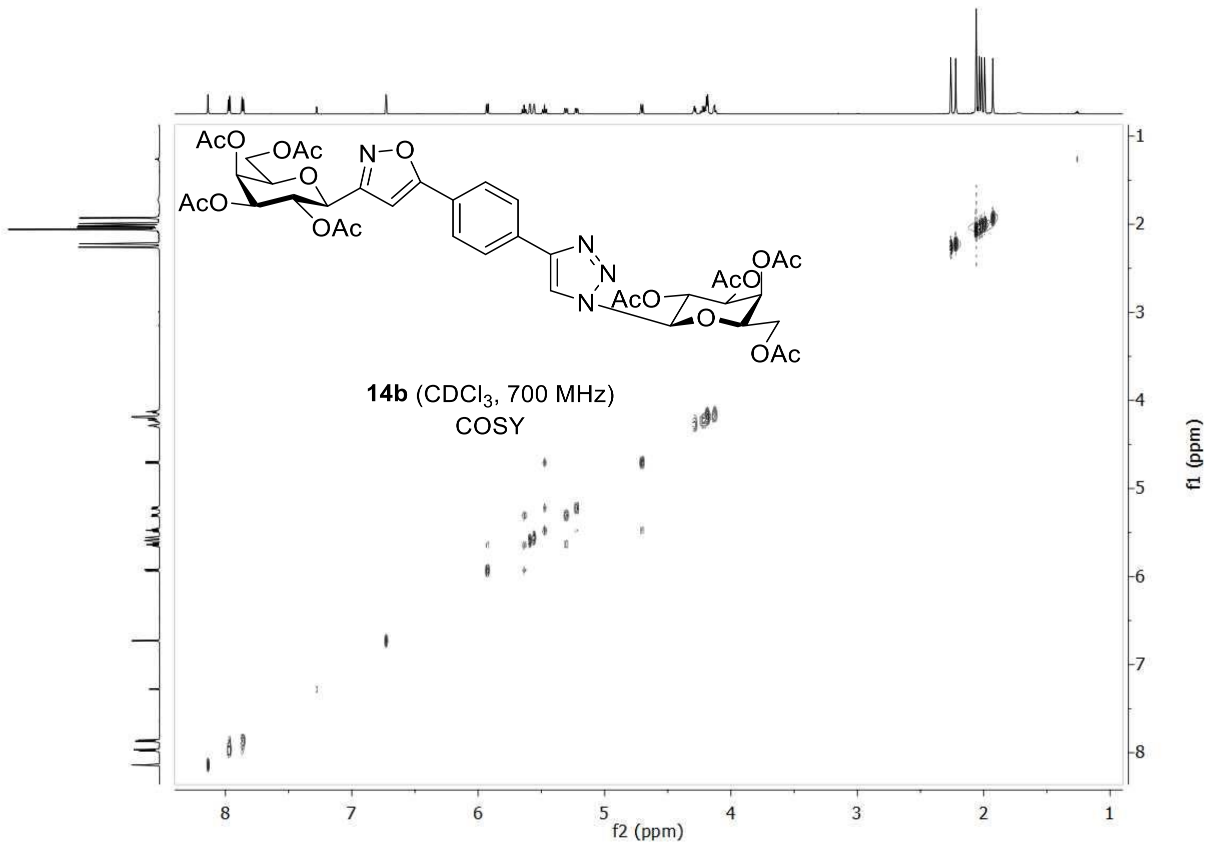

Figure S64. ^{1}H–^{1}H COSY spectrum of **14b**

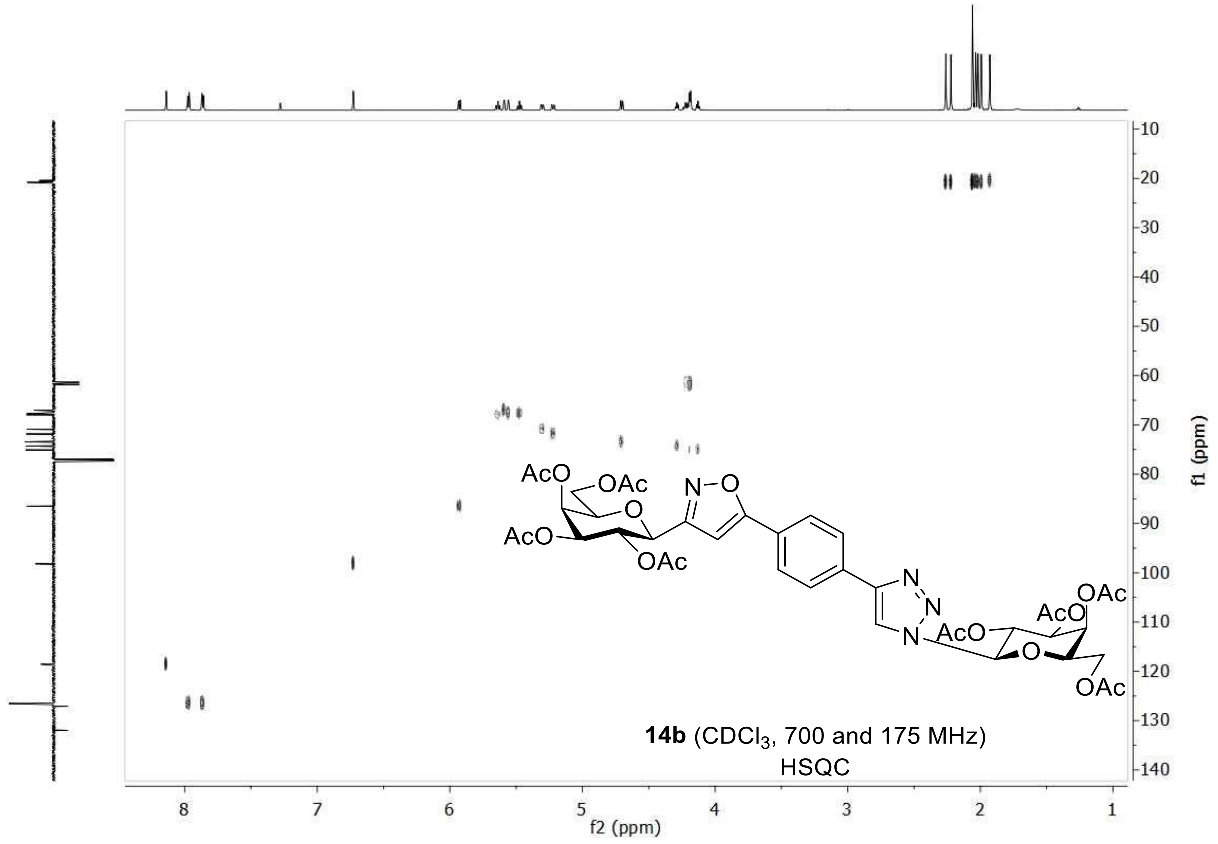

Figure S65. ^{1}H–^{13}C HSQC spectrum of **14b**

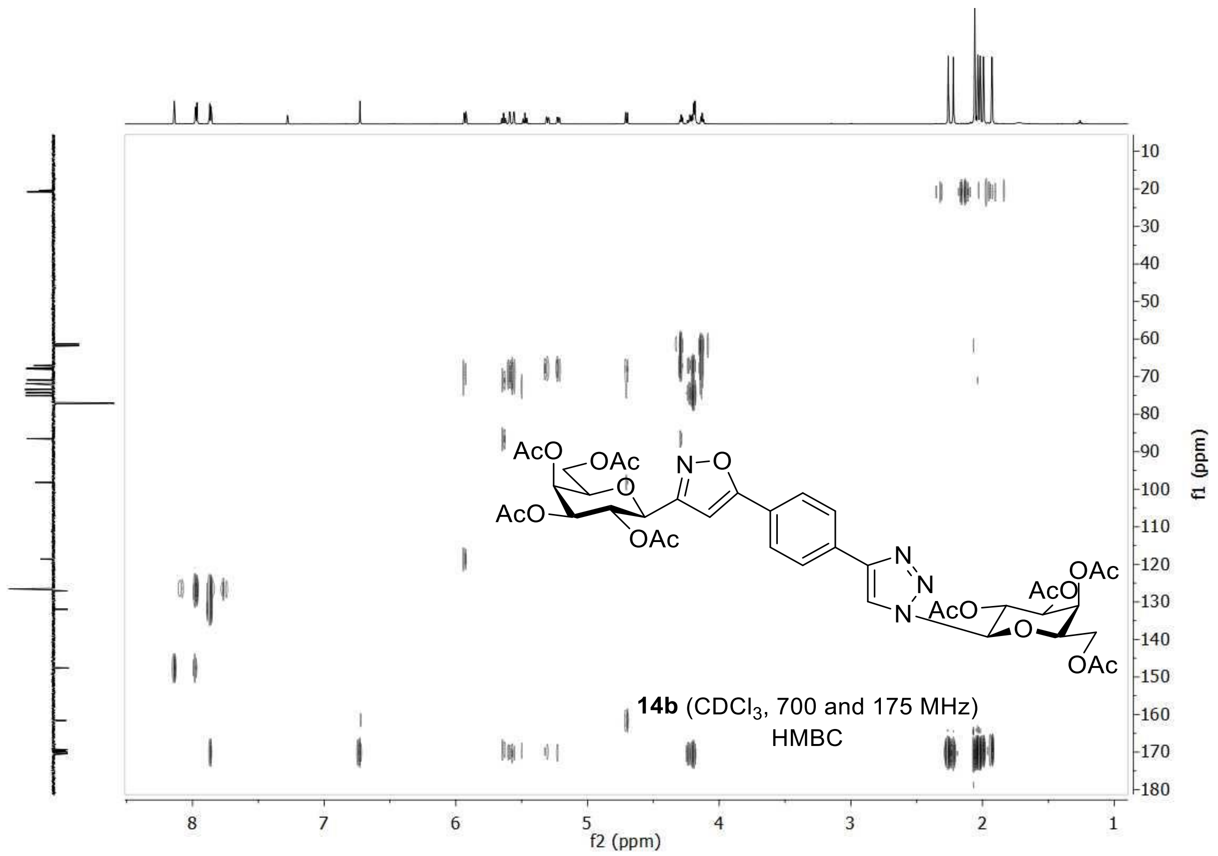

Figure S66. $^{1}H–^{13}C$ HMBC spectrum of **14b**

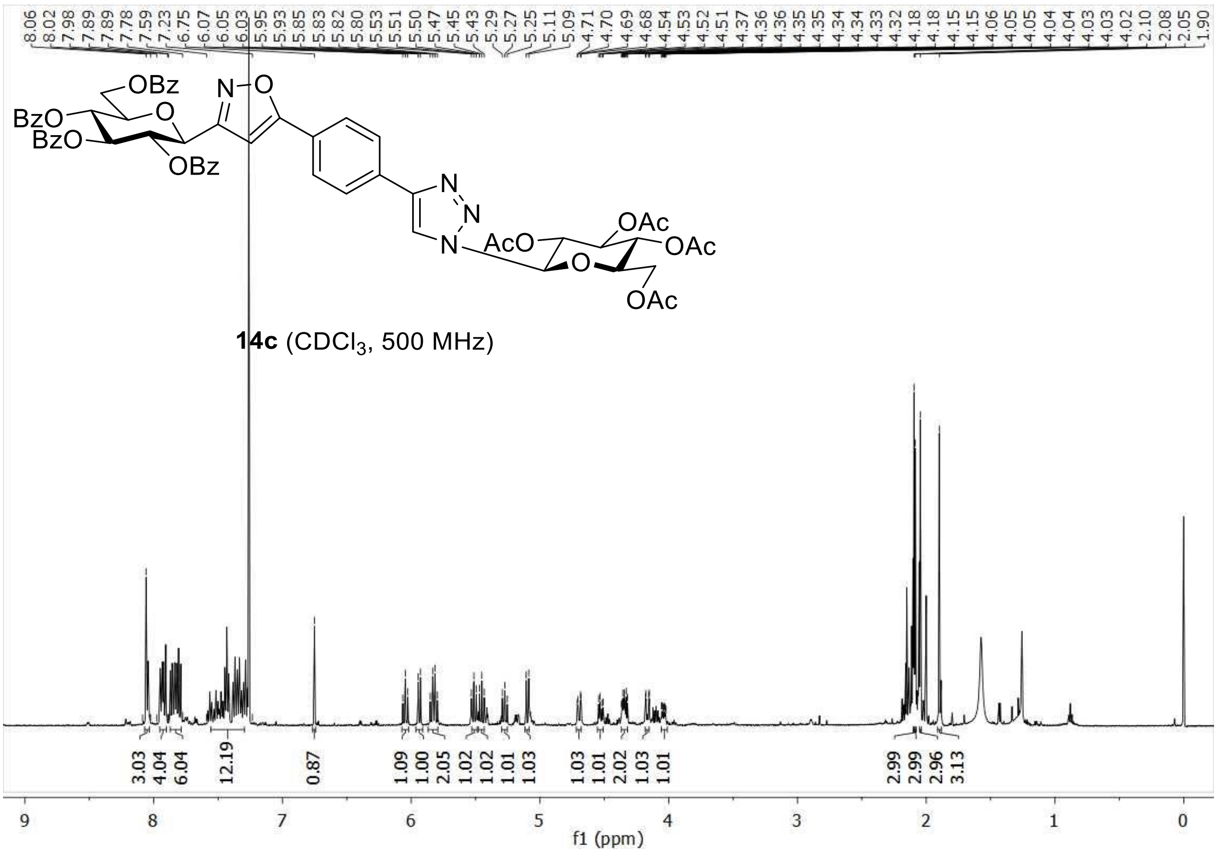

Figure S67. ^{1}H NMR spectrum of **14c**

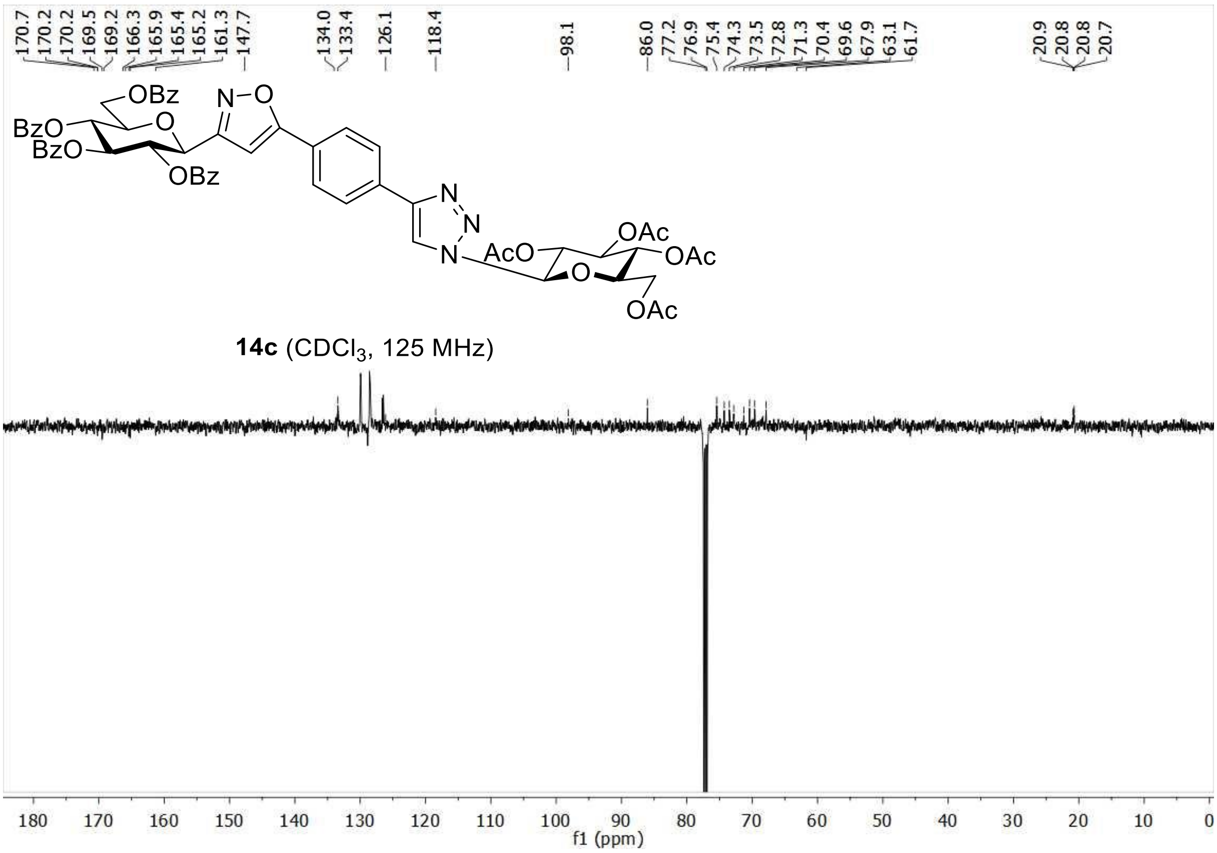

Figure S68. ^{13}C NMR spectrum of **14c**

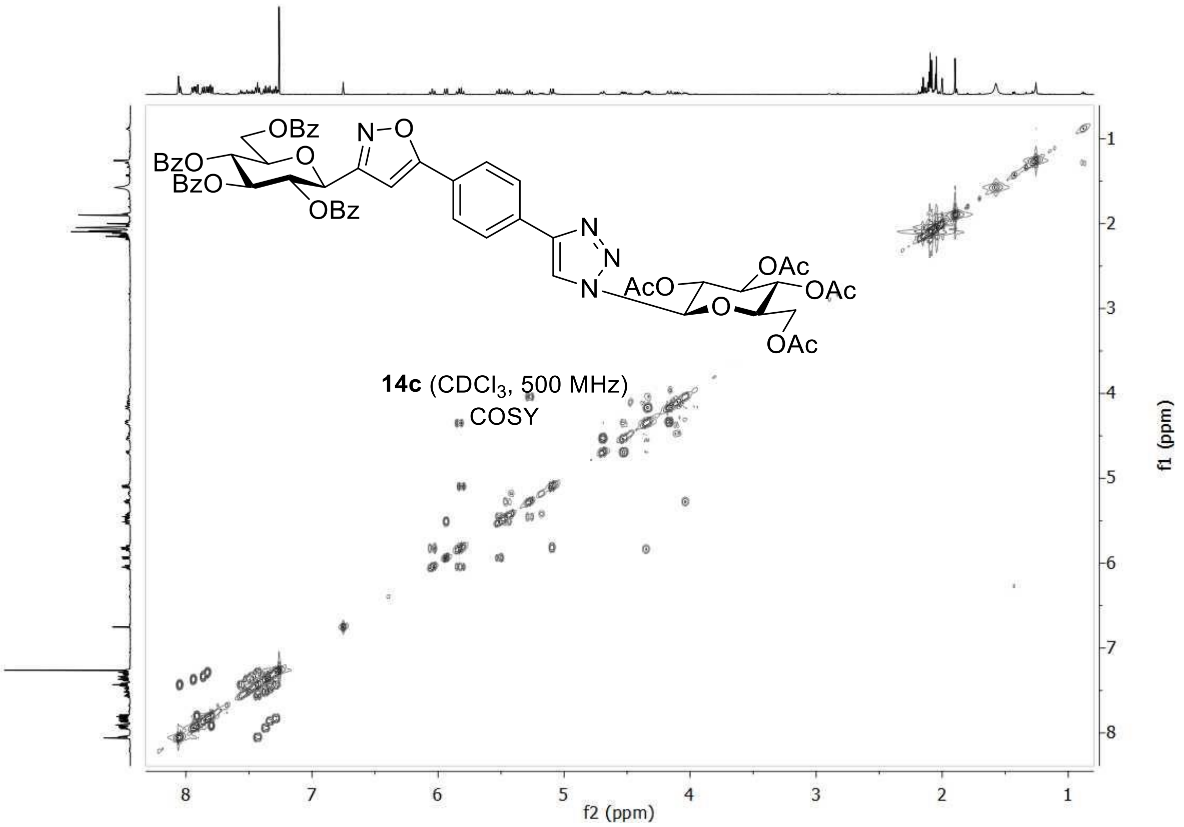

Figure S69. ${}^{1}H$–${}^{1}H$ COSY spectrum of **14c**

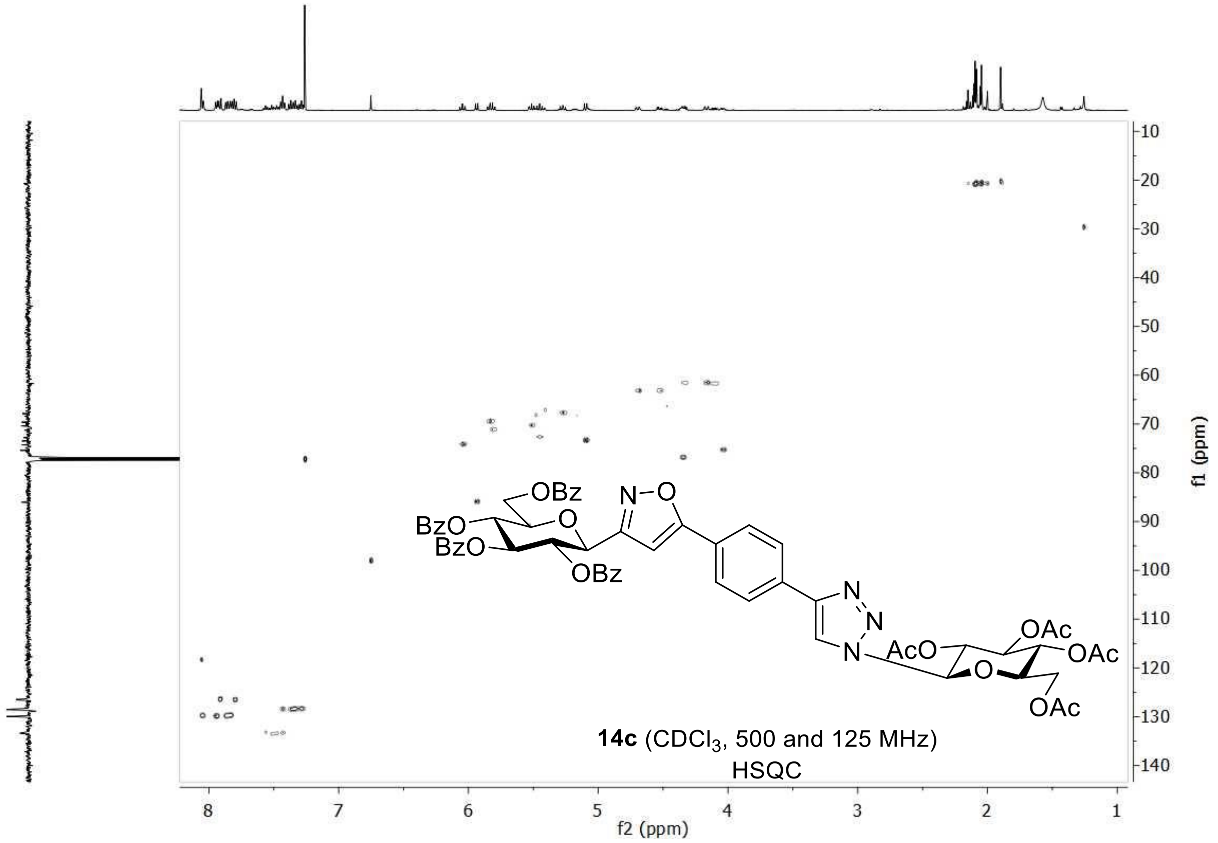

Figure S70. ${}^{1}H$–${}^{13}C$ HSQC spectrum of **14c**

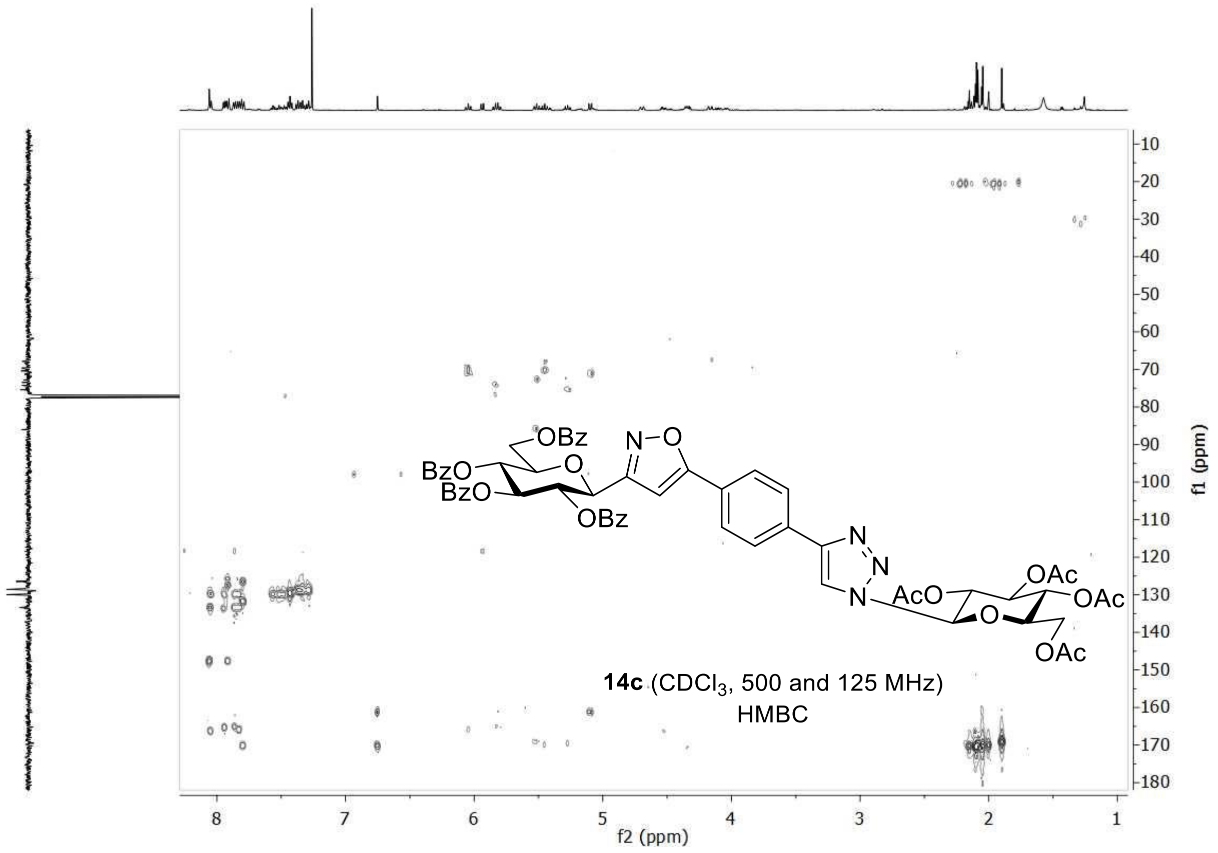

Figure S71. ^1H–^{13}C HMBC spectrum of **14c**

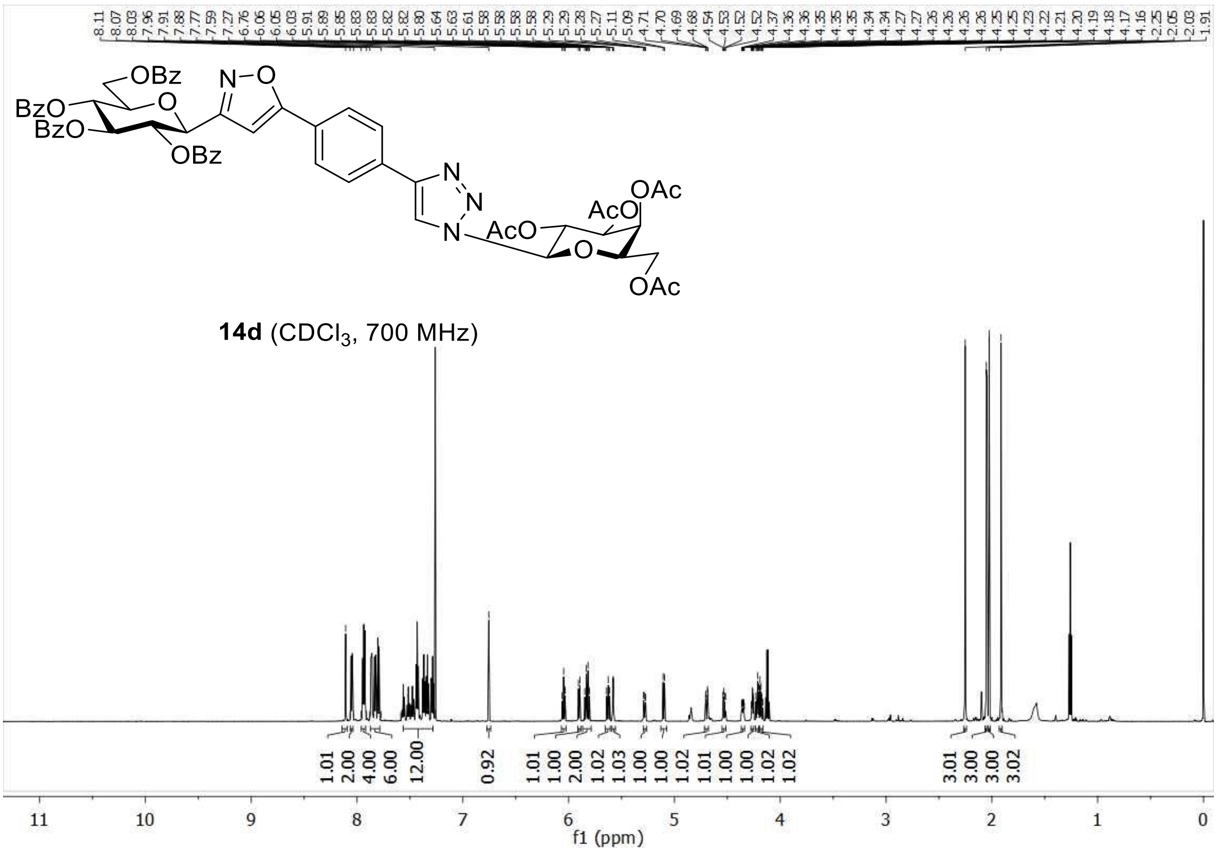

Figure S72. ^{1}H NMR spectrum of **14d**

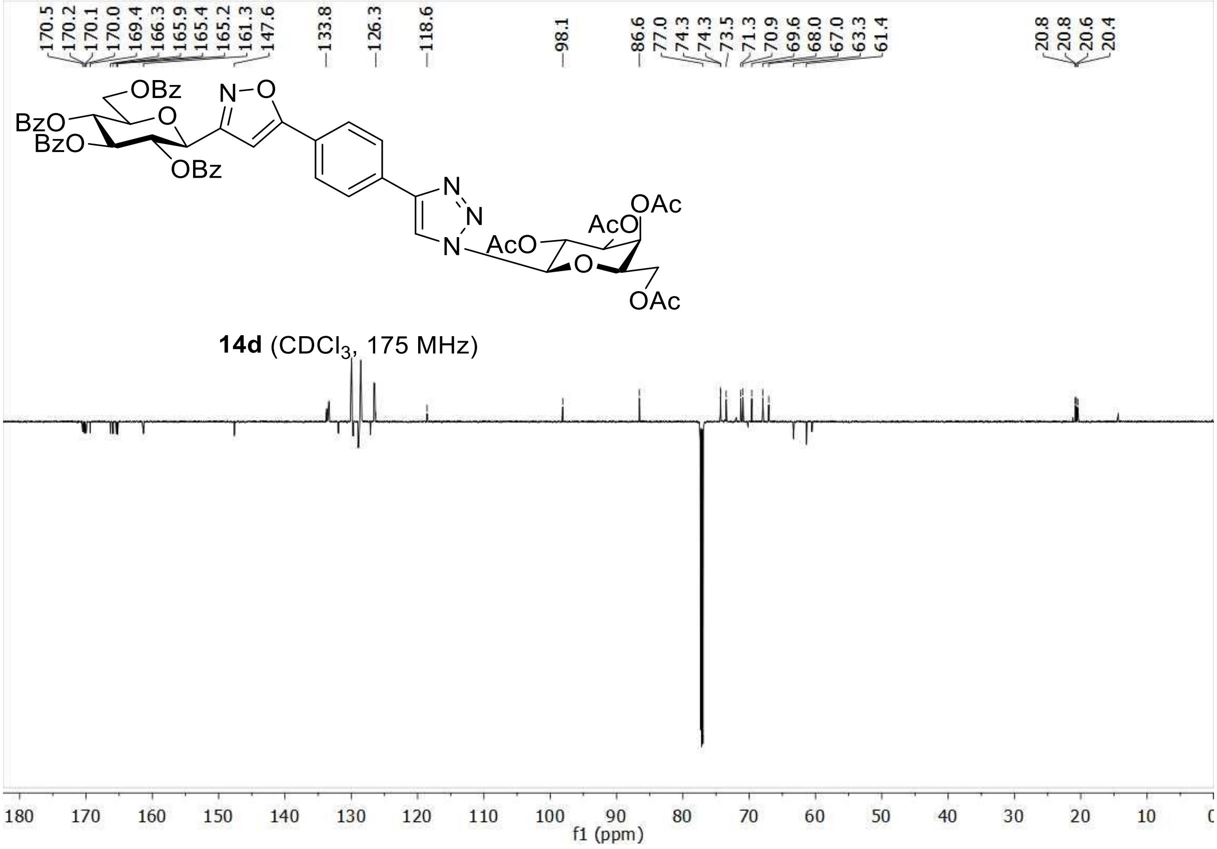

Figure S73. ^{13}C NMR spectrum of **14d**

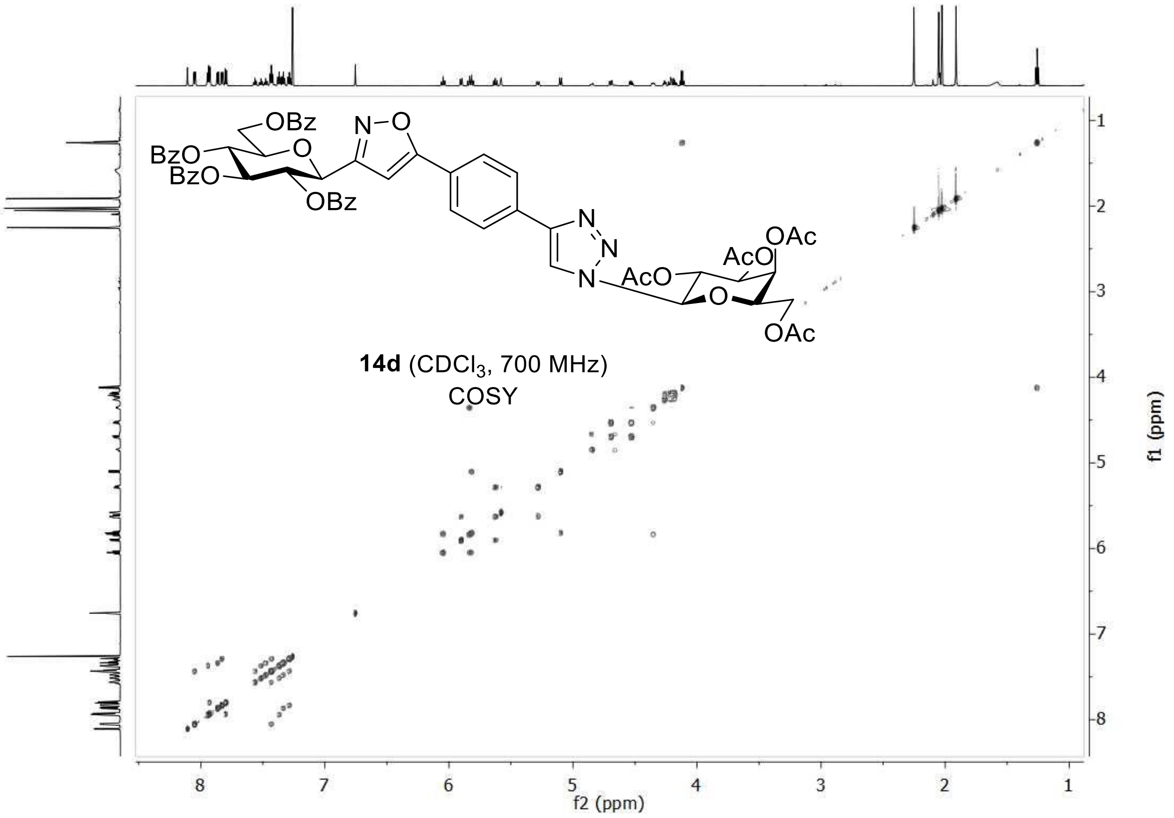

Figure S74. ^{1}H–^{1}H COSY spectrum of **14d**

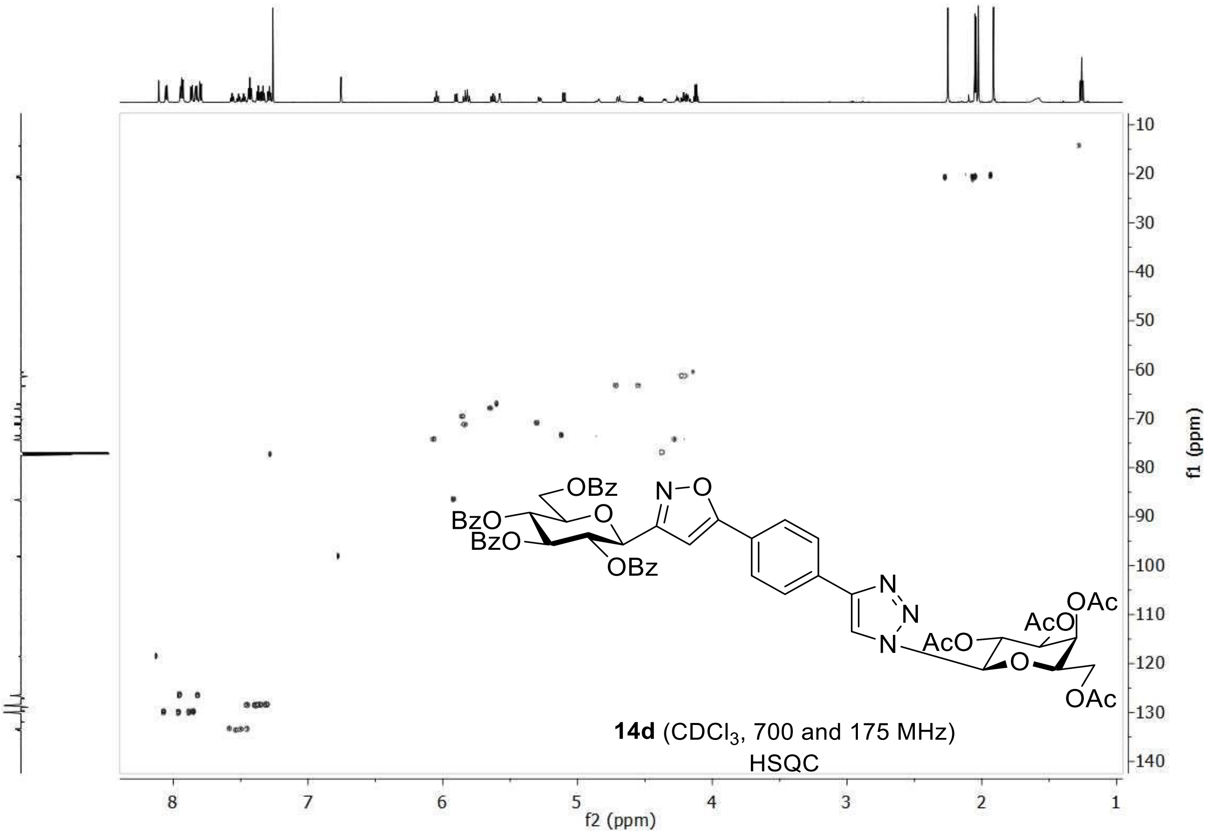

Figure S75. ^{1}H–^{13}C HSQC spectrum of **14d**

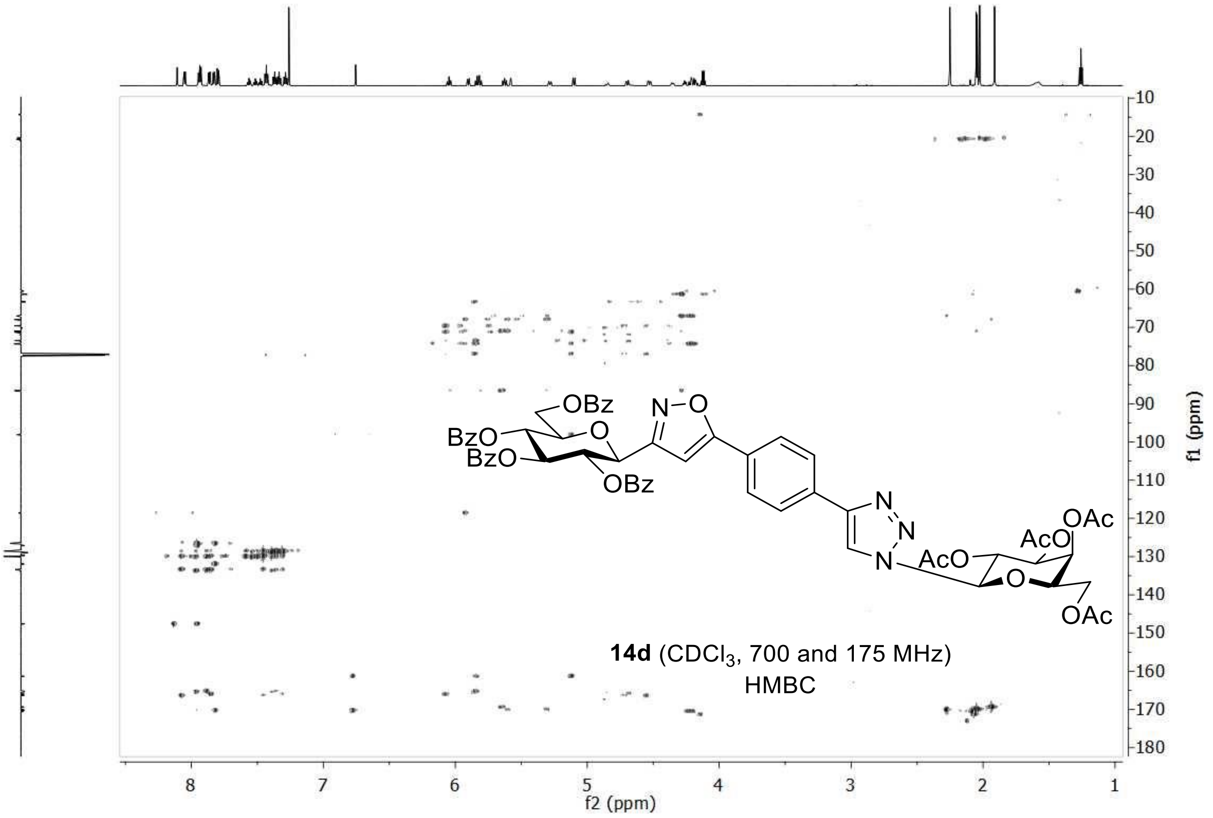

Figure S76. ^{1}H–^{13}C HMBC spectrum of **14d**

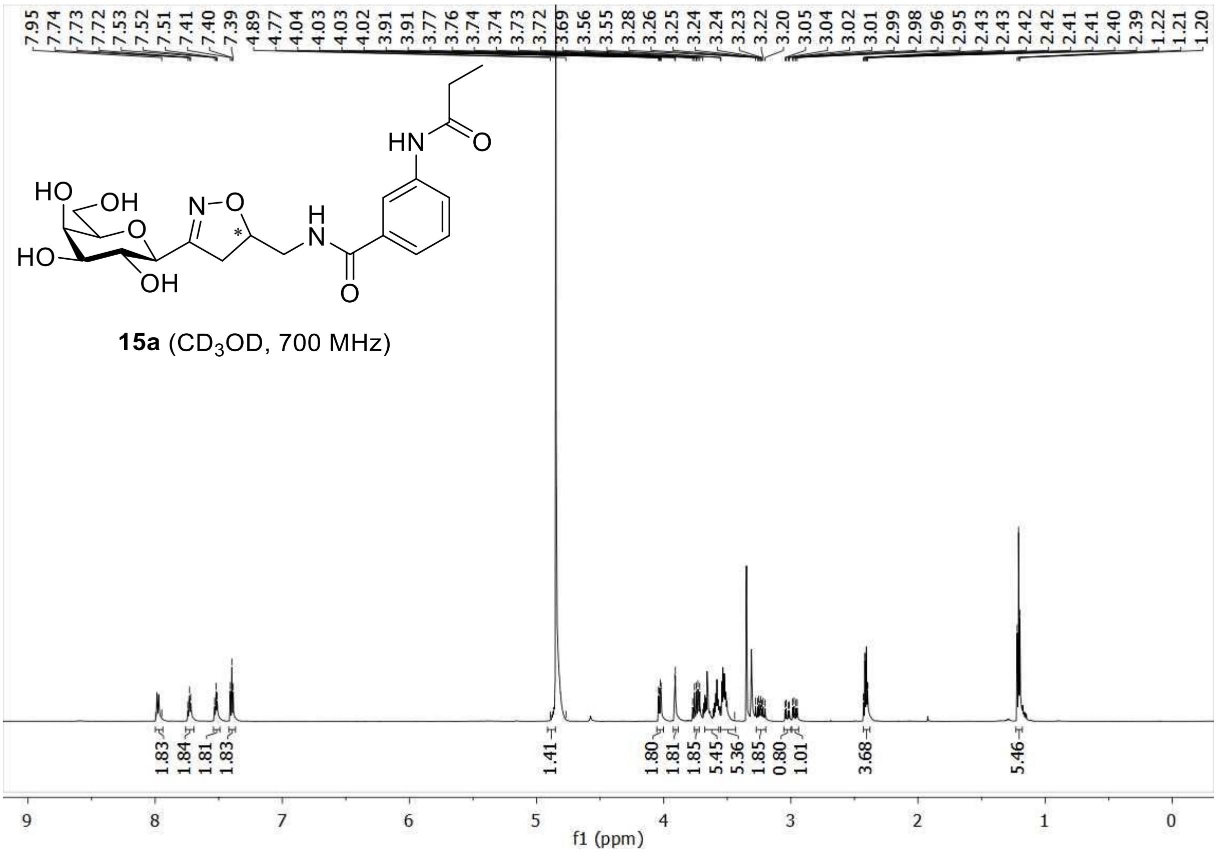

Figure S77. ^{1}H NMR spectrum of **15a**

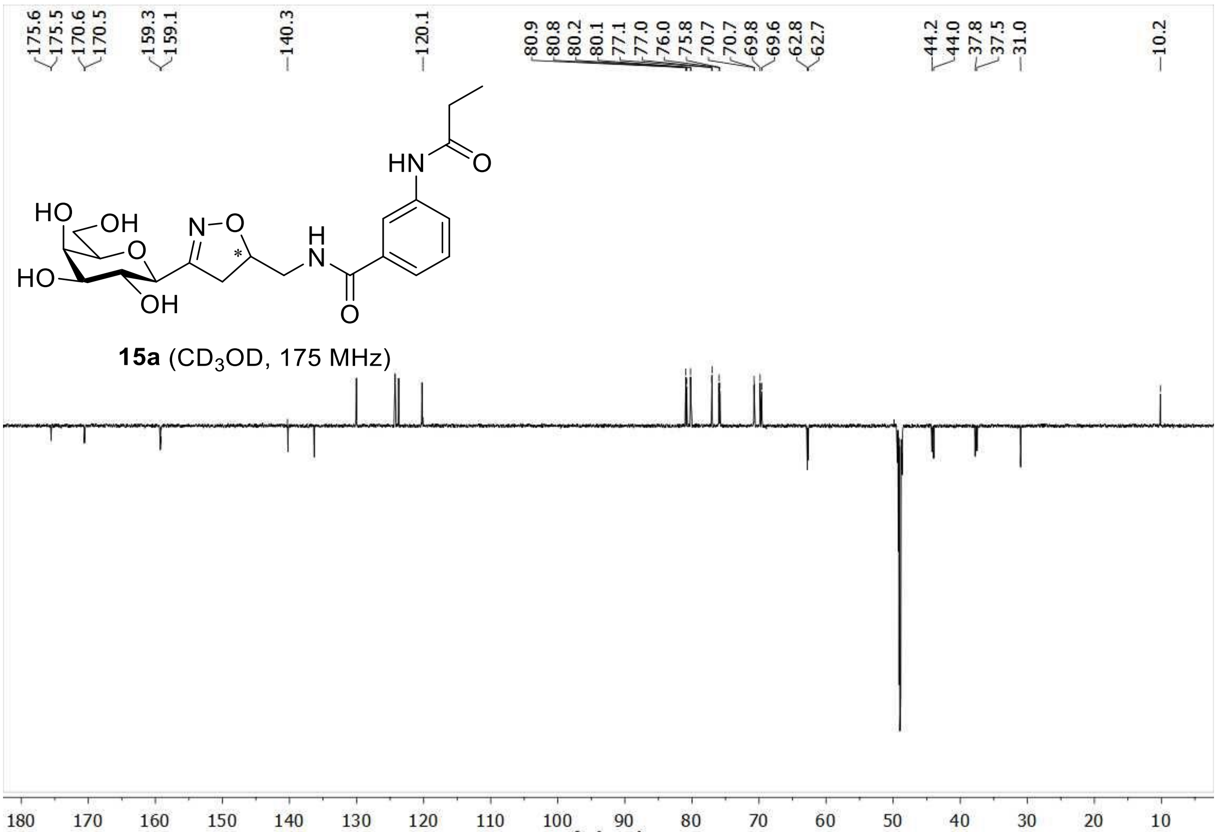

Figure S78. ^{13}C NMR spectrum of **15a**

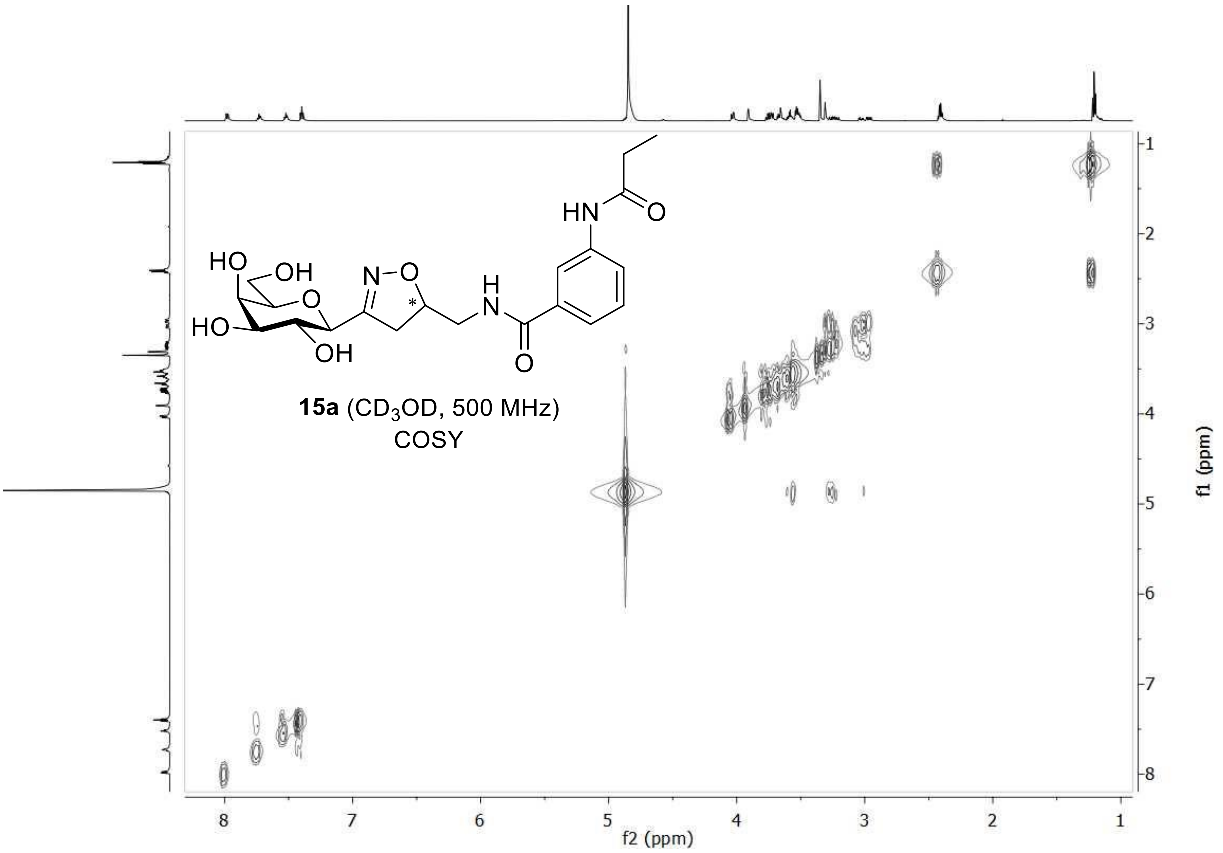

Figure S79. ^{1}H–^{1}H COSY spectrum of **15a**

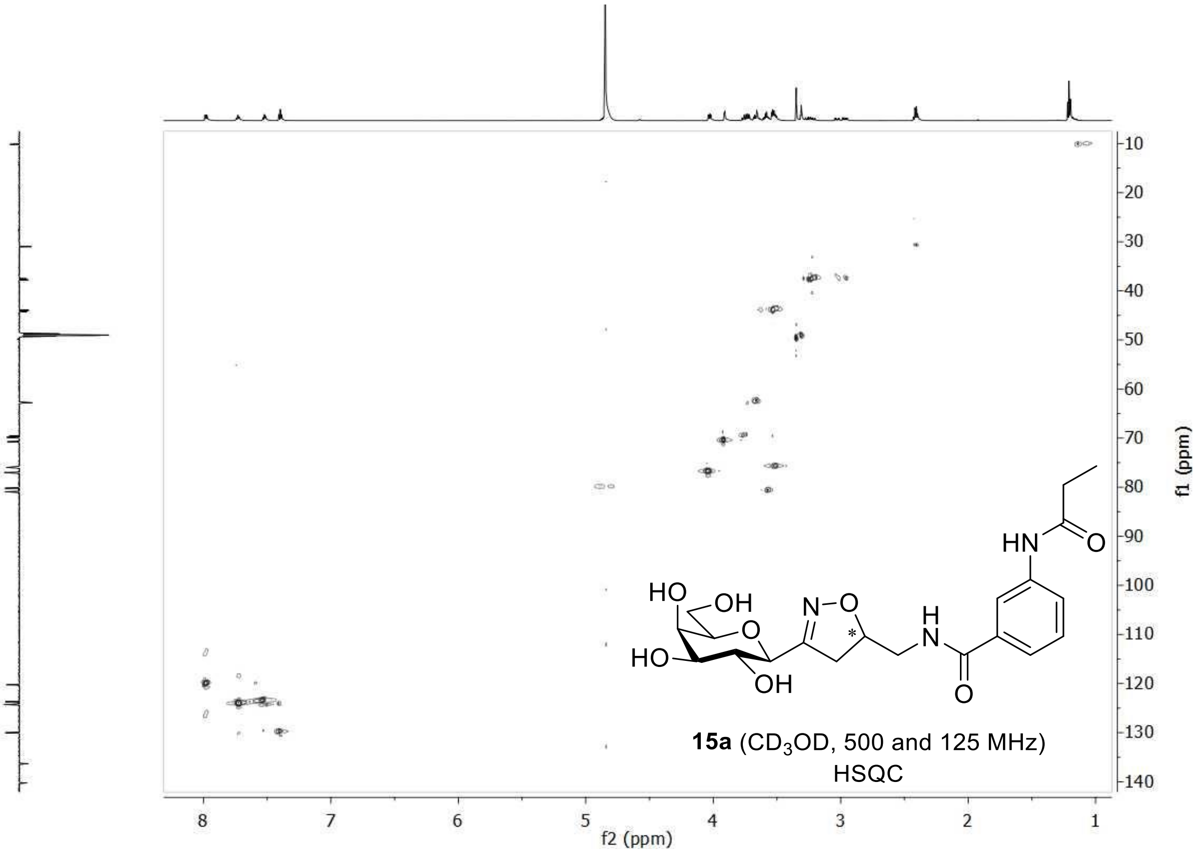

Figure S80. ^{1}H–^{13}C HSQC spectrum of **15a**

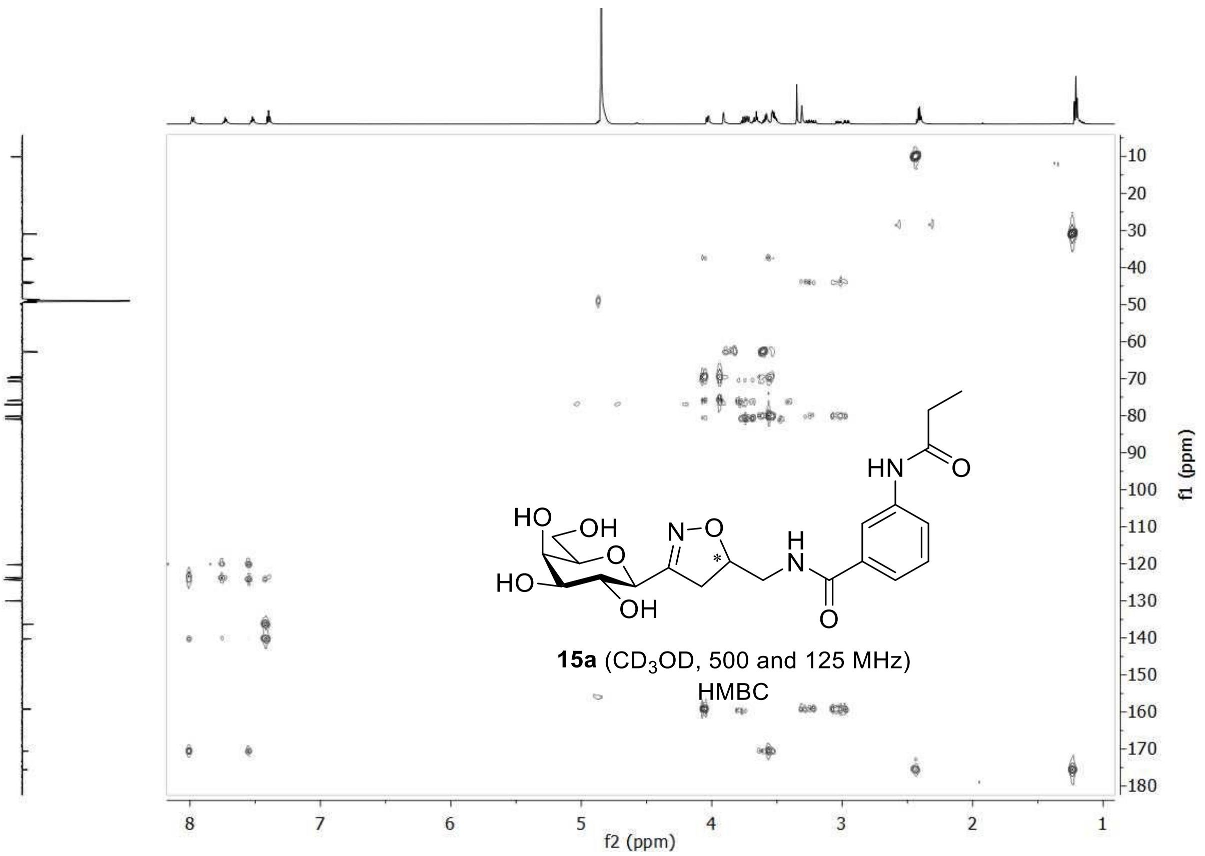

Figure S81. ^{1}H–^{13}C HMBC spectrum of **15a**

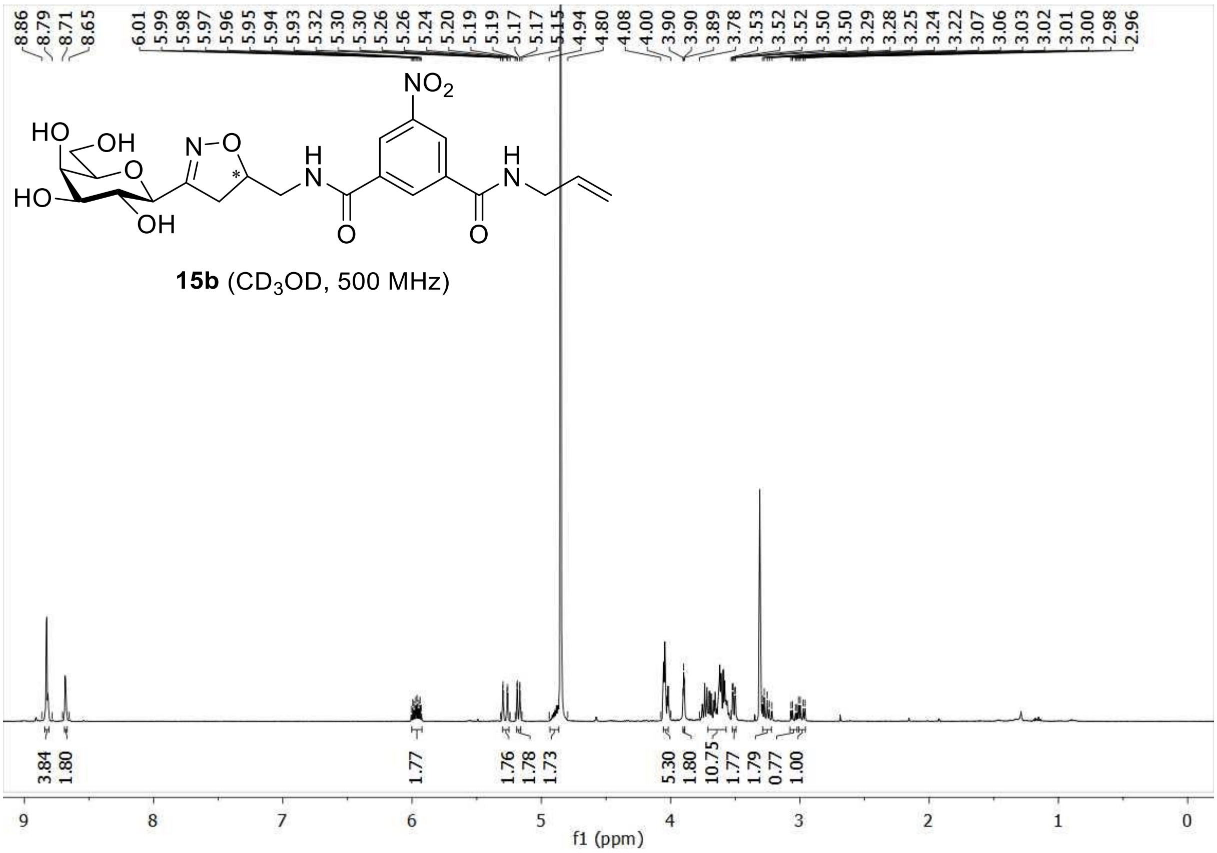

Figure S82. ^{1}H NMR spectrum of **15b**

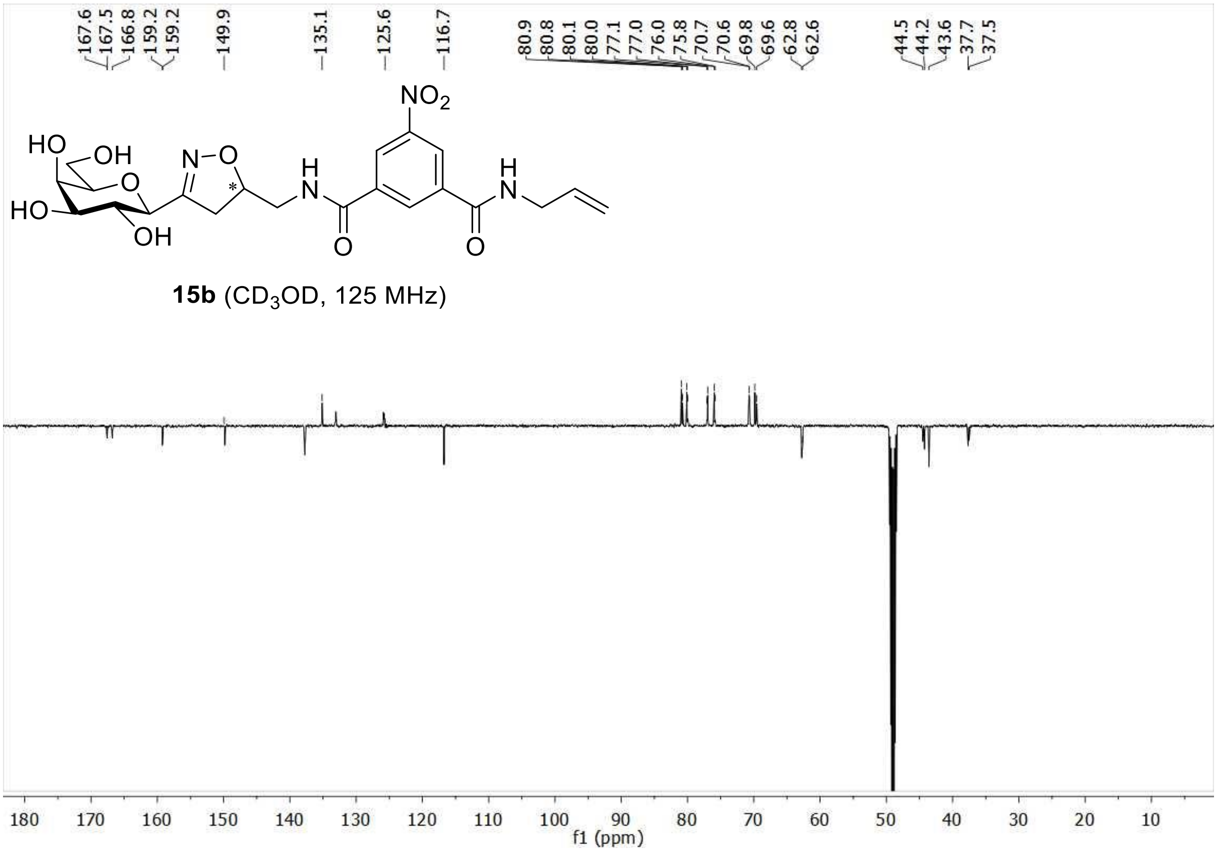

Figure S83. ^{13}C NMR spectrum of **15b**

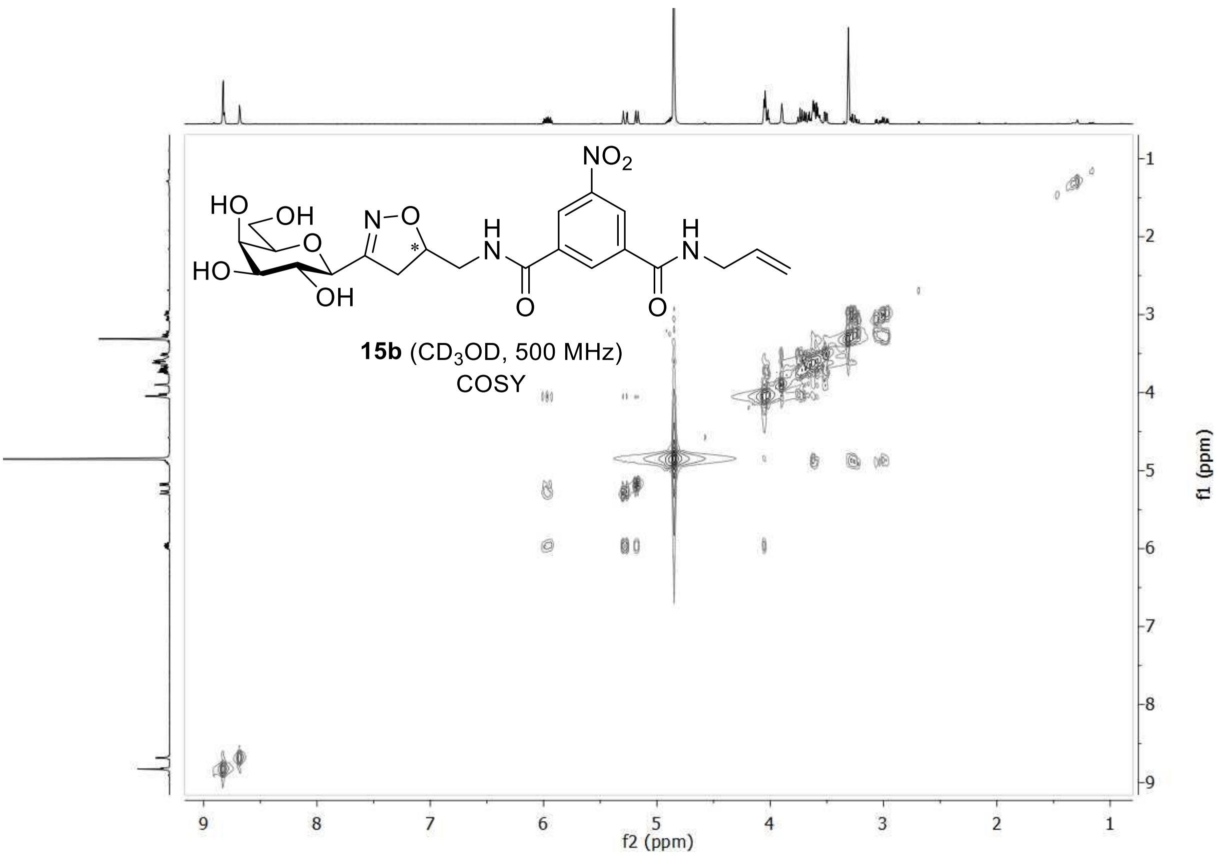

Figure S84. ^{1}H–^{1}H COSY spectrum of **15b**

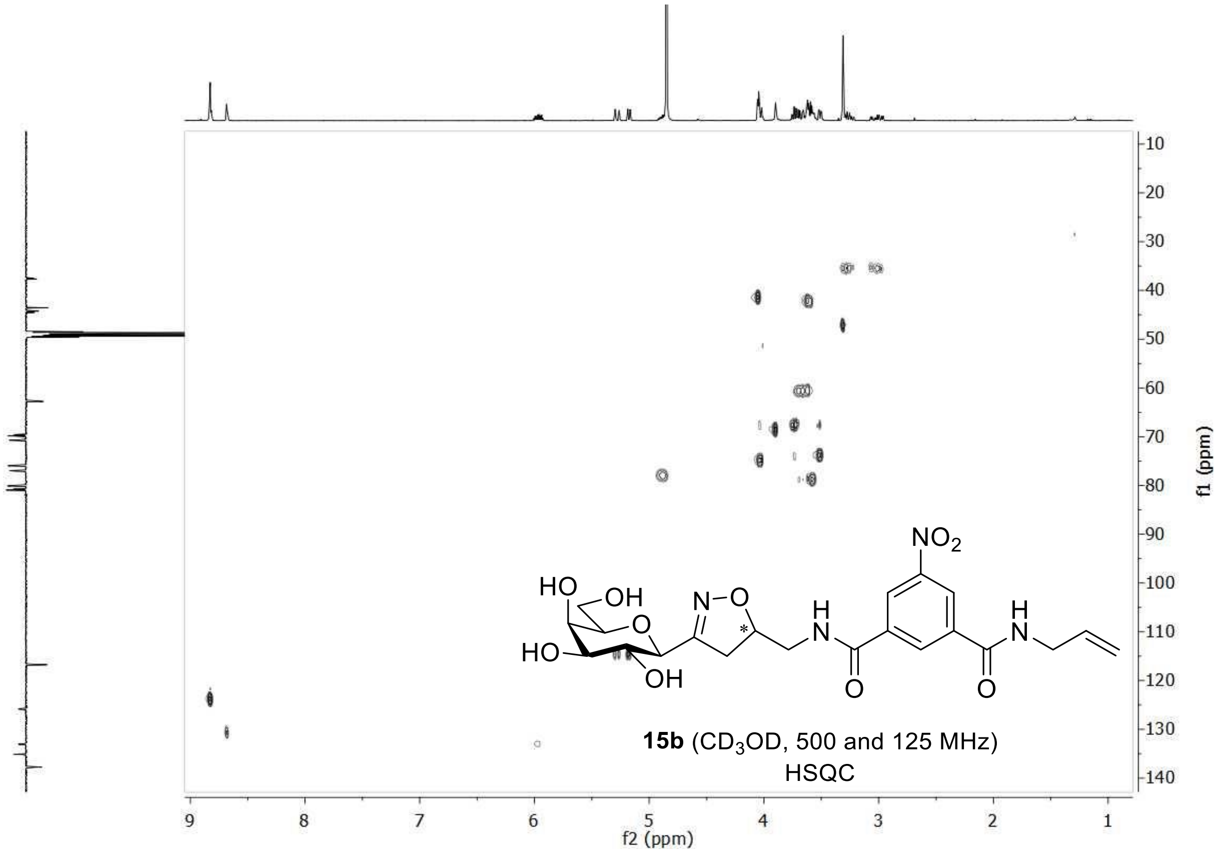

Figure S85. ^{1}H–^{13}C HSQC spectrum of **15b**

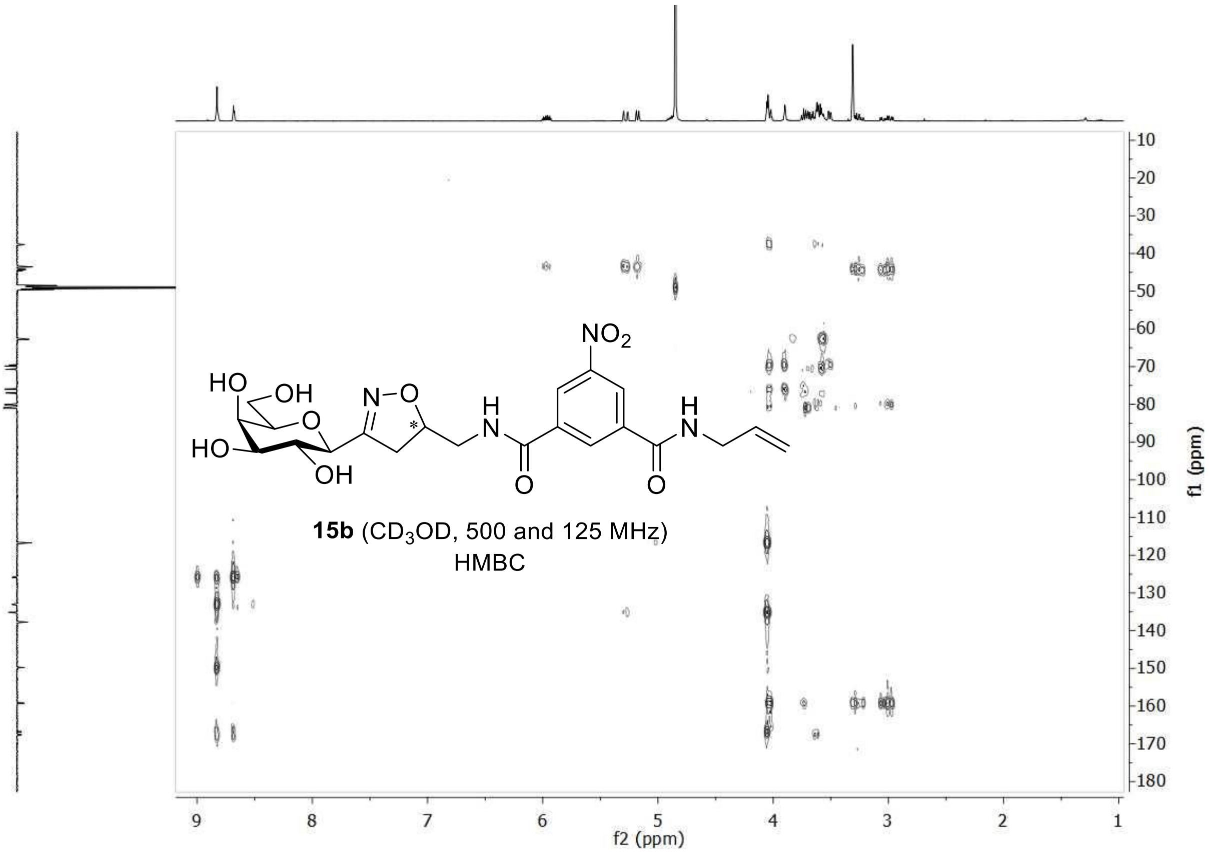

Figure S86. ^{1}H–^{13}C HMBC spectrum of **15b**

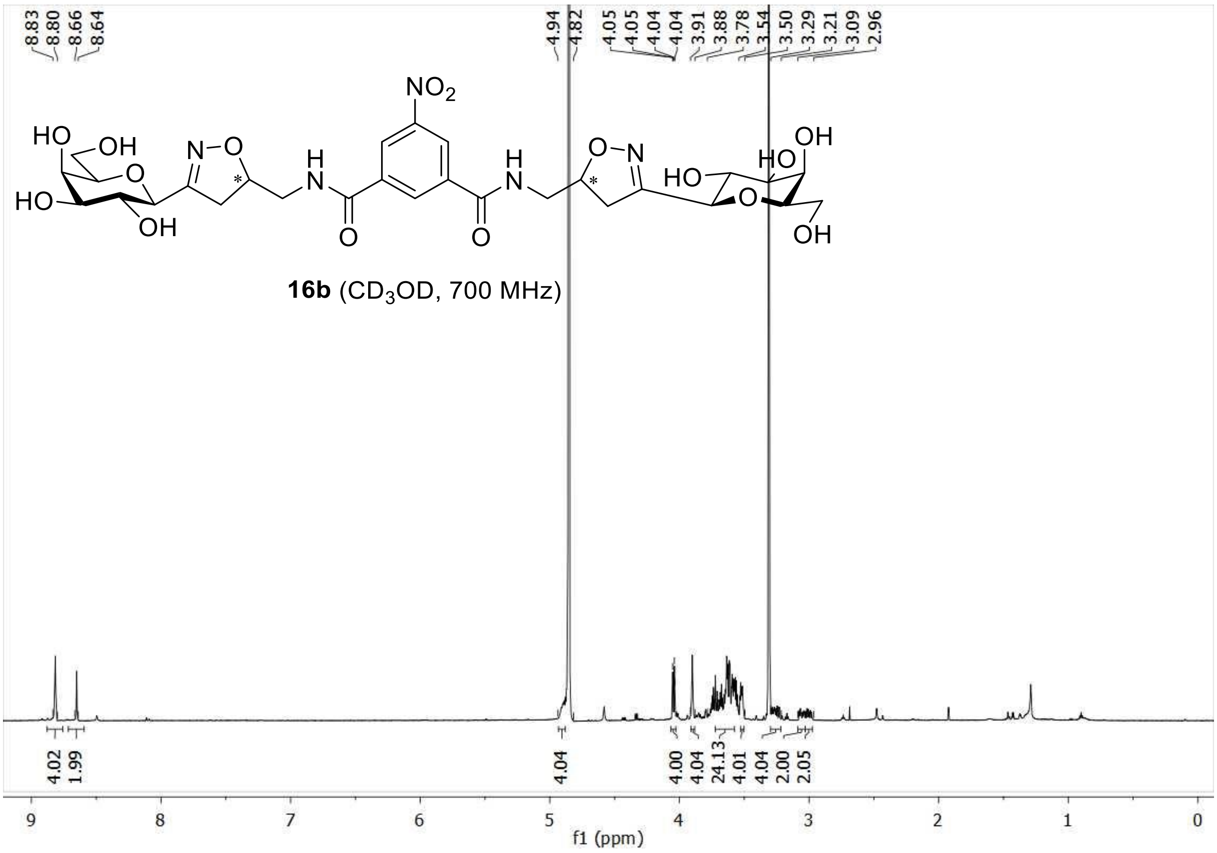

Figure S87. ^{1}H NMR spectrum of **16b**

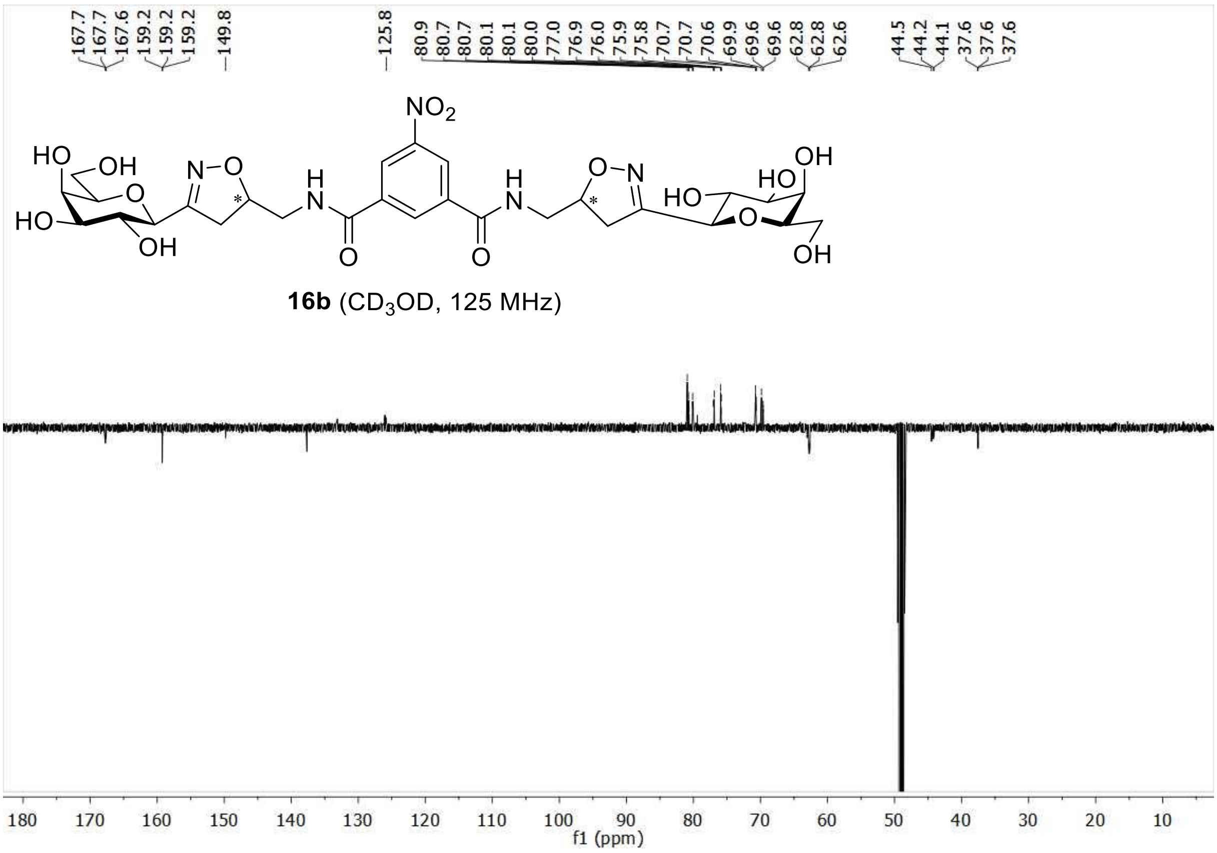

Figure S88. ^{13}C NMR spectrum of **16b**

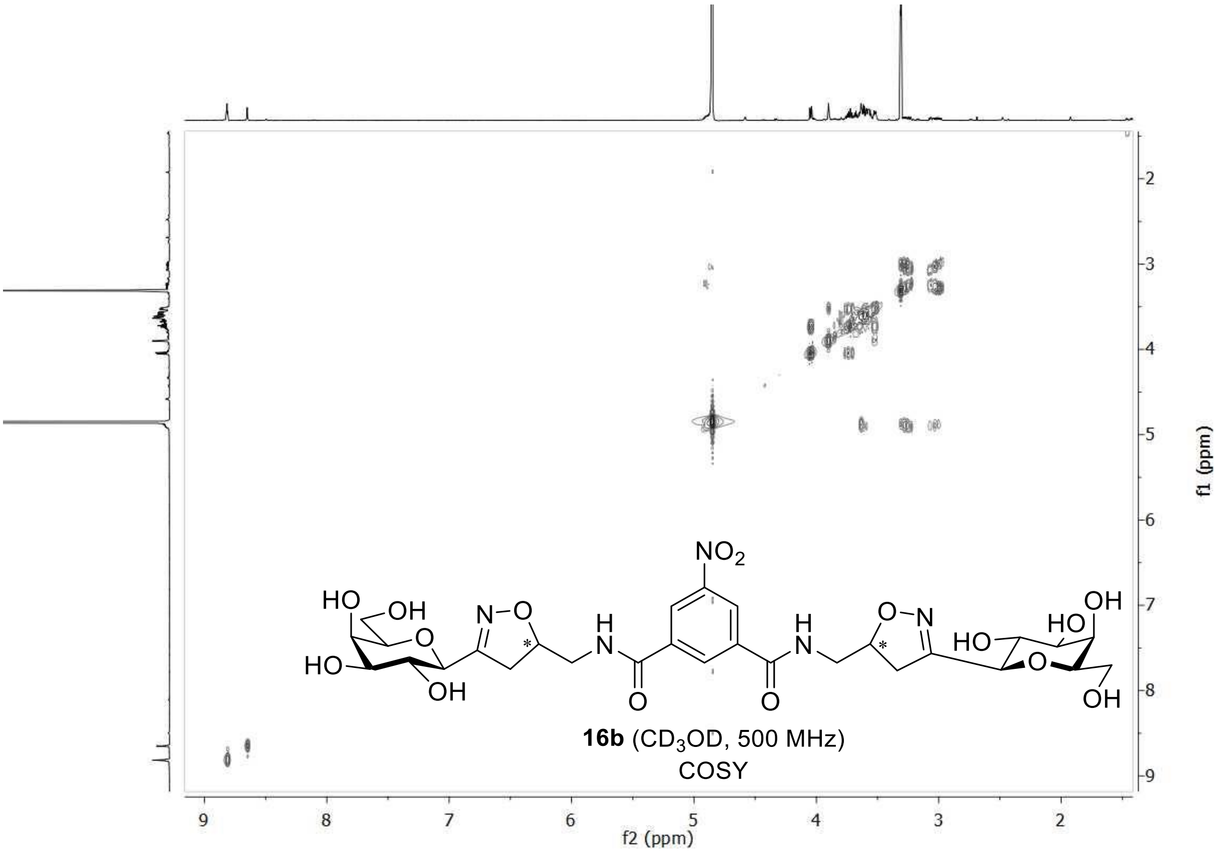

Figure S89. ^{1}H–^{1}H COSY spectrum of **16b**

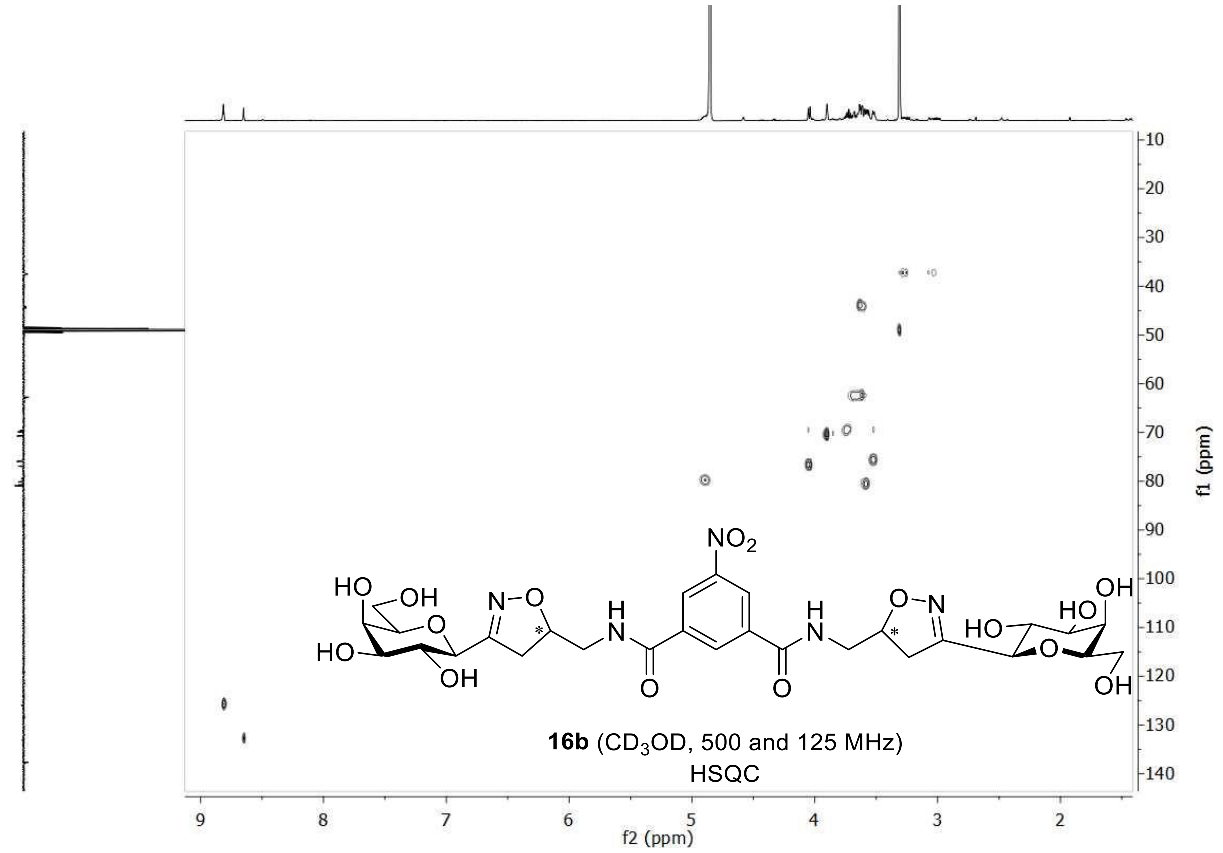

Figure S90. ^{1}H–^{13}C HSQC spectrum of **16b**

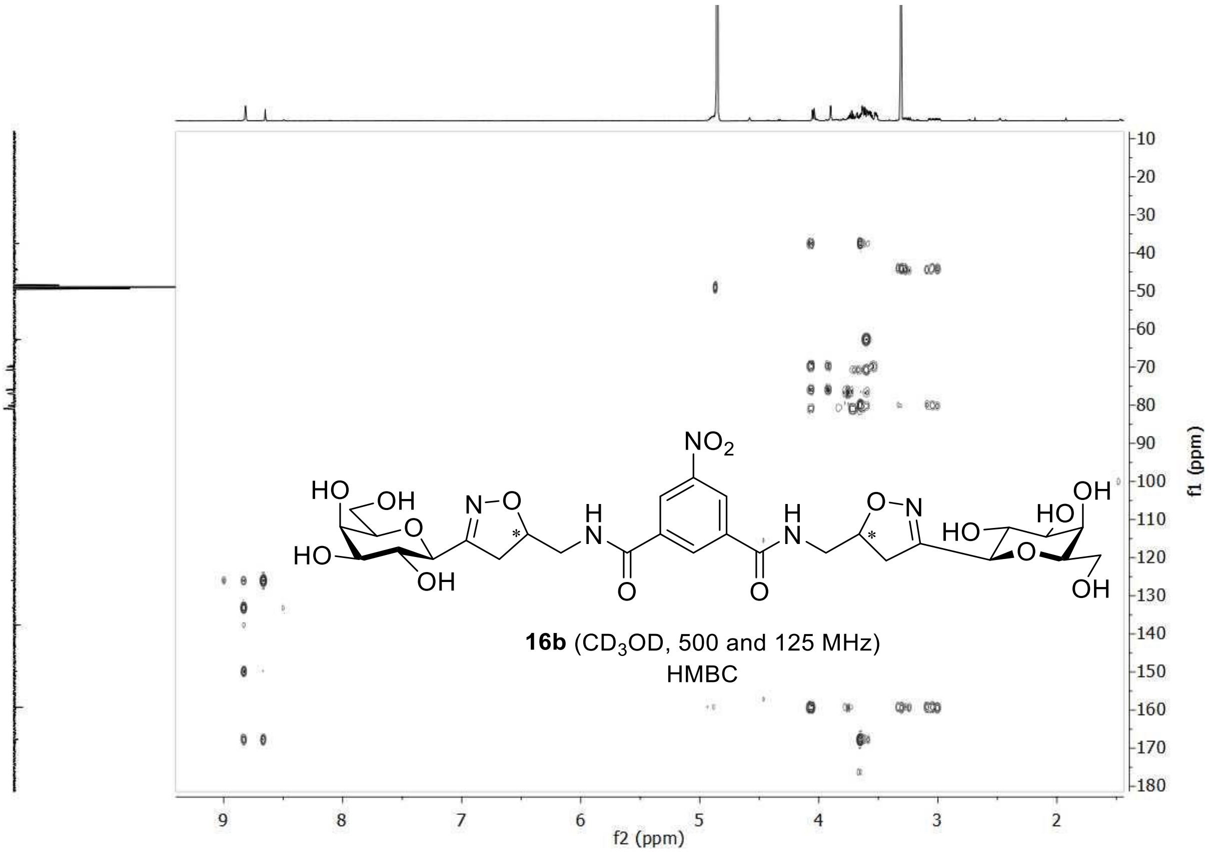

Figure S91. ^{1}H–^{13}C HMBC spectrum of **16b**

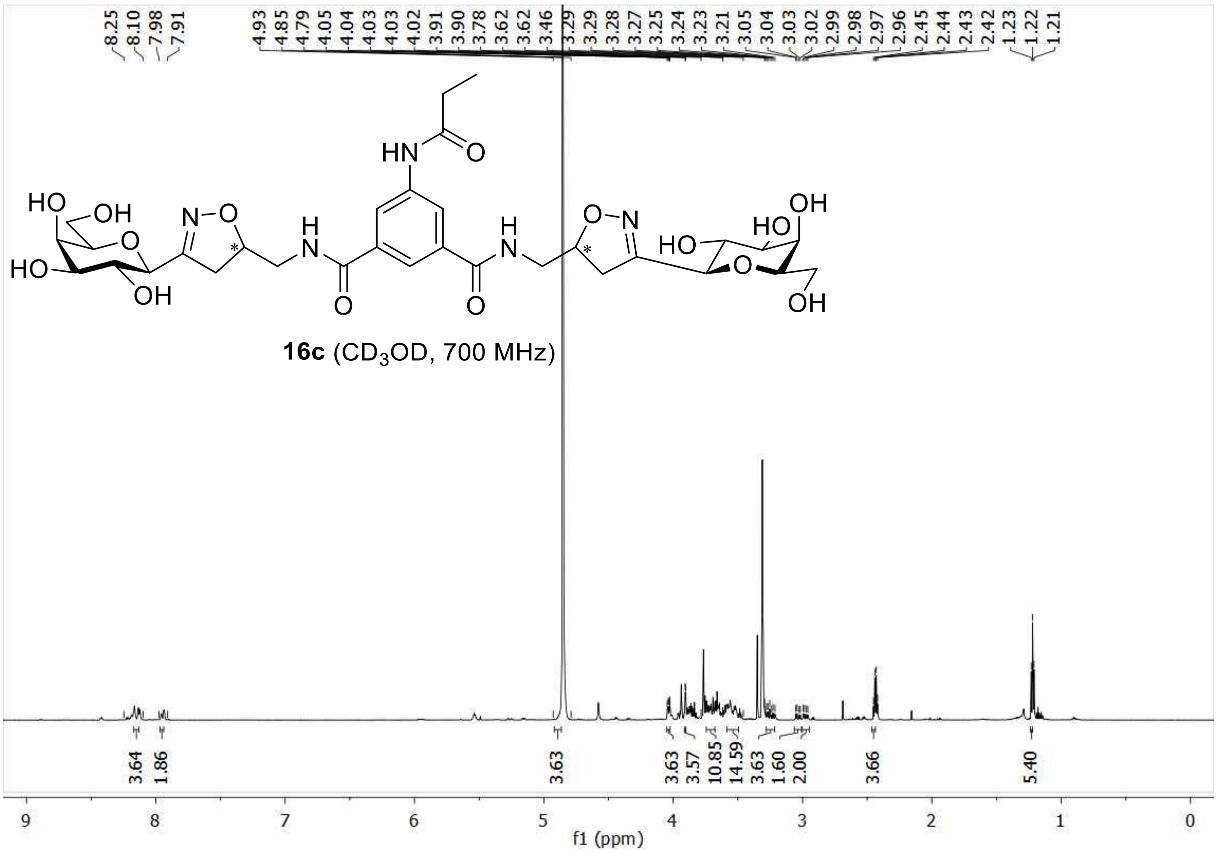

Figure S92. ^{1}H NMR spectrum of **16c**

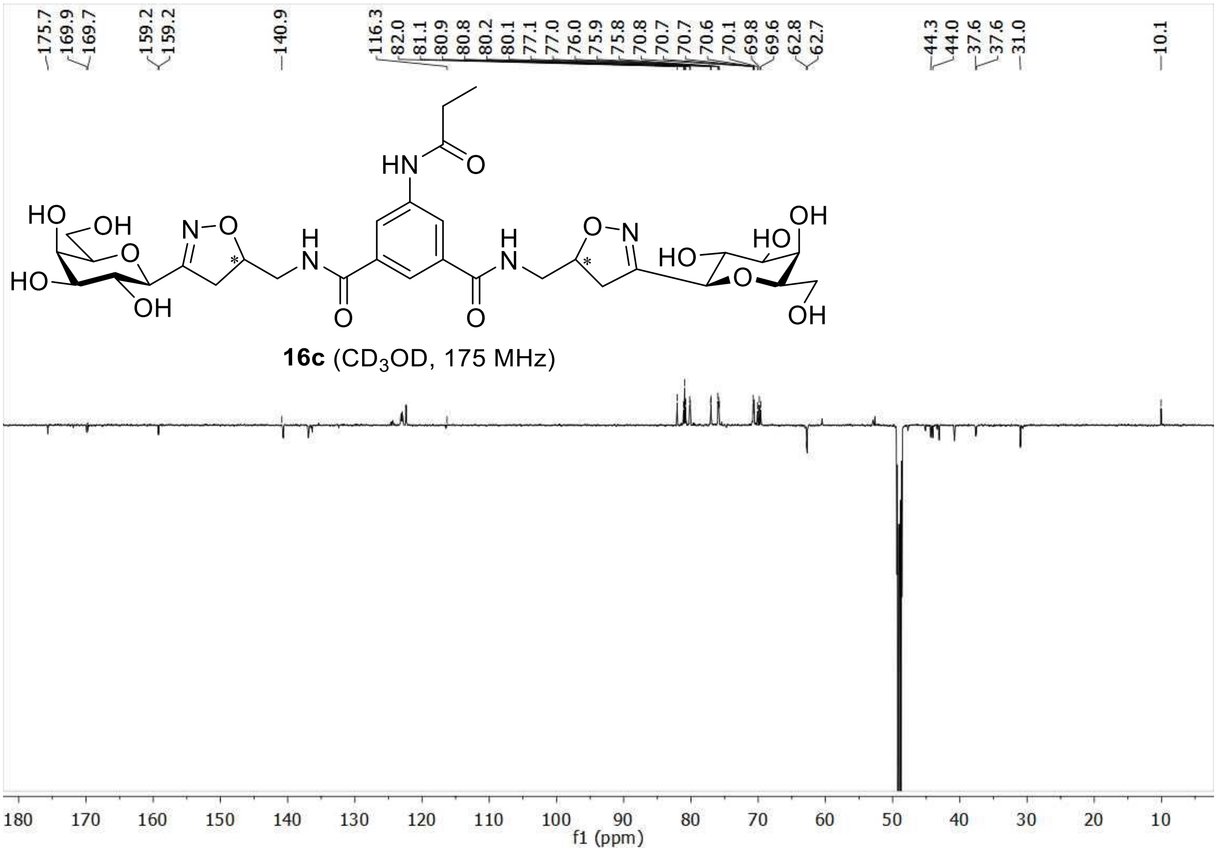

Figure S93. ^{13}C NMR spectrum of **16c**

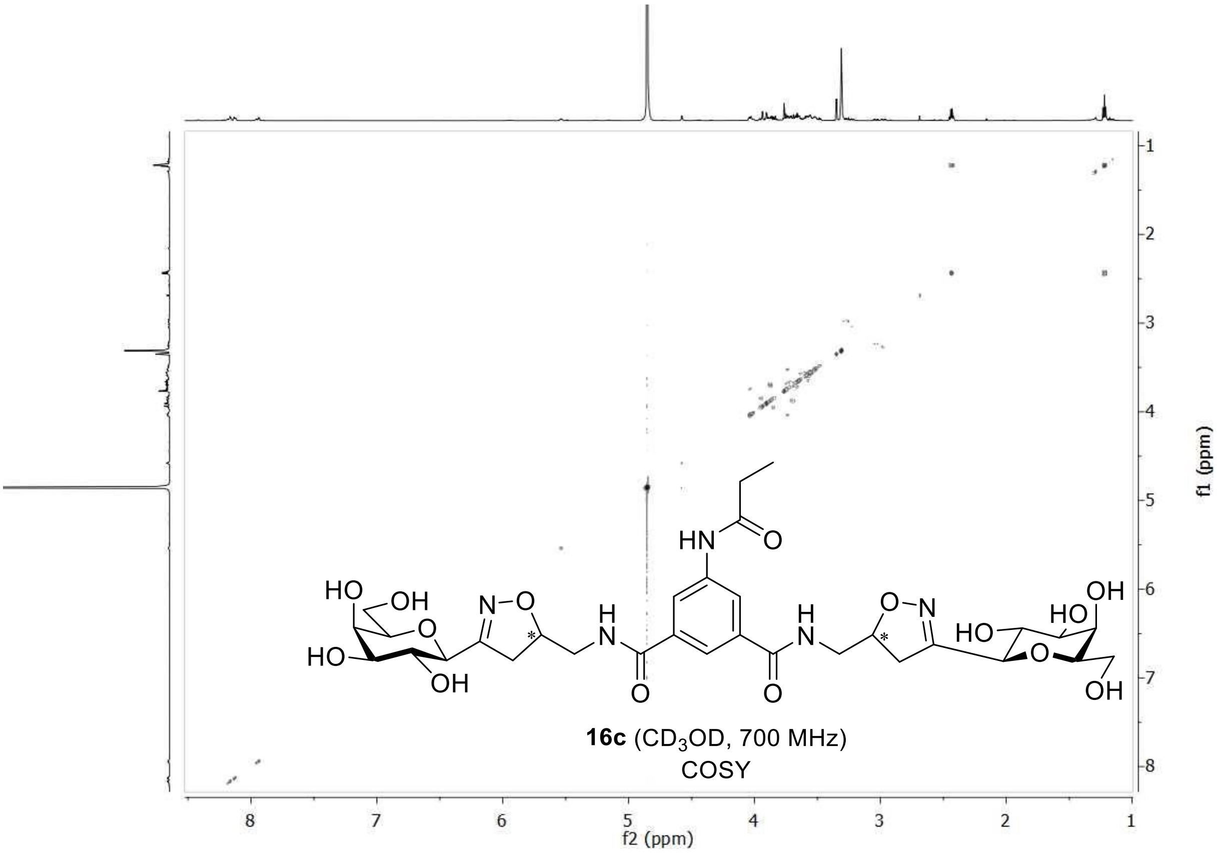

Figure S94. ^{1}H–^{1}H COSY spectrum of **16c**

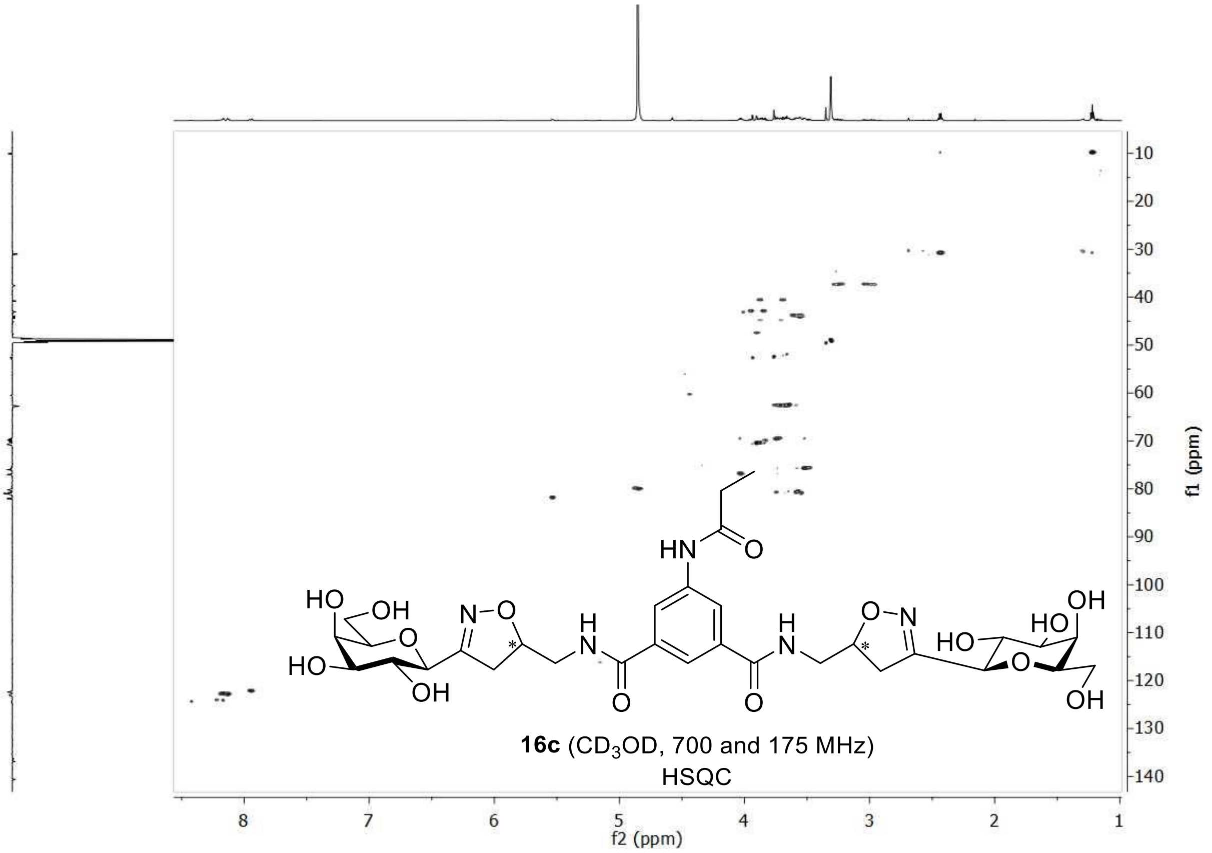

Figure S95. ^{1}H–^{13}C HSQC spectrum of **16c**

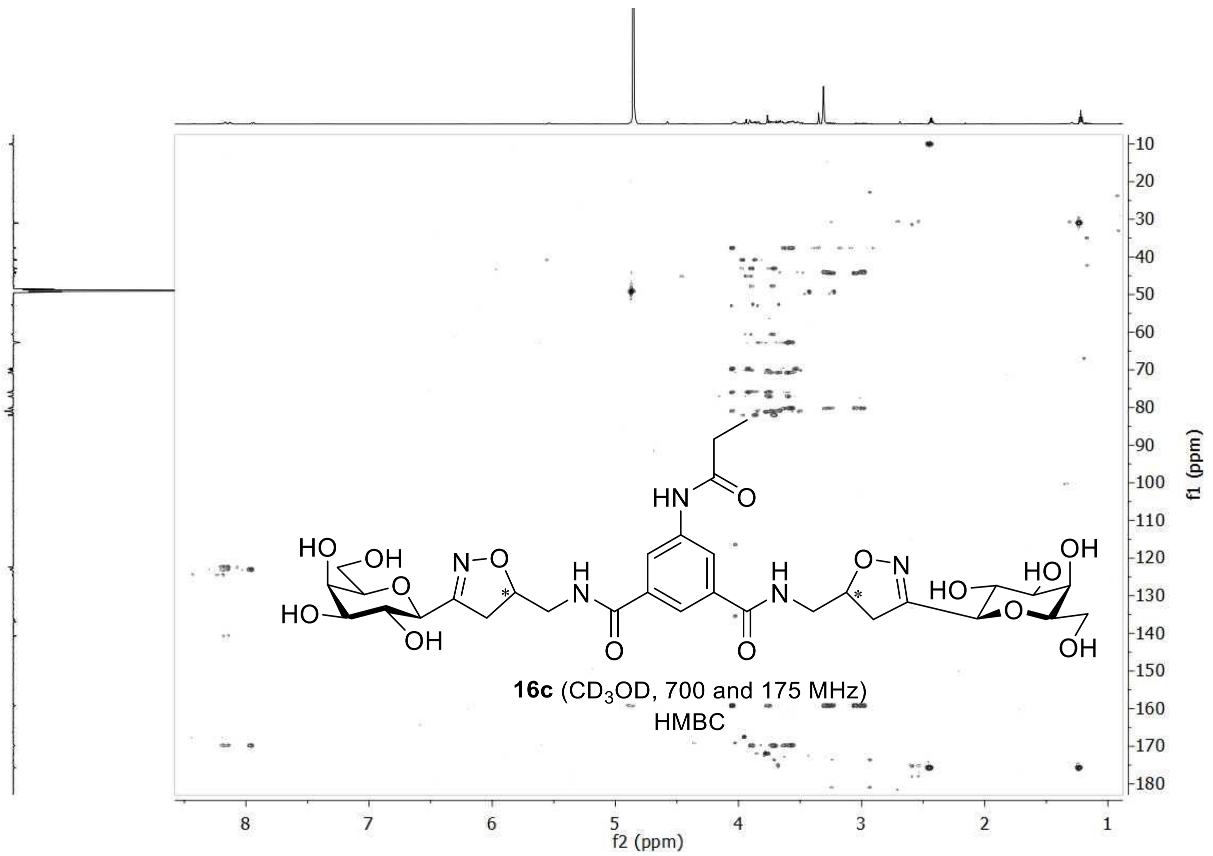

Figure S96. ^{1}H–^{13}C HMBC spectrum of **16c**

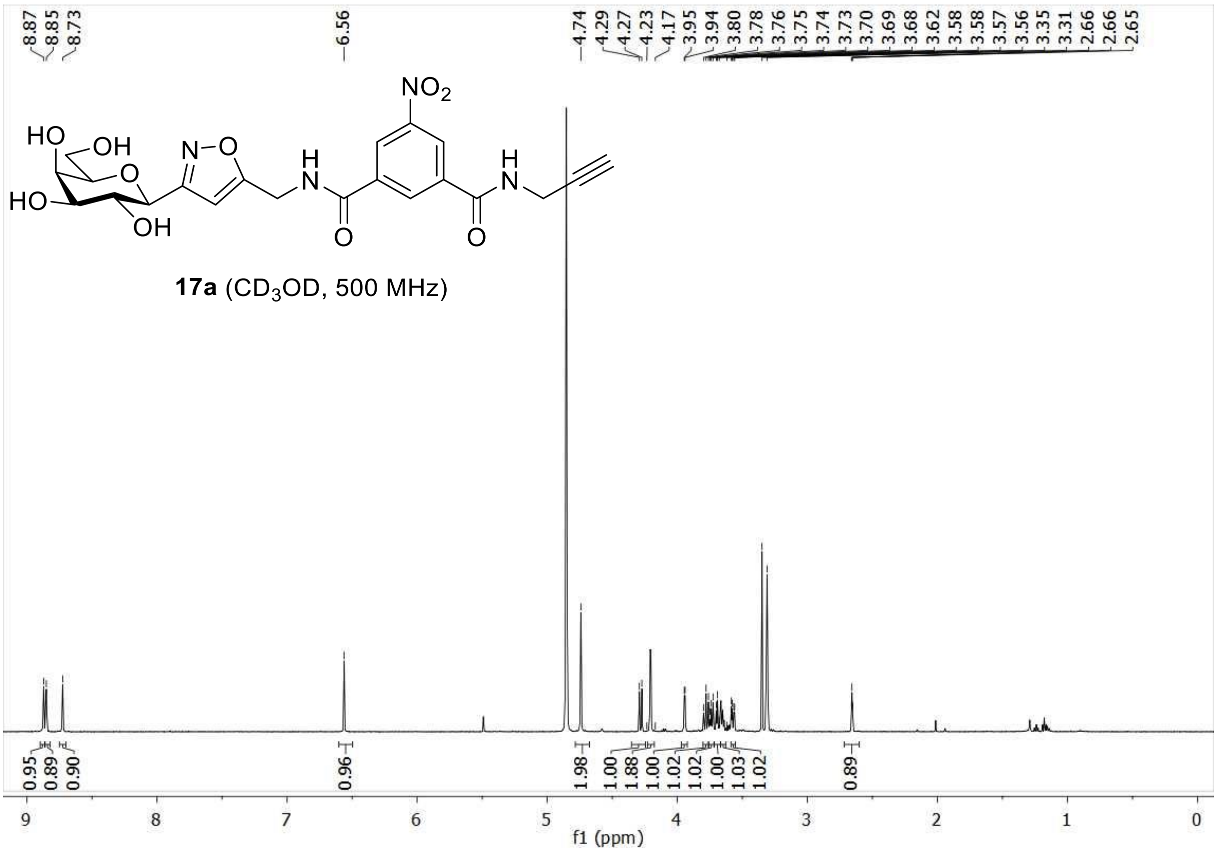

Figure S97. ^{1}H NMR spectrum of **17a**

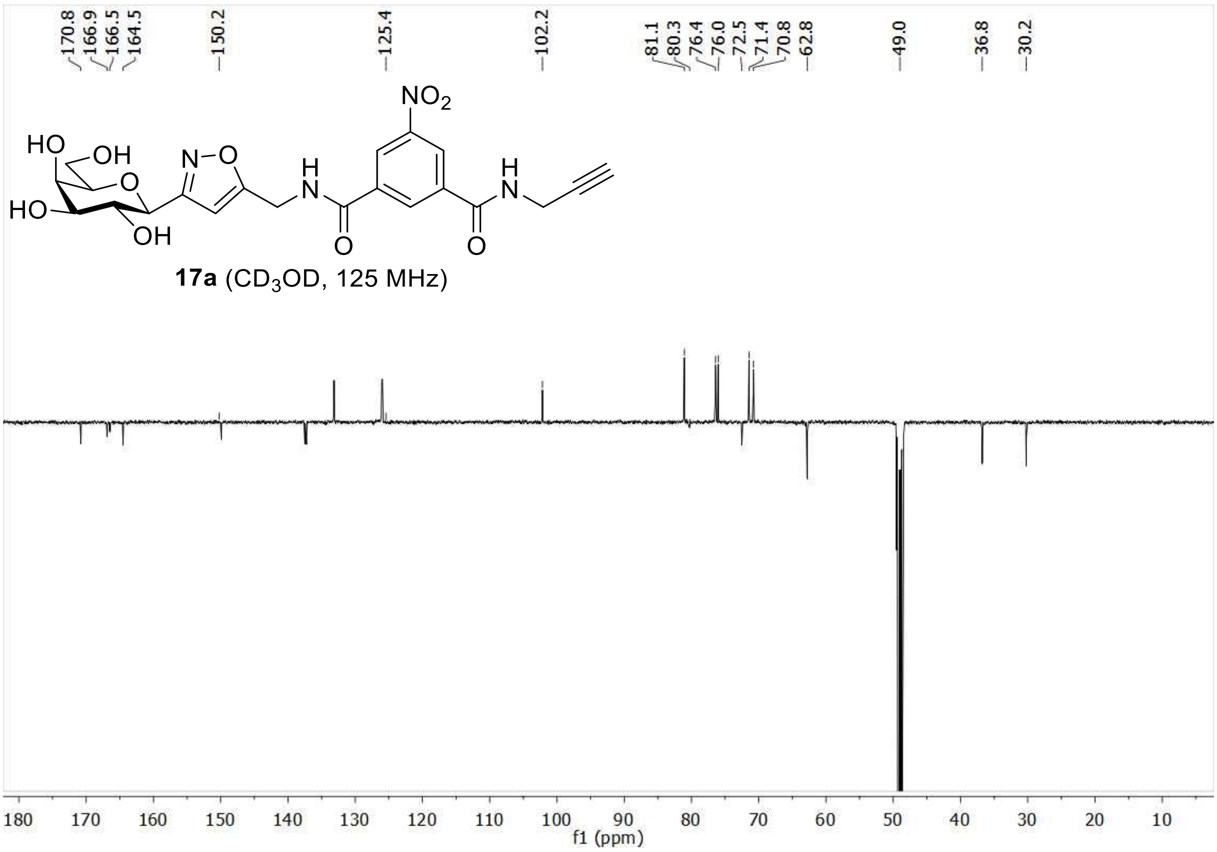

Figure S98. ^{13}C NMR spectrum of **17a**

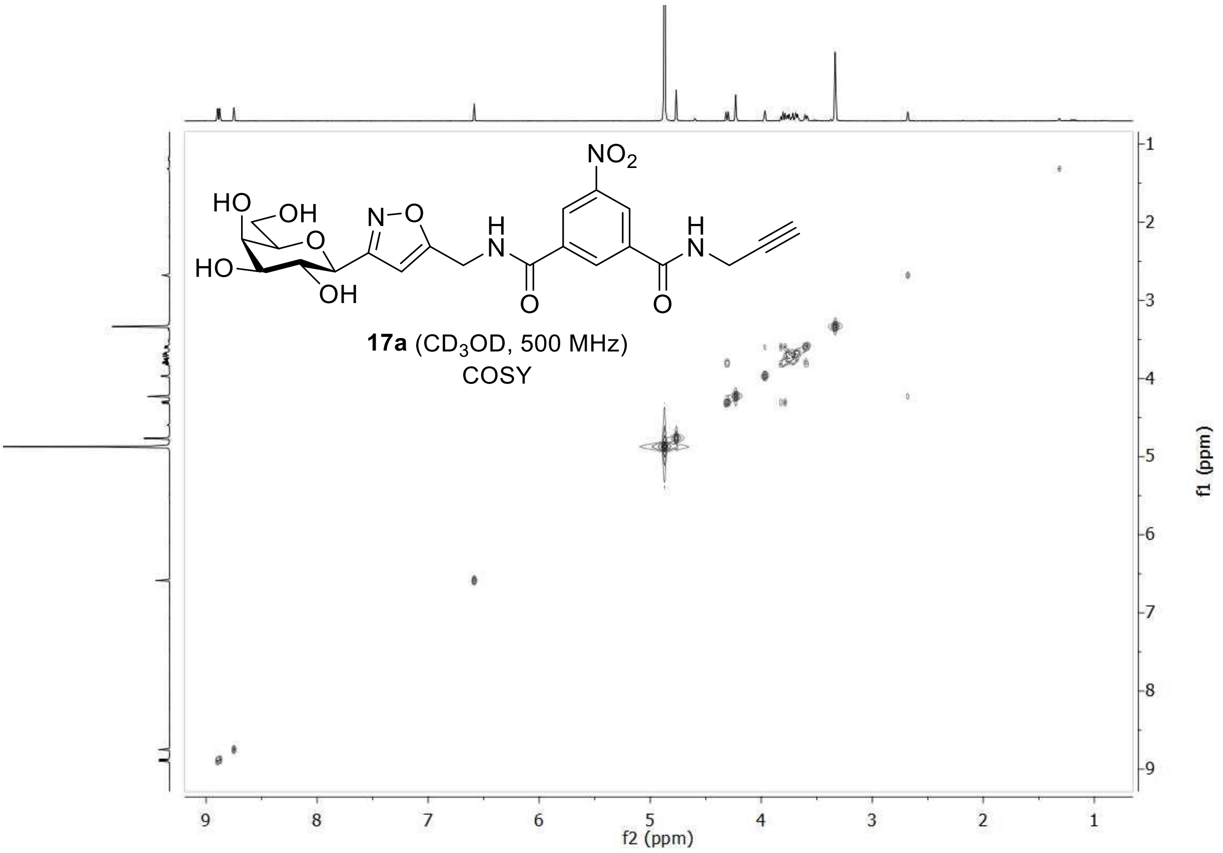

Figure S99. $^{1}H–^{1}H$ COSY spectrum of **17a**

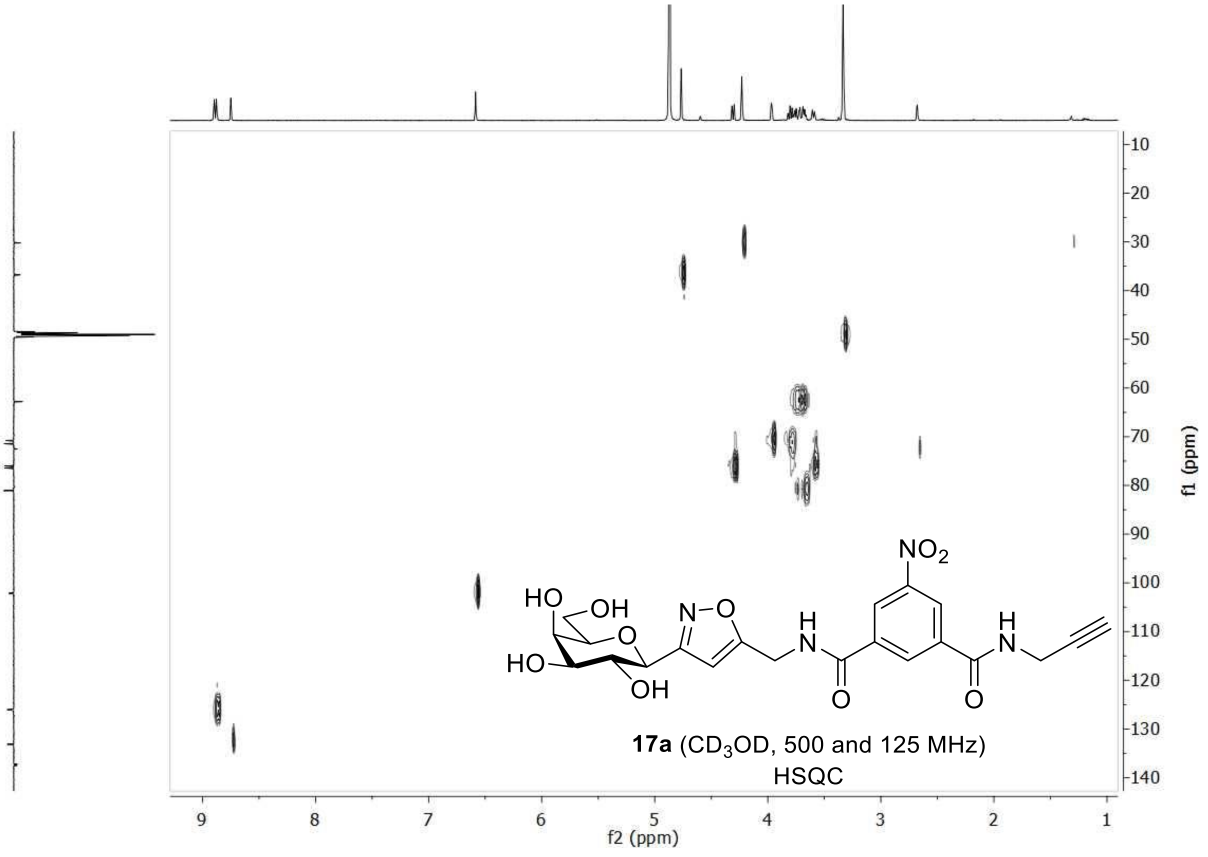

Figure S100. $^{1}H–^{13}C$ HSQC spectrum of **17a**

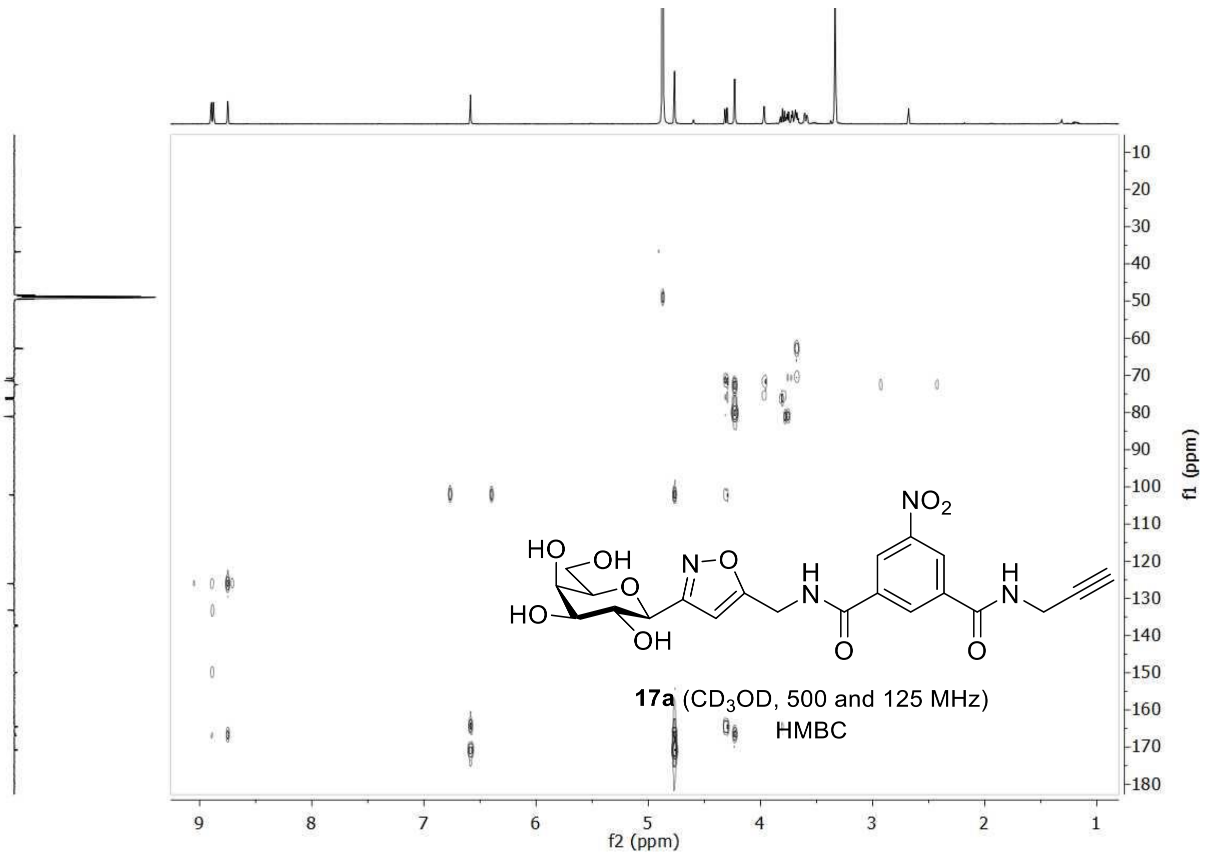

Figure S101. ^{1}H–^{13}C HMBC spectrum of **17a**

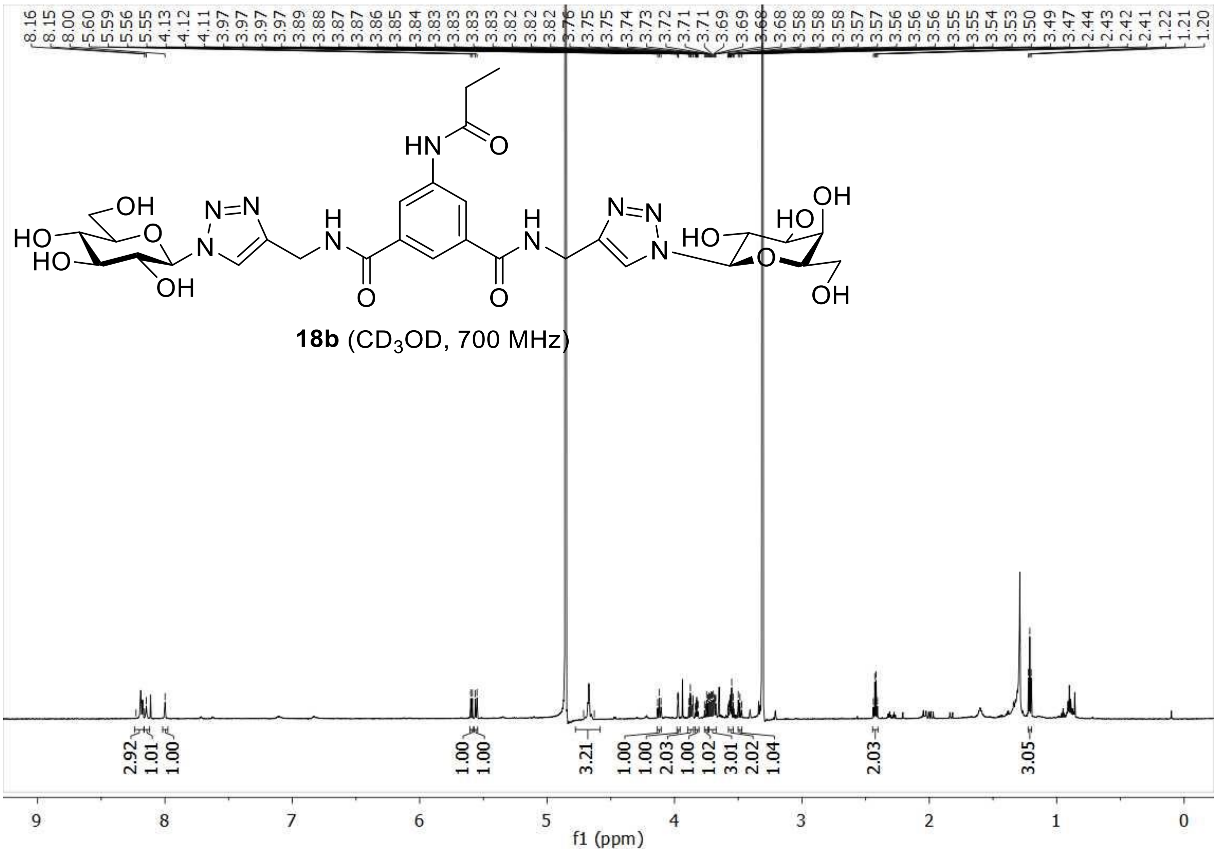

Figure S102. ^{1}H NMR spectrum of **18b**

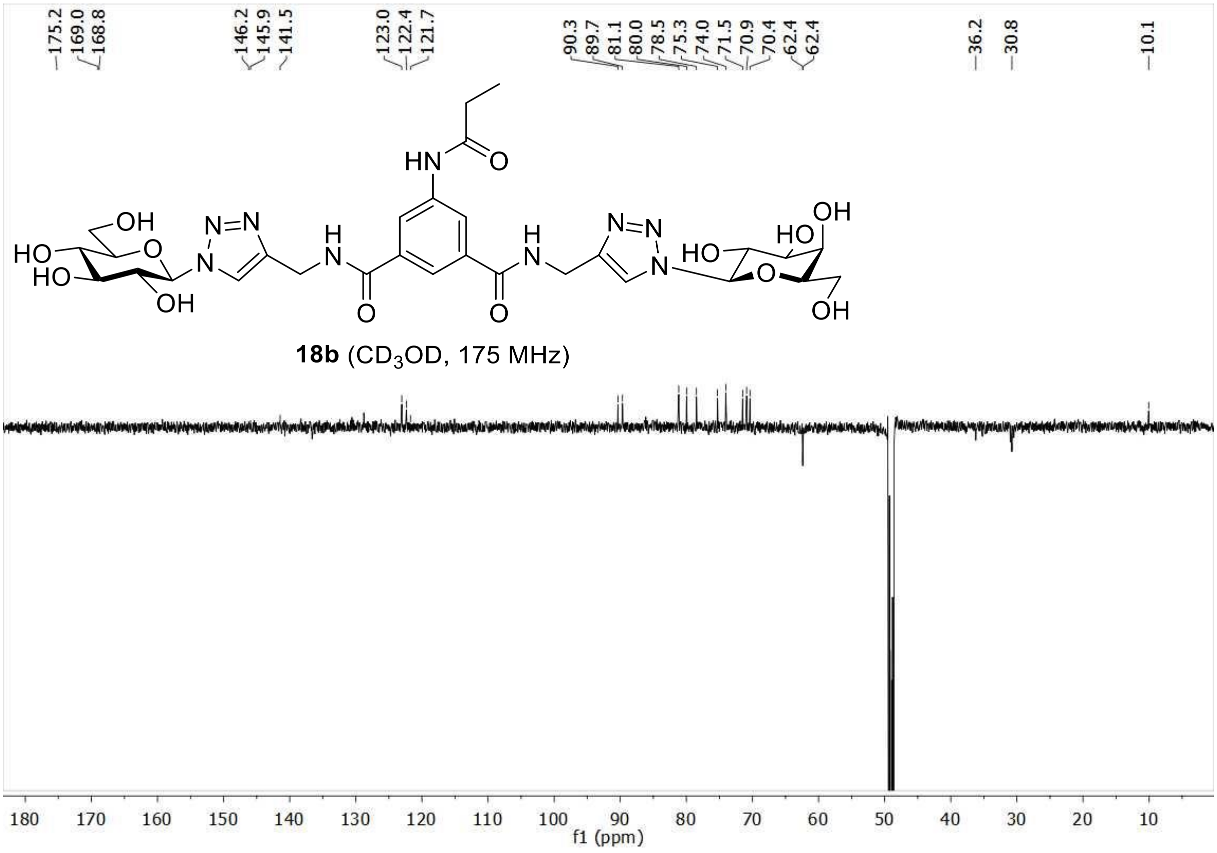

Figure S103. ^{13}C NMR spectrum of **18b**

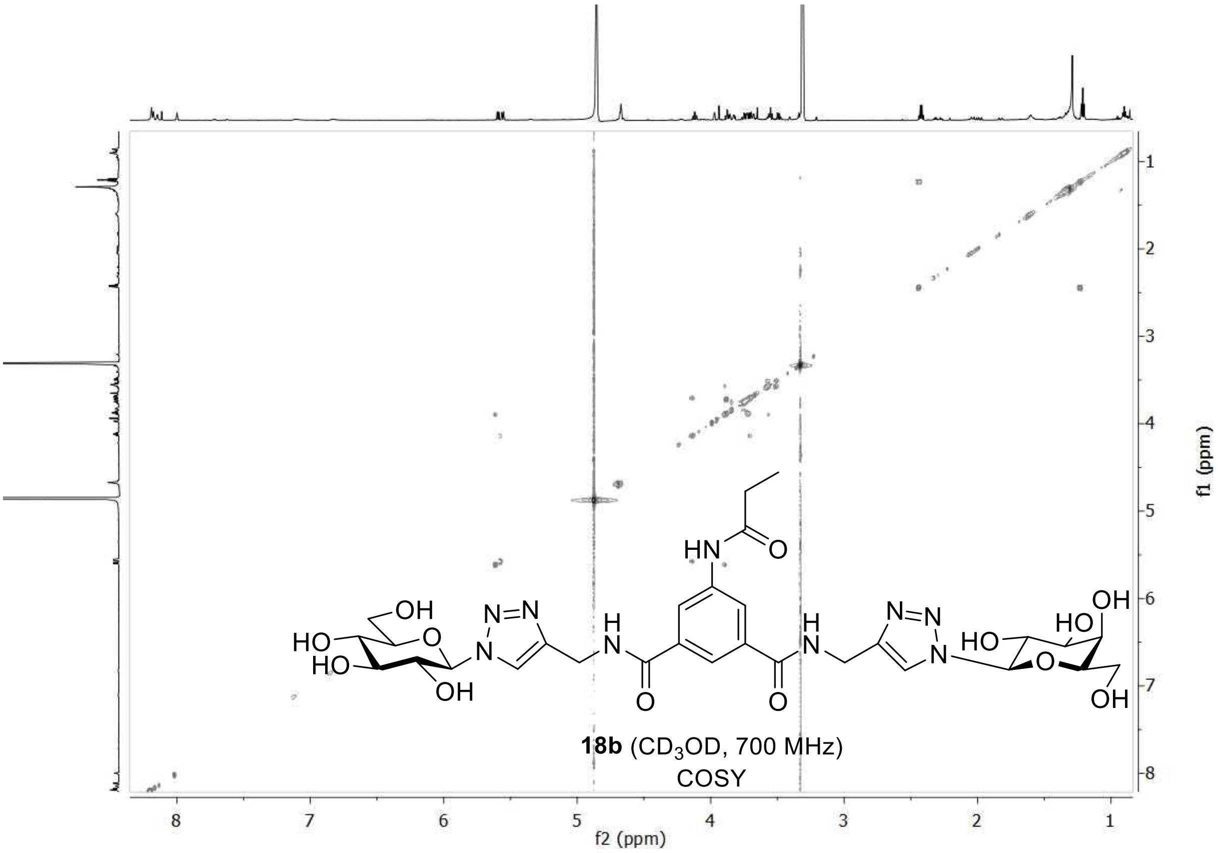

Figure S104. ^{1}H–^{1}H COSY spectrum of **18b**

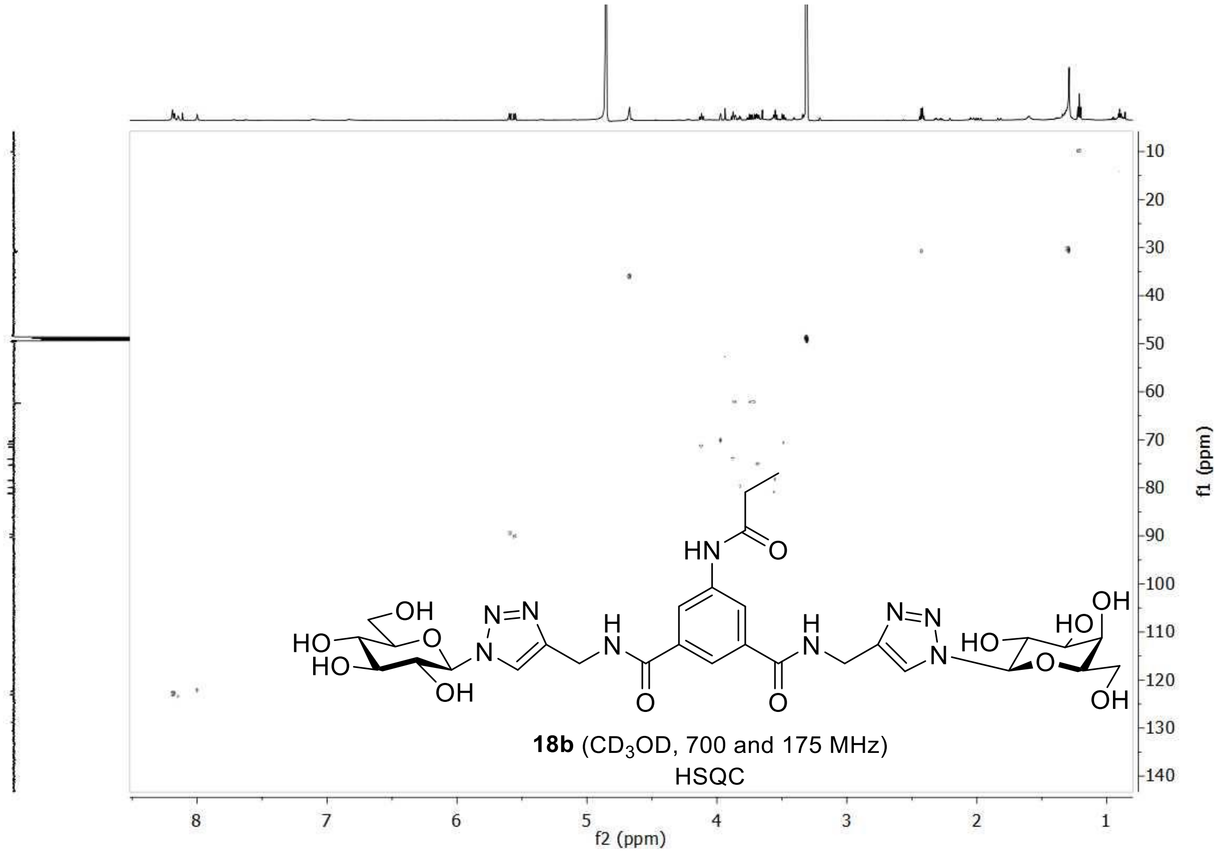

Figure S105. ^{1}H–^{13}C HSQC spectrum of **18b**

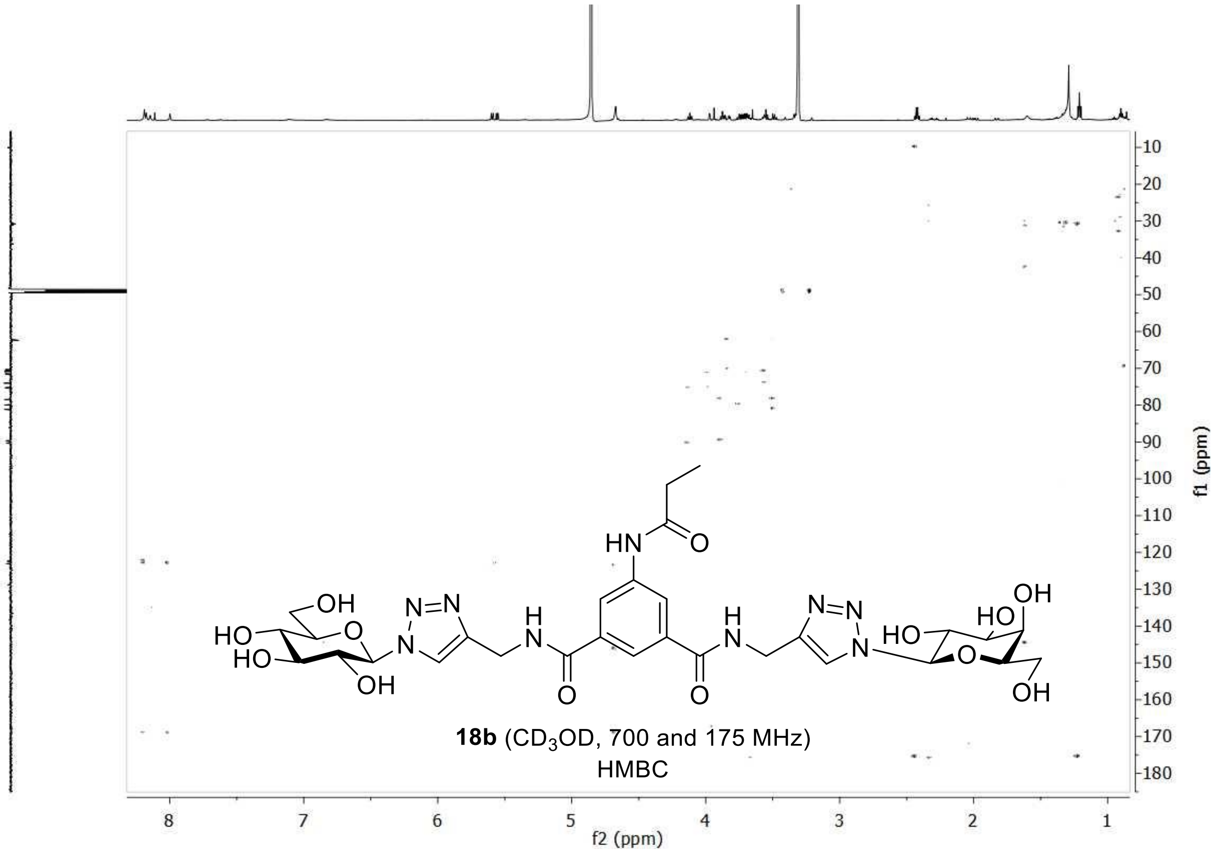

Figure S106. ^{1}H–^{13}C HMBC spectrum of **18b**

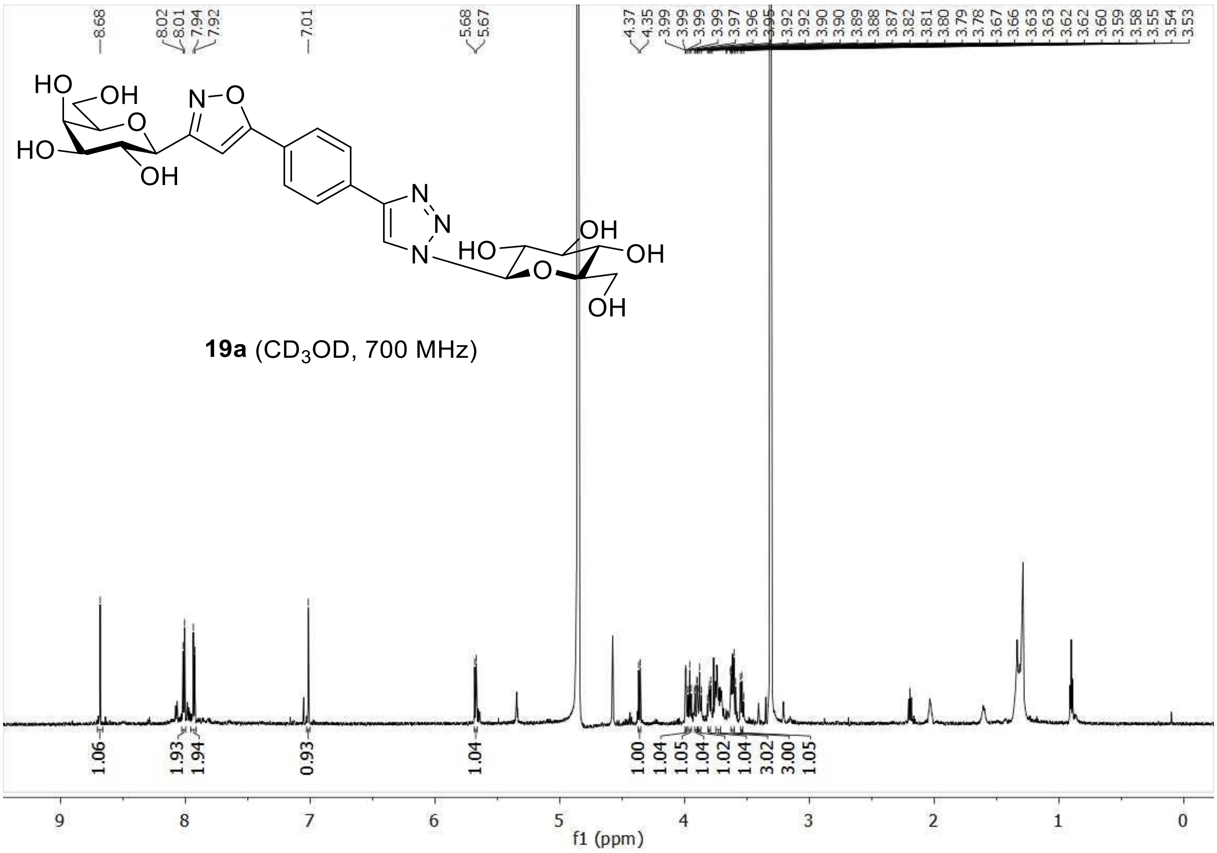

Figure S107. ^{1}H NMR spectrum of **19a**

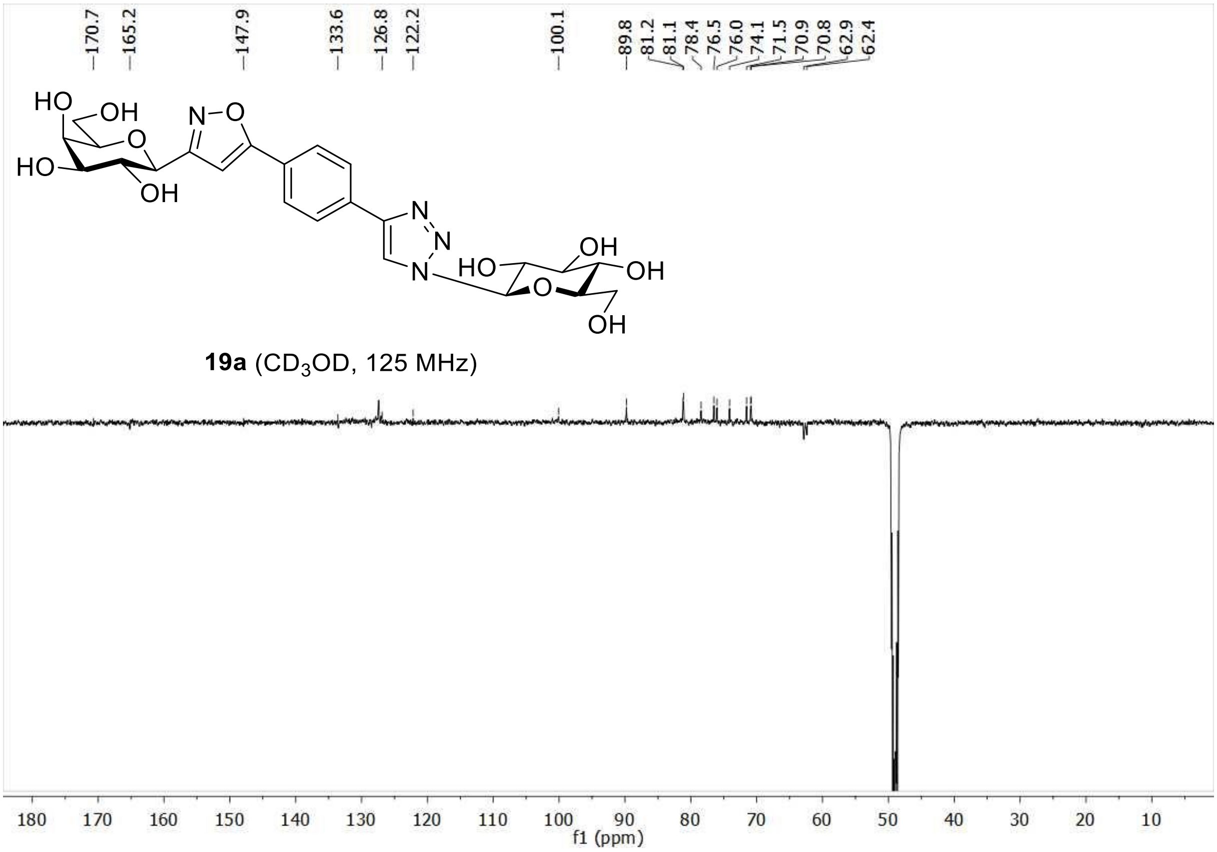

Figure S108. ^{13}C NMR spectrum of **19a**

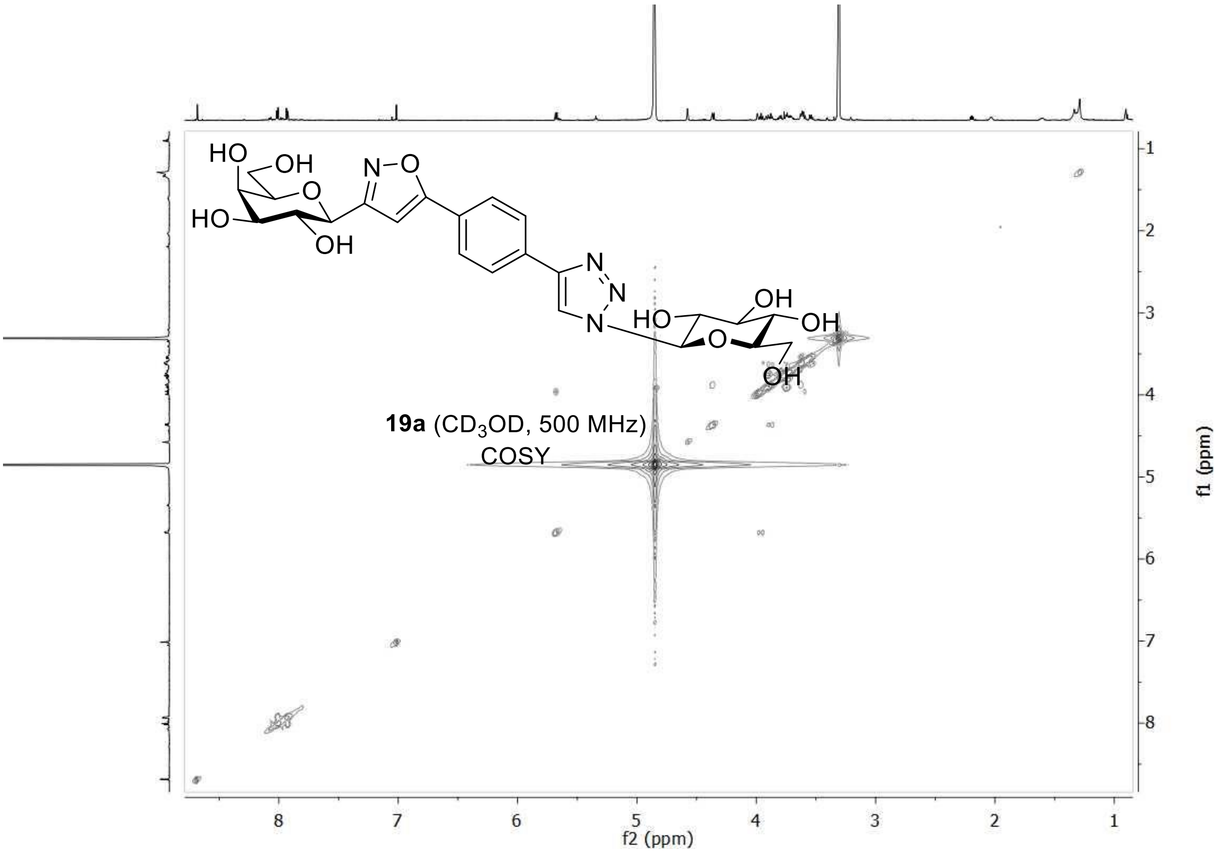

Figure S109. ^{1}H–^{1}H COSY spectrum of **19a**

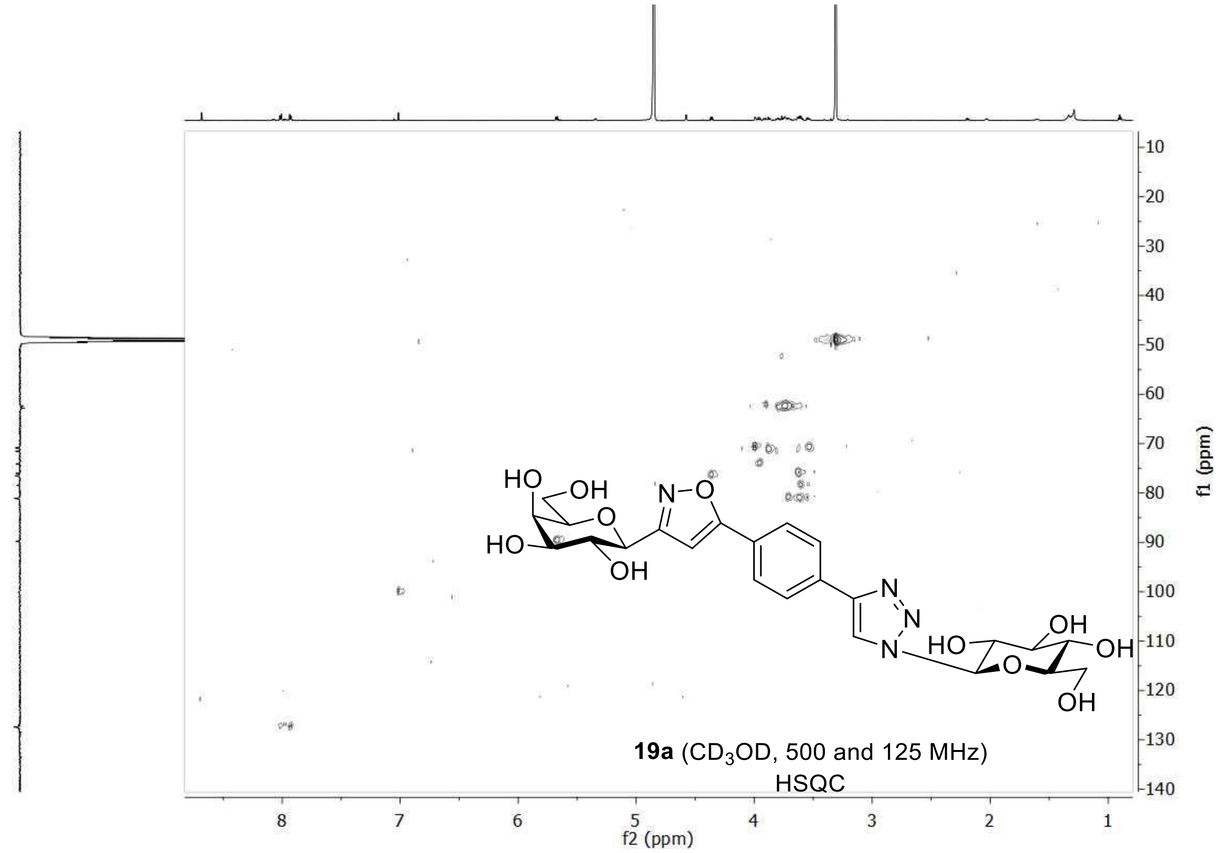

Figure S110. ^{1}H–^{13}C HSQC spectrum of **19a**

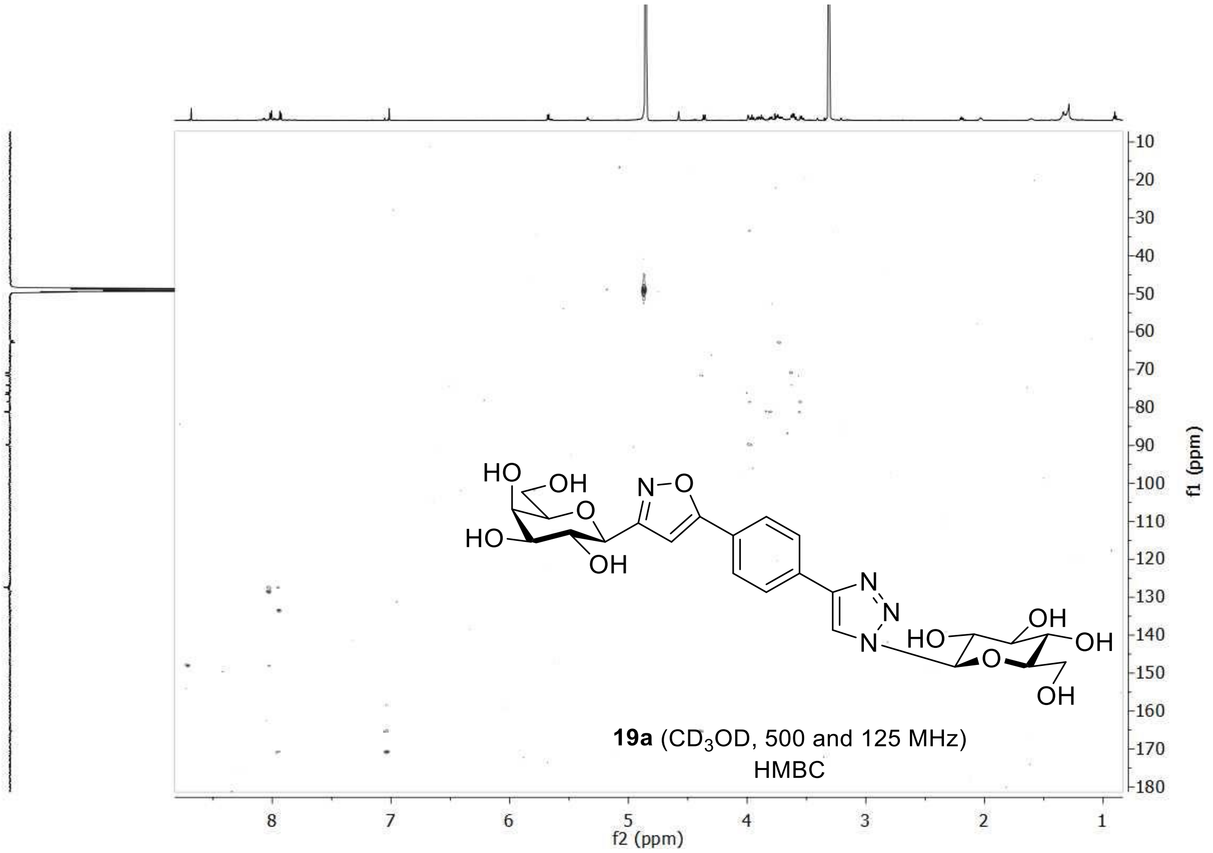

Figure S111. ^{1}H–^{13}C HMBC spectrum of **19a**

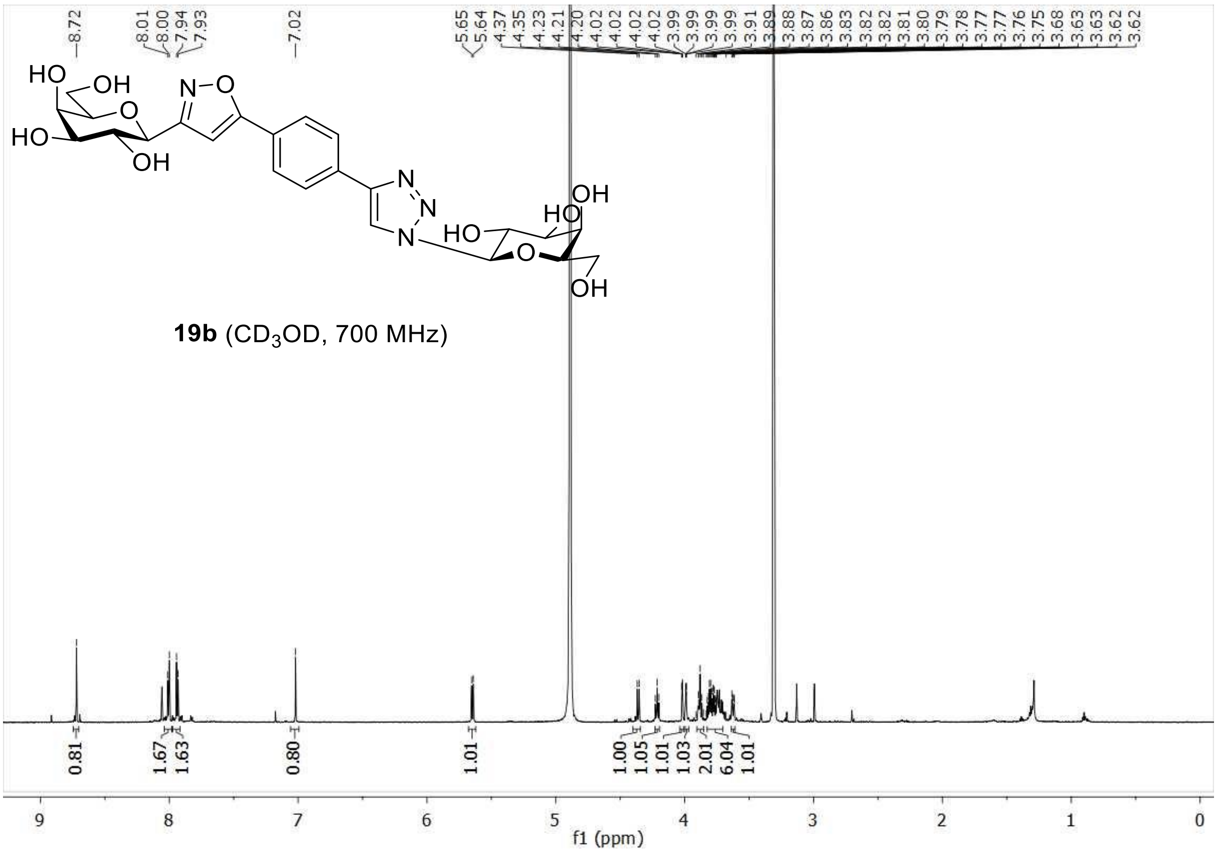

Figure S112. ^{1}H NMR spectrum of **19b**

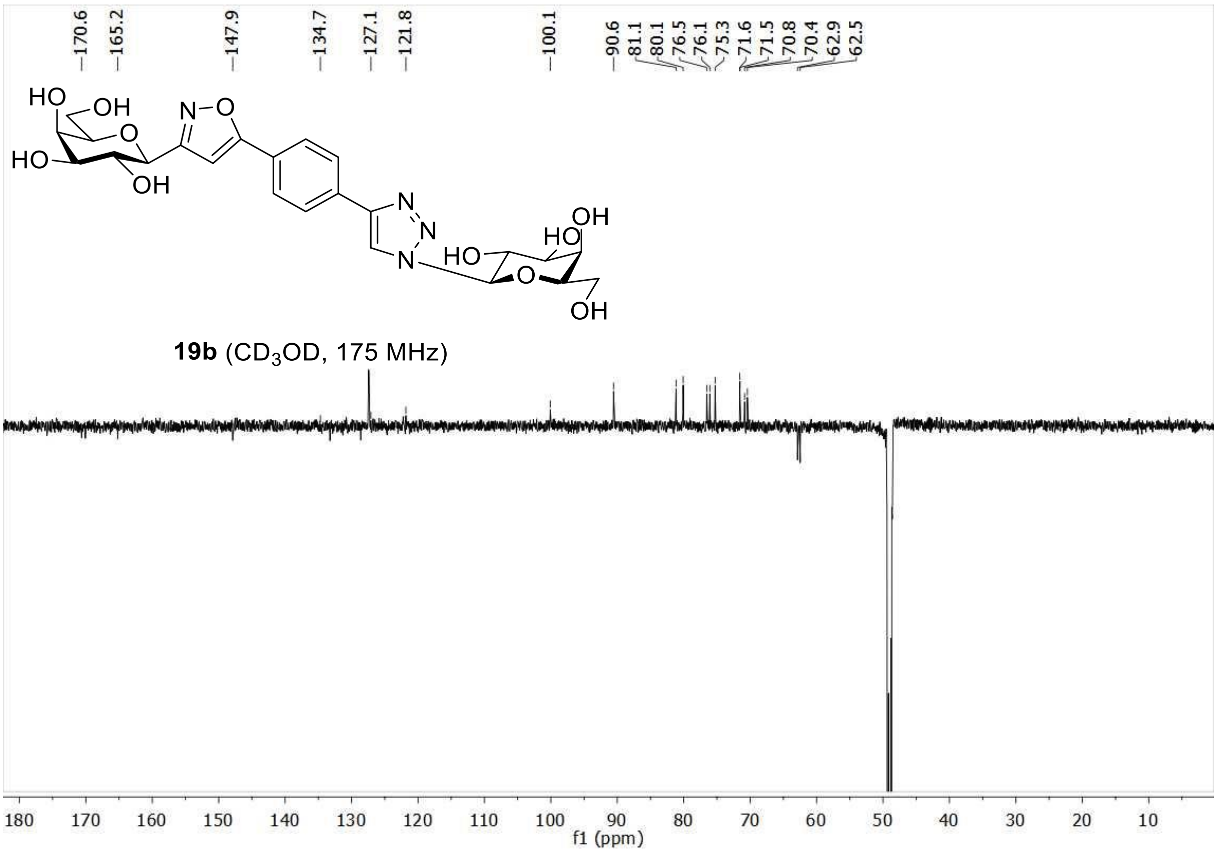

Figure S113. ^{13}C NMR spectrum of **19b**

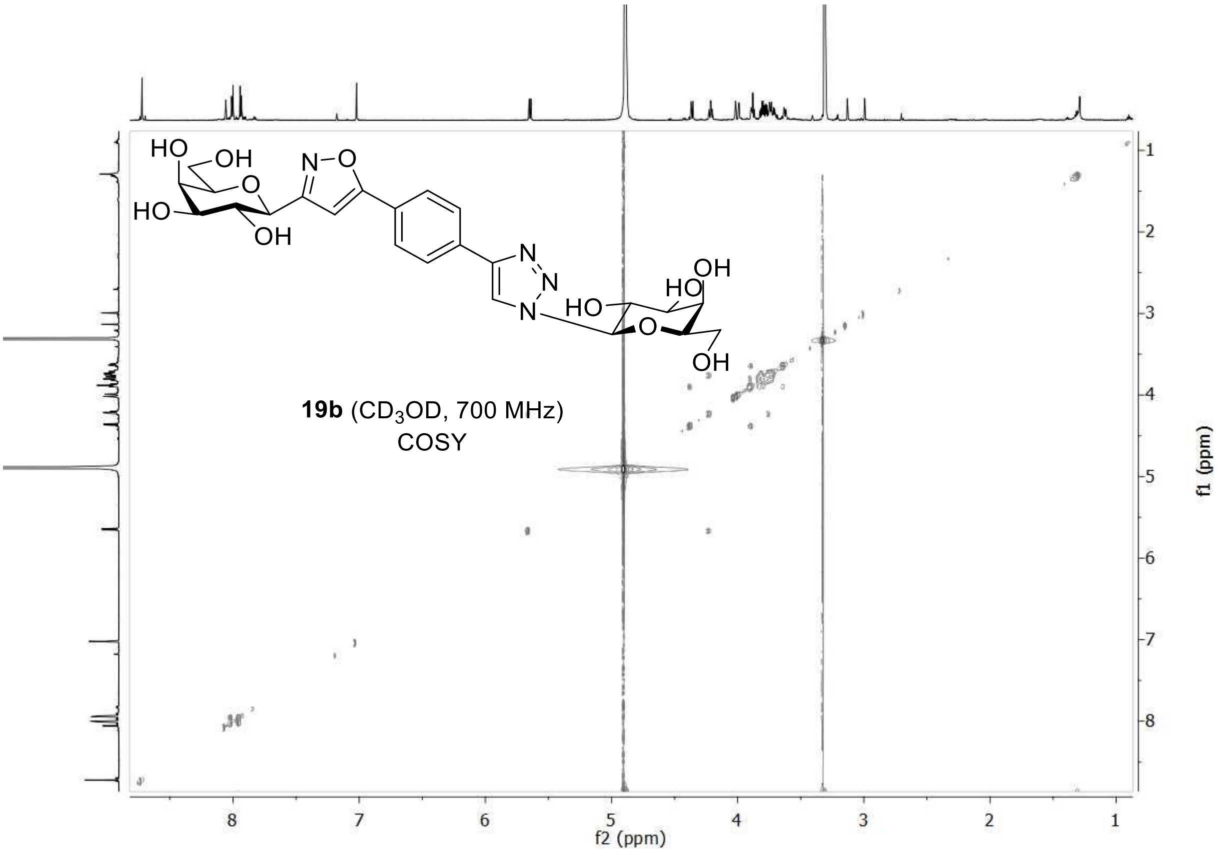

Figure S114. ^{1}H–^{1}H COSY spectrum of **19b**

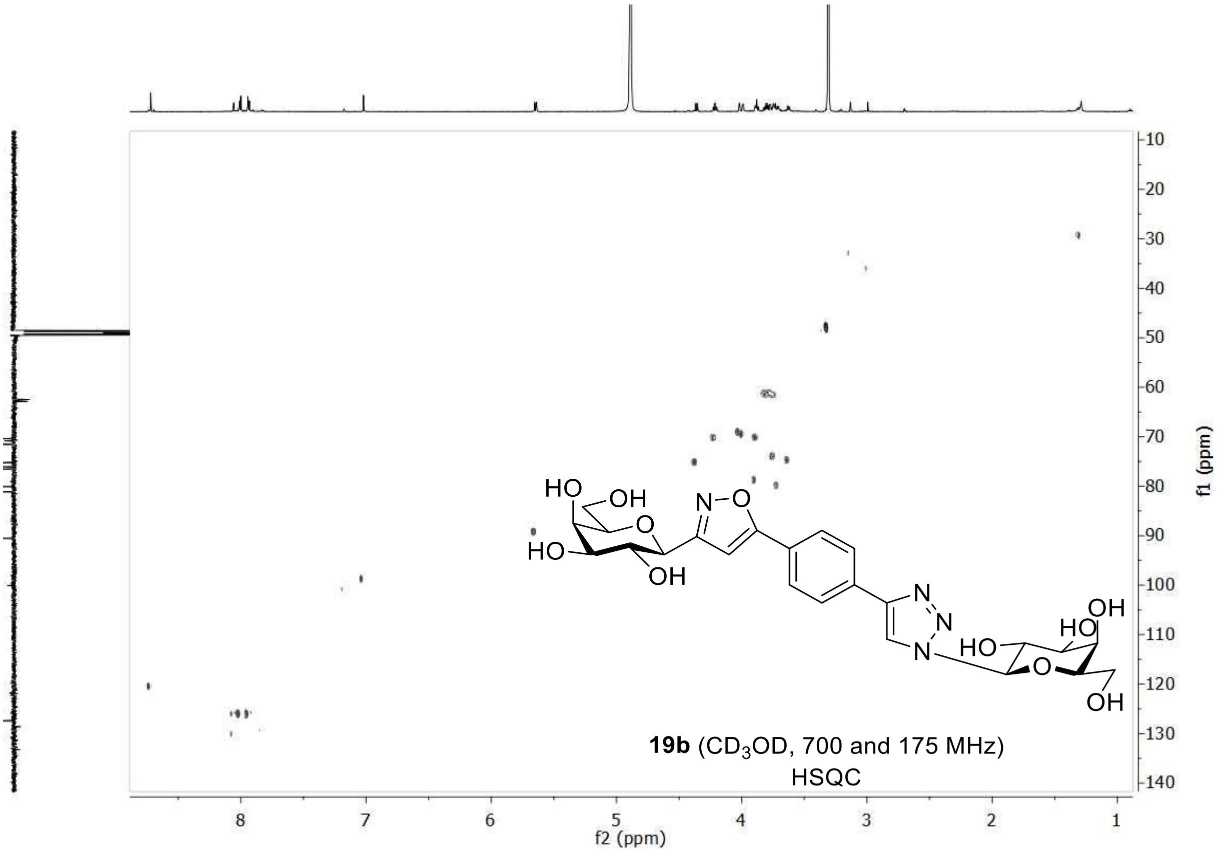

Figure S115. ^{1}H–^{13}C HSQC spectrum of **19b**

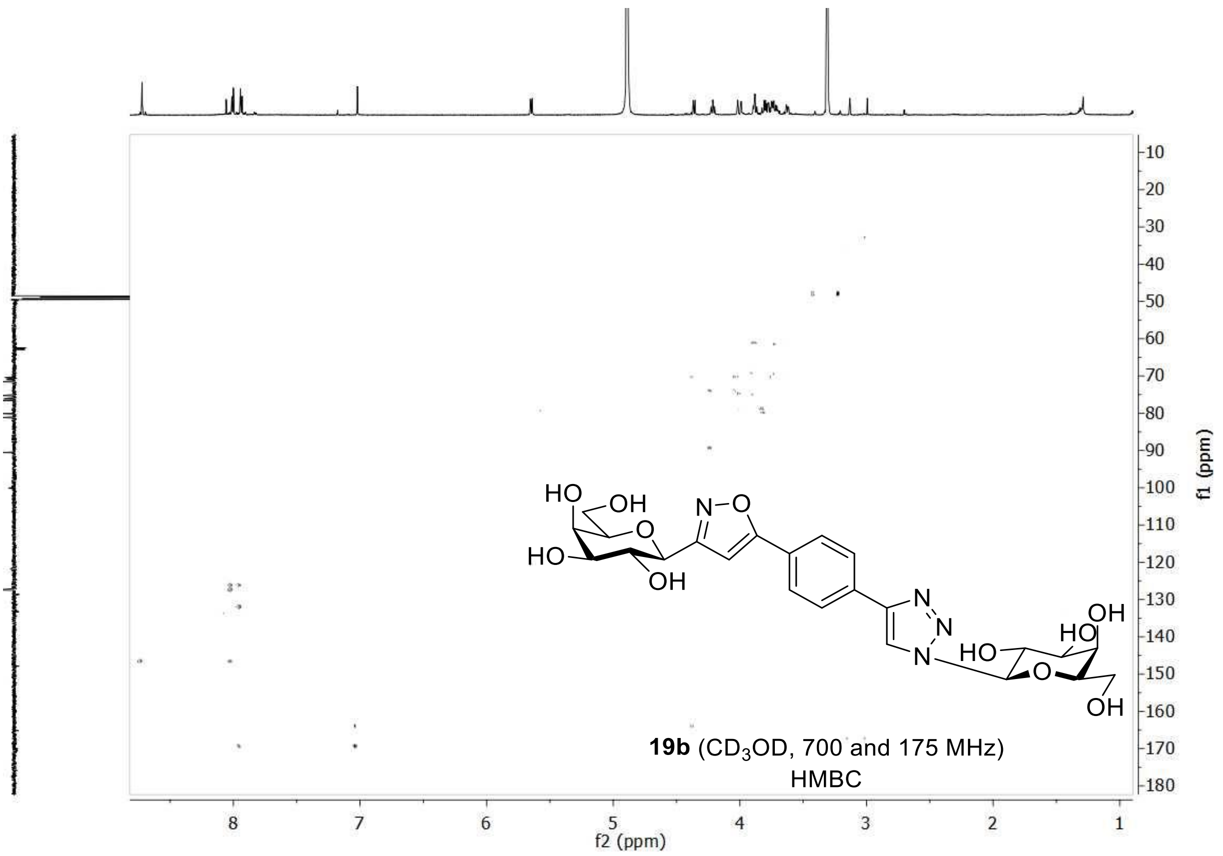

Figure S116. ^{1}H–^{13}C HMBC spectrum of **19b**

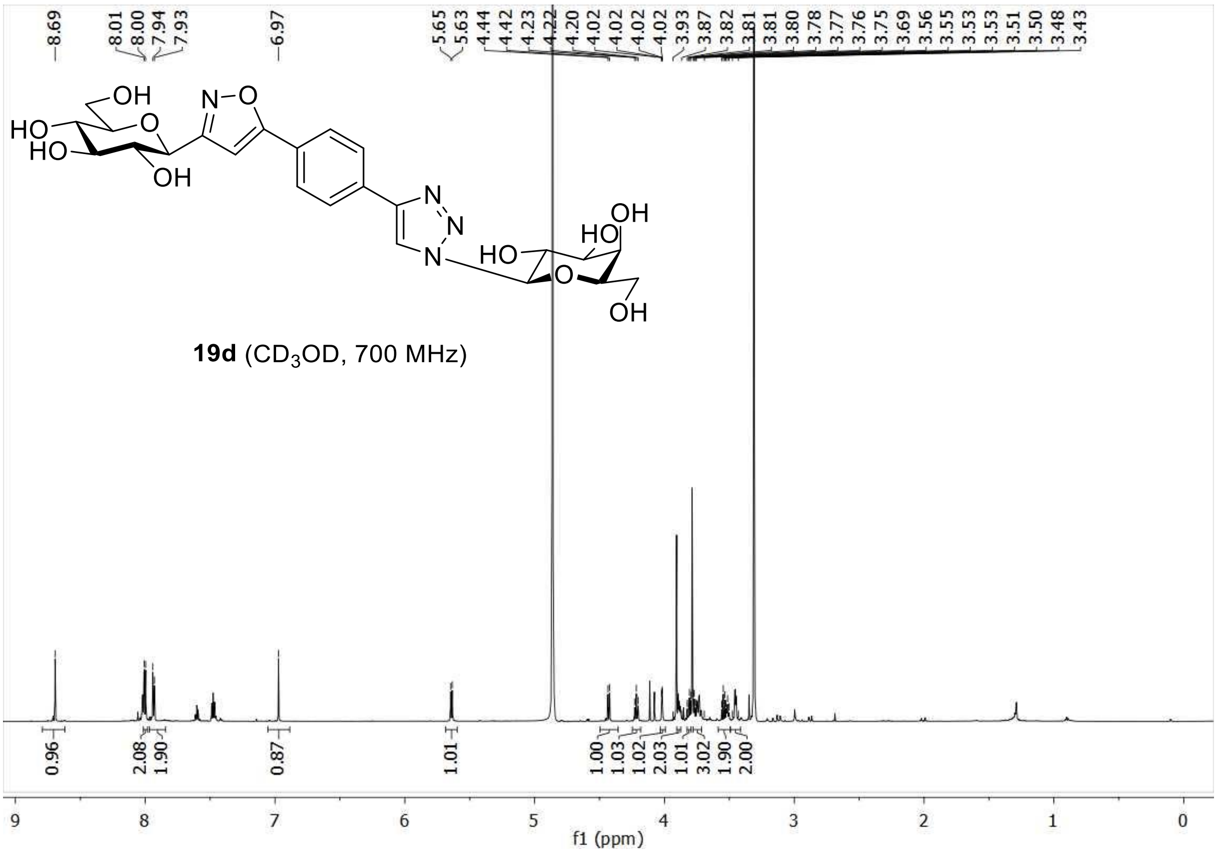

Figure S117. ^{1}H NMR spectrum of **19d**

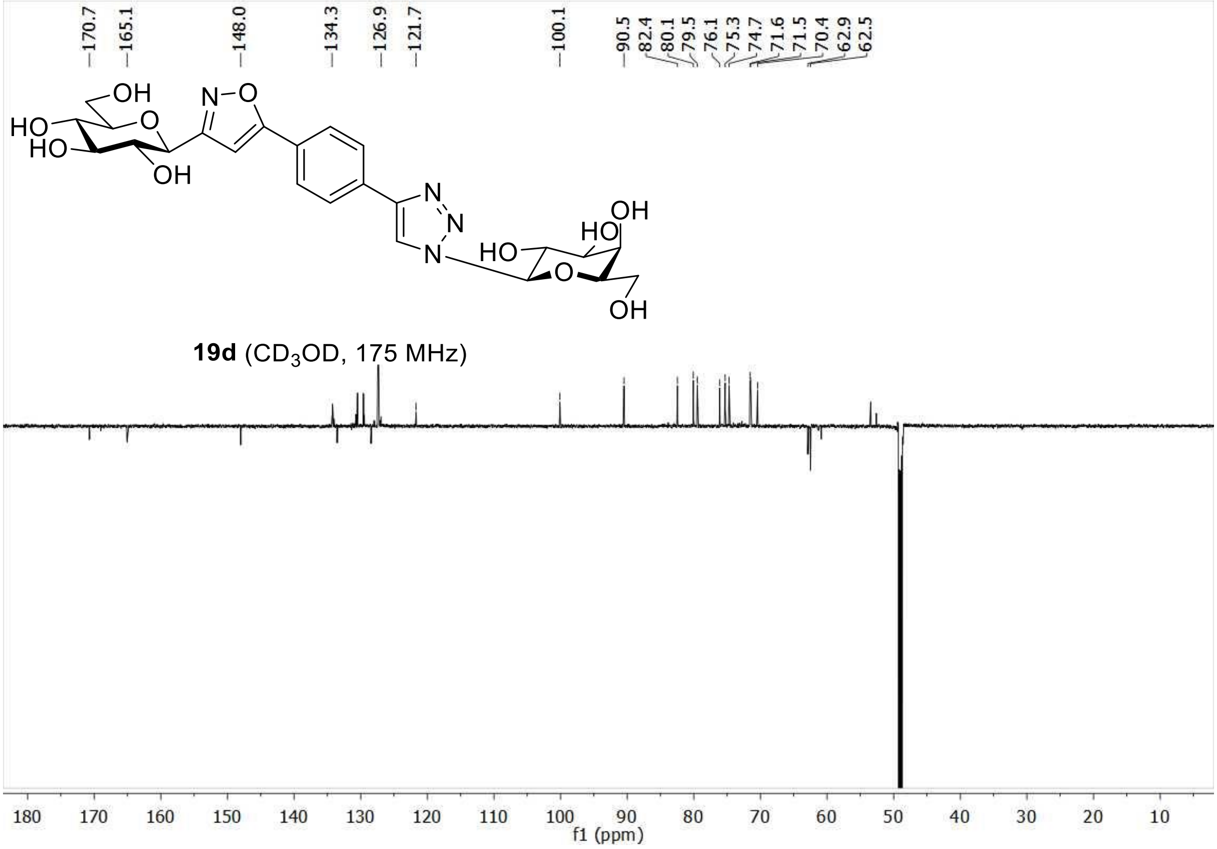

Figure S118. ^{13}C NMR spectrum of **19d**

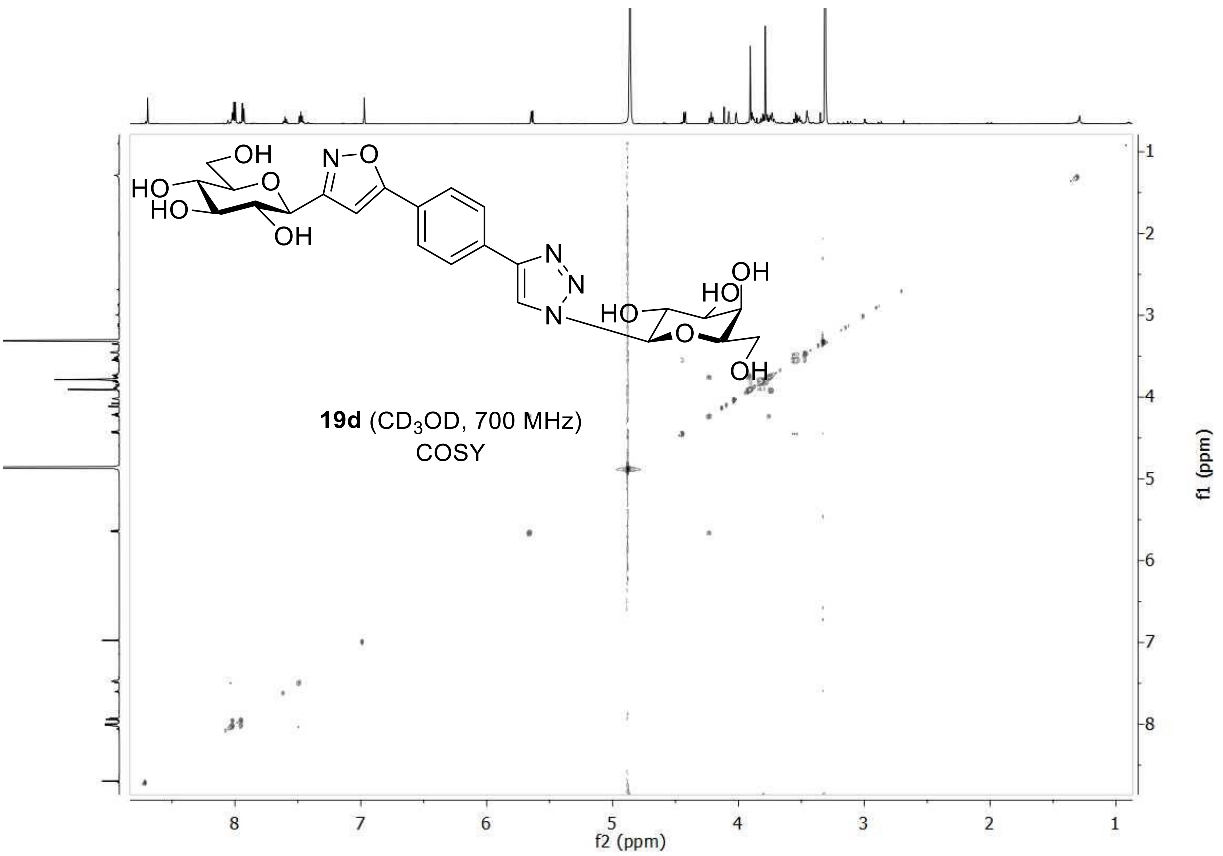

Figure S119. ^{1}H–^{1}H COSY spectrum of **19d**

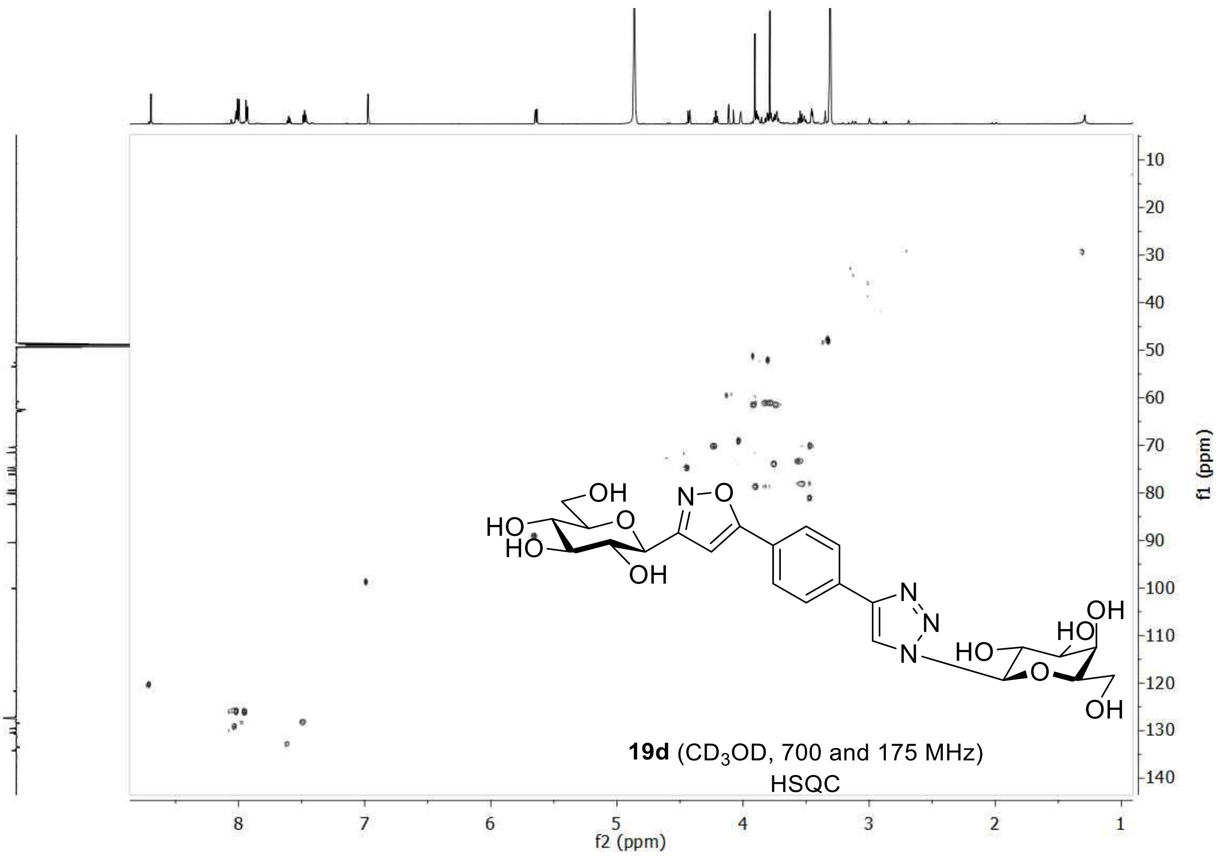

Figure S120. ^{1}H–^{13}C HSQC spectrum of **19d**

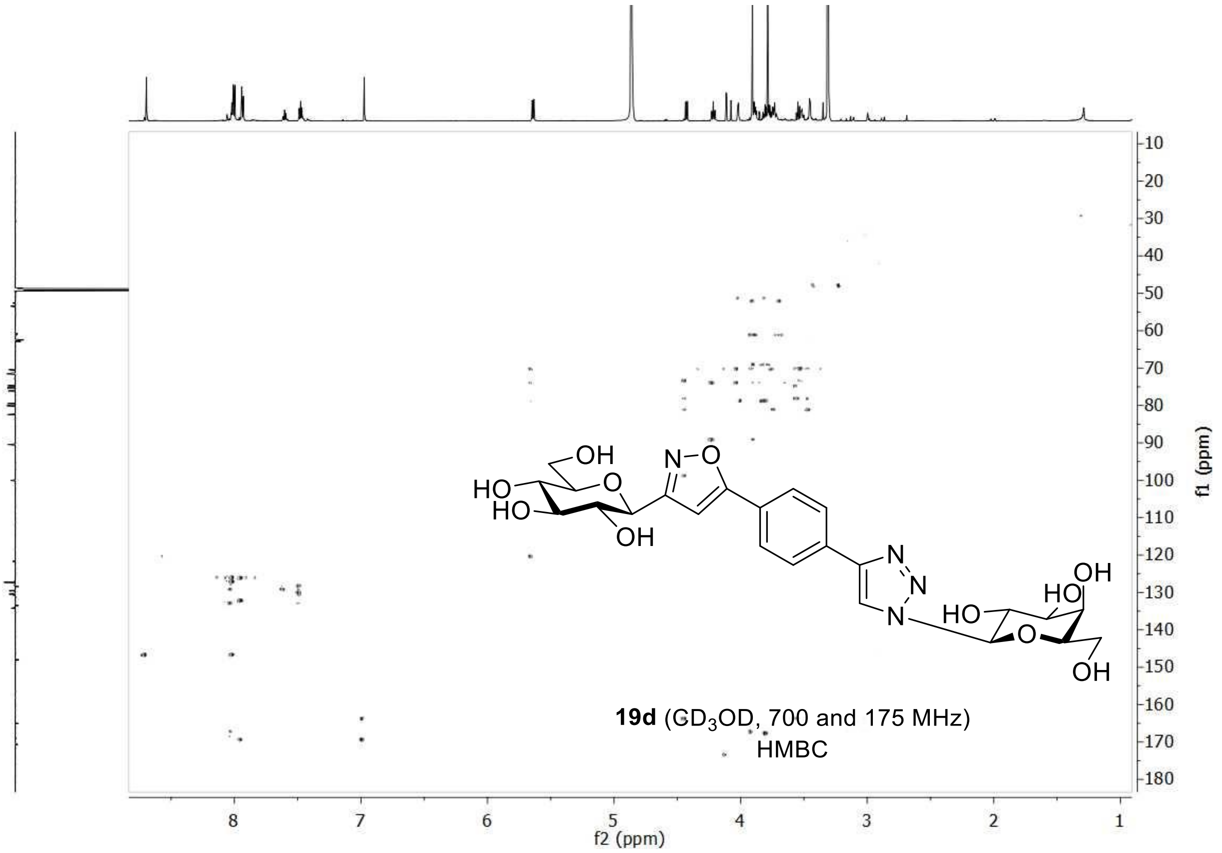

Figure S121. ^{1}H–^{13}C HMBC spectrum of **19d**

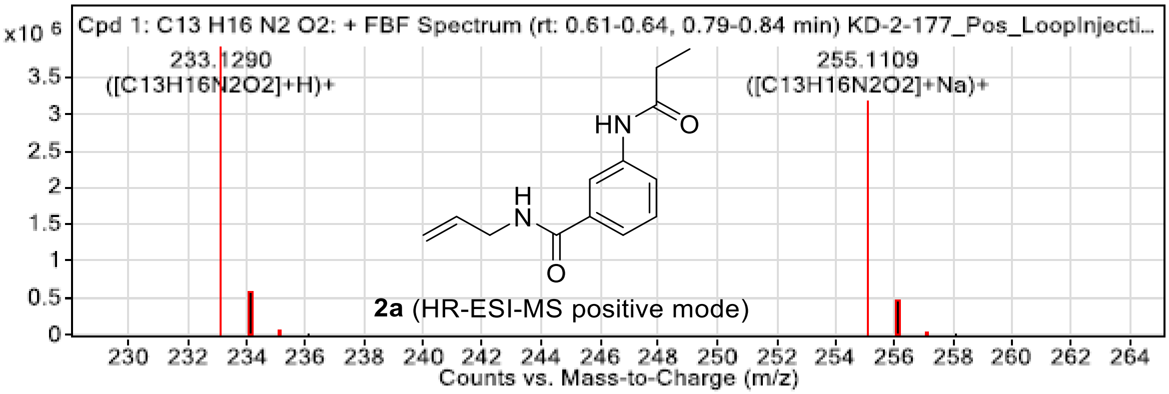

Figure S122. HR-ESI-MS spectrum of **2a**

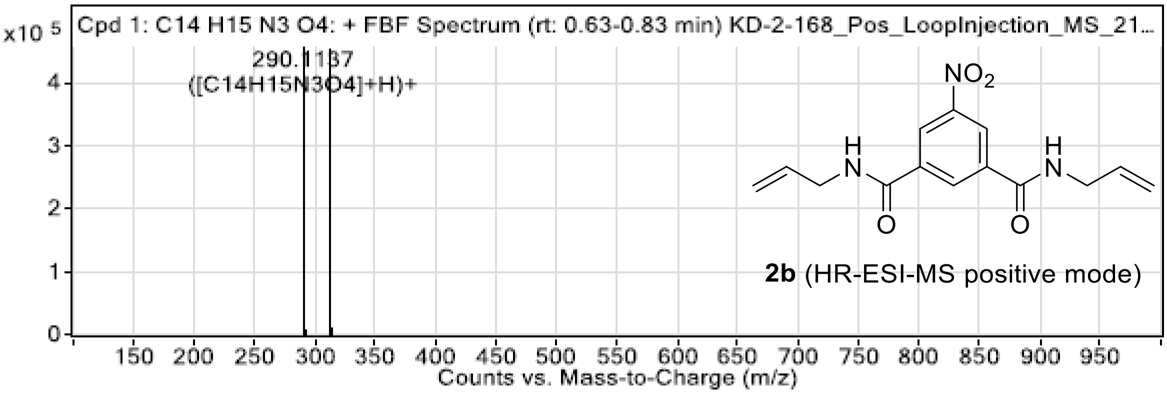

Figure S123. HR-ESI-MS spectrum of **2b**

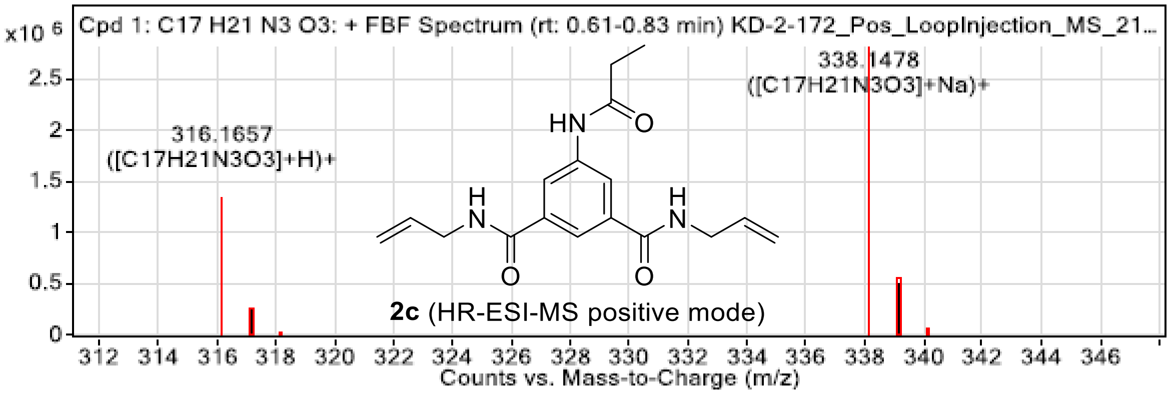

Figure S124. HR-ESI-MS spectrum of **2c**

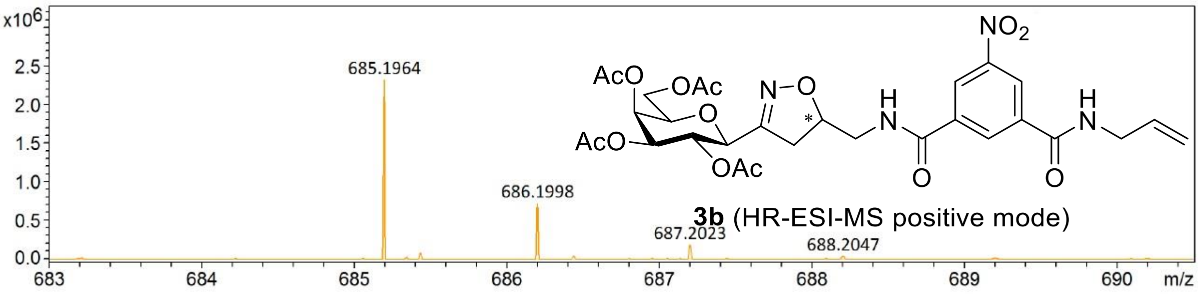

Figure S125. HR-ESI-MS spectrum of **3b**

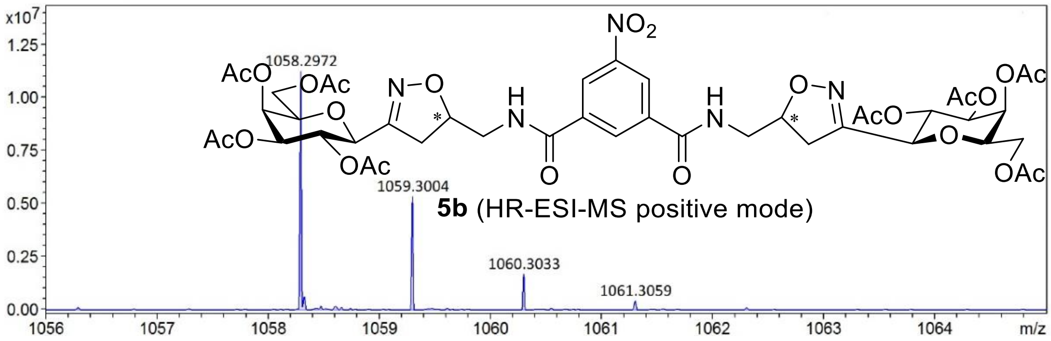

Figure S126. HR-ESI-MS spectrum of **5b**

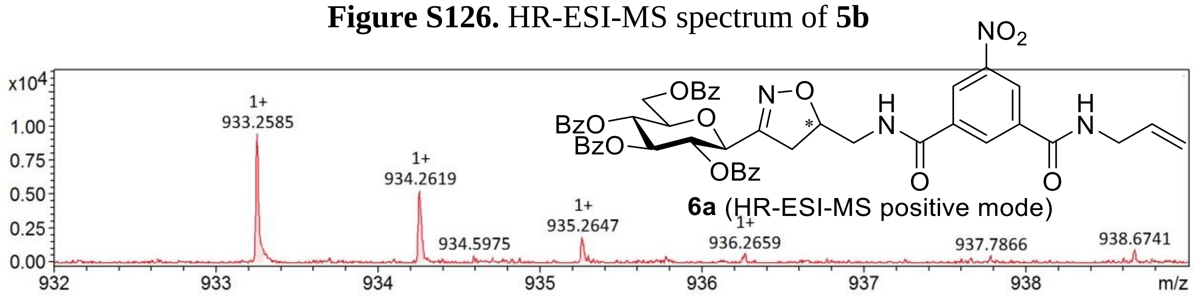

Figure S127. HR-ESI-MS spectrum of **6a**

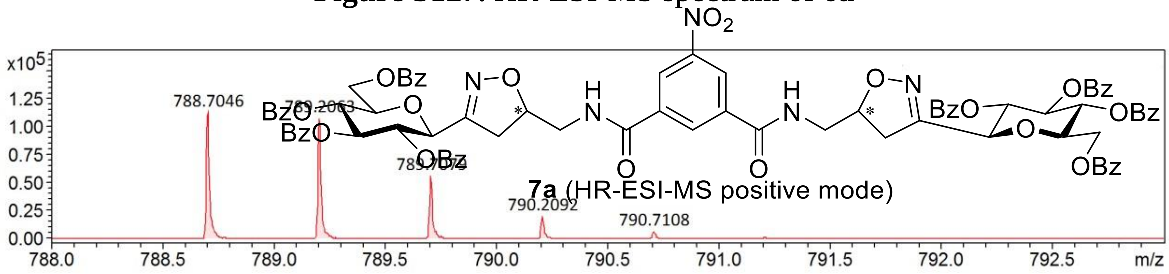

Figure S128. HR-ESI-MS spectrum of **7a**

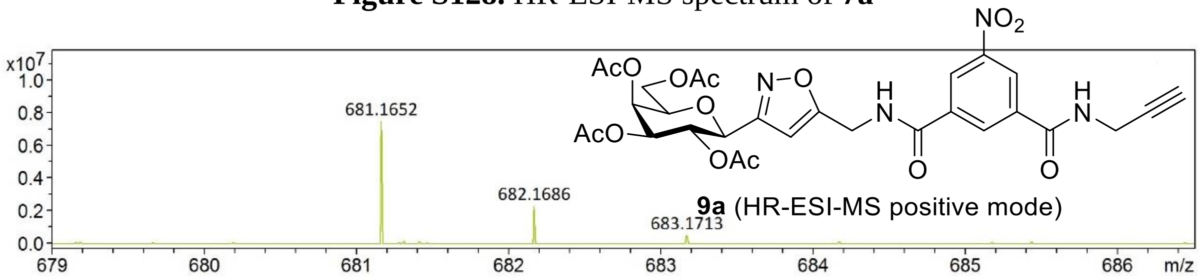

Figure S129. HR-ESI-MS spectrum of **9a**

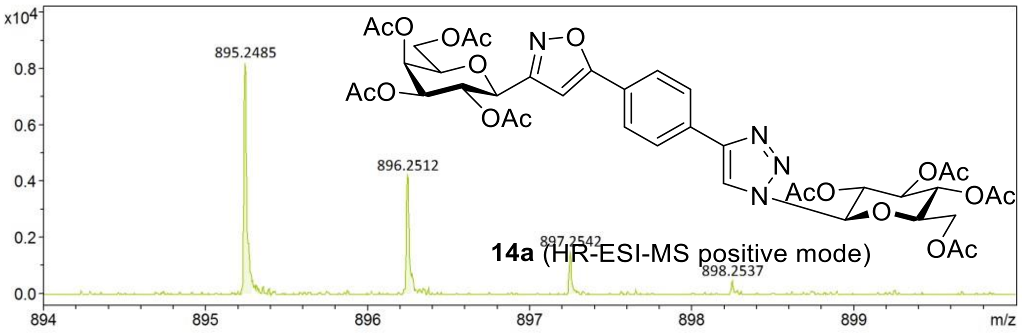

Figure S130. HR-ESI-MS spectrum of **14a**

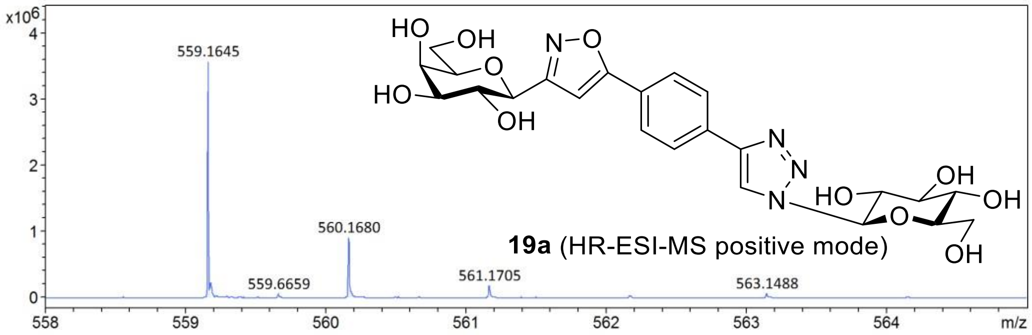

Figure S131. HR-ESI-MS spectrum of **19a**

References

1. Tóth, M.; Somsák, L., Preparation of 2,6-anhydro-aldose acylhydrazones, -semicarbazones and -oximes from 2,6-anhydro-aldononitriles (glycosyl cyanides). *Carbohydr. Res.* **2003**, *338* (12), 1319–1325. https://doi.org/10.1016/S0008-6215(03)00163-0.
2. Baker, K. W. J.; March, A. R.; Parsons, S.; Paton, R. M.; Stewart, G. W., 3,4-Dipyranosyl-1,2,5-oxadiazole 2-oxides: synthesis and X-ray structure. *Tetrahedron.* **2002**, *58* (42), 8505–8513. https://doi.org/10.1016/S0040-4020(02)01023-2.
3. Kaszás, T.; Szakács, B.; Bertalan, M.; Blága, T.; Hameed, F.; Lengyel, Á.; Saifi, S.; Juhász-Tóth, É.; Varga, L. A.; Docsa, T.; Sipos, A.; Bai, P.; Ábrahám, A.; Kiss-Szikszai, A.; Kun, S.; Kiss, Gy. A.; József, J.; Juhász, L.; Tóth, M., Regioselective synthesis of 5-substituted 3-(β-D-glycopyranosyl)isoxazoles and -isoxazolines by 1,3-dipolar cycloaddition as potential anticancer agents and glycogen phosphorylase inhibitors. *Int. J. Mol. Sci.* **2025**, *26* (17), 8167. https://doi.org/10.3390/ijms26178167.
4. Doherty, K.; Kessie, K.; Martin, H.; Loughlin, J.; Dulawa, O.; Kasemets, K.; Velasco-Torrijos, T., Synthesis of aromatic glycoconjugates as anti-fungal agents against *Candida* spp. and assessment of their covalent crosslinking capabilities. *Bioorg. Med. Chem.* **2025**, *117*, 118020. https://doi.org/10.1016/j.bmc.2024.118020.
5. Martin, H.; Govern, M. M.; Abbey, L.; Gilroy, A.; Mullins, S.; Howell, S.; Kavanagh, K.; Velasco-Torrijos, T., Inhibition of adherence of the yeast *Candida albicans* to buccal epithelial cells by synthetic aromatic glycoconjugates. *Eur. J. Med. Chem.* **2018**, *160*, 82–93. https://doi.org/10.1016/j.ejmech.2018.10.011.
6. Bertho, A., Über Azidoderivate der Glucose. *Ber. Dtsch. Chem. Ges. (A/B)* **1930**, *63* (4), 836–843. https://doi.org/10.1002/cber.19300630415.
7. Bertho, A.; Maier, J., Die katalytische Hydrierung von Aziden. *Justus. Liebigs. Ann. Chem.* **1932**, *498* (1), 50–61. https://doi.org/10.1002/jlac.19324980104.
8. Martin, H. The synthesis and biological evaluation of anti-adhesion glycoconjugates against opportunistic pathogenic *Candida albicans*. PhD thesis, National University of Ireland Maynooth, Maynooth University, Maynooth, 2019.
9. Zemplén, G.; Pacsu, E., Über die Verseifung acetylierter Zucker und verwandter Substanzen. *Ber. Dtsch. Chem. Ges. (A/B)* **1929**, *62* (6), 1613–1614. https://doi.org/10.1002/cber.19290620640.
